# Cosmin reporting guideline for studies on measurement properties of patient-reported outcome measures and its explanation and elaboration document: translation into Brazilian Portuguese

**DOI:** 10.1007/s11136-026-04282-0

**Published:** 2026-06-05

**Authors:** Guilherme Tavares de Arruda, Caroline B. Terwee, Patricia Driusso, Mariana Arias Avila, Lidwine B. Mokkink

**Affiliations:** 1 Physical Therapy Department, Universidade Federal de Juiz de Fora–UFJF-GV, Avenida Moacir Paleta, 1167 - São Pedro, Governador Valadares, Minas Gerais 35020-360 Brazil; 2 Physical Therapy Department, Universidade Federal de São Carlos – UFSCar, São Paulo, São Carlos, Brazil; 3 Department of Epidemiology and Data Science, Amsterdam UMC, Vrije Universiteit Amsterdam, Amsterdam, Netherlands; 4 Amsterdam Public Health research institute, Methodology, Amsterdam, Netherlands

**Keywords:** Reporting recommendations, Measurement properties, Patient-reported outcome measures, COSMIN, Translation

## Abstract

**Purpose:**

The COSMIN Reporting Guideline 2.0 and its Explanation & Elaboration document have been published to guide researchers in reporting studies on measurement properties of patient-reported outcome measures (PROMs). We aimed to translate the COSMIN Reporting Guideline 2.0 and its Explanation & Elaboration document to report studies on measurement properties of PROMs into Brazilian Portuguese.

**Methods:**

We followed an adapted four-step methodology, including artificial intelligence (AI) forward-translation and back-translation, and appraisal of all written reports by the developers/committee.

**Results:**

The AI produced uncommon terms, later standardized to usual Brazilian Portuguese expressions. Back-translation showed minor discrepancies without altering meaning. Expert review corrected grammar and refined terminology.

**Conclusion:**

The COSMIN Reporting Guideline 2.0 and its Explanation & Elaboration document have been translated into Brazilian Portuguese and can overcome language barriers and guide Brazilian-speaking researchers, clinicians, patients, and policymakers in reporting studies on measurement properties of PROMs. Both documents are available on the COSMIN website.

**Supplementary Information:**

The online version contains supplementary material available at 10.1007/s11136-026-04282-0.

## Abstract (plain language summary)

The COSMIN Reporting Guideline 2.0 Explanation & Elaboration document was originally developed in English to help users apply the guideline correctly. For this study, we translated the guideline and its Explanation & Elaboration document into Brazilian Portuguese to improve accessibility and usability in Portuguese-speaking contexts. The translated Explanation & Elaboration document offers detailed explanations, rationales, and examples of good reporting for each guideline item, helping researchers and clinicians understand why each item should be reported and how to report it adequately. The document includes an introduction to the guideline and explanations of general and measurement property-specific items. These explanations are structured into sections that cover essential elements and item examples. Making this translated Explanation & Elaboration document available aims to facilitate the comprehensive and transparent reporting of studies on the measurement properties of patient-reported outcome measures in Brazil.

**Supplementary Information:**

The online version contains supplementary material available at 10.1007/s11136-026-04282-0.

## Introduction

Patient-Reported Outcome Measures (PROMs) are tools frequently used in clinical practice and research to reflect the patient’s perspective (e.g., feelings, functions, etc.). The quality of PROMs depends on whether their measurement properties (i.e., validity, reliability, and responsiveness) are sufficient [1]. In addition, well-reported studies provide enough information, enabling researchers, clinicians, patients and policymakers to understand the methods employed, accurately interpret the results, and draw correct conclusions [2]. In recent years, increased attention has been given to the quality of measurement instruments [3], and this movement has also gained strength in Brazil and other Portuguese-speaking countries, with initiatives such as the translation of the COSMIN taxonomy and the COSMIN checklist (cosmin.nl/research-publications/), as well as the successful development of collaborative research networks such as the “Grupo de Estudo e discussão sobre Propriedades de Medida para países de Língua Portuguesa” (GEProM).

Recently, the COSMIN (COnsensus-based Standards for the selection of health Measurement INstruments) initiative updated the COSMIN Reporting Guideline 2.0 [4] and developed the Explanation & Elaboration document for this guideline in English [5]. The COSMIN Reporting Guideline 2.0 contains a set of 68 items, divided into general items and measurement property specific items, that can guide researchers in reporting studies on measurement properties of PROMs [4]. The Explanation & Elaboration document of the COSMIN Reporting Guideline 2.0 includes the items of the guideline, and provides explanations, rationales, and examples for good reporting for each item of the COSMIN Reporting Guideline 2.0, thus making it easier for users to apply the guideline [5].

However, widespread use and understanding of this guideline face significant challenges in Brazil due to language barriers. Portuguese is the official and predominant language in Brazil, as well as the primary language of communication in all social, cultural and economic areas of the country. In contrast, according to the British Council, only 5% of the Brazilian population, including researchers, has knowledge of English and only 1% is fluent [6]. Estimates indicate that English proficiency is limited to certain segments of the population, typically in urban areas, professionals working in sectors related to international trade, technology, and education [6, 7].

Translating the COSMIN Reporting Guideline 2.0 [4] and its Explanation & Elaboration document [5] into Brazilian Portuguese helps overcome language barriers and allows for wider dissemination of critical scientific knowledge, as many researchers, clinicians, and students in Brazil are not fully proficient in English. This is particularly important in promoting the adoption of international standards and improving the quality of research and clinical practice throughout the country. By making these tools more accessible, it will ensure that the evidence and recommendations presented in guidelines can be effectively implemented, improving the use of PROMs with sufficient measurement properties in Brazil. Thus, the aim of this study was to translate the COSMIN Reporting Guideline 2.0 and its Explanation & Elaboration document to report studies on measurement properties of PROMs into Brazilian Portuguese.

## Methods

To translate the COSMIN Reporting Guideline 2.0 and its Explanation & Elaboration document into Brazilian Portuguese, we adapted the four steps of Beaton et al. [8]. In our adaptation, we used the ChatGPT^®^ 4.0 via the web interface (chat.openai.com) as one forward-translation, and a Brazilian professional translator as a second translator. ChatGPT^®^ 4.0 is an open website based on artificial intelligence (AI) and can be used to translate texts and documents into multiple languages. We also used translated terms into Brazilian Portuguese from the COSMIN taxonomy, published on the COSMIN website (cosmin.nl/research-publications/). The translation and backtranslation were conducted in March and May of 2025, respectively.

The COSMIN Reporting Guideline 2.0 is an integral part of the Explanation & Elaboration document. Therefore, the two documents were not translated in exactly the same way. First, we translated the guideline using rigorous steps to ensure consistent translation of all items. Then, we incorporated these exact translations of the guideline items into the Explanation & Elaboration document. However, because the Explanation & Elaboration is much more extensive and descriptive, applying the same thorough, multi-step translation procedures to the entire document was not feasible. Instead, the additional explanatory content (such as rationales and examples) was translated to ensure consistency with the translated guideline items.

In the first step (direct translation), an author (GTA) of this paper copied and pasted the guideline and the Explanation & Elaboration document sentence by sentence into the ChatGPT^®^ 4.0 translator box while another Brazilian translator independently translated the documents. In the second step (translation synthesis), the same author (GTA) compared the translations of the documents from the previous step, resulting in one synthesized forward translated version of each document. Distinctions between the two forward translations were decided by consensus between the two authors (GTA, MAA). In the third step (back-translation), the synthesized forward translated version of the COSMIN Reporting Guideline 2.0 was back-translated into English in ChatGPT^®^ 4.0. Discrepancies between the back-translated version and the original English document were systematically identified and analyzed. The discrepancies were classified as semantic, idiomatic, or conceptual and reviewed by three authors (GTA, PD, MAA) to determine if they reflected acceptable linguistic variation or required modification. When discrepancies indicated potential shifts in meaning, the corresponding Portuguese translations were revised to better align with the original intent.

To translate the Explanation & Elaboration document, we used the translations of the items for which we reached consensus in the guideline translation process. The additional text (i.e., explanations, rationales, and good examples) was initially forward-translated once into Brazilian Portuguese by one author of this study (GTA). To strengthen the rigor of this streamlined approach, the content was subjected to targeted consistency checks against the finalized guideline items and internal reviews during the synthesis process. This step yielded a forward-translated version of the Explanation & Elaboration document, which was then submitted to the expert committee for evaluation. In the fourth step (committee of judges), we invited 11 Brazilian researchers to review the translations of the documents. The judges reviewed the translated documents independently and were instructed to assess three predefined domains: (1) clarity and comprehensibility, (2) conceptual equivalence with the original English version, and (3) consistency of terminology throughout the guideline and Explanation & Elaboration document. Evaluations were performed individually using structured written feedback. The judges were asked to identify unclear or potentially misleading passages and suggest revisions when necessary. The research team collected and compiled all feedback, and discrepancies or conflicting suggestions were resolved through discussion among the authors until consensus was reached. Revisions were made accordingly, prioritizing conceptual accuracy and consistency with the original documents. The judges were particularly attentive to potential subtle shifts in meaning that could affect interpretation. To mitigate the risk of interpretive drift resulting from the streamlined translation process of the Explanation & Elaboration document, we implemented additional quality assurance procedures targeting the explanatory content (i.e., rationales and examples). First, we translated all explanatory sections explicitly referencing the corresponding guideline items to preserve conceptual alignment. Second, during the synthesis phase, we reviewed the translated explanatory content to ensure consistency in terminology and meaning across both documents. Third, during the expert committee review, reviewers were instructed to identify subtle shifts in meaning that could affect interpretation, especially in examples and justifications. In the end, all versions of the documents and reports from each step were sent to the COSMIN developer for approval.

The prompts used in ChatGPT^®^ 4.0 for translating and back-translating documents are presented in Box 1. The prompts were standardized and applied consistently throughout the translation process. No adaptive learning or feedback loops were used. The total number of queries corresponded to the number of text segments translated, i.e., a sentence-by-sentence translation of the full documents. The prompts were designed to reflect Brazilian Portuguese language use to ensure contextual and cultural appropriateness.

**Box 1:** Prompts used for translating and back-translating the COSMIN reporting guideline 2.0 and its Explanation & elaboration document

For translating: “Please translate the following text from American English to Brazilian Portuguese, maintaining the meaning and comprehension of the original text. Keep only certain acronyms in English, such as PROMs and others. Please translate everything, including quoted text and examples, even if they are direct quotes; please double check that all examples are translated. Output only the translated text, no additional comments. This is the text:”
For back-translating: “Please translate the following text from Brazilian Portuguese to American English, maintaining the meaning and comprehensibility of the original text. Keep only certain acronyms in English, such as PROMs and others. Please translate everything, including quoted text and examples, even if they are direct quotes; please double check that all examples are translated. Output only the translated text, no additional comments. This is the text:”

All translations were conducted using the default system settings. No manual adjustments were made to parameters such as temperature or randomness. Each text segment was translated once, with no multiple runs or iterative prompt refinement. One researcher (GTA) evaluated AI-generated translations through a structured comparison with human translations. Since the study focused on the measurement properties of PROMs, topic randomization was not used.

Management and reporting of AI use will follow the METRICS checklist (Model, Evaluation, Timing, Range/Randomization, Individual factors, Count, and Specificity of prompts and language) [9].

We invited individuals who met one or more of the following criteria to serve on the committee of judges: Brazilian researchers who have published a guideline or explanation & elaboration document on measurement properties; authors of a book on measurement properties; authors of at least three studies on measurement properties published within the last five years; editors of journals specialized in publishing studies on measurement properties; or researchers with experience in qualitative studies, or factor analysis, or item response theory/Rasch analysis, or reliability studies. To ensure a systematic evaluation process, all judges received the same instructions that outlined the review’s objectives and evaluation criteria. Although no numeric rating scale was used, the structured qualitative assessment focused on identifying issues related to meaning, clarity, and usability.

In order to maintain the accessibility, traceability, and clarity of the original examples, we have decided to keep the original examples in English, while adding a translated version in Brazilian Portuguese. This approach ensures that Brazilian readers can fully understand the content. It also allows for direct comparison with the original wording, which preserves contextual meaning and avoids potential misinterpretation of specific terminology.

## Results

The current versions of the COSMIN Reporting Guideline 2.0 and its Explanation & Elaboration document, translated into Brazilian Portuguese, are available for free download on the COSMIN website (cosmin.nl/wp-content/uploads/RG_BRPt.pdf and cosmin.nl/wp-content/uploads/EE_BRPt_version-1.0.pdf).

First, we carefully translated all 68 items of the COSMIN Reporting Guideline 2.0 to ensure conceptual equivalence and consistency. During this process, we identified and replaced some unusual terms generated by the AI with their standard Brazilian Portuguese counterparts. The agreed-upon translations of the guideline items were then incorporated into the Explanation & Elaboration document. Next, the additional explanatory content of the Explanation & Elaboration document (e.g., explanations, rationales, and examples of good reporting) was forward-translated, reviewed, and checked for accuracy and consistency with the guideline items. A summary of the changes and the rationale behind them are presented in Table 1. Nine terms underwent substantial revisions beyond grammar. Only one unusual term translated by the AI (i.e., “construct”) was identified and corrected. To avoid confusion, we decided not to translate the names of the COSMIN tools and PROMs.

**Table 1:** Justification of the terms synthesized throughout the COSMIN reporting guideline 2.0 and its explanation & elaboration document

Original term (English) / local in the documents	Term translated into Brazilian Portuguese	Synthesis	Justification
Measurement properties Throughout the documents	Propriedades de medição	Propriedades de medida	To maintain standardization of a term throughout the text, avoid confusion with the verb “measure”
Contruct Throughout the documents	Construção	Construto	The correct translation of the word
Factor analysis Structural validity, internal consistency, and cross-cultural validity items	Análise de fatores	Análise fatorial	The most usual translation of the term
Multigroup confirmatory factor analysis Cross-cultural validity items	Análise de fator confirmatório de vários grupos	Análise fatorial confirmatória multigrupo	The most usual translation of the term
Sum scores Structural validity and cross-cultural validity items	Pontuações de soma	Soma das pontuações	The most usual translation of the term
Measurement error Measurement error items	Erro de medição	Erro de medida	The most usual translation of the term
Design Throughout the documents	Desenho	Delineamento	The most usual translation of the term
Factorial loadings Structural validity and cross-cultural validity items	Carregamentos fatoriais	Cargas fatoriais	The most usual translation of the term

Some discrepancies were found when the back-translations made by human translators and AI were compared to the original COSMIN Reporting Guideline 2.0. A supplementary table (Table S1) provides a structured overview of all identified discrepancies. Overall, the discrepancies were systematically analyzed and categorized as semantic, idiomatic, experiential, or conceptual. Most discrepancies were semantic, involving differences in wording that did not alter meaning. A smaller number of discrepancies were conceptual and required careful revision to preserve the original intent. Resolution decisions were made by consensus among the authors during the synthesis phase and refined based on expert committee feedback when necessary. Figure S1 presents a flow diagram that summarizes the translation process and the steps for identifying and resolving discrepancies.

Eleven Brazilian researchers met the criteria and were invited to join the committee of judges. Six of them agreed to participate in the study. However, after accepting the invitation, one researcher withdrew due to personal issues. The judging panel consisted of five researchers whose areas of expertise partly overlapped. These areas included qualitative studies (*n* = 2), factor analysis (*n* = 2), item response theory/Rasch analysis (*n* = 1), and reliability studies (*n* = 5). The judges were involved in reviewing the translation of the Explanation and Elaboration document, as well as evaluating the translated guideline items. Most of the revisions by the judging panel were about grammatical errors throughout the documents. They also requested that the translation of “Instrumentos de Medida de Resultados Relatados pelo Paciente (MRRP)” be changed to “Instrumentos de Desfechos Relatados pelo Paciente”, as this is the correct and usual translation of “Patient-Reported Outcome Measures”. The acronym “MRRP” was deleted because it is not commonly used and unknown in Brazilian Portuguese. The judging panel also requested that the list of acronyms be deleted from the end of the COSMIN Reporting Guideline 2.0, as it already includes acronyms in the text and spells them out. Regarding items A1 and I4, which relate to reporting objectives, the term “objetivos específicos” (specific objectives) was excluded to avoid confusion about reporting all the secondary objectives of the study instead of the main objective(s).

## Discussion

In this study, we translated the COSMIN Reporting Guideline 2.0 and its Explanation & Elaboration document into Brazilian Portuguese. The COSMIN Reporting Guideline 2.0 guides the reporting of studies on measurement properties of PROMs, and the Explanation & Elaboration document makes it easier for researchers and clinicians to apply the COSMIN Reporting Guideline 2.0. This is the first translation of the COSMIN Reporting Guideline 2.0 and its E&E document into any language other than English.

Translation of guidelines is a common procedure in the literature [10–12]. This practice is important for overcoming language barriers and facilitating the dissemination of key scientific findings. In countries where English is not widely spoken, making guidelines and explanation & elaboration documents available in their native language promotes the adoption of international standards and improves the quality and consistency of research and clinical practice. By ensuring accessibility, particularly for the COSMIN Reporting Guideline 2.0 and its Explanation & Elaboration document, the translation of these documents supports the appropriate reporting of studies on measurement properties of PROMs by native users. Furthermore, the availability of these translated documents may stimulate further research and development in the area of measurement properties by lowering the barrier to entry for professionals who may have otherwise struggled to engage with the original English-language versions. As a result, translating the COSMIN Reporting Guideline 2.0 and its Explanation & Elaboration document is not only a matter of accessibility, but also a strategy for strengthening the research infrastructure and health outcomes in non-English-speaking regions.

We used ChatGPT^®^ 4.0 machine translation to translate the COSMIN Reporting Guideline 2.0 [4] and its Explanation & Elaboration document and back-translate the COSMIN Reporting Guideline 2.0 [5]. AI translation uses algorithms and large amounts of data to translate text, and can be faster and more cost-effective than human translation [13]. However, it does not impact the validity or utility of the translated COSMIN Reporting Guideline 2.0 and its Explanation & Elaboration document, as their original and translated versions were compared by experts in studies on measurement properties of PROMs. We acknowledge that the streamlined translation process for the Explanation & Elaboration document could raise concerns about reduced rigor, especially since subtle interpretive shifts in explanatory content are possible. To address this concern, we have implemented targeted quality assurance strategies, including aligning with validated guideline items, conducting internal reviews, and performing focused expert evaluations. Additionally, this study was conducted specifically for Brazilian Portuguese. Although there are similarities across Portuguese-speaking countries, linguistic and cultural differences may influence the interpretation and applicability of translated documents. Therefore, while this version can be used in other Portuguese-speaking contexts, it should be done so with caution. Further translations are recommended to ensure cultural and linguistic appropriateness. As a result, translating the COSMIN Reporting Guideline 2.0 and its Explanation & Elaboration document is a strategy for strengthening the health outcomes in non-English-speaking regions.

## Conclusions

The COSMIN Reporting Guideline 2.0 and its Explanation & Elaboration document are translated into Brazilian Portuguese. Brazilian-speaking researchers and clinicians can use both documents to clearly understand how to report their studies on measurement properties of PROMs.

## Supplementary Information

Below is the link to the electronic supplementary material.

Supplementary Material 1

Supplementary Material 2

Supplementary Material 3

Supplementary Material 4

## Data Availability

No datasets were generated or analysed during the current study.

## References

[1] Prinsen, C. A. C., Mokkink, L. B., Bouter, L. M., Alonso, J., Patrick, D. L., de Vet, H. C. W., et al. (2018). COSMIN guideline for systematic reviews of patient-reported outcome measures. *Quality of Life Research*, *27*, 1147–1157. 10.1007/s11136-018-1798-3 29435801 10.1007/s11136-018-1798-3 PMC5891568

[2] Mokkink, L. B., Elsman, E. B. M., & Terwee,C. B. (2024). COSMIN guideline for systematic reviews of patient-reported outcome measures version 2.0. *Quality of Life Research*. 10.1007/s11136-024-03761-6 10.1007/s11136-024-03761-6 PMC11541334 39198348

[3] Ioannidis, J. P. A. (2014). How to Make More Published Research True. *Plos Medicine*, *11*, e1001747. 10.1371/journal.pmed.1001747 25334033 10.1371/journal.pmed.1001747 PMC4204808

[4] Gagnier, J. J., de Arruda, G. T., Terwee, C. B., Mokkink, L. B., Elsman, E. B. M., Firth, A. D., et al. (2025). COSMIN reporting guideline for studies on measurement properties of patient-reported outcome measures: version 2.0. *Quality of Life Research*, *34*, 1901–1911. 10.1007/s11136-025-03950-x 40153128 10.1007/s11136-025-03950-x

[5] de Arruda, G. T., Terwee, C. B., Elsman, E. B. M., Avila, M. A., Gagnier, J. J., Mokkink, L. B., et al. (2025). Explanation & Elaboration document of the COSMIN Reporting Guideline 2.0 for studies on measurement properties of patient-reported outcome measures. *Quality of Life Research*, *34*, 1891–1899. 10.1007/s11136-025-03949-4 40244497 10.1007/s11136-025-03949-4

[6] Council, B. (2024). accessed November 1,. Demandas de Aprendizagem de Inglês no Brasil. Instituto de Pesquisa Data Popular 2014:36. https://www.britishcouncil.org.br/sites/default/files/demandas_de_aprendizagempesquisacompleta.pdf

[7] EF Education First EF English Proficiency Index: A Ranking of 100 Countries and Regions by English Skills. EF Education First 2023. https://www.ef.com/wwen/epi/

[8] Beaton, D. E., Bombardier, C., Guillemin, F., & Ferraz, M. B. (2000). Guidelines for the Process of Cross-Cultural Adaptation of Self-Report Measures. *Spine (Phila Pa 1976)*, *25*, 3186–3191. 10.1097/00007632-200012150-00014 11124735 10.1097/00007632-200012150-00014

[9] Sallam, M., Barakat, M., & Sallam, M. (2024). A Preliminary Checklist (METRICS) to Standardize the Design and Reporting of Studies on Generative Artificial Intelligence–Based Models in Health Care Education and Practice: Development Study Involving a Literature Review. *Interact J Med Res*, *13*, e54704. 10.2196/54704 38276872 10.2196/54704 PMC10905357

[10] GalvãoTF, TigumanGMB, & Sarkis-OnofreR (2022). A declaração PRISMA 2020 em português: recomendações atualizadas para o relato de revisões sistemáticas. *Epidemiologia e Serviços de Saúde*, *31*. 10.1590/ss2237-9622202200011 10.1590/SS2237-9622202200011 PMC9887953 35894393

[11] Werbrouck, A., de Bekker-Grob, E., Al, M., Putman, K., & Willems, R. (2024). Consolidated Health Economic Evaluation Reporting Standards 2022 (CHEERS II) statement: a validated Dutch translation. *Expert Rev Pharmacoecon Outcomes Res*, *24*, 551–557. 10.1080/14737167.2024.2324048 38400836 10.1080/14737167.2024.2324048

[12] Hoffmann, T., Glasziou, P., Boutron, I., Milne, R., Perera, R., Moher, D., et al. (2016). Die TIDieR Checkliste und Anleitung – ein Instrument für eine verbesserte Interventionsbeschreibung und Replikation. *Das Gesundheitswesen*, *78*, 175–188. 10.1055/s-0041-111066 26824401 10.1055/s-0041-111066

[13] Moneus, A. M., & Sahari, Y. (2024). Artificial intelligence and human translation: A contrastive study based on legal texts. *Heliyon*, *10*, e28106. 10.1016/j.heliyon.2024.e28106 38524597 10.1016/j.heliyon.2024.e28106 PMC10958410

