# Supplementary material for: Cosmin reporting guideline for studies on measurement properties of patient-reported outcome measures and its explanation and elaboration document: translation into Brazilian Portuguese
Source: Qual Life Res. 2026 Jun 5;35(7):165. doi: 10.1007/s11136-026-04282-0 (PMC13241396; doi:10.1007/s11136-026-04282-0)
Supplement: Supplementary file 1 — Supplementary Material 1 [file 11136_2026_4282_MOESM1_ESM.pdf]

COSMIN *Reporting Guideline* 2.0

**Recomendações gerais de relatos relevantes para todos os estudos sobre propriedades de medida**		
Item	Nome do item	Descrição do item
Seção do relato: Título		
T1	Título	Identifique o relato como um estudo de uma ou mais propriedades de medida de um instrumento de desfechos relatados pelo paciente/*Patient Reported Outcome Measures* (PROM) específica para medir um construto específico em uma população específica.
Seção do relato: Resumo		
A1	Objetivos	Forneça o(s) objetivo(s) da pesquisa, especificando (1) o nome (e a versão, se relevante) e o(s) construto(s) da PROM, (2) as propriedades de medida que estão sendo avaliadas e (3) as características relevantes do estudo.
A2	Delineamento	Especifique os detalhes do delineamento do estudo usado para avaliar as propriedades de medida.
A3	Métodos	Especifique os métodos de avaliação de cada propriedade de medida.
A4	Resultados	Forneça os principais resultados para todas as propriedades de medida avaliadas.
A5	Discussão/Conclusões	Forneça uma breve declaração das implicações dos resultados no contexto das evidências existentes sobre a PROM.
Seção do relato: Introdução		
I1	PROM	Especifique o nome e, se relevante a versão e o(s) construto(s) da PROM.
I2	População-alvo e contexto de uso	Especifique a população-alvo e o contexto de uso para o qual a PROM foi desenvolvida.
I3	Estado do conhecimento e justificativa	Forneça uma descrição do conhecimento científico atual (o que se sabe e o que não se sabe) em relação às propriedades de medida da PROM. Explique por que o novo estudo é necessário. Forneça citações do(s) documento(s) original(is) de desenvolvimento.
I4	Objetivos	Forneça o(s) objetivo(s) da pesquisa, especificando (1) o nome (e versão, se relevante) da PROM, (2) as propriedades de medida que estão sendo avaliadas e (3) as características relevantes da amostra do estudo.
Seção do relato: Métodos gerais		
GM1	Delineamento	Especifique os detalhes do delineamento do estudo usado para avaliar as propriedades de medida.
GM2	Participantes	Especifique como os participantes do estudo foram selecionados. Especifique os critérios de inclusão e exclusão.
GM3	Detalhes da PROM	Forneça detalhes sobre a versão original da PROM, bem como da versão da PROM que está sendo estudada, especifique a estrutura conceitual (modelo reflexivo/formativo), detalhes sobre a estrutura (o número de itens e subescalas), o idioma, as opções de resposta, o período de recordação, a direção da pontuação e o algoritmo de pontuação da PROM. Especifique como a PROM foi administrada (por exemplo, em que ambiente, modo de administração (por exemplo, papel, eletrônico), quais instruções foram dadas), inclusive o país em que foi administrada.
GM4	Coleta de dados adicionais	Descreva por que e como outros dados foram coletados (por exemplo, construto e propriedades de medida dos instrumentos de comparação, características dos grupos que estão sendo comparados e justificativa para a escolha dos grupos), incluindo o modo de administração (por exemplo, papel, eletrônico).
GM5	Procedimentos de pontos de tempo	Forneça todos os pontos de tempo de todas as medições.
GM6	Justificativa para o tamanho da amostra	Forneça uma justificativa para o tamanho da amostra para todas as análises das propriedades de medida (incluindo subgrupos).
GM7	Análises estatísticas	Descreva as análises estatísticas correspondentes a todos os objetivos (consulte as recomendações específicas das propriedades de medida). Descreva os critérios para boas propriedades de medida. Cite o pacote estatístico usado e a versão.
GM8	Dados ausentes	Descreva as abordagens para lidar com dados ausentes.
GM9	Análise não planejada	Especifique as análises que não foram planejadas inicialmente e que foram realizadas, incluindo a justificativa para sua realização.
Seção do relato: Resultados gerais		
GR1	Características dos participantes	Forneça as características dos participantes do estudo, especificadas por subgrupo, se aplicável.

GR2	Tamanho da amostra	Forneça o número total de participantes incluídos no estudo e o tamanho da amostra para cada análise.
GR3	Dados ausentes	Forneça a quantidade (proporção ou contagem) e os motivos da falta de dados para cada análise da PROM e para quaisquer análises de outros instrumentos de desfechos.
GR4	Resultados	Descreva os resultados correspondentes a todos os objetivos (consulte as recomendações específicas das propriedades de medida).
Seção do relato: Discussão/conclusões		
DC1	Evidência de propriedade de medida	Forneça os principais achados e se cada propriedade de medida é suficiente ou insuficiente e sua justificativa.
DC2	Relevância prática	Discuta a relevância prática dos achados em termos de recomendações para usar ou não a PROM.
DC3	Pontos fortes e limitações	Discuta os pontos fortes e as limitações de cada estudo. Por exemplo, se houve algum viés em potencial no estudo que poderia ter afetado os resultados.
DC4	Generalização	Discuta a generalização dos resultados. Por exemplo, se os resultados poderiam ser generalizados para outras populações, considerando a amostra estudada.
DC5	Mudanças no instrumento	Discuta quais modificações são necessárias na PROM existente.
DC6	Pesquisas futuras	Descreva novas perguntas ou hipóteses de pesquisa geradas a partir desses resultados e forneça/descreva a pesquisa necessária para responder a essas perguntas.
DC7	Conclusões	Forneça as conclusões gerais sobre o uso da PROM.
Seção do relato: Outras informações		
O1	Conflitos de interesse	Declare qualquer conflito de interesse que você possa ter em relação à PROM. Isso pode incluir qualquer envolvimento no desenvolvimento da PROM ou qualquer financiamento ou lucro comercial.

**Recomendações específicas de relatos para estudos sobre Validade de conteúdo**		
Item	Nome do item	Descrição do item
**Validade de conteúdo: Métodos**		
CV1	Relevância	Especifique se, e como, os pacientes e/ou profissionais foram questionados se as instruções, cada um dos itens, as opções de resposta e o período de recordação eram relevantes para o(s) construto(s), a população e o contexto de uso.
CV2	Abrangência	Especifique se, e como, os pacientes e/ou profissionais foram questionados se todos os conceitos-chave estão incluídos na PROM.
CV3	Compreensão	Especifique se, e como, os pacientes e/ou profissionais avaliaram a compreensão das instruções, dos itens, das opções de resposta e do período de recordação da PROM.
**Validade de conteúdo: Resultados**		
CV4	Relevância	Especifique se as instruções, todos os itens, as opções de resposta e o período de recordação foram considerados relevantes pelos pacientes e/ou profissionais, para o construto, para a população e para o contexto de uso.
CV5	Abrangência	Especifique se os pacientes e/ou profissionais consideraram que todos os conceitos-chave foram incluídos na PROM.
CV6	Compreensão	Especifique se os pacientes entenderam as instruções, os itens, as opções de resposta e o período de recordação da PROM conforme pretendido e/ou se os profissionais consideraram que as instruções, os itens, as opções de resposta e o período de recordação estavam adequadamente redigidos.

**Recomendações específicas de relatos para estudos sobre Validade estrutural**		
Item	Nome do item	Descrição do item
**Validade estrutural: Métodos**		
SV1	Justificativa da abordagem	Forneça uma justificativa para a abordagem utilizada (por exemplo, análise fatorial, Teoria de Resposta ao Item (TRI)/Análise de Rasch).
SV2	Análises estatísticas	<u>Análises fatoriais exploratórias (AFE) ou confirmatórias (AFC)</u><br>Descreva o modelo testado (por exemplo, número de fatores, quais itens estão incluídos em cada fator), o método de estimativa, o tipo de matriz de correlação e os métodos e critérios para um bom ajuste do modelo.<br><br><u>TRI/Análise de Rasch</u><br>Descreva o tipo de modelo TRI/Rasch, o método de estimativa, os métodos para verificar as suposições (por exemplo, dimensionalidade, independência local,

		monotonicidade), os métodos e critérios para bons parâmetros de itens e ajuste do modelo. Indique o software e a versão utilizada.<br><br>Outras abordagens<br>Forneça detalhes dos métodos, caso tenham sido usadas outras abordagens.
**Validade estrutural: Resultados**		
SV3	Análises estatísticas	AFE ou AFC<br>Para AFE: forneça todas as cargas fatoriais, autovalores e % de variância explicada do modelo que reflete a estrutura original da PROM e o modelo de melhor ajuste, se aplicável.<br>Para AFC: forneça todas as cargas fatoriais e os resultados dos índices de ajuste do modelo que refletem a estrutura original da PROM e o modelo de melhor ajuste, se aplicável.<br><br>TRI/Análise de Rasch<br>Forneça os resultados do ajuste do item/modelo, todos os parâmetros do item e figuras, se apropriado (por exemplo, curvas características do item, mapeamento pessoa-item, funções de informação do item e/ou do teste).<br><br>Outras abordagens<br>Forneça os resultados relevantes de outras abordagens utilizadas.
**Recomendações específicas de relatos para estudos sobre Consistência interna**		
Item	Nome do item	Descrição do item
**Consistência interna: Métodos**		
IC1	Análises estatísticas	Forneça evidências da unidimensionalidade da PROM (subescalas) e forneça evidências da ausência de dependência local do item. Descreva os métodos estatísticos usados para calcular a consistência interna.
**Consistência interna: Resultados**		
IC2	Análises estatísticas	Forneça os resultados de consistência interna para cada escala unidimensional ou subescala separadamente.
**Recomendações específicas de relatos para estudos sobre Validade transcultural/Invariância da medida**		
Item	Nome do item	Descrição do item
**Validade transcultural/Invariância da medida: Métodos**		
CCV1	Variável de grupo	Descreva a variável que difere entre os subgrupos que estão sendo comparados.
CCV2	Justificativa da abordagem	Forneça uma justificativa para a abordagem usada (por exemplo, análise fatorial confirmatória multigrupo ou análise de regressão logística do funcionamento diferencial do item/*Differential Item Functioning* (DIF) usando Theta ou pontuações de soma, ou outra).
CCV3	Análises estatísticas	Análise fatorial confirmatória multigrupo (AFCMG)<br>Forneça o modelo testado, o método de estimativa, o tipo de matriz de correlação e os métodos e critérios para alteração no ajuste do modelo.<br><br>Análises do funcionamento diferencial do item/*differential item functioning* (DIF)<br>Descreva a abordagem estatística utilizada para calcular a pontuação do paciente (por exemplo, pontuação *Theta* baseada em TRI/Rasch ou pontuação de soma (não) ponderada), uma descrição dos modelos de regressão testados e os critérios utilizados para sinalizar itens para DIF.<br><br>Outras abordagens<br>Forneça detalhes dos métodos caso outras abordagens tenham sido usadas.
**Validade transcultural/Invariância da medida: Resultados**		
CCV4	Análises estatísticas	Forneça resultados sobre mudanças ou diferenças no ajuste do modelo entre os modelos testados (por exemplo, AFCMG, TRI/Análise de Rasch, análises de regressão logística de DIF ou outras abordagens).
**Recomendações específicas de relatos para estudos sobre Confiabilidade**		
Item	Nome do item	Descrição do item
**Confiabilidade: Métodos**		

R1	Estabilidade dos pacientes	Forneça argumentos para assumir a estabilidade dos pacientes no construto de interesse entre as medições repetidas.
R2	Análises estatísticas	Forneça o coeficiente de correlação intraclasse/*Intraclass Correlation Coefficient* (ICC) específico ou fórmula kappa usada e justifique a escolha da fórmula usada.
R3	Fontes de variação	Especifique qual fonte de variação foi propositalmente variada entre as medições repetidas.
R4	Intervalo de tempo	Forneça argumentos para a adequação do intervalo de tempo.
**Confiabilidade: Resultados**		
R5	Estabilidade dos pacientes	Descreva se os pacientes permaneceram estáveis no construto de interesse entre as medições repetidas.
R6	Análises estatísticas	Forneça resultados para as estatísticas calculadas (ICC ou kappa) e medidas de precisão (erros padrão ou intervalos de confiança). Forneça resultados de todos os componentes de variância ou tabelas de contingência.
**Recomendações específicas de relatos para estudos sobre Erro de medida**		
Item	Nome do item	Descrição do item
**Erro de medida: Métodos**		
ME1	Estabilidade dos pacientes	Forneça argumentos para assumir a estabilidade dos pacientes no construto de interesse entre as medições repetidas.
ME2	Análises estatísticas	Forneça e justifique a fórmula estatística específica usada para o erro padrão de medida/*Standard Error of Measurement* (SEM), menor mudança detectável/ *Smallest Detectable Change* (SDC), limites de concordância/*Limits of Agreement* (LoA) ou porcentagem de concordância (específica).
ME3	Fontes de variação	Especifique qual fonte de variação foi propositalmente variada entre as medições repetidas.
ME4	Intervalo de tempo	Forneça argumentos para a adequação do intervalo de tempo.
**Erro de medida: Resultados**		
ME5	Estabilidade dos pacientes	Descreva se os pacientes permaneceram estáveis no construto de interesse entre as medições repetidas.
ME6	Análises estatísticas	Forneça resultados para estatísticas calculadas e medidas de precisão (se aplicável). Forneça resultados de todos os componentes de variância incluídos no SEM, forneça a mudança sistemática ou diferença entre as medições repetidas (quando LoA foi aplicado) ou tabelas de contingência.
**Recomendações específicas de relatos para estudos sobre Validade de critério**		
Item	Nome do item	Descrição do item
CriV1	Critério	Justifique a suposição de que o instrumento de comparação é um padrão-ouro aceitável.
**Validade de critério: Métodos**		
CriV2	Análises estatísticas	Forneça e justifique as estatísticas usadas: correlações quando o critério tem pontuações contínuas ou área sob a curva *Receiver Operating Characteristic* (ROC)/ *area under the ROC curve*, e sensibilidade e especificidade quando o critério é dicotômico.
**Validade de critério: Resultados**		
CriV3	Análises estatísticas	Forneça resultados para as estatísticas calculadas.
**Recomendações específicas de relatos para estudos sobre Teste de hipóteses para validade de construto**		
Item	Nome do item	Descrição do item
**Teste de hipóteses para validade de construto: Métodos**		
ConV1	Hipóteses	Formule hipóteses e forneça justificativas para cada hipótese.
ConV2	Análises estatísticas	Especifique todos os métodos estatísticos usados para testar as hipóteses.
**Teste de hipóteses para validade de construto: Resultados**		
ConV3	Análises estatísticas	Forneça todos os resultados e especifique se cada resultado está de acordo com sua hipótese.
**Recomendações específicas de relatos para estudos sobre Responsividade**		
Item	Nome do item	Descrição do item
**Responsividade: Métodos**		
Resp1	Hipóteses	Formule hipóteses e forneça justificativas para cada hipótese.

Resp2	Intervenção/Exposição	Forneça a intervenção dada ou a exposição no período intermediário (ou informe que nenhuma intervenção foi fornecida).
Resp3	Análises estatísticas	Especifique todos os métodos estatísticos usados para testar as hipóteses.
**Responsividade: Resultados**		
Resp4	Análises estatísticas	Forneça resultados para as estatísticas calculadas e especifique, para a abordagem de construto, se cada resultado está de acordo com sua hipótese.
