# Supplementary material for: Cosmin reporting guideline for studies on measurement properties of patient-reported outcome measures and its explanation and elaboration document: translation into Brazilian Portuguese
Source: Qual Life Res. 2026 Jun 5;35(7):165. doi: 10.1007/s11136-026-04282-0 (PMC13241396; doi:10.1007/s11136-026-04282-0)
Supplement: Supplementary file 2 — Supplementary Material 2 [file 11136_2026_4282_MOESM2_ESM.pdf]

COSMIN

Documento de Explicação & Elaboração

Reporting Guideline

para estudos sobre propriedades de medida de instrumentos de desfechos relatados pelo paciente

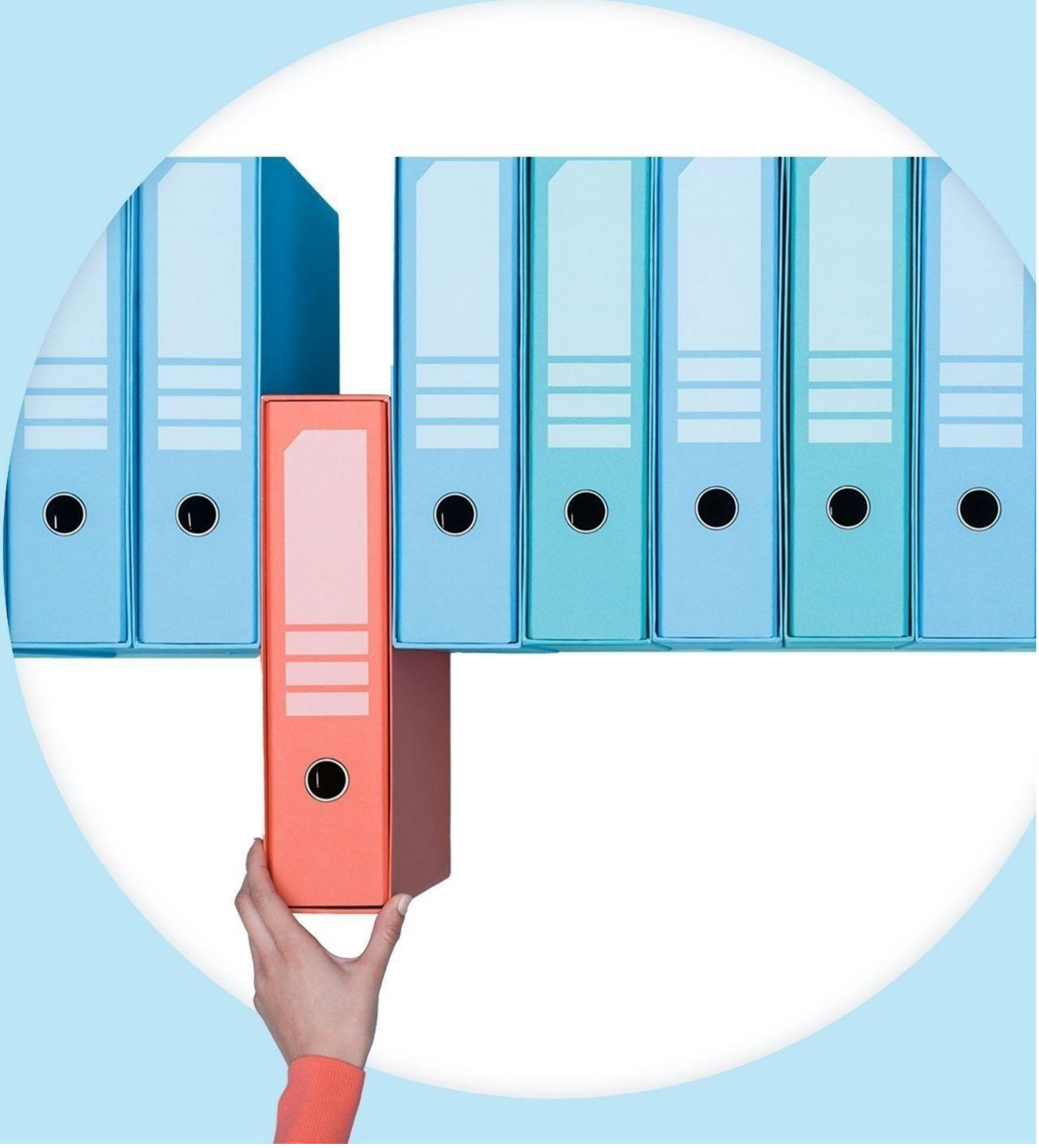

Versão:	Versão 1.0 Setembro de 2024

Escrito por:

Guilherme T de Arruda
Ellen BM Elsman
Mariana A Avila
Almir V Dibai-Filho
Andrew D Firth
Ava Mehdipour
Christos Mousoulis
Conrad J Harrison
John D Peipert
Karen Matvienko-Sikar
Kirstie L Haywood
Leonie H Klaufus
Letícia B Calixtre
Luiz A Brusaca
Mark A Dubbelman
Nan Luo
Patricia Driusso
Sandrine Herbelet
Caroline B Terwee
Joel J Gagnier
Lidwine B Mokkink

O desenvolvimento do Documento de Explicação e Elaboração (E&E) para o COSMIN *Reporting Guideline* 2.0 para estudos sobre propriedades de medida de instrumentos de desfechos relatados pelo paciente foi apoiado financeiramente pela Coordenação de Aperfeiçoamento de Pessoal de Nível Superior - Brasil (CAPES) - Código de Financiamento 001 e pela Fundação de Amparo à Pesquisa do Estado de São Paulo (2021/11871-9 e 2022/16124-0).

Autor correspondente

LB Mokkink, PhD
w.mokkink@amsterdamumc.nl
Amsterdam UMC
Department of Epidemiology and Data Science
Amsterdam Public Health research institute
Amsterdam
The Netherlands
www.cosmin.nl

© G. Tavares de Arruda, L.B. Mokkink, J. Gagnier e C.B. Terwee. 2025.

Esta publicação está protegida por direitos autorais. Sujeito a exceções legais e às disposições dos acordos de licenciamento coletivo relevantes, nenhuma reprodução de qualquer parte pode ocorrer sem a autorização por escrito da Amsterdam UMC.

Tabela de conteúdo

Conteúdo

Contexto 7
COSMIN *Reporting Guideline* para estudos sobre propriedades de medida de PROMs 11
Explicação & elaboração 12
Terminologia e abreviações 13
Recomendações gerais de relatos relevantes para todos os estudos sobre propriedades de medida 15
Seção do relato: Título 15
T1 - Título 15
Seção do relato: Resumo 17
A1 - Objetivos 17
A2 - Delineamento 20
A3 - Métodos 22
A4 - Resultados 25
A5 - Discussão/Conclusões 28
Seção do relato: Introdução 31
I1 - PROM 31
I2 - População-alvo e contexto de uso 33
I3 - Estado do conhecimento e justificativa 35
I4 - Objetivos 39
Seção do relato: Métodos gerais 41
GM1 - Delineamento 41
GM2 - Participantes 43
GM3 - Detalhes da PROM 45
GM4 - Coleta de dados adicionais 47
GM5 - Procedimentos de pontos de tempo 50
GM6 - Justificativa para o tamanho da amostra 52
GM7 - Análises estatísticas 55
GM8 - Dados ausentes 57
GM9 - Análise não planejada 58
Seção do relato: Resultados gerais 59
GR1 - Características dos participantes 59
GR2 - Tamanho da amostra 62
GR3 - Dados ausentes 64
GR4 - Resultados 66

Seção do relato: Discussão/conclusões 70
DC1 - Evidência de propriedade de medida 70
DC2 - Relevância prática 72
DC3 - Pontos fortes e limitações 75
DC4 - Generalização 78
DC5 - Mudanças no instrumento 80
DC6 - Pesquisas futuras 82
DC7 - Conclusões 84
Seção do relato: Outras informações 86
O1 - Conflitos de interesse 86
Recomendações específicas de relatos para estudos para cada propriedade de medida 88
Validade de conteúdo 88
Validade de conteúdo: Métodos 88
CV1 - Relevância 88
CV2 - Abrangência 92
CV3 - Compreensão 95
Validade de conteúdo: Resultados 97
CV4 - Relevância 97
CV5 - Abrangência 99
CV6 - Compreensão 102
Validade estrutural 104
Validade estrutural: Métodos 104
SV1 - Justificativa da abordagem 104
SV2 - Análises estatísticas 106
Validade estrutural: Resultados 110
SV3 - Análises estatísticas 110
Consistência interna 118
Consistência interna: Métodos 118
IC1 - Análises estatísticas 118
Consistência interna: Resultados 120
IC2 - Análises estatísticas 120
Validade transcultural/Invariância da medida 121
Validade transcultural/Invariância da medida: Métodos 122
CCV1 - Variável de grupo 122
CCV2 - Justificativa da abordagem 123
CCV3 - Análises estatísticas 125
Validade transcultural/Invariância da medida: Resultados 128

CCV4 - Análises estatísticas 128
Confiabilidade..... 133
Confiabilidade: Métodos 133
R1 - Estabilidade dos pacientes..... 133
R2 - Análises estatísticas 135
R3 - Fontes de variação 137
R4 - Intervalo de tempo 139
Confiabilidade: Resultados 140
R5 - Estabilidade dos pacientes..... 140
R6 - Análises estatísticas 141
Erro de medida..... 142
Erro de medida: Método 142
ME1 - Estabilidade dos pacientes 142
ME2 - Análises estatísticas..... 144
ME3 - Fontes de variação 146
ME4 - Intervalo de tempo..... 147
Erro de medida: Resultados 148
ME5 - Estabilidade dos pacientes 148
ME6 - Análises estatísticas..... 149
Validade de critério 151
CriV1 - Critério 151
Validade de critério: Métodos..... 153
CriV2 - Análises estatísticas 153
Validade de critério: Resultados 155
CriV3 - Análises estatísticas 155
Teste de hipóteses para validade de construto 157
Teste de hipóteses para validade de construto: Métodos..... 157
ConV1 - Hipóteses 157
ConV2 - Análises estatísticas..... 161
Teste de hipóteses para validade de construto: Resultados 164
ConV3 - Análises estatísticas..... 164
Responsividade..... 167
Responsividade: Métodos 167
Resp1 – Hipóteses 167
Resp2 - Intervenção/Exposição..... 170
Resp3 - Análises estatísticas 171
Responsividade: Resultados..... 173

Resp4 - Análises estatísticas 173
Referências 176

Contexto

O uso de instrumentos de desfechos relatados pelo paciente/*Patient Reported Outcome Measures* (PROM) na prática clínica e na pesquisa para avaliar o estado de saúde relatado pelo paciente aumentou significativamente. As PROMs devem ser suficientemente confiáveis, válidas e responsivas para que se possa confiar nas pontuações obtidas. Para conhecer a qualidade de uma PROM, é necessário realizar estudos sobre as propriedades de medida. As propriedades de medida são os aspectos de qualidade de um instrumento de desfecho, referindo-se à sua validade, confiabilidade e responsividade [1]. Os estudos sobre propriedades de medida devem ser de alta qualidade metodológica para garantir conclusões adequadas e confiáveis sobre as propriedades de medida de um instrumento de desfecho (por exemplo, PROMs). Nove propriedades de medida foram definidas pela iniciativa COSMIN (*COnsensus-based Standards for the selection of health Measurement INstruments*), conforme mostrado na Figura 1 e na Tabela 1 [2].

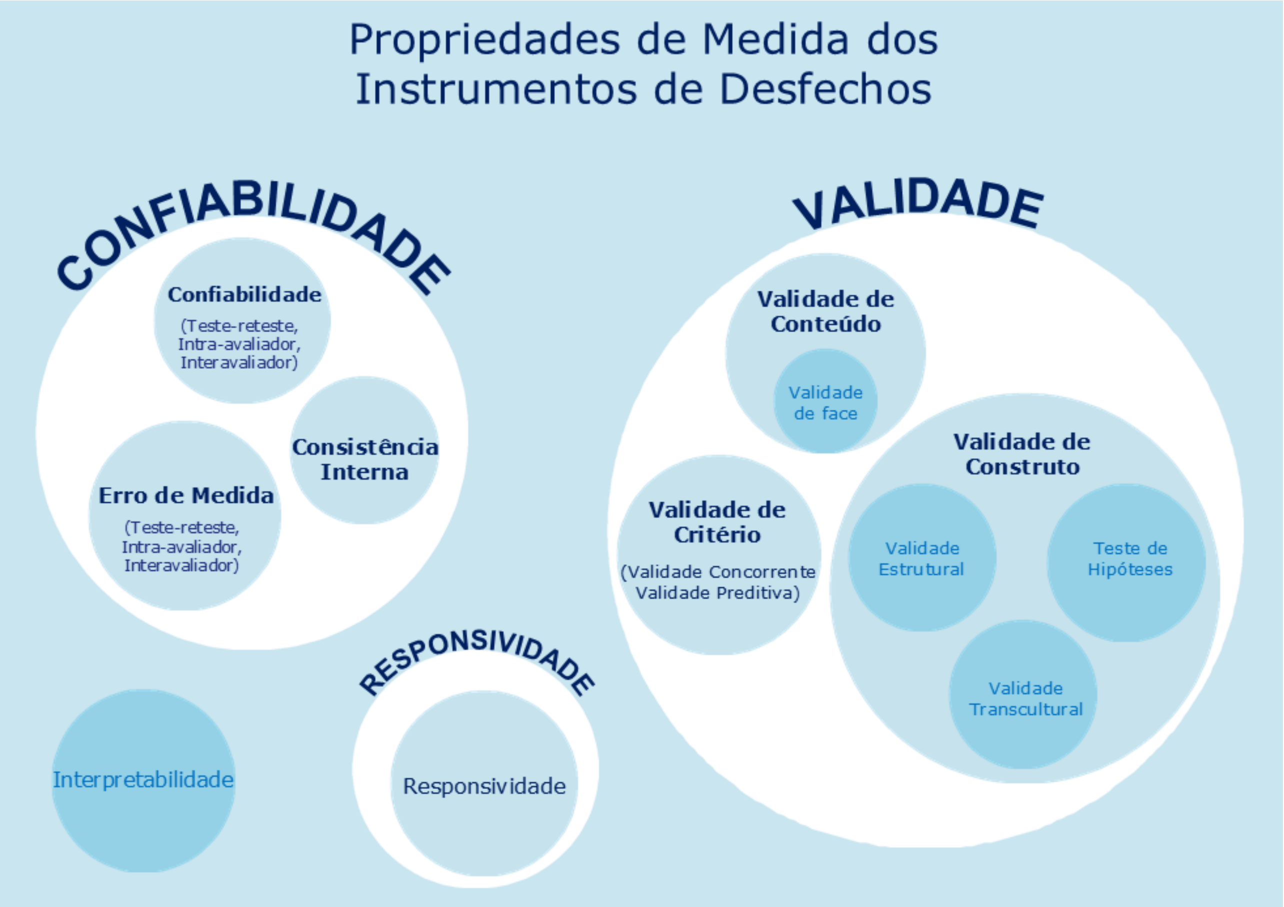

Figura 1 A taxonomia do COSMIN [3]

Tabela 1 Definições COSMIN de domínios, propriedades de medida e aspectos das propriedades de medida [1]

Domínios	Definições
Confiabilidade	O grau em que a medida está livre de erro de medida
Confiabilidade (definição estendida)	A extensão em que as pontuações de pacientes, que não apresentaram alterações, permanecem as mesmas em medidas repetidas sob diferentes condições: por exemplo, diferentes conjuntos de itens da mesma PROM (consistência interna); ao longo do tempo (teste-reteste); por diferentes pessoas na mesma ocasião (interavaliador) ou pelas mesmas pessoas (ou seja, avaliadores ou respondedores) em diferentes ocasiões (intra-avaliador)
Validade	O grau em que uma PROM mede o(s) construto(s) que se propõe a medir
Validade de construto[1]	O grau em que as pontuações de uma PROM são consistentes com as hipóteses (por exemplo, no que diz respeito às relações internas, relações com pontuações de outros instrumentos ou diferenças entre grupos relevantes) com base na suposição de que a PROM mede de forma válida o construto a ser medido
Responsividade	A capacidade de uma PROM em detectar mudanças ao longo do tempo no construto a ser medido
**Propriedades de medida**	**Definições**
Validade de conteúdo	O grau em que o conteúdo de uma PROM é um reflexo adequado do construto a ser medido
Validade de face[2]	O grau em que (os itens de) uma PROM realmente parece(m) ser um reflexo adequado do construto a ser medido
Validade estrutural	O grau em que as pontuações de uma PROM são um reflexo adequado da dimensão do construto a ser medido
Consistência interna	O grau de interrelação entre os itens
Validade transcultural	O grau em que o desempenho dos itens em uma PROM traduzida ou adaptada culturalmente é um reflexo adequado do desempenho dos itens da versão original da PROM
Confiabilidade	A proporção da variância total nas medidas que pode ser atribuída a diferenças “verdadeiras”[3] entre os pacientes
Erro de medida	O erro sistemático e aleatório na pontuação de um paciente que não é atribuído a mudanças reais no construto a ser medido

**Validade de critério**	O grau em que as pontuações de uma PROM são um reflexo adequado de um 'padrão-ouro'
**Teste de hipóteses para validade de construto**	O grau em que as pontuações de uma PROM são consistentes com as hipóteses (por exemplo, no que diz respeito a relações internas, relações com pontuações de outros instrumentos ou diferenças entre grupos relevantes) com base na suposição de que a PROM mede de forma válida o construto a ser medido
**Responsividade**	A capacidade de uma PROM em detectar mudanças ao longo do tempo no construto a ser medido
**Outras propriedades relevantes**	**Definições**
**Viabilidade**[4]	Facilidade de aplicação da PROM em seu contexto de uso pretendido, dadas as restrições, como tempo ou dinheiro [4]
**Interpretabilidade**[4]	O grau em que se pode atribuir significado qualitativo, isto é, conotações clínicas ou comumente compreendidas às pontuações quantitativas de uma PROM ou à mudança nas pontuações

Abreviações: PROM: instrumento de desfechos relatados pelo paciente/*Patient Reported Outcome Measures*, TCT: teoria clássica dos testes.

[1] a validade do construto tem várias formas (ou seja, propriedades de medida), ou seja, validade estrutural, teste de hipóteses para validade do construto e validade transcultural\invariância da medida; [2] a validade de face é uma forma não estruturada de validade de conteúdo; [3] A palavra "verdadeiras" deve ser vista no contexto da TCT, que afirma que qualquer observação é composta por dois componentes: valor verdadeiro e erro associado à observação. "Verdadeira" é a pontuação média que seria obtida se a escala fosse dada um número infinito de vezes. Refere-se apenas à consistência da pontuação, e não à sua precisão [5] [4] A viabilidade e a interpretabilidade não são consideradas uma propriedade de medida, mas um aspecto importante na seleção de instrumentos de desfechos.

Diretrizes do COSMIN

O COSMIN desenvolveu várias diretrizes. Na Figura 2, mostramos quando usar cada diretriz do COSMIN.

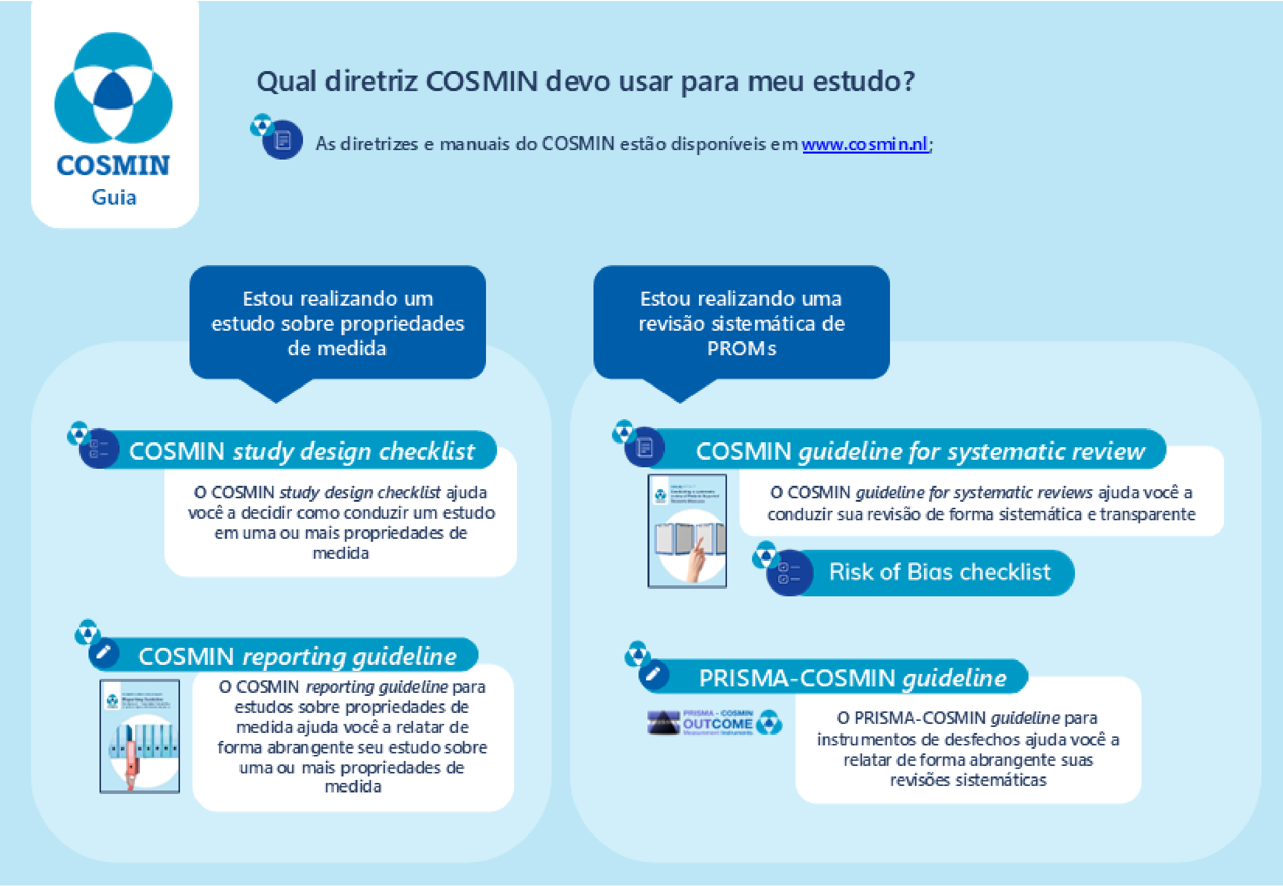

Figura 2 Visão geral das diretrizes do COSMIN

O COSMIN *Reporting Guideline* se concentra nos relatos de estudos sobre a avaliação das propriedades de medida de PROMs. Ele não se concentra no desenvolvimento de PROMs. Além disso, não se destina a orientar a condução de estudos de propriedades de medida; para tanto, você pode usar o "COSMIN *study design checklist*". Também não se destina a avaliar a qualidade metodológica dos estudos sobre propriedades de medida; para isso, você pode usar a Lista de Verificação COSMIN *Risk of Bias*, que faz parte do COSMIN *guideline for systematic review* [6].

COSMIN *Reporting Guideline* para estudos sobre propriedades de medida de PROMs

Os estudos sobre as propriedades de medida de PROMs devem ser relatados na íntegra para que os leitores tenham todas as informações necessárias para entender o estudo e verificar a interpretação e as conclusões [7]. A primeira versão do COSMIN *Reporting Guideline* para estudos sobre propriedades de medida de PROMs foi desenvolvida em 2021 [7] e atualizada em 2025 [8]. O objetivo desta diretriz é orientar os pesquisadores sobre o que deve ser relatado nos estudos sobre as propriedades de medida de PROMs.

A versão 2.0 da diretriz inclui um conjunto de 68 itens e está separada em duas seções. A seção "Recomendações gerais de relatos relevantes para todos os estudos sobre propriedades de medida" contém 31 itens agrupados em sete aspectos gerais de um estudo de relato sobre propriedades de medida:

- Título (1 item);
- Resumo (5 itens);
- Introdução (4 itens);
- Métodos gerais (9 itens);
- Resultados gerais (4 itens);
- Discussão/conclusões (7 itens);
- Outras informações - conflitos de interesse (1 item).

A seção "Recomendações específicas de relato para cada propriedade de medida" contém 37 itens agrupados em nove propriedades de medida e identifica questões específicas sobre métodos e análises que devem ser relatados para cada propriedade de medida:

- Validade de conteúdo (6 itens);
- Validade estrutural (3 itens);
- Consistência interna (2 itens);
- Validade transcultural/invariância da medida (4 itens);
- Confiabilidade (6 itens);
- Erro de medida (6 itens);
- Validade de critério (3 itens);
- Teste de hipóteses para validade de construto (3 itens);
- Responsividade (4 itens).

O COSMIN *Reporting Guideline* foi desenvolvido como uma ferramenta modular, ou seja, ao usar esse guia, você deve sempre usar os itens da seção “Recomendações gerais de relatos relevantes para todos os estudos sobre propriedades de medida” e escolher as seções relevantes das “Recomendações específicas de relatos para cada propriedade de medida” referentes à propriedade de medida que você estudou e deseja relatar. Por exemplo, se você avaliou a validade estrutural e a consistência interna de uma PROM, você pode usar os itens gerais e os itens específicos das subseções sobre validade estrutural e consistência interna.

Como citar este documento

Consulte este documento de Explicação & Elaboração do COSMIN *Reporting Guideline* 2.0 para relatar estudos sobre propriedades de medida de instrumentos de desfechos relatados pelo paciente:

Arruda GT, et al. Explanation & Elaboration document of the COSMIN Reporting Guideline 2.0 for studies on measurement properties of patient-reported outcome measures. Quality of Life Research, 2025;34(7):1891-1899. Doi: 10.1007/s11136-025-03949-4

Explicação & elaboração

Um documento de Explicação & Elaboração (E&E) ainda não foi desenvolvido para a versão 1.0 do COSMIN *Reporting Guideline* para facilitar seu uso. Um documento de E&E fornece explicações detalhadas e abrangentes sobre um determinado tópico e oferece exemplos práticos, garantindo que os pesquisadores entendam as nuances do uso eficaz do guia. No documento de E&E, cada item é explicado com os motivos *pelos quais* o relato do item é recomendado e exemplos de bons relatos de estudos publicados em acesso aberto. Cada item deste documento é dividido em “Identificação do item”, “Explicação”, “Elementos essenciais” e “Exemplos de itens”. Para os exemplos de bons relatos, removemos todas as citações bibliográficas dentro dos exemplos para evitar possíveis confusões com a nossa citação bibliográfica, removemos os nomes dos autores e explicamos as abreviações para facilitar a compreensão.

Terminologia e abreviações

O Quadro 1 apresenta um glossário dos termos usados neste documento.

Quadro 1 Glossário de termos usados no documento de E&E para o COSMIN *Reporting Guideline* para estudos sobre propriedades de medida de PROMs

**Propriedades de medida**<br>Os aspectos de qualidade de um instrumento de desfecho, referentes à sua validade, confiabilidade e responsividade da pontuação do instrumento [1]. Cada propriedade de medida requer seu próprio delineamento do estudo e métodos estatísticos de avaliação. Diferentes definições de propriedades de medida estão sendo usadas. O COSMIN desenvolveu uma taxonomia com definições baseadas em consenso para propriedades de medida [1]. Outro termo para propriedades de medida é propriedades psicométricas.
**Domínio de desfecho**<br>Refere-se *ao que* está sendo medido (por exemplo, fadiga, função física, glicemia, intensidade da dor). Outros termos para o domínio de desfecho incluem construto, conceito, traço latente, fator, atributo.
**Resultado relatado pelo paciente**<br>"Qualquer relato do estado da condição de saúde de um paciente que venha diretamente do paciente, sem interpretação da resposta do paciente por um clínico ou qualquer outra pessoa." [9]
**Instrumentos de desfechos relatados pelo paciente**<br>Um instrumento de desfecho (geralmente um questionário) preenchido pelos pacientes para medir os desfechos relatados pelo paciente.<br>"Um instrumento de desfechos relatados pelo paciente pode ser usado para medir o efeito de uma intervenção em saúde em um ou mais construtos (ou seja, o que está sendo medido, como um sintoma ou grupo de sintomas, efeitos em uma função específica ou grupo de funções, ou um grupo de sintomas ou funções que demonstram medir a gravidade de uma condição de saúde)". [9]
**Resultados das propriedades de medida**<br>Resultados de um estudo sobre uma propriedade de medida. Os resultados das propriedades de medida têm formatos diferentes, dependendo da propriedade de medida. Por exemplo, os resultados da confiabilidade podem ser a estimativa do coeficiente de correlação intraclasse/*intraclass correlation coefficient* (ICC), ou os resultados da validade estrutural podem ser as cargas fatoriais dos itens para suas respectivas escalas e a porcentagem da variância explicada.

Quadro 2 Lista de siglas usadas no documento de E&E para o COSMIN *Reporting Guideline* para estudos sobre propriedades de medida de PROMs

AFC	Análise fatorial confirmatória
AFCMG	Análise fatorial confirmatória multigrupo
AFE	Análise fatorial exploratória
AUC	Área sob a curva ROC
CFI	*Comparative Fit Index*
COSMIN	*COnsensus-based Standards for the selection of health Measurement INstruments*
DIF	*Differential Item Functioning*
DP	Desvio Padrão
E&E	Explicação & Elaboração
ICC	*Intraclass Correlation Coefficient*
IM	Índices de modificação
KMO	*Kaiser-Meyer-Olkin*
LoA	*Limits of Agreement*
MIC	*Minimal Important Change*
MLR	*Maximum Likelihood with Robust standard errors*
PROM	Instrumentos de desfechos relatados pelo paciente
RMSEA	*Root Mean Square Error of Approximation*
SDC	*Smallest Detectable Change*
SEM	*Standard Error of Measurement*
SRMR	*Standardized Root Mean Squared Residual*
TAC	Teste adaptativo de computador
TLI	*Tucker-Lewis Index*
TRI	Teoria da Resposta ao Item

Recomendações gerais de relatos relevantes para todos os estudos sobre propriedades de medida

Seção do relato: Título

T1 - Título

Identifique o relato como um estudo de uma ou mais propriedades de medida de um instrumento de desfechos relatados pelo paciente/*Patient Reported Outcome Measures* (PROM) específica para medir um construto específico em uma população específica.

Explicação: Com um título claro e preciso para o seu artigo, você pode facilitar a compreensão do leitor sobre o que está descrito em seu artigo. Um título descritivo também facilita a indexação e a recuperação subsequente em bases de dados eletrônicas, como Medline e EMBASE [7]. Recomendamos que você identifique seu relato como um estudo de uma ou mais propriedades de medida de uma PROM específica para medir um construto específico em uma população específica. Os detalhes sobre a PROM específica referem-se ao nome e à versão da PROM. Ao incluir a versão da PROM no título, especialmente nos casos em que existem versões diferentes (por exemplo, SF-36, SF-12, SF-8a), traduções (por exemplo, a versão húngara do *Female Sexual Function Index* - FSFI-H) ou versões para populações específicas (por exemplo, FSFI para pacientes com câncer de mama - FSFI-BC), você permite a fácil identificação de qual PROM específica é avaliada em seu artigo. A justificativa para isso é que cada versão ou PROM modificada é considerada uma PROM exclusiva. Se o nome da PROM não identificar claramente o construto que está sendo medido, recomendamos incluir no título o construto que a PROM pretende medir (por exemplo, Escala Numérica para avaliar a gravidade da dor no joelho, Escala Visual Analógica para avaliar a gravidade da fadiga relacionada ao esforço físico ou Índice de Barthel para medir o desempenho nas atividades da vida diária). Ao examinar várias propriedades de medida, você pode declarar no título que "as propriedades de medida foram avaliadas" ou "a qualidade da [PROM] foi avaliada". Também é razoável usar termos para domínios de medida (por exemplo, validade, confiabilidade, responsividade) no título. Nesse caso, é importante deixar claro no resumo e no manuscrito quais propriedades de medida dentro de cada domínio estão sendo avaliadas. Também recomendamos que você use a terminologia das propriedades de medida de acordo com a terminologia COSMIN baseada em consenso internacional [1]. Para facilitar a

compreensão da generalização dos resultados, recomendamos que você descreva a população do estudo em termos das principais doenças e características demográficas.

Elementos essenciais

- Nome específico e versão da PROM.
- O construto que a PROM pretende medir.
- Propriedades de medida específicas avaliadas, usando a terminologia COSMIN.
- Principais doenças e características demográficas da população do estudo.

Exemplo geral

"[Propriedades de medida/propriedades de medida específicas de interesse] do [nome/versão da(s) PROM(s)] para medir [desfecho de interesse] em [população de interesse]"

Exemplo 1

"Structural validity of the Pittsburgh Sleep Quality Index among medical students in Iran" [10]

Tradução: "Validade estrutural do *Pittsburgh Sleep Quality Index* entre estudantes de medicina no Irã"

Exemplo 2

"The Brazilian-Portuguese version of the Upper Extremity Functional Index (UEFI): Translation, cross-cultural adaptation and measurement properties for Brazilian adults after a burn injury" [11]

Tradução: "Versão em português brasileiro do *Upper Extremity Functional Index* (UEFI): Tradução, adaptação transcultural e propriedades de medida para adultos brasileiros após uma lesão por queimadura"

Seção do relato: Resumo

É fundamental fornecer uma interpretação abrangente do estudo sobre as propriedades de medida das PROMs dentro do contexto mais amplo das evidências existentes. No entanto, a orientação específica e as limitações de palavras impostas pelas revistas aumentam a complexidade da tarefa de construir um resumo convincente. Portanto, recomendamos que você selecione os detalhes mais relevantes para relatar no resumo do seu manuscrito.

A1 - Objetivos

Forneça o(s) objetivo(s) da pesquisa, especificando (1) o nome (e a versão, se relevante) e o(s) construto(s) da PROM, (2) as propriedades de medida que estão sendo avaliadas e (3) as características relevantes do estudo.

Explicação: Os objetivos são declarações sobre as várias realizações que a pesquisa pretende alcançar. Recomendamos que você forneça o(s) objetivo(s) do seu estudo no resumo. Os objetivos devem incluir as **propriedades de medida (ou domínios) que estão sendo estudadas**, o **nome (e a versão, se relevante)** e **o(s) construto(s) que a PROM pretende medir**, e uma descrição geral das **características dos participantes do estudo**. Observe que alguns desses aspectos também podem ser relatados em uma frase de introdução ou na seção de métodos do resumo. A indicação das propriedades de medida que estão sendo investigadas no resumo fornece informações que permitirão aos leitores decidirem se o artigo completo é ou não relevante para eles. Se o limite de caracteres/palavras permitir, recomendamos que você indique claramente quais propriedades de medida específicas estão sendo estudadas, de acordo com a terminologia COSMIN [1]. No entanto, se muitas propriedades de medida forem estudadas, o resumo deve, pelo menos, indicar quais domínios (ou seja, confiabilidade, validade, responsividade) são estudados, uma vez que os detalhes sobre quais propriedades de medida específicas são avaliadas no artigo podem ficar claros na seção de métodos ou resultados do resumo. Fornecer informações importantes sobre a PROM que está sendo avaliada (por exemplo, nome, versão e construto que se pretende medir) no resumo também ajuda os leitores a decidirem se devem acessar o relato completo. Se você empregar um banco de itens, como um dos bancos de itens do PROMIS, recomendamos que informe explicitamente a versão (por exemplo, PROMIS *item bank* v1.0). Em situações em que o construto a ser medido não faz parte do nome da PROM, ou quando vários construtos estão sendo medidos (por exemplo, em uma PROM multidimensional), recomendamos descrever e definir explicitamente cada um dos construtos que a PROM pretende medir no resumo (por

exemplo, na frase de introdução do resumo). Uma descrição das características dos participantes do estudo em termos de características relevantes da doença e/ou características demográficas relevantes ajuda o leitor do seu estudo a entender a aplicabilidade ou generalização dos resultados a pacientes, subgrupos ou populações específicas. As propriedades de medida são específicas da população em que foram avaliadas. Em muitos casos, pode não ser razoável generalizar os resultados de um estudo para outra população [12].

Elementos essenciais

• Objetivo(s) da pesquisa, incluindo o nome - e a versão - da PROM, o construto que está sendo medido, as propriedades de medida que estão sendo estudadas e as características dos participantes do estudo.

Exemplo geral

"O objetivo deste estudo foi avaliar as [propriedades de medida/propriedades de medida específicas de interesse] do [nome/versão da(s) PROM(s)] para medir [desfecho de interesse] em [características dos participantes do estudo]"

Exemplo 1

"This study evaluated the reliability and validity of the Sinhala version of Katz index of activities of daily living (ADL) in assessing the functional status of older people aged >65 years." [13]

Tradução: "Este estudo avaliou a confiabilidade e a validade da versão cingalesa do índice Katz de atividades da vida diária (AVD) na avaliação do status funcional de idosos com idade >65 anos."

Exemplo 2

"The current study investigated the structural validity and internal consistency of the Strengths and Stressors (SSF) questionnaire (...) used in Swedish habilitation services to measure the positive and negative consequences that the fostering of a child with a developmental disability can have on family functioning." [14]

Tradução: "O presente estudo investigou a validade estrutural e a consistência interna do questionário *Strengths and Stressors* (SSF) (...) usado nos serviços de habilitação suecos para medir as consequências positivas e negativas que o acolhimento de uma criança com deficiência de desenvolvimento pode ter sobre a funcionalidade da família."

A2 - Delineamento

Especifique os detalhes do delineamento do estudo usado para avaliar as propriedades de medida.

Explicação: Recomendamos relatar os principais elementos do delineamento do estudo. Por exemplo, deve ficar claro se o estudo foi transversal ou longitudinal e se houve alguma intervenção. Por exemplo, quando a pesquisa envolver a tradução e a avaliação da validade de conteúdo de uma PROM usando um método qualitativo, recomendamos relatar o delineamento como "tradução e estudo qualitativo". Se o estudo avaliou a validade estrutural, a consistência interna, a validade transcultural, o teste de hipóteses para a validade do construto e/ou a validade do critério de uma PROM, o delineamento do estudo é normalmente chamado de "estudo transversal". Se o estudo avaliou a confiabilidade teste-reteste e o erro de medida de uma PROM, ele deve ser relatado como um "estudo observacional com medidas repetidas em pacientes estáveis" ou um "estudo longitudinal". Se o estudo avaliou a responsividade, ele poderia ser relatado como um "estudo longitudinal" ou "estudo de coorte prospectivo".

Elementos essenciais

- O delineamento do estudo em termos de número de momentos de avaliação de desfechos (por exemplo, transversal, delineamento do teste-reteste, longitudinal), tipo de dados (por exemplo, qualitativo) e/ou método de alocação de exposição/intervenção (por exemplo, estudo observacional, aleatorizado).

Exemplo 1

"This is a cross-sectional study in which we used confirmatory factor analysis to identify the best structure of the Roland-Morris Disability Questionnaire (RMDQ)." [15]

Tradução: "Este é um estudo transversal no qual usamos a análise fatorial confirmatória para identificar a melhor estrutura do *Roland-Morris Disability Questionnaire* (RMDQ)".

Exemplo 2

"A longitudinal study was performed, (…) patients with advanced chronic kidney disease (CKD) were invited by e-mail to complete the PROMs digitally at the KLIK research platform (www.hetklikt.nu) at 3 time points; at inclusion (i.e. baseline), after 2 weeks (for assessing test-retest reliability, as described in a separate paper [ref]) and after 6 months." [16]

Tradução: "Foi realizado um estudo longitudinal, (...) pacientes com doença renal crônica (DRC) avançada foram convidados por e-mail a preencher as PROMs digitalmente na plataforma de pesquisa KLIK (www.hetklikt.nu) em 3 momentos: na inclusão (ou seja, linha de base), após 2 semanas (para avaliar a confiabilidade teste-reteste, conforme descrito em um artigo separado [ref]) e após 6 meses."

A3 - Métodos

Especifique os métodos de avaliação de cada propriedade de medida.

Explicação: Para ajudar os leitores a entender como o estudo foi conduzido, é importante relatar os principais métodos de avaliação de cada propriedade de medida, incluindo as abordagens estatísticas usadas no estudo. No mínimo, recomendamos que os seguintes itens sejam relatados: Para a validade de conteúdo, se os pacientes e/ou profissionais/especialistas avaliaram a relevância, a abrangência e a compreensão da PROM. Em estudos de validade estrutural, a abordagem usada para analisar os dados (por exemplo, análise fatorial, Teoria de Resposta ao Item (TRI), etc.); Para a consistência interna, informe que a consistência interna de cada escala unidimensional foi avaliada; Para a validade transcultural/invariância da medida, forneça as características dos grupos que estão sendo comparados; Para a confiabilidade teste-reteste e o erro de medida, informe se o coeficiente de correlação intraclasse/*intraclass correlation coefficient* (ICC) e o erro padrão da medida/*standard error of measurement* (SEM) foram calculados; Para a validade de critério, informe qual critério foi usado; Para o teste de hipóteses para a validade do construto e a responsividade, quais hipóteses foram testadas (por exemplo, ao usar uma palavra como "expectativas", "hipóteses", "pressuposto" ou "nós esperamos/como esperado" - nos resultados); Em estudos de responsividade, informe se os pacientes receberam alguma intervenção ou exposição no período entre as avaliações. Ao especificar claramente os métodos no resumo, o leitor pode compreender rapidamente a abordagem e o método do estudo, e o artigo pode ser indexado de forma precisa e específica. Para evitar redundância, alguns detalhes de métodos ou abordagens estatísticas podem não ser descritos na seção de métodos, mas podem ser relatados na seção de resultados do resumo. Por exemplo, a faixa do intervalo de tempo em um delineamento de teste-reteste ou a fórmula específica do ICC usado.

Elementos essenciais

• Para a validade de conteúdo, se os profissionais e/ou pacientes avaliaram a relevância, a abrangência e a compreensão da PROM.

• Para a validade estrutural, a abordagem usada para analisar os dados.

• Para a consistência interna, o cálculo de cada escala unidimensional.

• Para a validade transcultural/invariância da medida, as características dos grupos que estão sendo comparados.

• Para a confiabilidade teste-reteste e erro de medida, se o ICC e o SEM foram calculados.

• Para a validade do critério, qual critério foi usado.

• Em relação ao teste de hipóteses para validade de construto e responsividade, informe que hipóteses específicas foram testadas.

• Para a responsividade, se os pacientes receberam alguma intervenção ou exposição no período entre as avaliações.

Exemplo 1

"Qualitative research included 35 Small Intestinal Bacterial Overgrowth (SIBO) patients in three study stages, using a hybrid concept elicitation (CE)/cognitive interview (CI) method with US patients, ≥ 18 years. Stage 1 included a literature review, clinician interviews, and initial CE interviews with SIBO patients to identify symptoms important to patients for inclusion in the Symptom Measure (SSM). Stage 2 included hybrid CE/CI to learn more about patients' SIBO experience and test the draft SSM. Finally, stage 3 used CIs to refine the instrument and test its content validity." [17]

Tradução: "A pesquisa qualitativa incluiu 35 pacientes com supercrescimento bacteriano do intestino delgado (SBID) em três estágios do estudo, usando um método híbrido de elicitação de conceito (EIC)/entrevista cognitiva (EnC) com pacientes dos EUA, ≥ 18 anos. O estágio 1 incluiu uma revisão da literatura, entrevistas com médicos e entrevistas iniciais de EIC com pacientes com SBID para identificar sintomas importantes para os pacientes para inclusão na Medida de Sintomas (MS). O estágio 2 incluiu EIC/EnC híbridos para saber mais sobre a experiência dos pacientes com SBID e testar a versão preliminar da MS. Finalmente, o estágio 3 usou EnCs para refinar o instrumento e testar sua validade de conteúdo."

Exemplo 2

"(...) structural validity was evaluated by exploratory and confirmatory factor analysis; internal consistency by Cronbach's Alpha [for each unidimensional scale]; measurement invariance by multigroup confirmatory factor analysis between geographic regions of Brazil; and construct validity [by testing a hypothesis about the correlation between] WHODAS 2.0 to the Numerical Rating Scale for pain severity." [18]

Tradução: "(...) a validade estrutural foi avaliada pela análise fatorial exploratória e confirmatória; a consistência interna pelo Alfa de Cronbach [para cada escala unidimensional]; a invariância da medida pela análise fatorial confirmatória multigrupo entre as regiões geográficas do Brasil; e a validade de construto [testando uma hipótese sobre a correlação entre] o WHODAS 2.0 e a *Numerical Rating Scale* para gravidade da dor."

Exemplo 3

"27 patient-reported outcomes version of the common terminology criteria for adverse events (PRO-CTCAE®) items representing 14 symptomatic adverse events (AEs) were collected using both a 24-hour recall (24 h) and the standard 7-day recall in a sample of patients receiving active cancer treatment (n = 113). Using data captured with a PRO-CTCAE-24h on days 6 and 7, and 20 and 21, we computed intra-class correlation coefficients ($ICC[_{agreement}]$) (...)" [19]

Tradução: "27 itens do *patient-reported outcomes version of the common terminology criteria for adverse events* (PRO-CTCAE®), representando 14 eventos adversos sintomáticos (EAs), foram coletados usando um período de recordação de 24 horas (24 h) e o período de recordação padrão de 7 dias em uma amostra de pacientes em tratamento ativo de câncer (n = 113). Usando dados capturados com um PRO-CTCAE-24h nos dias 6 e 7, e 20 e 21, calculamos o coeficiente de correlação intraclasse/*intraclass correlation coefficients* ($ICC[_{concordância}]$) (...)"

A4 - Resultados

Forneça os principais resultados para todas as propriedades de medida avaliadas.

Explicação: É importante fornecer os principais resultados de todas as propriedades de medida que estão sendo estudadas. Se o limite de palavras não permitir, é razoável informar se as propriedades de medida são suficientes, insuficientes ou desconhecidas para a PROM que está sendo estudada. Relatar brevemente os principais resultados de cada propriedade de medida pode ajudar os leitores a avaliarem se a(s) propriedade(s) de medida é(são) suficiente(s) para a PROM na população de interesse.

Elementos essenciais

- Principais resultados apresentados para cada propriedade de medida investigada.

Exemplo 1

"Patients confirmed Patient-Reported Outcomes Measurement Information System®-Fatigue Short Form 7a (PROMIS-SF-7a) items were relevant to assessing fatigue, instructions and response options were clear, and its 7-day recall period was appropriate." [20]

Tradução: "Os pacientes confirmaram que os itens do *Patient-Reported Outcomes Measurement Information System®-Fatigue Short Form* 7a (PROMIS-SF-7a) eram relevantes para a avaliação da fadiga, que as instruções e opções de resposta eram claras e que o período de recordação de 7 dias era apropriado."

Exemplo 2

"WHODAS 2.0 presented a single factor by exploratory factor analysis and adequate model by confirmatory factor analysis (CFI = 0.924, TLI = 0.900, RMSEA = 0.038), excellent internal consistency (α = 0.89) for all items and an invariancy across geographic regions (ΔCFI ≤ 0.01 and ΔRMSEA < 0.015)." [18]

Tradução: "O WHODAS 2.0 apresentou um único fator pela análise fatorial exploratória e um modelo adequado pela análise fatorial confirmatória (CFI = 0,924, TLI = 0,900, RMSEA = 0,038), excelente consistência interna (α = 0,89) para todos os itens e uma invariância entre as regiões geográficas (ΔCFI ≤ 0,01 e ΔRMSEA < 0,015)."

Exemplo 3

"Construct and known-groups validity against selected PROs [Patient Reported Outcomes] were overall strong and within ranges hypothesized a priori." [21]

Tradução: "A validade do construto e de grupos conhecidos em relação aos desfechos relatados pelo paciente selecionados foi, em geral, forte e dentro das faixas pressupostas a priori."

Exemplo 4

"Convergent validity was confirmed with high-to-moderate correlations ($r \geq 0.4$) for 12/15 hypothesized relationships between pairs of domains and/or total scores. Discriminant validity was confirmed with low correlations ($r < 0.3$) observed for 5/7 hypothesized relationships. Known-groups validity was confirmed with significant differences ($p \leq 0.05$) in mean Congenital Thrombotic Thrombocytopenic Purpura-Patient Experience Questionnaire (cTTP-PEQ) scores between the two Patient Global Impression of Severity (PGI-S) groups for most domains and items at both timepoints. Cronbach's alpha was 0.88 at baseline and 0.91 at Day 14, confirming internal consistency of the instrument. Test-retest reliability was also confirmed with a high $ICC_{[agreement]}$ (0.96)." [22]

Tradução: "A validade convergente foi confirmada com correlações altas a moderadas ($r \geq 0,4$) para 12/15 das hipóteses entre pares de domínios e/ou pontuações totais. A validade discriminante foi confirmada com correlações baixas ($r < 0,3$) observadas para 5/7 das hipóteses. A validade de grupos conhecidos foi confirmada com diferenças significativas ($p \leq 0,05$) nas pontuações médias do *Congenital Thrombotic Thrombocytopenic Purpura-Patient Experience Questionnaire* (cTTP-PEQ) entre os dois grupos de *Patient Global Impression of Severity* (PGI-S) para a maioria dos domínios e itens em ambos os momentos. O alfa de Cronbach foi de 0,88 na linha de base e de 0,91 no 14º dia, confirmando a consistência interna do instrumento. A confiabilidade teste-reteste também foi confirmada com um alto $ICC_{[concordância]}$ (0,96)."

Exemplo 5

"Most results were in accordance with expectations (70-91% of hypotheses confirmed), with some exceptions for PROMIS Anxiety and Ability to Participate (60% and 42% of hypotheses confirmed, respectively). For PROMIS Anxiety and Depression correlations with the Global Rating Scales (GRS) were too low (0.04 and 0.20, respectively)." [16]

Tradução: "A maioria dos resultados estava de acordo com as expectativas (70-91% das hipóteses confirmadas), com algumas exceções para o PROMIS Ansiedade e Capacidade de Participação (60% e 42% das hipóteses confirmadas, respectivamente). Para o PROMIS Ansiedade e Depressão, as correlações com as *Global Rating Scales* (GRS) foram muito baixas (0,04 e 0,20, respectivamente)."

A5 - Discussão/Conclusões

Forneça uma breve declaração das implicações dos resultados no contexto das evidências existentes sobre a PROM.

Explicação: A seção de discussão/conclusões do resumo é muito importante, pois geralmente é a parte mais lida do documento. Os principais resultados do estudo devem ser apresentados de forma clara em relação aos objetivos do estudo. É importante discutir as implicações clínicas e para a pesquisa, bem como a possibilidade de generalização dos resultados. Além disso, caso o espaço permita, é importante discutir quais pesquisas adicionais são necessárias em relação à PROM avaliada. Por exemplo, você pode afirmar que a PROM pode ser usada (todas as propriedades de medida foram avaliadas e são suficientes), ou a PROM não deve ser usada (uma das propriedades de medida é insuficiente e precisa ser melhorada primeiro), ou a PROM tem potencial para ser usada, as propriedades de medida testadas são suficientes, mas algumas ainda estão faltando.

Elementos essenciais

- Conclusões no contexto das evidências existentes e necessidade de pesquisas futuras.

Exemplo 1

"Sufficient structural validity, internal consistency and measurement invariance were found, both in the entire sample and in subsamples with and without chronic diseases. Requirements for sufficient evidence for construct validity were (almost) met for most subscales. Future studies should investigate test-retest reliability, measurement error, and responsiveness of the PROMIS-29." [23]

Tradução: "Foram encontradas validade estrutural, consistência interna e invariância da medida suficientes, tanto na amostra total quanto em subamostras com e sem doenças crônicas. Os requisitos de evidência suficiente para a validade do construto foram (quase) atendidos para a maioria das subescalas. Estudos futuros devem investigar a confiabilidade teste-reteste, o erro de medida e a responsividade do PROMIS-29."

Exemplo 2

"An active collaboration with patients and health-care professionals has supported the co-production of a potential new PROM of axial spondyloarthritis (axSpA) fatigue, underpinned by strong evidence of face and content validity." [24]

Tradução: "Uma colaboração ativa com pacientes e profissionais de saúde apoiou a coprodução de uma possível nova PROM de fadiga da espondiloartrite axial, sustentado por fortes evidências de validade de face e conteúdo."

Exemplo 3

"The study findings show that the items in the SF-36v2® Health Survey Acute (SF-36v2) are applicable to adults with hypoparathyroidism (HP). (...) The SF-36v2 is therefore recommended for usage in clinical trials examining adults with HP, although it is recommended that this generic measure be supplemented with disease-specific instruments such as the recently developed Hypoparathyroidism Patient Experience Scale-Symptom (HPES-Symptom) and Hypoparathyroidism Patient Experience Scale-Impact (HPES-Impact) measures." [25]

Tradução: "Os resultados do estudo mostram que os itens do SF-36v2® *Health Survey Acute* (SF-36v2) são aplicáveis a adultos com hipoparatireoidismo (HP). (...) O SF-36v2 é, portanto, recomendado para uso em ensaios clínicos que examinam adultos com HP, embora seja recomendado que essa medida genérica seja complementada com instrumentos específicos da doença, como as medidas recentemente desenvolvidas *Hypoparathyroidism Patient Experience Scale-Symptom* (HPES-Symptom) e *Hypoparathyroidism Patient Experience Scale-Impact* (HPES-Impact)."

Exemplo 4

"The Italian Version of the Palatable Eating Motives Scale (PEMS-IT) appears to be an instrument with promising psychometric properties and potential applications in clinical settings. However, it also has some limitations, and future studies could focus on improving the semantic content of the elements to increase the overall utility and precision of the instrument." [26]

Tradução: "A versão italiana da *Palatable Eating Motives Scale* (PEMS-IT) parece ser um instrumento com propriedades psicométricas promissoras e aplicações potenciais em ambientes clínicos. No entanto, ela também tem algumas limitações, e estudos futuros poderiam se concentrar em melhorar o conteúdo semântico dos elementos para aumentar a utilidade e a precisão gerais do instrumento."

Seção do relato: Introdução

I1 - PROM
Especifique o nome e, se relevante, a versão e o(s) construto(s) da PROM.

Explicação: O leitor do seu artigo precisa entender claramente qual PROM está sendo estudada, por isso é importante fornecer informações importantes sobre a PROM (ou seja, o **nome** e, se aplicável, **a versão da PROM**). A versão da PROM deve ser fornecida, especialmente nos casos em que existem várias versões, como formulários curtos, traduções, variações nos bancos de itens ou versões para populações específicas (veja o item A1). **O(s) construto(s)** que a PROM pretende medir e a definição do(s) construto(s) também devem ser relatados. Idealmente, a definição do(s) construto(s) deve ser extraída das investigações originais de desenvolvimento do instrumento ou de trabalhos subsequentes que esclareçam o(s) construto(s). No(s) objetivo(s) do estudo descrito(s) na seção de introdução do seu documento (veja o item I4), não incluímos o relato do(s) construto(s) que a PROM pretende medir. Isso ocorre porque consideramos crucial a descrição do(s) construto(s) que a PROM pretende medir na introdução, e ela deve ser relatada nesse item. Pode ser útil usar um modelo conceitual, como o modelo proposto por Wilson e Cleary [27], o modelo da CIF [28], ou o filtro OMERACT [29], para definir o que é (e o que não é) medido pela PROM. Quando a PROM em estudo consiste em várias subescalas, é importante descrever o construto e a definição que cada uma dessas subescalas pretende medir. Às vezes, nessas escalas multidimensionais, uma pontuação total também é usada, referindo-se a um construto abrangente ou de ordem superior medido pela PROM como um todo, que também deve ser descrito e definido. Em alguns casos, uma citação do documento ou da investigação de desenvolvimento da PROM (e outras citações relevantes) pode ser suficiente, ou alguns detalhes (por exemplo, as definições dos construtos das subescalas) podem ser fornecidos na seção Métodos, em vez de na seção Introdução.

Elementos essenciais

- O nome e a versão da PROM.
- O nome de cada construto medido pela PROM e a definição de cada construto que está sendo investigado.
- Citações do(s) documento(s) de desenvolvimento original e outras citações altamente relevantes que descrevam a PROM, suas subescalas, se houver, e as definições de todos os construtos.

Exemplo 1

"(...) the Pelvic Floor Distress Inventory (PFDI-20) [ref] is a PROM that is often used in clinical practice and clinical trials to assess the distress caused by the presence of pelvic floor dysfunction (PFD) (...) PFDI-20 assesses the distress of pelvic organ prolapse (POP), anorectal and urinary symptoms in three subscales, respectively, Pelvic Organ Prolapse Distress Inventory (POPDI-6), Colorectal-Anal Distress Inventory (CRADI-8) and Urinary Distress Inventory (UDI-6) [ref]. According to the PFDI-20 development study, (...) [ref]." [30]

Tradução: "(...) o *Pelvic Floor Distress Inventory* (PFDI-20) [ref] é uma PROM frequentemente usada na prática clínica e em estudos clínicos para avaliar o desconforto causado pela presença de disfunção do assoalho pélvico (DAP) (...). O PFDI-20 avalia o desconforto de prolapso de órgãos pélvicos (POP), sintomas anorretais e urinários em três subescalas, respectivamente, *Pelvic Organ Prolapse Distress Inventory* (POPDI-6), *Colorectal-Anal Distress Inventory* (CRADI-8) e *Urinary Distress Inventory* (UDI-6) [ref]. De acordo com o estudo de desenvolvimento do PFDI-20, (...) [ref]."

Exemplo 2

"Chen et al. [ref] developed the Dysmenorrhea Symptom Interference (DSI) scale. This scale measures dysmenorrhea symptom interference with physical, mental, and social activities." [31]

Tradução: "Chen et al. [ref] desenvolveram a escala *Dysmenorrhea Symptom Interference* (DSI). Essa escala mede a interferência dos sintomas da dismenorreia nas atividades físicas, mentais e sociais."

I2 - População-alvo e contexto de uso

Especifique a população-alvo e o contexto de uso para o qual a PROM foi desenvolvida.

Explicação: Algumas PROMs são adaptadas a uma população-alvo específica durante o desenvolvimento para medir melhor um construto de interesse dentro desse grupo [12]. Essa **população-alvo** para a qual a PROM foi desenvolvida pode ser diferente da população de interesse na qual você avaliará a PROM posteriormente (ou seja, a população do estudo). Portanto, é importante especificar a população-alvo para a qual a PROM foi originalmente desenvolvida. Isso pode incluir características relevantes da doença (por exemplo, tipo, gravidade, etc.) e demográficas (por exemplo, idade, sexo, idioma/país, etc.) da população-alvo. Além disso, o **contexto de uso** para o qual a PROM foi desenvolvida pode ser diferente do contexto de uso para o qual ela está sendo avaliada agora. Portanto, é importante especificar o contexto de uso para o qual a PROM foi desenvolvida. O contexto de uso pode ser descrito em termos da finalidade da medição (por exemplo, avaliativa ou discriminativa) e de um ambiente específico (por exemplo, hospital, domicílio). Além disso, especifique o país em que a PROM original foi desenvolvida. A apresentação das características da população-alvo e do contexto de uso para o qual a PROM foi desenvolvida ajuda os leitores do seu estudo a estabelecer a adequação e a relevância da PROM para a aplicação pretendida e fornece uma compreensão abrangente de sua utilidade e aplicabilidade no contexto especificado.

Elementos essenciais

- Doença e características demográficas relevantes da população-alvo para a qual a PROM foi desenvolvida.
- Contexto de uso para o qual a PROM foi desenvolvida, em termos de finalidade da medição e cenário pretendido, incluindo o país.

Exemplo 1

"(...) the CLEFT-Q, a condition-specific PROM for people aged 8 to 29 years, born with a cleft lip or palate (CL/P) or other craniofacial conditions." [32]

Tradução: "(...) o CLEFT-Q, uma PROM específica para pessoas com idade entre 8 e 29 anos, nascidas com fissura labial ou palatina (FL/P) ou outras condições craniofaciais."

Exemplo 2

"Initially, the Hospital Anxiety and Depression Scale (HADS) was developed for adults receiving treatment for physical health problems in general hospitals [ref]." [33]

Tradução: "Inicialmente, a *Hospital Anxiety and Depression Scale* (HADS) foi desenvolvida para adultos que estavam recebendo tratamento para problemas de saúde física em hospitais gerais [ref]."

I3 - Estado do conhecimento e justificativa

Forneça uma descrição do conhecimento científico atual (o que se sabe e o que não se sabe) em relação às propriedades de medida da PROM. Explique por que o novo estudo é necessário. Forneça citações do(s) documento(s) original(is) de desenvolvimento.

Explicação: É imperativo fornecer uma descrição do conhecimento científico atual (o que é conhecido e o que não é conhecido) em relação às propriedades de medida relevantes da PROM que está sendo estudada, fornecendo citações do(s) documento(s) original(is) de desenvolvimento e outras citações relevantes desse conhecimento. Idealmente, se houver uma revisão sistemática das propriedades de medida da PROM, ela deverá ser resumida e citada. Dependendo da propriedade de medida sob investigação, talvez não seja necessário descrever todas as pesquisas anteriores sobre as propriedades de medida das PROMs. Em vez disso, pode ser apropriado discutir apenas as propriedades de medida relevantes para o objetivo do estudo. Por exemplo, se o foco for a validade estrutural, as informações sobre a validade do conteúdo são relevantes, mas os detalhes sobre outras propriedades de medida são menos cruciais. Da mesma forma, se o objetivo for avaliar a consistência interna, os dados sobre a validade estrutural devem ser descritos detalhadamente. Além disso, é necessário fornecer a justificativa para todos os objetivos do seu estudo. A justificativa do seu estudo deve incluir as lacunas na literatura, porque os objetivos são importantes e porque é necessário avaliar as propriedades de medida específicas na amostra específica do estudo. A avaliação das propriedades de medida é relevante porque uma PROM de alta qualidade permite que você confie na pontuação obtida com ela. Por exemplo, uma nova PROM é desenvolvida, mas nem todas as propriedades de medida foram estudadas ainda; ou se o seu objetivo for avaliar uma propriedade de medida específica em uma nova população e você fornecer uma visão geral dos estudos realizados em outras populações em que a mesma propriedade de medida foi avaliada, você pode mostrar que há uma lacuna no conhecimento, pois não se sabe se os resultados dos outros estudos podem ser generalizados para a sua população; ou um estudo sobre uma propriedade de medida específica de uma PROM foi realizado porque os resultados de uma revisão sistemática das propriedades de medida da PROM mostraram que essas propriedades de medida específicas não foram avaliadas de forma alguma, ou foram avaliadas apenas em estudos de baixa qualidade; ou há um estudo de alta qualidade na população de interesse, mas a intenção é repetir o estudo para reprodutibilidade dos resultados. Conforme observado acima, além das citações do(s) documento(s) original(is) de desenvolvimento da PROM, é importante fornecer outras citações altamente relevantes relacionadas à qualidade da PROM. Essas citações dão crédito ao desenvolvedor da PROM, ajudam seus leitores a encontrarem o

desenvolvimento original da PROM e os auxiliam com informações relevantes encontradas em outros estudos sobre as propriedades de medida da PROM.

Elementos essenciais

- Estado atual do conhecimento científico com relação às propriedades de medida relevantes para o foco do estudo.
- Lacunas na literatura.
- Justificativa de todos os objetivos do estudo.
- Citação do(s) documento(s) original(is) de desenvolvimento da PROM.
- Citação de qualquer documento relevante sobre as propriedades de medida da PROM.

Exemplo 1

"In this study, we assess the measurement invariance of the SF-12 with respect to multiple demographic characteristics that are highly relevant in health-related research: gender, age, educational level, and ethnicity. Previous studies have used such an approach to investigate measurement invariance of the SF-12 or the SF-36 [ref] and some of them indeed indicated a violation of the assumption of measurement invariance, with regard to one or more demographic characteristics [ref]. Most of these studies focused on the SF-36 or were performed among specific patient groups [ref]. (...) [ref] conducted the only study that explored DIF of the SF-12 in a general population sample. In this study, performed in the US, a comparison was made between white Americans, African-Americans and Hispanic Americans. (...) African and Hispanic American participants also gave higher ratings on this item [item measuring feeling calm and peaceful], compared with white Americans with a similar health status. (...) The results for these groups are not necessarily generalizable to European populations, however, no such studies have been performed in a European setting. Given the wide-spread use of the SF-12, there is a need for further studies on its measurement invariance in the general population. Not only do European countries host different ethnic minority groups compared to the US, also educational or other subgroups within the majority population might differ, e.g. with respect to cultural beliefs." [34]

Tradução: “Neste estudo, avaliamos a invariância da medida do SF-12 em relação a várias características demográficas que são altamente relevantes em pesquisas relacionadas à saúde: sexo, idade, nível educacional e etnia. Estudos anteriores usaram essa abordagem para investigar a invariância da medida do SF-12 ou do SF-36 [ref] e alguns deles de fato indicaram uma violação da suposição de invariância da medida, com relação a uma ou mais características demográficas [ref]. A maioria desses estudos se concentrou no SF-36 ou foi realizada em grupos específicos de pacientes [ref]. (...) [ref] conduziu o único estudo que explorou o [*Differential Item Functioning*] DIF do SF-12 em uma amostra da população geral. Nesse estudo, realizado nos Estados Unidos, foi feita uma comparação entre americanos brancos, afro-americanos e hispano-americanos. (...) Os participantes afro-americanos e hispano-americanos também atribuíram classificações mais altas a esse item [item que mede a sensação de calma e tranquilidade], em comparação com os americanos brancos com estado de saúde semelhante. (...) Os resultados para esses grupos não são necessariamente generalizáveis para as populações europeias, no entanto, nenhum estudo desse tipo foi realizado em um ambiente europeu. Dado o uso generalizado do SF-12, são necessários mais estudos sobre sua invariância da medida na população em geral. Os países europeus não só abrigam diferentes grupos étnicos minoritários em comparação com os EUA, mas também subgrupos educacionais ou outros subgrupos da população majoritária podem ser diferentes, por exemplo, com relação a crenças culturais.”

Exemplo 2

"The Dutch-Flemish versions of the PROMIS v1.0 adult sleep item banks showed adequate content validity in adolescents (...), meaning the items were considered relevant and comprehensible for adolescents, parents and sleep experts and no key issues were considered missing. Additional psychometric evaluation in a community sample of over 1,000 Dutch adolescents, however, showed that the one-factor models found in adults could not be replicated. Thus, the items used for adolescents did not reflect the same single construct measured in adults and the item banks were not unidimensional. Adaptation of the item banks to improve the unidimensionality needed for IRT analyses resulted in a shortened version of the Sleep-Related Impairment item bank (11 instead of 16 items) with adequate fit (comparative fit index [CFI] 0.98) and a shortened version of the Sleep Disturbance item bank (23 instead of 27 items) with fit indices just below the recommended value (CFI 0.90, recommended value > 0.95). (…) is necessary to optimize structural validity in order to enable CAT. In contrast, the Sleep-Related Impairment-11 item bank has sufficient structural validity and performed well as CAT. It can properly distinguish between clinical and non-clinical groups of adolescents, but test-retest reliability was just below the recommended criterion." [35]

Tradução: "As versões em holandês e flamengo dos bancos de itens de sono para adultos do PROMIS v1.0 apresentaram validade de conteúdo adequada em adolescentes (...), o que significa que os itens foram considerados relevantes e compreensíveis para adolescentes, pais e especialistas em sono, e nenhuma questão importante foi considerada ausente. No entanto, uma avaliação psicométrica adicional em uma amostra comunitária de mais de 1.000 adolescentes holandeses mostrou que os modelos de um fator encontrados em adultos não puderam ser replicados. Assim, os itens usados para adolescentes não refletiam o mesmo construto único medido em adultos e os bancos de itens não eram unidimensionais. A adaptação dos bancos de itens para melhorar a unidimensionalidade necessária para as análises da TRI resultou em uma versão reduzida do banco de itens do *Sleep-Related Impairment* (11 em vez de 16 itens) com ajuste adequado (*Comparative Fit Index* [CFI] 0,98) e uma versão reduzida do banco de itens do *Sleep Disturbance* (23 em vez de 27 itens) com índices de ajuste um pouco abaixo do valor recomendado (CFI 0,90, valor recomendado > 0,95). (...) é necessário otimizar a validade estrutural para permitir o [Teste adaptativo de computador] TAC. Em contraste, o banco de itens do *Sleep-Related Impairment*-11 tem validade estrutural suficiente e teve bom desempenho como TAC. Ele pode distinguir adequadamente entre grupos sintomáticos e não sintomáticos de adolescentes, mas a confiabilidade teste-reteste ficou um pouco abaixo do critério recomendado."

I4 - Objetivos

Forneça o(s) objetivo(s) da pesquisa, especificando (1) o nome (e versão, se relevante) da PROM, (2) as propriedades de medida que estão sendo avaliadas e (3) as características relevantes da amostra do estudo.

Explicação: Normalmente, o(s) objetivo(s) é(são) declarado(s) no final da seção Introdução. Em um artigo sobre as propriedades de medida de uma PROM, pelo menos um objetivo deve ser declarado sobre quais propriedades de medida estão sendo avaliadas (no entanto, objetivos adicionais podem ser investigados, por exemplo, referentes à tradução ou à avaliação da interpretabilidade da PROM). No objetivo do seu estudo, é importante especificar todas as **propriedades de medida** que estão sendo estudadas. Ao contrário de relatar o(s) objetivo(s) do estudo no resumo (veja o item A1), no qual permitimos relatar os domínios (por exemplo, validade, confiabilidade e responsividade), recomendamos que você descreva todas as propriedades de medida específicas (aspectos de (por exemplo, tradução, interpretabilidade)) nessa seção. Isso deixa claro para seus leitores exatamente quais (aspectos das) propriedades de medida foram avaliadas. Conforme mencionado anteriormente, recomendamos o uso da terminologia COSMIN de propriedades de medida. De acordo com o objetivo descrito no resumo, o **nome (e a versão, se relevante) da PROM** que está sendo estudada e uma descrição geral das **características dos participantes do estudo** (em termos de características da doença e características demográficas, se relevante) também devem ser especificados no objetivo (veja o item A1).

Elementos essenciais

• Objetivo(s) específico(s) da pesquisa, incluindo o nome - e a versão - da PROM, todas as propriedades de medida que estão sendo estudadas e as características dos participantes do estudo.

• Objetivos adicionais (por exemplo, sobre tradução ou interpretabilidade) podem ser especificados.

Exemplo 1

"This study's aim was to evaluate the test-retest reliability and measurement error of the Danish WHO-Five Well-being Index (WHO-5) and the Danish Problem Areas in Diabetes (PAID) questionnaire used in a PRO-based telehealth solution (DiabetesFlex) among patients with type 1 diabetes." [36]

Tradução: "O objetivo deste estudo foi avaliar a confiabilidade teste-reteste e o erro de medida do WHO-*Five Well-being Index* (WHO-5) dinamarquês e do questionário *Problem Areas in Diabetes* (PAID) dinamarquês usados em um serviço de telessaúde baseada em desfechos relatados pelo paciente (DiabetesFlex) entre pacientes com diabetes tipo 1."

Exemplo 2

"(…) the aim of this study is to examine measurement invariance of the SF-12 regarding age, gender, educational level, and ethnicity, using a multi-ethnic sample (HELIUS) of over 23,000 participants collected in the Netherlands." [34]

Tradução: "(...) o objetivo deste estudo é examinar a invariância da medida do SF-12 em relação à idade, gênero, nível educacional e etnia, usando uma amostra multiétnica (HELIUS) de mais de 23.000 participantes coletados nos Países Baixos."

Seção do relato: Métodos gerais

GM1 - Delineamento

Especifique os detalhes do delineamento do estudo usado para avaliar as propriedades de medida.

Explicação: Apresentar claramente os principais elementos do delineamento do estudo é aconselhável em todos os casos, particularmente quando o estudo está sendo usado em revisões sistemáticas ou meta-análises de instrumentos de desfechos. Essa clareza é fundamental para avaliar com precisão o risco de viés em seu estudo. No item A2, fornecemos recomendações específicas e exemplos adaptados a estudos com foco em diferentes propriedades de medida. Além disso, sugerimos que esses elementos sejam relatados no resumo e na seção de métodos. Se o seu estudo utilizar dados secundários, é importante citar o estudo original ou a fonte de dados na seção de métodos. Para estudos baseados em ensaios clínicos, recomendamos citar o registro do protocolo do ensaio.

Elementos essenciais

• O delineamento do estudo em termos de número de medidas (por exemplo, corte transversal, delineamento de teste-reteste, longitudinal), tipo de dados (por exemplo, qualitativo) e/ou exposição/intervenção (por exemplo, observacional, estudo clínico).

Exemplo 1

"This was a cross-sectional study to investigate the structural validity of the Roland-Morris Disability Questionnaire (RMDQ)." [15]

Tradução: "Este foi um estudo transversal para investigar a validade estrutural do *Roland-Morris Disability Questionnaire* (RMDQ)."

Exemplo 2

"This was a qualitative study to develop a conceptual model and develop/evaluate a new PRO instrument suitable for use in chronic ocular surface pain (COSP) treatment trials." [37]

Tradução: "Este foi um estudo qualitativo para desenvolver um modelo conceitual e desenvolver/avaliar um novo instrumento de desfechos relatados pelo paciente adequado para uso em estudos de tratamento de dor crônica na superfície ocular (DCSO)."

Exemplo 3

"De-identified patient responses to the Knee Injury and Osteoarthritis Outcome Score (KOOS) were obtained from the Surgical Outcome System (SOS), a patient-reported outcome database maintained by Arthrex (Naples, FL, USA)." [38]

Tradução: "As respostas anonimizadas dos pacientes ao K*nee Injury and Osteoarthritis Outcome Score* (KOOS) foram obtidas do *Surgical Outcome System* (SOS), um banco de dados de resultados relatados pelos pacientes mantido pela Arthrex (Naples, FL, EUA)."

GM2 - Participantes

Especifique como os participantes do estudo foram selecionados. Especifique os critérios de inclusão e exclusão.

Explicação: As PROMs devem ser testadas na população e no contexto em que se pretende usá-las [12], uma vez que os resultados das propriedades de medida podem variar em diferentes populações. É importante especificar quais critérios foram usados para selecionar os participantes (por exemplo, critérios de inclusão e exclusão), inclusive quem aplicou esses critérios (ou seja, seu treinamento ou experiência relevante) e quais métodos de amostragem foram usados para selecioná-los. Os critérios de inclusão e exclusão devem deixar claro de qual população a amostra foi selecionada. Portanto, normalmente são especificadas as características relevantes da doença (por exemplo, aqueles com a condição de interesse ou sem comorbidades, etc.) e as características demográficas (por exemplo, idade, sexo). Além da estratégia de amostragem (por exemplo, amostragem aleatória simples, amostragem estratificada, amostragem agrupada, amostragem por conveniência, etc.), devem ser descritas informações sobre o local do estudo ou locais de recrutamento. É necessário relatar tudo isso para que os leitores avaliem se a população de interesse foi amostrada de forma adequada e apropriada e se os resultados são aplicáveis a uma população de interesse do leitor.

Elementos essenciais

- Critérios de inclusão e exclusão.
- Métodos de amostragem dos participantes do estudo.

Exemplo 1

"Sixty adult idiopathic Parkinson disease (PD) patients, according to the criteria of the International Parkinson and Movement Disorder Society and the UK Parkinson's Disease Society Brain Bank that did not suffer from dementia or conditions that would interfere with the study's assessments were consecutively enrolled from June 2019 to December 2019." [39]

Tradução: "Sessenta pacientes adultos com doença de Parkinson (DP) idiopática, de acordo com os critérios da Sociedade Internacional de Parkinson e Distúrbios do Movimento e do Banco de Cérebros da Sociedade de Doença de Parkinson do Reino Unido, que não sofriam de demência ou condições que interferissem nas avaliações do estudo, foram incluídos consecutivamente de junho de 2019 a dezembro de 2019."

Exemplo 2

"Participants were invited to participate in the study through Facebook®, Instagram®, WhatsApp®, emails from universities and schools through a Google Forms link. Brazilian women aged between 14 and 42 years, with a report of dysmenorrhea in the last three months and able to speak, read and write in Brazilian Portuguese were included. The lower age limit is the mean menarche of Brazilian women [ref] and the upper limit decreases the probability of perimenopausal women [ref]. Pregnant women, women with up to 6 months of puerperium and transgender were excluded. We excluded transgender people due to hormonal issues that may interfere with the assessment of dysmenorrhea, which was beyond the scope of this study." [18]

Tradução: "As participantes foram convidadas a participar do estudo por meio do Facebook®, Instagram®, WhatsApp®, e-mails de universidades e escolas por meio de um link do Google Forms. Foram incluídas mulheres brasileiras com idade entre 14 e 42 anos, com relato de dismenorreia nos últimos três meses e capazes de falar, ler e escrever em português brasileiro. O limite inferior de idade é a menarca média das mulheres brasileiras [ref] e o limite superior diminui a probabilidade de mulheres na perimenopausa [ref]. Foram excluídas mulheres grávidas, mulheres com até 6 meses de puerpério e transgêneros. Excluímos os transgêneros devido a questões hormonais que podem interferir na avaliação da dismenorreia, o que estava fora do escopo deste estudo."

GM3 - Detalhes da PROM

Forneça detalhes sobre a versão original da PROM, bem como da versão da PROM que está sendo estudada, especifique a estrutura conceitual (modelo reflexivo/formativo), detalhes sobre a estrutura (o número de itens e subescalas), o idioma, as opções de resposta, o período de recordação, a direção da pontuação e o algoritmo de pontuação da PROM. Especifique como a PROM foi administrada (por exemplo, em que ambiente, modo de administração (por exemplo, papel, eletrônico), quais instruções foram dadas), inclusive o país em que foi administrada.

Explicação: É importante fornecer detalhes tanto sobre a PROM original quanto sobre a versão da PROM que está sendo estudada (ou declarar que as versões são as mesmas, se aplicável). Com essas informações, o leitor do seu estudo poderá determinar a generalização dos resultados do seu estudo. Um dos detalhes relevantes sobre as versões da PROM é o **idioma**. Se a PROM tiver sido traduzida, é importante informar detalhes sobre o processo de tradução (ou uma citação). Fornecer detalhes sobre o idioma em que a PROM está sendo avaliada, em combinação com o **país** em que a PROM foi desenvolvida (veja o item I2), também é importante para ajudar os leitores a entender o contexto cultural e linguístico do estudo (por exemplo, a versão holandesa da PROM foi aplicada na Bélgica ou a versão espanhola da PROM foi aplicada no norte do Chile). Em seguida, devem ser fornecidos detalhes sobre sua **estrutura conceitual**. Especificamente, deve ser informado se os itens de uma (sub)escala são baseados em um modelo reflexivo ou em um modelo formativo. Em um modelo reflexivo, o construto se manifesta nos itens e espera-se que os itens de uma escala se correlacionem até certo ponto. Em um modelo formativo, o construto é definido pelos itens, e os itens juntos formam o construto e não precisam ser correlacionados [40]. Para decidir qual modelo é aplicável (reflexivo ou formativo), existem algumas regras de decisão (por exemplo, recomenda-se a leitura do artigo de Jarvis et al. [41]). Recomendamos que você descreva a estrutura da PROM em detalhes, mencionando **quais e quantos itens estão incluídos (por subescala)**. Também é importante informar as **opções de resposta**, que pode assumir a forma de uma escala Likert, escala numérica, escala visual ou escala facial. O número de categorias ou o intervalo de opções de resposta e a que se refere cada nível de cada resposta (por exemplo, zero para nenhum e 10 para sempre, ou 1 para discordo totalmente e 5 para concordo totalmente) também devem ser descritos. O **período de recordação** refere-se ao período de tempo específico no qual os pacientes são instruídos a refletir sobre sua experiência ou estado relacionado à saúde ao responder aos itens de uma PROM (por exemplo, agora, nas últimas 24 horas, na última semana). A duração do período de recordação pode afetar significativamente o potencial de viés de recordação, enfatizando a importância da transparência nos relatórios para garantir a interpretação precisa dos dados e

resultados. Por fim, a **regra ou o algoritmo de pontuação** deve ser claramente descrito. O algoritmo de pontuação indica como as respostas do participante são calculadas em uma ou mais pontuações. Por exemplo, é possível calcular a média das pontuações dos itens em uma pontuação de escala não ponderada; ou algumas pontuações de itens recebem mais importância usando uma pontuação de soma ponderada; ou são usadas pontuações *Theta* baseadas em parâmetros da TRI ou das análises de Rasch. Também é importante informar o significado da **direção da pontuação** (ou seja, uma pontuação mais alta representa um resultado melhor ou pior?). Por fim, é importante informar o **modo de administração** das versões da PROM, pois isso pode influenciar a forma como a PROM é pontuada. O modo de administração refere-se ao desenvolvimento ou uso de uma versão em papel, uma versão eletrônica ou uma versão para entrevistadores. Além disso, é importante informar o dispositivo usado para entregar a PROM eletronicamente (por exemplo, um smartphone ou computador de tela pequena). Se um entrevistador estiver envolvido, é importante informar se, e como, o entrevistador foi treinado ou quanta experiência ele tinha para realizar as entrevistas.

Elementos essenciais

Tanto para a versão original da PROM quanto para a versão da PROM que está sendo usada:

- Idioma.
- A estrutura conceitual da PROM (por exemplo, modelo reflexivo ou formativo).
- Estrutura: número de itens por (sub)escala e ordenação dos itens por subescala.
- A escala de resposta e o período de recordação.
- O algoritmo de pontuação e a direção da pontuação.
- O modo de administração.

Exemplo Geral

"Esta PROM foi desenvolvida em [país] em [idioma] para medir [construto] em [período de recordação] em [população] a ser usada [modo de administração]. O [nome da PROM] é baseado na [estrutura conceitual] por causa de [explicação da estrutura conceitual]. O [nome da PROM] tem [número] itens divididos em [número] subescalas: [subescala A] [itens 1 a 4], [subescala B] [itens 5 a 7] e [subescala C] [itens 8 a 10]. As opções de resposta de cada item variam de x [significado] a y [significado], em que pontuações mais altas indicam [direção da pontuação] para [subescala A], (...) [subescala B], (...) [subescala C]. Para cada subescala, a pontuação é calculada pelo [algoritmo de pontuação]."

GM4 - Coleta de dados adicionais

Descreva por que e como outros dados foram coletados (por exemplo, construto e propriedades de medida dos instrumentos de comparação, características dos grupos que estão sendo comparados e justificativa para a escolha dos grupos), incluindo o modo de administração (por exemplo, papel, eletrônico).

Explicação: Dados adicionais são comumente coletados em estudos sobre propriedades de medida e usados para uma variedade de propósitos. Recomendamos informar por que e como os dados adicionais foram coletados. Os dados adicionais podem ser coletados para **descrever a amostra do estudo** (por exemplo, dados coletados sobre doenças e características demográficas), para **estratificar o grupo** para investigações específicas (por exemplo, para avaliar a validade transcultural/invariância da medida, grupo conhecido em testes de hipóteses para validade de construto ou responsividade) ou para **verificar se os participantes estavam estáveis** no construto entre medições repetidas (por exemplo, em um estudo sobre confiabilidade e erro de medida) ou **se** mudaram (por exemplo, para determinar os valores de mínima mudança importante/*Minimal Important Change* - MIC). A forma como os dados foram coletados pode ser baseada em perguntas de item único, como uma variável para descrever a amostra ou dividi-la em grupos (por exemplo, idade, sexo), ou uma escala de item único (por exemplo, uma pergunta de mudança global percebida); ou pode ser baseada em instrumentos de desfechos específicos (por exemplo, usados como instrumentos de comparação). Se outro instrumento de desfecho for usado, também é importante descrever as propriedades de medida desse instrumento de comparação. Se um instrumento de comparação for usado como padrão-ouro, é importante fornecer argumentos de que ele pode ser considerado como tal para o construto de interesse (ou seja, o construto que é medido pela PROM que está sendo estudada).

Elementos essenciais

- Descrição de por que e como os dados sobre doenças e características demográficas foram coletados.
- Descrição de por que e como a amostra foi separada em grupos, se aplicável.
- Descrição do(s) construto(s) que o(s) instrumento(s) comparador(es) pretende(m) medir e suas propriedades de medida.
- Descrição de por que o(s) instrumento(s) de comparação foi(ram) escolhido(s).
- Descrição de por que o instrumento de comparação é considerado um 'padrão-ouro'.
- Descrição de por que e como a estabilidade do paciente, se aplicável, foi verificada.

Exemplo 1

"Body appreciation scale (BAS-2) validated for Brazilian Portuguese was used to assess body appreciation. This one-dimensional PROM showed excellent test-retest reliability ($ICC_{agreement}$=0.81) (...) in Brazilian adults." [42]

Tradução: "A *Body appreciation scale* (BAS-2) validada para o português brasileiro foi usada para avaliar a apreciação corporal. Essa PROM unidimensional mostrou excelente confiabilidade teste-reteste ($ICC_{concordância}$ = 0,81) (...) em adultos brasileiros."

Exemplo 2

"Known-groups validity was evaluated by comparing the scale scores of the Rheumatoid Arthritis Symptom and Impact Questionnaire (RASIQ) between groups of participants with different levels of disease activity according to the following categories: Disease activity score-28 (DAS28) [ref] was used to split participants into four groups: disease remission (DAS28 ≤ 2.6), low disease activity (2.6 < DAS28 ≤ 3.2), moderate disease activity (3.2 < DAS28 ≤ 5.1) and high disease activity (DAS28 > 5.1); (…) Patient's assessment of arthritis pain (measured using a validated visual analog scale [VAS] scale [ref]) was used to split participants into three groups: no pain to mild pain (VAS ≤ 40), moderate pain (40 < VAS ≤ 70) and severe pain (VAS > 70)." [43]

Tradução: "A validade de grupos conhecidos foi avaliada comparando-se as pontuações da escala do *Rheumatoid Arthritis Symptom and Impact Questionnaire* (RASIQ) entre grupos de participantes com diferentes níveis de atividade da doença, de acordo com as seguintes categorias: O *Disease activity score*-28 (DAS28) [ref] foi usado para dividir os participantes em quatro grupos: remissão da doença (DAS28 ≤ 2,6), baixa atividade da doença (2,6 < DAS28 ≤ 3,2), atividade moderada da doença (3,2 < DAS28 ≤ 5,1) e alta atividade da doença (DAS28 > 5. 1); (...) A avaliação do paciente sobre a dor da artrite (medida por meio de uma escala visual analógica [EVA] validada [ref]) foi usada para dividir os participantes em três grupos: sem dor a dor leve (EVA ≤ 40), dor moderada (40 < EVA ≤ 70) e dor severa (EVA > 70)."

Exemplo 3

"In order to test the invariance of the factor model in the two samples, (...) Chile and Ecuador were selected for comparison. Although both are Spanish-speaking countries, they are nations with significant cultural differences. Based on the cultural dimensions of the Hofstede framework, Hofstede and Bond found differences between Chilean and Ecuadorian citizens for power distance, individualism vs. collectivism, masculinity vs. femininity and in the uncertainty avoidance dimension [ref]. (…) While in Chile the prevalence of malnutrition is very low, and while it is one of the South American countries with the highest proportion of obese adults (about 30%), Ecuador has a moderately high prevalence of malnutrition [ref]." [44]

Tradução: "Para testar a invariância do modelo de fatores nas duas amostras, (...) o Chile e o Equador foram selecionados para comparação. Embora ambos sejam países de língua espanhola, são nações com diferenças culturais significativas. Com base nas dimensões culturais da estrutura de Hofstede, Hofstede e Bond encontraram diferenças entre os cidadãos chilenos e equatorianos em relação à distância do poder, individualismo vs. coletivismo, masculinidade vs. feminilidade e na dimensão de prevenção de incertezas [ref]. (...) Enquanto no Chile a prevalência de desnutrição é muito baixa e é um dos países sul-americanos com a maior proporção de adultos obesos (cerca de 30%), o Equador tem uma prevalência moderadamente alta de desnutrição [ref]."

GM5 - Procedimentos de pontos de tempo

Forneça todos os pontos de tempo de todas as medições.

Explicação: Os pontos de tempo referem-se a ocasiões ou intervalos específicos durante o período do estudo em que os dados são coletados ou as medições são feitas. É importante relatar todos os pontos de tempo para todas as medições da PROM em estudo e todas as outras medições descritas neste item. Além disso, é importante fornecer uma justificativa clara para esses pontos de tempo em todas as investigações. Em estudos transversais, é necessário informar se todas as medições foram feitas dentro do mesmo período de tempo (por participante). Em um delineamento de medições repetidas (por exemplo, para avaliar a confiabilidade e o erro de medida) ou em um projeto longitudinal (por exemplo, para avaliar a responsividade), alguns ou todos os instrumentos de desfechos são repetidos ao longo do tempo. Recomendamos que você informe quais medições foram realizadas e em quais pontos específicos de acompanhamento. Quando a PROM em estudo e outras medições não foram realizadas nos mesmos pontos da linha de tempo do estudo, isso deve ser claramente descrito, e os motivos dessas diferenças também devem ser explicados.

Elementos essenciais

- Todos os pontos de tempo de todas as medidas para todos os instrumentos que estão sendo investigados.
- Número e intervalo de tempo de medições repetidas realizadas em estudos de confiabilidade e erro de medida, quando aplicável.
- Justificativa do motivo pelo qual as medições não foram feitas no mesmo momento, se aplicável.

Exemplo 1

"For data collection, an initial assessment was designed to provide the sample's demographical and clinical characteristics, including the following instruments: Widespread Pain Index (WPI), Symptom Severity Scale (SSS), FiRST® Fibromyalgia Rapid Screening Tool (FiRST®), Numerical Rating Scale (NRS), Fibromyalgia Impact Questionnaire-Revised (FIQ-R®) and Brief Pain Inventory (BPI®). Seven to ten days after answering the questionnaires, participants were asked to answer again the translated version of the FiRST® for test-retest reliability and measurement errors. This period of time followed the COSMIN recommendations in which seven days after the test is the minimum time for the patients not to have changed in the measured construct and not to remember their answers to the instrument." [45]

Tradução: "Para a coleta de dados, foi elaborada uma avaliação inicial para fornecer as características demográficas e clínicas dos participantes, incluindo os seguintes instrumentos: *Widespread Pain Index* (WPI), *Symptom Severity Scale* (SSS), FiRST® *Fibromyalgia Rapid Screening Tool* (FiRST®), *Numerical Rating Scale* (NRS), *Fibromyalgia Impact Questionnaire-Revised* (FIQ-R®) e *Brief Pain Inventory* (BPI®). Sete a dez dias após responderem aos questionários, os participantes foram solicitados a responder novamente à versão traduzida do FiRST® para verificar a confiabilidade teste-reteste e o erro de medida. Esse período de tempo seguiu as recomendações do COSMIN, segundo as quais sete dias após o teste é o tempo mínimo para que os pacientes não tenham mudado no construto medido e não se lembrem de suas respostas ao instrumento de desfecho."

Exemplo 2

"The study included 3 time points. Time 1 (T1) occurred 1 to 2 days before an early course of chemotherapy. Time 2 (T2) occurred 7 to 16 days following chemotherapy initiation at the time when the patient's nadir was projected. T3 occurred within 1 to 2 days preceding the next course of scheduled chemotherapy or approximately two weeks following T2." [46]

Tradução: "O estudo incluiu 3 pontos de tempo. O tempo 1 (T1) ocorreu 1 a 2 dias antes de um ciclo inicial de quimioterapia. O Tempo 2 (T2) ocorreu de 7 a 16 dias após o início da quimioterapia, no momento em que o nadir do paciente foi estimado. O T3 ocorreu dentro de 1 a 2 dias antes do próximo ciclo de quimioterapia programado ou aproximadamente duas semanas após o T2."

GM6 - Justificativa para o tamanho da amostra

Forneça uma justificativa para o tamanho da amostra para todas as análises das propriedades de medida (incluindo subgrupos).

Explicação: Informar a justificativa para o tamanho da amostra garante que o estudo tenha o poder adequado, reduzindo, assim, o risco de descobertas equivocadas. Recomenda-se fornecer uma justificativa para o tamanho da amostra que você pretende incluir - incluindo tamanhos de amostra para subgrupos - para cada análise de propriedades de medida e adicionar citações relevantes para a justificativa do tamanho da amostra escolhido. Em geral, para estudos sobre propriedades de medida, não há métodos formais para cálculos de tamanho de amostra. Portanto, é razoável fazer referência a regras práticas para todas as análises. No entanto, se uma fórmula para calcular o(s) tamanho(s) da amostra for usada, ela deve ser relatada. Em estudos de validade de conteúdo, uma abordagem qualitativa é frequentemente usada com o objetivo de alcançar uma geração abrangente de itens. Alguns autores recomendam um número mínimo de entrevistas em um estudo qualitativo (por exemplo, 12 a 20 [47], 10 a 15 [48]), enquanto outros enfatizam a importância de se atingir uma geração abrangente de itens [49]. Para um número mínimo de participantes em um estudo quantitativo, diferentes recomendações foram publicadas (por exemplo, 30 [50], 50 [51]). Na validade estrutural, as análises fatoriais e as análises da teoria da resposta ao item (TRI)/de Rasch exigem grandes tamanhos de amostra e podem se basear em várias diretrizes sobre o número necessário de participantes [52–54]. Observe que os testes de validade transcultural/invariância da medida exigem que os subgrupos sejam comparados, e os requisitos de tamanho da amostra se referem a ambos os subgrupos. Em estudos sobre confiabilidade e erro de medida com pontuações contínuas, há um aplicativo online desenvolvido para fornecer recomendações para tamanhos de amostra adequados com base em estudos de simulação [55]. Para pontuações nominais/ordinais, recomendamos que você consulte as recomendações baseadas em estatísticas kappa [56] e tabelas de contingência [57, 58]. Em estudos sobre validade de critério, teste de hipótese para validade de construto e responsividade, os requisitos de tamanho de amostra são baseados nas estatísticas usadas para cada hipótese. Ao informar a justificativa para o tamanho da amostra, você aumenta a transparência do seu estudo.

Elementos essenciais

- Justificativa para o tamanho da amostra para todas as análises das propriedades de medida (incluindo subgrupos).

Exemplo 1

Content validity: "We recruited at least three experts from each profession because COSMIN recommends that more than seven experts participate [ref]." [59]

Tradução - Validade de conteúdo: "Recrutamos pelo menos três especialistas de cada profissão porque o COSMIN recomenda que participem mais de sete especialistas [ref]."[58]

Exemplo 2

Structural and cross-cultural validity: "For factor analysis, the minimum sample size of 7 times the number of items in the instrument to be validated and greater than 100 individuals is advisable [ref]. Therefore, for this study, a minimum sample of 140 women was appropriate, since the Pelvic Floor Distress Inventory (PFDI-20) has 20 items." [30]

Tradução - Validade estrutural e validade transcultural: "Para a análise fatorial, é aconselhável o tamanho mínimo da amostra de 7 vezes o número de itens do instrumento a ser validado e mais de 100 indivíduos [ref]. Portanto, para este estudo, uma amostra mínima de 140 mulheres foi adequada, uma vez que o *Pelvic Floor Distress Inventory* (PFDI-20) tem 20 itens."

Exemplo 3

Hypothesis testing for construct validity: "(...) for the comparison of mean Oral Health Literacy Instrument - French (OHLI-F) scores with education levels or frequency of dental visits, the two-means formula was applied, with alpha = 0.05, power = 80%, standard deviation = 18 (15), expected difference = 10, and expected dropout rate = 20%, resulting in n = 51 per group. Since level of education and frequency of dental visit were classified into 3 groups, the total sample size required was 191 participants." [60]

Tradução - Testes de hipóteses para validade de construto: "(...) para a comparação das pontuações médias do *Oral Health Literacy Instrument - French* (OHLI-F) com os níveis de escolaridade ou frequência de visitas ao dentista, foi aplicada a fórmula de duas médias, com alfa = 0,05, poder = 80%, desvio padrão = 18 (15), diferença esperada = 10 e taxa de desistência esperada = 20%, resultando em n = 51 por grupo. Como o nível de escolaridade e a frequência de visitas ao dentista foram classificados em 3 grupos, o tamanho total da amostra necessário foi de 191 participantes."

Exemplo 4

Test-retest reliability and measurement error: "In this study, the sample size for intraclass correlation coefficient (ICC) was calculated with a correlation coefficient of 0.7 as an effect size [ref], power 0.8, and target width 0.3 of the 95% confidence interval of ICCs ($ICC_{2,1}$). The required same size was a minimum of 40 for 3 raters with systematic deviation based on the recommendation by [sample size decision assistant developed by Mokkink et al. [ref]." [61]

Tradução - Confiabilidade teste-reteste e erro de medida: "Neste estudo, o tamanho da amostra para o coeficiente de correlação intraclasse/*intraclass correlation coefficient* (ICC) foi calculado com um coeficiente de correlação de 0,7 como um tamanho de efeito [ref], poder 0,8 e largura-alvo 0,3 do intervalo de confiança de 95% dos ICCs ($ICC_{2,1}$). O mesmo tamanho necessário foi um mínimo de 40 para 3 avaliadores com desvio sistemático com base na recomendação de [assistente de decisão de tamanho de amostra desenvolvido por Mokkink et al. [ref]."

Exemplo 5

Responsiveness: "According to the study of [ref], and α: 0.05, β: 0.2, μ_1 i.e. menstrual problems domain mean score before oral contraceptive: 3.9; S_1: 1.4, μ_2 i.e. menstrual problems domain mean score after oral contraceptive: 5.26; S_2: 1.6, estimated 10% drop out the sample size were estimated at least 150 women per group (single and married) as followed [46]:

$$n = \frac{\left(z_{1-\frac{\alpha}{2}} + z_{1-\beta}\right)^2 \left(s_1^2 + S_2^2\right)}{\left(\mu_1 - \mu_2\right)^2}$$ [62]

Tradução - Responsividade: "De acordo com o estudo de [ref], e α: 0,05, β: 0,2, μ_1 ou seja, pontuação média do domínio de problemas menstruais antes do contraceptivo oral: 3,9; S_1: 1,4, μ_2 ou seja, pontuação média do domínio de problemas menstruais após o contraceptivo oral: 5,26; S_2: 1,6, estimou-se que 10% de abandono o tamanho da amostra foi estimado em pelo menos 150 mulheres por grupo (solteiras e casadas) conforme a seguir [46]:

$$n = \frac{\left(z_{1-\frac{\alpha}{2}} + z_{1-\beta}\right)^2 \left(s_1^2 + S_2^2\right)}{\left(\mu_1 - \mu_2\right)^2}$$

GM7 - Análises estatísticas

Descreva as análises estatísticas correspondentes a todos os objetivos (consulte as recomendações específicas das propriedades de medida). Descreva os critérios para boas propriedades de medida. Cite o pacote estatístico usado e a versão.

Explicação: Fornecer uma descrição detalhada de suas análises estatísticas permitirá que os leitores avaliem a validade de seus métodos e sua implementação, auxiliando na interpretação dos resultados e permitindo a replicação das análises [63]. Recomendamos que você relate todas as análises estatísticas correspondentes aos objetivos e às propriedades de medida. Especificamente, é imperativo relatar os testes e procedimentos estatísticos específicos usados (por exemplo, correlação de Pearson ou Spearman, em vez de apenas correlação). Também recomendamos que você descreva os critérios para boas propriedades de medida que você usa para avaliar os resultados e formular conclusões (ou seja, um valor predeterminado usado como referência para interpretar a análise) com uma citação. Em seguida, recomendamos que você informe o pacote estatístico utilizado, inclua os números das versões (por exemplo, STATA/MP 14.2) e citações, conforme apropriado. Descrevemos detalhes adicionais para relatar as análises de propriedades de medida específicas nos itens para propriedades de medida específicas abaixo.

Elementos essenciais

- Análises estatísticas correspondentes a todos os objetivos e propriedades de medida.
- Critérios para boas propriedades de medida para cada propriedade de medida avaliada.
- O pacote estatístico utilizado.

Exemplo 1

"For the confirmatory factor analysis, (...) we used the maximum likelihood robust, Root Mean Square Error of Approximation (RMSEA), Standardized Root Mean Squared Residual (SRMR), Comparative Fit Index (CFI) and Tucker-Lewis Index (TLI). The model was considered adequate when RMSEA and SRMR < 0.08, CFI and TLI > 0.90 [ref]. (...) Internal consistency was evaluated by Cronbach's Alpha (α), in which $\alpha = 0.70$ to 0.95 was considered adequate [ref]. For the hypothesis test for construct validity, Pearson correlations were calculated between the WHODAS 2.0 and numerical rating scale. We followed the correlation magnitudes by Cohen [ref]: weak ($r < 0.30$), moderate ($r = 0.30$ to 0.50) and strong ($r > 0.50$). We hypothesized a positive and moderate correlation between human functioning/disability and dysmenorrhea pain severity [ref]. (...) All analyses were conducted in Psych package in RStudio." [18]

Tradução: "Para a análise fatorial confirmatória, (...) usamos a máxima verossimilhança robusta, *Root Mean Square Error of Approximation* (RMSEA), *Standardized Root Mean Squared Residual* (SRMR), *Comparative Fit Index* (CFI) e *Tucker-Lewis Index* (TLI). O modelo foi considerado adequado quando RMSEA e SRMR < 0,08, CFI e TLI > 0,90 [ref]. (...) A consistência interna foi avaliada pelo Alfa de Cronbach (α), em que α = 0,70 a 0,95 foi considerado adequado [ref]. Para o teste de hipótese de validade do construto, as correlações de Pearson foram calculadas entre o WHODAS 2.0 e a escala de classificação numérica. Seguimos as magnitudes de correlação de Cohen [ref]: fraca (r < 0,30), moderada (r = 0,30 a 0,50) e forte (r > 0,50). Levantamos a hipótese de uma correlação positiva e moderada entre a funcionalidade/incapacidade humana e a gravidade da dor da dismenorreia [ref]. (...) Todas as análises foram realizadas no pacote Psych no RStudio."

GM8 - Dados ausentes

Descreva as abordagens para lidar com dados ausentes.

Explicação: Dados ausentes referem-se à ausência ou indisponibilidade de observações ou valores em um conjunto de dados que deveriam ser coletados durante um estudo. Em estudos sobre propriedades de medida, dados ausentes podem ocorrer porque um ou mais itens de uma PROM não foram preenchidas, ou a PROM inteira não foi preenchida em determinadas ocasiões. Além disso, outros dados que foram planejados para serem coletados (veja o item GM5) podem estar ausentes. Vários motivos podem levar à falta de dados, como erros na coleta de dados, perda de informações, desistência de participantes ou outros problemas encontrados durante o processo de pesquisa. Recomendamos que você informe e justifique quaisquer abordagens (por exemplo, imputação ou remoção de dados) que serão usadas para lidar com dados ausentes sobre as medidas da PROM em estudo e outros dados coletados. A comunicação de planos para lidar com dados ausentes garante transparência e reprodutibilidade na pesquisa ou na tomada de decisões baseadas em dados.

Elementos essenciais

• Abordagens para lidar com dados ausentes para cada propriedade de medida.

Exemplo 1

"Patients with a missing value for the studied Utrecht Symptom Diary (USD) item were excluded for analysis for that item." [64]

Tradução: "Os pacientes com um valor ausente para o item estudado do *Utrecht Symptom Diary* (USD) foram excluídos da análise desse item."

GM9 - Análise não planejada

Especifique as análises que não foram planejadas inicialmente e que foram realizadas, incluindo a justificativa para sua realização.

Explicação: Em um estudo bem elaborado, geral e idealmente, as perguntas de pesquisa e a análise estatística específica são planejadas antes do início da coleta de dados. Análises não planejadas podem surgir por vários motivos, como a descoberta de padrões inesperados ou novas questões de pesquisa que surgem durante o curso do estudo. Recomendamos que você especifique e forneça a justificativa para quaisquer análises não planejadas sobre propriedades de medida (ou seja, descreva o desvio do protocolo). O objetivo dessa explicação é ajudar os leitores a entender quais análises não planejadas foram feitas e o porquê.

Elementos essenciais

• Análises que não foram planejadas e sua justificativa.

Exemplo 1

"An initial confirmatory factor analysis (CFA) was conducted in Mplus on the polychoric correlation matrix with weighted least squares with mean and variance adjustment (WLSMV) estimation for each item bank separately, to examine the expected unidimensionality of the item banks. (...) When model fit for an item bank was not found to meet the recommended criteria of Comparative Fit Index (CFI) > 0.95, Tucker-Lewis Index (TLI) > 0.95, and Root Means Square error or Approximation (RMSEA) < 0.06, [ref] an exploratory approach similar to what was used in the item banks' primary development procedure was adopted [ref]. This provided the opportunity to further assess the dimensionality and to understand the misfit of the model in adolescents." [65]

Tradução: "Uma análise fatorial confirmatória (AFC) inicial foi conduzida no Mplus na matriz de correlação policórica com estimação de *weighted least squares with mean and variance adjustment* (WLSMV) para cada banco de itens separadamente, para examinar a unidimensionalidade esperada dos bancos de itens. (...) Quando se constatou que o ajuste do modelo para um banco de itens não atendia aos critérios recomendados de *Comparative Fit Index* (CFI) > 0,95, *Tucker-Lewis Index* (TLI) > 0,95 e *Root Means Square error or Approximation* (RMSEA) < 0,06, [ref] foi adotada uma abordagem exploratória semelhante à usada no procedimento de desenvolvimento primário dos bancos de itens [ref]. Isso proporcionou a oportunidade de avaliar ainda mais a dimensionalidade e entender o desajuste do modelo em adolescentes."

Seção do relato: Resultados gerais

GR1 - Características dos participantes

Forneça as características dos participantes do estudo, especificadas por subgrupo, se aplicável.

Explicação: Ao descrever a população do estudo em termos de doença e características demográficas relevantes, é ideal fornecer informações suficientes para que os leitores determinem a generalização e a aplicabilidade dos resultados a subgrupos ou populações específicas. Embora não haja uma resposta universal para a questão de quais características descritivas dos participantes do estudo devem ser relatadas, recomendamos que você relate **as características relevantes da doença** (por exemplo, gravidade, duração da doença) e outras **características demográficas relevantes** (por exemplo, sexo, idade). Se subgrupos de participantes forem usados em algumas das análises, recomendamos que você descreva as características tanto do grupo inteiro quanto do(s) subgrupo(s). Por exemplo, quando parte dos pacientes foi solicitada a participar do estudo de confiabilidade ou quando a amostra total foi dividida para a realização de análise fatorial exploratória em um subgrupo e análise fatorial confirmatória no outro subgrupo. Além disso, as pontuações da (sub)escala da PROM (linha de base e pontuações alteradas, se aplicável) também devem ser fornecidas para cada (sub)grupo, em termos de média (desvio padrão) ou mediana (intervalo ou pontuações interquartílicas). O fornecimento de dados descritivos detalhados ajuda a demonstrar se a amostra do estudo é representativa da população-alvo pretendida para a PROM e também facilita a interpretação das pontuações da PROM. É aceitável que os dados descritivos e as pontuações da PROM sejam resumidos em uma tabela.

Elementos essenciais

- Doença e características demográficas relevantes.
- Pontuações da PROM de linha de base e alteradas (ou seja, média e desvio padrão, ou mediana e pontuações interquartis ou intervalo).

Exemplo 1

"(...) Table 1 presents sample characteristics. Notably, the majority had a stroke (92%), and time since injury varied substantially across rehabilitation settings (Figure 1). In community-based rehabilitation, median time since injury was 9-months, ranging from 1-161 months. For stroke inpatients, median time since injury was 13 days, ranging from 5-47 days. (...) Statistics on Dutch Multifactor Fatigue Scale (DMFS) subscales are reported in Table 2."

Table 1. Characteristics of participants.

Characteristics	Community-Based (n = 100)	Sub-Acute (n = 49)	Full Sample (N = 149)
Sex, n (%)			
Female	37 (37.0)	19 (38.8)	56 (37.6)
Male	63 (63.0)	30 (61.2)	93 (62.4)
Age, M (SD)	54.3 (10.7)	66.7 (12.2)	58.4 (12.6)
Education, M (SD)[1]	15.0 (3.3)[2]	14.4 (4.0)	14.8 (3.6)[3]
Days since injury, M (SD)	540.8 (801.3)[4]	17.2 (10.5)[5]	367.4 (669.4)[6]
Type of injury, n (%)			
Stroke	89 (89.0)	49 (100.0)	138 (92.6)
Traumatic brain injury	5 (5.0)	0 (0.0)	5 (3.4)
Other[7]	6 (6.0)	0 (0.0)	6 (4.0)
Type of Stroke, n (%)			
Ischemic	59 (66.3)	33 (67.3)	92 (66.7)
Hemorrhagic	26 (29.2)	12 (24.5)	38 (27.5)
Both	3 (3.4)	0 (0.0)	3 (2.2)
Missing data	1 (1.2)	4 (8.2)	5 (3.6)
Previous brain injury, n (%)	16 (16.0)	9 (18.4)	25 (16.8)
Missing data	4 (4.0)	7 (14.3)	11 (7.4)

Note. [1] Reported in years. [2] n = 99. [3] n = 148. [4] n = 97. [5] n = 48. [6] n = 145. [7] Includes central nervous system infection, aneurisma surgery, anoxia, and hydrocephalus.

Table 2. Statistics on subscales of Dutch Multifactor Fatigue Scale.

Scale	Descriptive Statistics		
	N	M (SD)	Range
IF	147	38.7 (10.4)	11-55
SC	149	28.9 (7.6)	9-45
MF	148	25.2 (6.3)	7-35
PF	148	17.4 (5.4)	6-30
CF	148	15.3 (3.9)	7-25

[66]

Tradução: "(...) A Tabela 1 apresenta as características dos participantes. Notavelmente, a maioria teve um acidente vascular encefálico (92%) e o tempo desde que a lesão variou substancialmente entre os ambientes de reabilitação (Figura 1). Na reabilitação baseada na comunidade, o tempo médio desde a lesão foi de 9 meses, variando de 1 a 161 meses. Para pacientes internados por acidente vascular encefálico, o tempo médio desde a lesão foi de 13 dias, variando de 5 a 47 dias. (...) As estatísticas das subescalas da *Dutch Multifactor Fatigue Scale* (DMFS) estão relatadas na Tabela 2."

Tabela 1. Características dos participantes.

**Características**	**Baseado na comunidade (n = 100)**	**Subaguda (n = 49)**	**Amostra completa (N = 149)**
Sexo, n (%)			
Feminino	37 (37,0)	19 (38,8)	56 (37,6)
Masculino	63 (63,0)	30 (61,2)	93 (62,4)
Idade, M (DP)	54,3 (10,7)	66,7 (12,2)	58,4 (12,6)
Educação, M (DP)[1]	15,0 (3,3)[2]	14,4 (4,0)	14,8 (3,6)[3]
Dias desde a lesão, M (DP)	540,8 (801,3)[4]	17,2 (10,5)[5]	367,4 (669,4)[6]
Tipo de lesão, n (%)			
Acidente vascular encefálico	89 (89,0)	49 (100,0)	138 (92,6)
Lesão cerebral traumática	5 (5,0)	0 (0,0)	5 (3,4)
Outro[7]	6 (6,0)	0 (0,0)	6 (4,0)
Tipo de acidente vascular encefálico, n (%)			
Isquêmico	59 (66,3)	33 (67,3)	92 (66,7)
Hemorrágico	26 (29,2)	12 (24,5)	38 (27,5)
Ambos	3 (3,4)	0 (0,0)	3 (2,2)
Dados faltantes	1 (1,2)	4 (8,2)	5 (3,6)
Lesão cerebral anterior, n (%)	16 (16,0)	9 (18,4)	25 (16,8)
Dados faltantes	4 (4,0)	7 (14,3)	11 (7,4)

Nota. [1] Relatado em anos. [2] n = 99. [3] n = 148. [4] n = 97. [5] n = 48. [6] n = 145. [7] Inclui infecção do sistema nervoso central, cirurgia de aneurisma, inóxia e hidrocefalia.

Tabela 2. Estatísticas sobre subescalas da *Dutch Multifactor Fatigue Scale*.

**Escala**	**Estatísticas descritivas**		
	**N**	**M (DP)**	**Faixa**
IF	147	38,7 (10,4)	11-55
SC	149	28,9 (7,6)	9-45
MF	148	25,2 (6,3)	7-35
PF	148	17,4 (5,4)	6-30
CF	148	15,3 (3,9)	7-25

GR2 - Tamanho da amostra

Forneça o número total de participantes incluídos no estudo e o tamanho da amostra para cada análise.

Explicação: São necessários tamanhos de amostra adequados para estimar os resultados com precisão suficiente para tirar conclusões válidas. Recomendamos que você forneça o número total de participantes incluídos no estudo, bem como para cada subgrupo e em cada análise (por propriedade de medida). Por exemplo, em um estudo sobre validade transcultural/invariância da medida, recomendamos que você informe o tamanho da amostra para cada grupo incluído na análise; ou o tamanho da amostra em um estudo de confiabilidade, quando apenas parte de todos os participantes foi convidada a participar desse subestudo. O relato dessas informações aumenta a transparência do seu estudo e permite que os leitores comparem o tamanho previsto da amostra (conforme descrito na seção de método) com o tamanho real da amostra do seu estudo.

Elementos essenciais

- O número total de participantes incluídos no estudo.
- O tamanho da amostra para cada análise e (sub)grupos.

Exemplo 1

“A total of 172 atrial fibrillation (AF) patients visited the Cardiology Clinic from January to March 2022, of whom 133 patients met the inclusion criteria. Twelve patients were excluded due to cognitive impairment, recent hospitalization, or physical disabilities. Of 121 patients who consented to participate, 6 were excluded because of acute conditions and 13 others could not be contacted for the retest procedure, leaving 102 patients who were included in the analyses (…).” [67]

Tradução: “Um total de 172 pacientes com fibrilação atrial (FA) visitou a Clínica de Cardiologia de janeiro a março de 2022, dos quais 133 pacientes atenderam aos critérios de inclusão. Doze pacientes foram excluídos devido a comprometimento cognitivo, hospitalização recente ou deficiências físicas. Dos 121 pacientes que consentiram em participar, 6 foram excluídos devido a condições agudas e outros 13 não puderam ser contatados para o procedimento de reteste, resultando 102 pacientes que foram incluídos nas análises (...).”

Exemplo 2

"Structural validity was carried out on the original sample of 262 patients (...). Internal consistency was calculated using the baseline scores of the 262 patients (...). Test-retest reliability was assessed on a sample of 64 patients from the general sample who completed the Michigan Hand Outcomes Questionnaire (MHQ) for the second time. From the initial sample, 222 patients completed the MHQ, Disability of Arm, Shoulder and Hand (DASH) and DASH-work as well as grip strength and Visual Analogue Scale (VAS) pain at 5 weeks after baseline." [68]

Tradução: "A validade estrutural foi realizada na amostra original de 262 pacientes (...). A consistência interna foi calculada usando as pontuações de linha de base dos 262 pacientes (...). A confiabilidade teste-reteste foi avaliada em uma amostra de 64 pacientes da amostra geral que preencheram o *Michigan Hand Outcomes Questionnaire* (MHQ) pela segunda vez. Da amostra inicial, 222 pacientes preencheram o MHQ, o *Disability of Arm, Shoulder and Hand* (DASH) e o DASH-work, bem como a força de preensão e a escala visual analógica (EVA) de dor 5 semanas após a linha de base."

GR3 - Dados ausentes

Forneça a quantidade (proporção ou contagem) e os motivos da falta de dados para cada análise da PROM e para quaisquer análises de outros instrumentos de desfechos.

Explicação: Dados ausentes são a ausência ou indisponibilidade de determinadas observações ou valores em um conjunto de dados que deveriam ser coletados durante um estudo. Você deve fornecer a quantidade e os motivos (por exemplo, erros na coleta de dados, perda de informações ou outros problemas encontrados durante o processo de pesquisa) para os dados ausentes em cada análise da PROM ou de outros instrumentos de desfechos por ponto de tempo. Os dados ausentes podem ocorrer, por exemplo, se alguns itens não foram respondidos pelos pacientes ou se os pacientes em um estudo longitudinal desistiram ou não conseguiram concluir qualquer medição em um ponto de tempo específico. A comunicação de dados ausentes é um aspecto fundamental da transparência em um estudo das propriedades de medida das PROMs e permite que os leitores entendam as limitações do estudo e da PROM e tomem decisões informadas sobre como interpretar os resultados.

Elementos essenciais

- Quantidade e motivos da falta de dados para cada análise da PROM em estudo, em qualquer momento.
- Quantidade e motivos da falta de dados em outros instrumentos de desfechos.

Exemplo 1

"(...) one patient (0.7%) and 38 patients (26%) did not answer the WHO-Five Well-being Index (WHO-5) or Problem Areas in Diabetes (PAID) questionnaires, respectively. Furthermore, missing data in single items ranged from 0.7% in the general health status item to 25.3% in the decreased feet feeling and pain in feet items. The highest proportion of missing data was found in the erectile dysfunction item (60.3%) (Table 5). Informal conversations with patients related to non-response indicated two primary reasons for not completing the second questionnaire: 1). Some patients did not understand the purpose of the second questionnaire as they had already answered the same questionnaire at test 1, and 2). Some patients expressed a lack of energy to fill in an additional questionnaire." [36]

Exemplo 1

Tradução: "(...) um paciente (0,7%) e 38 pacientes (26%) não responderam aos questionários WHO-*Five Well-being Index* (WHO-5) ou *Problem Areas in Diabetes* (PAID), respectivamente. Além disso, os dados faltantes em itens individuais variaram de 0,7% no item de estado geral de saúde a 25,3% nos itens sobre diminuição da sensibilidade e dor nos pés. A maior proporção de dados faltantes foi encontrada no item de disfunção erétil (60,3%) (Tabela 5). As conversas informais com os pacientes relacionadas à não resposta indicaram dois motivos principais para o não preenchimento do segundo questionário: 1). Alguns pacientes não entenderam a finalidade do segundo questionário, pois já haviam respondido ao mesmo questionário no teste 1; e 2). Alguns pacientes expressaram falta de motivação para preencher um questionário adicional."

GR4 - Resultados

Descreva os resultados correspondentes a todos os objetivos (consulte as recomendações específicas das propriedades de medida).

Explicação: Os resultados correspondentes a cada objetivo fornecem uma compreensão abrangente das descobertas do seu estudo. Isso facilita a interpretação dos seus resultados no contexto de perguntas ou objetivos de pesquisa específicos, ajudando os leitores a discernir a relevância e as implicações do estudo. Recomendamos que você descreva todos os resultados de acordo com todos os objetivos. Os resultados podem ser relatados em tabelas e os detalhes em Apêndices/material suplementar. Por exemplo, para a validade estrutural, os índices de ajuste dos modelos testados podem ser relatados em uma tabela no manuscrito, enquanto as cargas fatoriais dos itens de diferentes modelos podem ser relatadas em apêndices/materiais suplementares. A apresentação de todos os resultados ajuda os leitores a entender o objetivo e os resultados do seu estudo, especificamente quando os resultados forem usados em uma futura revisão sistemática de instrumentos de desfechos para facilitar a seleção de PROMs. Os detalhes por propriedade de medida são fornecidos abaixo nas recomendações por propriedade de medida.

Elementos essenciais

- Descrição dos resultados correspondentes a todos os objetivos.
- Os resultados podem ser fornecidos em texto e/ou em tabelas, e os resultados detalhados podem ser fornecidos em um apêndice/material suplementar.

Exemplo 1

Structural validity and internal consistency: "Structural validity was evaluated through exploratory factor analysis (EFA) and confirmatory factor analysis (CFA) on 400 women of reproductive age (conducting EFA and CFA) on same sample). Kaiser-Meyer-Olkin (KMO) value equaled 0.726. The KMO > 0.7 confirmed the significance of Bartlett's test and model adequacy (P ≤ 0.001). Moreover, the three-factor structure in the EFA process obtained a total variance of 58.15% (Table 3). The first factor was Pelvic Organ Prolapse Distress Inventory-6 (POPDI-6), which comprised 6 items that explained 18.25% of the total variance. It should be mentioned that item 6 of factor POPDI-6 has a factor loading of less than 0.3, but according to the opinion of the research team, this item had a high importance and weight and we could not remove it, but it should be noted that in the CFA, this item was significant (p < 0.001), so it was not removed. Colorectal-Anal Distress Inventory-8 (CRADI-8) was the second factor that covered 8 items that explained 17.10% of the total variance. Finally, the third factor included Urinary Distress Inventory 6 (UDI-6) comprised 6 items that explained 22.80% of the whole variance.
In the CFA phase, three factors were obtained and then used in EFA by CFA. According to results (RMSEA = 0.07, SRMR = 0.07, TLI = 0.97, CFI = 0.99, $\chi 2/df$ (normed chi-square) = 3.19), this model had an optimal fit. Hence, factor structure could be confirmed (Table 4)." [69]

Tradução: *Validade estrutural e consistência interna:* "A validade estrutural foi avaliada por meio da análise fatorial exploratória (AFE) e da análise fatorial confirmatória (AFC) em 400 mulheres em idade reprodutiva (realizando AFE e AFC na mesma amostra). O valor de *Kaiser-Meyer-Olkin* (KMO) foi igual a 0,726. O KMO > 0,7 confirmou a importância do teste de Bartlett e a adequação do modelo (P ≤ 0,001). Além disso, a estrutura de três fatores no processo AFE obteve uma variância total de 58,15% (Tabela 3). O primeiro fator foi o *Pelvic Organ Prolapse Distress Inventory*-6 (POPDI-6), composto por 6 itens que explicaram 18,25% da variância total. Deve-se mencionar que o item 6 do fator POPDI-6 tem uma carga fatorial inferior a 0,3, mas, de acordo com a opinião da equipe de pesquisa, esse item tinha grande importância e peso e não pudemos removê-lo, mas deve-se observar que, na AFC, esse item foi significativo (p < 0,001), portanto não foi removido. O *Colorectal-Anal Distress Inventory*-8 (CRADI-8) foi o segundo fator que abrangeu 8 itens que explicaram 17,10% da variância total. Por fim, o terceiro fator, o *Urinary Distress Inventory*-6 (UDI-6), incluiu 6 itens que explicaram 22,80% de toda a variância.

Na fase de AFC, três fatores foram obtidos e, em seguida, usados na AFE por AFC. De acordo com os resultados (RMSEA = 0,07, SRMR = 0,07, TLI = 0,97, CFI = 0,99, χ2/gl (qui-quadrado normalizado) = 3,19), esse modelo teve um ajuste ideal. Portanto, a estrutura do fator pode ser confirmada (Tabela 4).”

Exemplo 2

Internal consistency, test-retest reliability and measurement error: “Cronbach’s alpha ranged between 0.84 and 0.90 (Table 3). The lower limits of all ICC confidence intervals were greater than 0.7. For each of the short forms, 18% to 20% of the test-retest sample had the best possible scores on both test occasions. Both the standard error of measurement (SEM_{agr}) and smallest detectable changes (SDC_{90}) were higher for pain intensity (PAIN) and pain interference (PI) compared to physical function (PF). The effect size based on SEM_{agr} was around 5 to 6 for all three short forms, and smaller than that for the Oxford Knee Score (OKS) (9.5).”

Table 3. Reliability, agreement and smallest detectable change

	Crobanch’s α[a]	ICC[a]	SEM_{agr}	SDC90	Effect size based on SEM_{agr}
PROMIS PAIN	0.84 (0.79 to 0.88)	0.93 (0.88 to 0.96)	3.55	8.28	5.51
PROMIS PI	0.90 (0.78 to 0.92)	0.90 (0.83 to 0.94)	3.34	7.78	4.84
PROMIS PF	0.88 (0.74 to 0.91)	0.97 (0.94 to 0.98)	1.72	4.02	6.33
OKS	-	-	1.78	4.15[b]	9.52

ICC intraclass correlation coefficient; SEM_{agr} agreement for T-scores assessed using standard error of measurement from test-retest; SDC90 smallest detectable change for individuals that can be considered above the measurement error with a 90% confidence level; Effect size based on SEM_{agr} calculated as absolute value of the mean change score divided by SEM_{agr} PROMIS Patient Reported Outcomes Measurement Information System; PAIN pain intensity; PI pain interference; PF physical function; OKS Oxford Knee Score

[a]95% confidence interval in parentheses

[b]According to: Beard DJ, Harris K, Dawson J, Doll H, Murray DW, Carr AJ, et al. Meaningful changes for the Oxford hip and knee scores after joint replacement surgery. J Clin Epidemiol. 2015;68(1):73-9

[70]

Tradução: *Consistência interna, confiabilidade teste-reteste e erro de medida*: "O alfa de Cronbach variou entre 0,84 e 0,90 (Tabela 3). Os limites inferiores de todos os intervalos de confiança do ICC foram maiores que 0,7. Para cada um dos formulários curtos, 18% a 20% da amostra de teste-reteste tiveram as melhores pontuações possíveis em ambas as ocasiões de teste. Tanto o *standard error of measurement* (SEM_{agr}) quanto as *smallest detectable changes* (SDC90) foram maiores para a intensidade da dor (PAIN) e a interferência da dor (PI) em comparação com a função física (PF). O tamanho do efeito com base no SEM_{agr} foi de cerca de 5 a 6 para todas as três formas curtas e menor do que o *Oxford Knee Score* (OKS) (9,5)."

Tabela 3. Confiabilidade, concordância e menor alteração detectável

	Alfa de Crobanch[a]	ICC[a]	SEM_{agr}	SDC90	Tamanho do efeito com base no SEM_{agr}
PROMIS PAIN	0,84 (0,79 to 0,88)	0,93 (0,88 to 0,96)	3,55	8,28	5,51
PROMIS PI	0,90 (0,78 to 0,92)	0,90 (0,83 to 0,94)	3,34	7,78	4,84
PROMIS PF	0,88 (0,74 to 0,91)	0,97 (0,94 to 0,98)	1,72	4,02	6,33
OKS	-	-	1,78	4,15[b]	9,52

ICC *intraclass correlation coefficient*; SEM_{agr} concordância para pontuações T avaliadas usando o *standard error of measurement* do teste-reteste; SDC90 *smallest detectable change* para indivíduos que pode ser considerada acima do erro de medida com um nível de confiança de 90%; Tamanho do efeito com base no SEM_{agr} calculado como valor absoluto da pontuação de alteração média dividido pelo SEM_{agr} PROMIS *Patient Reported Outcomes Measurement Information System*; PAIN intensidade da dor; PI interferência da dor; PF função física; OKS *Oxford Knee Score*

[a]Intervalo de confiança de 95% entre parênteses

[b]De acordo com: Beard DJ, Harris K, Dawson J, Doll H, Murray DW, Carr AJ, et al. Meaningful changes for the Oxford hip and knee scores after joint replacement surgery. J Clin Epidemiol. 2015;68(1):73-9

Seção do relato: Discussão/conclusões

DC1 - Evidência de propriedade de medida

Forneça os principais achados e se cada propriedade de medida é suficiente ou insuficiente e sua justificativa.

Explicação: A seção de discussão do seu estudo desempenha um papel crucial na interpretação e contextualização das principais descobertas do seu estudo com a literatura, ajudando os leitores a entender as implicações dos seus resultados e comparar com outros estudos. Recomendamos que você declare as principais descobertas para cada propriedade de medida, se a PROM é suficiente ou insuficiente, e compare suas descobertas com outros estudos da mesma PROM. Oferecer um resumo das descobertas ajuda seus leitores a compreender rapidamente os principais resultados dos seus estudos. Saber se uma PROM tem confiabilidade, validade e/ou responsividade suficientes informa sua seleção e aplicabilidade em diferentes cenários. PROMs com propriedades de medida insuficientes não devem ser recomendadas para uso na prática clínica ou pesquisa. Ao comparar suas descobertas com outros estudos da mesma PROM, você ajuda seus leitores a entender a generalização dos resultados na mesma população.

Elementos essenciais

- As principais conclusões de cada propriedade de medida.
- Se cada propriedade de medida é suficiente ou insuficiente.

Exemplo 1

“Cronbach’s alpha values were all around 0.9 or higher, except for sleep disturbance (alpha=0.75), thereby showing sufficient internal consistency.” [23]

Tradução: “Os valores de alfa de Cronbach foram todos em torno de 0,9 ou mais, exceto para distúrbios do sono (alfa=0,75), mostrando assim consistência interna suficiente.”

Exemplo 2

"Of our a priori defined hypotheses 78% could be confirmed, thereby meeting the 75% required for sufficient construct validity according to the COSMIN criteria for good measurement properties [ref]. For most domains, this criterion was also (almost) met. Although we based our hypothesis on analyses with other Dutch datasets [ref] and previous experiences, one should note that a one point difference, as used in some hypotheses, might not (always) be meaningful. It is not yet clear what a minimal important difference in scores between groups is for PROMIS measures, but most studies suggest a within-person change of at least three points to be meaningful [ref]." [23]

Tradução: "Das nossas hipóteses definidas a priori, 78% puderam ser confirmadas, atendendo, assim, aos 75% necessários para uma validade de construto suficiente, de acordo com os critérios COSMIN para boas propriedades de medida [ref]. Para a maioria dos domínios, esse critério também foi (quase) atendido. Embora tenhamos baseado nossa hipótese em análises com outros conjuntos de dados holandeses [ref] e em experiências anteriores, é preciso observar que uma diferença de um ponto, conforme usada em algumas hipóteses, pode não ser (sempre) significativa. Ainda não está claro o que é uma diferença mínima importante nas pontuações entre os grupos para as medidas PROMIS, mas a maioria dos estudos sugere uma mudança individual de pelo menos três pontos para ser significativa [ref]."

DC2 - Relevância prática

Discuta a relevância prática dos achados em termos de recomendações para usar ou não a PROM.

Explicação: A seção Discussão pode fornecer uma oportunidade para contextualizar seus resultados dentro de aplicações da PROM no mundo real. Com base em suas descobertas e resultados de outros estudos sobre a mesma propriedade de medida da mesma PROM, recomendamos que você discuta a relevância prática para a seleção do instrumento (ou seja, explique se a PROM pode ser recomendada para uso, se mais pesquisa é necessária ou se a PROM em sua forma atual não deve ser usada em pesquisa e/ou prática clínica devido aos resultados insuficientes ou inconsistentes). A descrição da relevância prática pode orientar o processo de seleção para futuros leitores, fornecendo informações sobre como e quando a PROM pode ser aplicada/utilizada (por exemplo, como um instrumento de avaliação para monitorar pacientes ao longo do tempo na área da saúde ou em pesquisas). Ao incluir uma discussão sobre a relevância prática, você pode destacar como o uso dos instrumentos pode contribuir para melhores resultados para os pacientes (por exemplo, melhor monitoramento dos sintomas). Essas informações são cruciais para embasar decisões sobre o uso de PROMs e, em última análise, para a padronização do uso de PROMs e para melhorar os resultados em vários campos.

Elementos essenciais

• Relevância prática para a recomendação/seleção e uso do instrumento.

Exemplo 1

"Our research contributes to the field of female sexuality and sexual medicine in Romania, for research, as well as teaching purposes and in clinical practice. The clinical utility of the Romanian version of the female sexual function index (FSFI-RO) lies in its potential to enhance the assessment and understanding of female sexual dysfunction (FSD) in clinical practice and research settings. (...) the Romanian female sexual function index (FSFI) can be used as an outcome measure in clinical trials or intervention studies. Its sensitivity to change allows researchers and clinicians to evaluate the effectiveness of various treatments or interventions for female sexual dysfunction. By administering the Romanian FSFI before and after treatment, researchers can assess the impact of interventions on different aspects of sexual function and quantify the magnitude of change. This not only contributes to the evidence base for treatment effectiveness but also helps guide clinical decision-making. Moreover, the availability of

the Romanian FSFI can facilitate cross-cultural and international research collaborations. Researchers and clinicians in Romania can use the same standardized measurement tool as their counterparts in other countries, allowing for direct comparisons and meta-analyses across different cultural and linguistic contexts. This enables a more comprehensive understanding of the global prevalence, determinants, and consequences of female sexual dysfunction." [71]

Tradução: "Nossa pesquisa contribui para o campo da sexualidade feminina e da medicina sexual na Romênia, para fins de pesquisa, ensino e prática clínica. A utilidade clínica da versão romena do *Female Sexual Function Index* (FSFI-RO) está em seu potencial para aprimorar a avaliação e a compreensão da disfunção sexual feminina (DSF) na prática clínica e em ambientes de pesquisa. (...) o *Female Sexual Function Index* (FSFI) romeno pode ser usado como uma medida de resultado em estudos clínicos ou de intervenção. Sua sensibilidade à mudança permite que pesquisadores e clínicos avaliem a eficácia de vários tratamentos ou intervenções para a disfunção sexual feminina. Ao administrar o FSFI romeno antes e depois do tratamento, os pesquisadores podem avaliar o impacto das intervenções em diferentes aspectos da função sexual e quantificar a magnitude da mudança. Isso não apenas contribui para a base de evidências da eficácia do tratamento, mas também ajuda a orientar a tomada de decisões clínicas. Além disso, a disponibilidade do FSFI romeno pode facilitar colaborações de pesquisa interculturais e internacionais. Os pesquisadores e clínicos da Romênia podem usar a mesma ferramenta de medição padronizada que seus colegas de outros países, permitindo comparações diretas e metanálises em diferentes contextos culturais e linguísticos. Isso permite uma compreensão mais abrangente da prevalência global, dos determinantes e das consequências da disfunção sexual feminina."

Exemplo 2

"Developing a reliable and valid measure of nausea/vomiting in this population will facilitate the measurement of these reactions in clinical trials. This study supported many of the psychometric characteristics of a short daily diary designed to measure nausea and vomiting. The Nausea/Vomiting Symptom Assessment (NVSA©) may be used to support study endpoints in clinical trials comparing the nausea and vomiting profile of novel Secondary hyperparathyroidism (SHPT) therapies." [72]

Tradução: "O desenvolvimento de uma medida confiável e válida de náusea/vômito nessa população facilitará a medição dessas reações em estudos clínicos. Este estudo respaldou muitas das características psicométricas de um diário curto projetado para

medir náuseas e vômitos. A *Nausea/Vomiting Symptom Assessment* (NVSA©) pode ser usada para apoiar os desfechos do estudo em ensaios clínicos que comparam o perfil de náusea e vômito de novas terapias para hiperparatireoidismo secundário (THPS).”

DC3 - Pontos fortes e limitações

Discuta os pontos fortes e as limitações de cada estudo. Por exemplo, se houve algum viés em potencial no estudo que poderia ter afetado os resultados.

Explicação: Um estudo é fortalecido quando emprega um método bem projetada e rigorosa. Isso inclui amostragem apropriada (técnicas), métodos de coleta de dados, análises estatísticas e métodos de interpretação dos resultados. Por outro lado, a seção de limitações identifica possíveis vieses significativos ou evidências de imprecisão em seu estudo que poderiam ter afetado seus resultados e conclusões. Por exemplo, (1) se você realizou um estudo sobre a validade do conteúdo de uma PROM e o número de adultos foi predominante com alto nível de escolaridade, você deve discutir que os resultados não devem ser generalizados para adultos com níveis mais baixos de escolaridade; (2) um estudo de confiabilidade requer medidas repetidas em um paciente estável, mas alguns dos pacientes incluídos podem ter mudado; ou (3) alguns dos participantes não preencheram os instrumentos de comparação usados para avaliar a validade ou a responsividade no mesmo dia. Recomendamos que você discuta os pontos fortes e as limitações de cada propriedade de medida descrita em seu artigo em termos de possíveis vieses que poderiam ter afetado seus resultados. A discussão transparente dos pontos fortes e das limitações aumenta a credibilidade de cada um dos estudos que podem afetar a aplicabilidade mais ampla de seus resultados.

Elementos essenciais

• Pontos fortes do estudo.

• Limitações do estudo.

Exemplo 1

"A strength of this study is the very large sample size, enabling us to perform the analyses for subgroups with and without chronic diseases and to investigate differential item functioning (DIF) for important sociodemographic and clinical characteristics. A limitation of our study is the representativeness of the Lifelines cohort, in which males, younger persons, and persons with an immigration background are underrepresented compared with the general Dutch population. Furthermore, in our sample, 62% reported not having a chronic condition, whereas according to registries in 2019, 43% of the Dutch population had no chronic condition [ref]. Thus, our sample was not representative for the Dutch population, and therefore, reported T-scores should not be interpreted as reference values for the Dutch population. Papers regarding reference values for the Dutch population on the domains included in the PROMIS-29 have recently been or will

soon be published ref]. Finally, formulating challenging hypotheses in which both the direction and the magnitude of the difference or relationship are included, is difficult. We based our hypotheses on findings of previous research, to show that PROMIS-29 functions in our population as expected." [23]

Tradução: "Um ponto forte deste estudo é o tamanho muito grande da amostra, o que nos permitiu realizar as análises para subgrupos com e sem doenças crônicas e investigar o *differential item functioning* (DIF) para características sociodemográficas e clínicas importantes. Uma limitação de nosso estudo é a representatividade da coorte *Lifelines*, na qual os homens, os jovens e as pessoas com histórico de imigração estão sub-representados em comparação com a população holandesa em geral. Além disso, em nossa amostra, 62% relataram não ter uma condição crônica, enquanto, de acordo com os registros de 2019, 43% da população holandesa não tinha nenhuma condição crônica [ref]. Assim, nossa amostra não foi representativa da população holandesa e, portanto, as pontuações T/*t-scores* relatadas não devem ser interpretadas como valores de referência para a população holandesa. Artigos sobre valores de referência para a população holandesa nos domínios incluídos no PROMIS-29 foram publicados recentemente ou serão publicados em breve [ref]. Por fim, é difícil formular hipóteses desafiadoras nas quais tanto a direção quanto a magnitude da diferença ou relação estejam incluídas. Baseamos nossas hipóteses nos resultados de pesquisas anteriores para mostrar que o PROMIS-29 funciona em nossa população como esperado."

Exemplo 2

"Although the numbers of patient participants in the focus groups were limited, the voice of patients was widely represented throughout the development process and further enhanced by the active involvement of our patients as research partners (PRPs) at all key stages (Supplementary Table S1, available at Rheumatology Advances in Practice online). Although all patient participants in stage 3 cognitive interviews were male, the contribution of the PRPs (4 female and 3 male) to the analysis ensured that a gendered view of the data was facilitated. A rigorous approach to all phases of the qualitative research is described, which involved patient, clinical and research experts participating in an iterative approach to item development and refinement. This process increases confidence that our multifaceted approach has minimized the risk that any patient-important outcomes have been omitted from the measurement framework. The involvement of health-care professionals in additional focus groups was essential to enhancing the clinical relevance of the model and is a further strength of the Warwick Axial Spondyloarthritis faTigue and Energy questionnaire (WASTEd)." [24]

Tradução: “Embora o número de pacientes participantes nos grupos focais tenha sido limitado, a opinião dos pacientes foi amplamente representada durante todo o processo de desenvolvimento e aprimorada pelo envolvimento ativo de nossos Pacientes como Parceiros de Pesquisa (PPPs) em todos os estágios principais (Tabela suplementar S1, disponível no *Rheumatology Advances in Practice on-line*). Embora todos os pacientes participantes das entrevistas cognitivas do estágio 3 fossem do sexo masculino, a contribuição dos PPPs (4 do sexo feminino e 3 do sexo masculino) para a análise garantiu que uma visão de gênero dos dados fosse facilitada. É descrita uma abordagem rigorosa para todas as fases da pesquisa qualitativa, que envolveu pacientes, especialistas clínicos e de pesquisa que participaram de uma abordagem iterativa para o desenvolvimento e o refinamento de itens. Esse processo aumenta a confiança de que nossa abordagem multifacetada minimizou o risco de que qualquer resultado importante para o paciente tenha sido omitido da estrutura de medição. O envolvimento de profissionais de saúde em grupos focais adicionais foi essencial para aumentar a relevância clínica do modelo e é mais um ponto forte do *Warwick Axial Spondyloarthritis faTigue and Energy questionnaire* (WASTEd).”

Exemplo 3

“Limitations included the post hoc secondary analysis of patients, all of whom had recurrent or metastatic disease and were subject to recall bias. This limitation was mitigated by cognitive interviews with patients actively undergoing radiation therapy (RT) or who had recently completed RT for head and neck squamous cell carcinoma (HNSCC). Sample sizes were limited for both the physician-directed survey and the cognitive interviews but were consistent with other published PRO measure development and content validation studies [ref], and went beyond the standard content validity sample size guidelines [ref].” [73]

Tradução: “As limitações incluíram a análise secundária post hoc de pacientes, todos com doença recorrente ou metastática e sujeitos a viés de memória. Essa limitação foi atenuada por entrevistas cognitivas com pacientes que estavam sendo submetidos ativamente à radioterapia (RT) ou que haviam concluído recentemente a RT para carcinoma de células escamosas de cabeça e pescoço (CCECE). Os tamanhos das amostras foram limitados tanto para a pesquisa dirigida pelo médico quanto para as entrevistas cognitivas, mas foram consistentes com outros estudos publicados de desenvolvimento de instrumentos de desfechos relatados pelo paciente e validação de conteúdo [ref], e foram além das diretrizes padrão de tamanho de amostra de validade de conteúdo [ref].”

DC4 - Generalização

Discuta a generalização dos resultados. Por exemplo, se os resultados poderiam ser generalizados para outras populações, considerando a amostra estudada.

Explicação: Generalização é a aplicabilidade de seus resultados a outras populações e circunstâncias. Diferentes populações ou ambientes podem ter características exclusivas que influenciam o desempenho de uma PROM. Recomendamos que você discuta a generalização dos resultados para outras populações, considerando a amostra estudada, o construto em estudo e a propriedade de medida em estudo. Por exemplo, se a PROM foi avaliada em uma população diferente daquela para a qual foi desenvolvida, você pode refletir sobre as diferenças ou semelhanças nos resultados (se conhecidas); ou algumas propriedades de medida (por exemplo, confiabilidade e validade de conteúdo) que dependem especificamente da população em que são aplicadas. Além disso, se a PROM mede um resultado mais genérico, os resultados podem ser mais fáceis de generalizar em comparação com um resultado relacionado a uma doença. A generalização pode ou não estar diretamente relacionada ao contexto de uso para o qual a PROM foi desenvolvida. Por exemplo, um instrumento de desfecho foi desenvolvido para triagem de pacientes, mas posteriormente é usado para avaliar pacientes. A discussão da generalização permite que os leitores considerem os fatores contextuais que podem afetar a aplicabilidade da PROM em diversas situações. Essas informações são valiosas para pesquisadores, clínicos e formuladores de políticas que buscam implementar a PROM em diferentes ambientes.

Elementos essenciais

• Generalização dos resultados (para outras populações em termos de doença e características demográficas, para outros ambientes, para outros modos de administração, etc.).

Exemplo 1

"The sample of this study was collected in a referral rehabilitation hospital, therefore, community samples or samples assisted in clinics may have particular characteristics. The Brazilian Portuguese version of the Functional Assessment Questionnaire (FAQ) showed adequate understanding and psychometric properties, but it should be investigated in other languages. Our study only involved patients with cerebral palsy (CP) and, therefore, extrapolations to other populations should be avoided." [74]

Tradução: “A amostra deste estudo foi coletada em um hospital de reabilitação de referência, portanto, amostras da comunidade ou amostras atendidas em clínicas podem ter características particulares. A versão em português brasileiro do *Functional Assessment Questionnaire* (FAQ) apresentou compreensão e propriedades psicométricas adequadas, mas deve ser investigada em outros idiomas. Nosso estudo envolveu apenas pacientes com paralisia cerebral (PC) e, portanto, devem ser evitadas extrapolações para outras populações.”

Exemplo 2

“Furthermore, since this study was conducted in a heterogeneous sample (i.e., diversity of diagnoses) of chronic hand orthotic users, wearing the three most commonly prescribed types of hand orthoses, we are confident that the results can be generalized to the population of chronic hand orthotic users at large.” [75]

Tradução: “Além disso, como esse estudo foi conduzido em uma amostra heterogênea (ou seja, diversidade de diagnósticos) de usuários crônicos de órteses manuais, usando os três tipos mais comumente prescritos de órteses manuais, estamos confiantes de que os resultados podem ser generalizados para a população de usuários crônicos de órteses manuais em geral.”

DC5 - Mudanças no instrumento

Discuta quais modificações são necessárias na PROM existente.

Explicação: Os resultados dos estudos sobre as propriedades de medida podem informar ajustes ou melhorias da PROM que está sendo estudada. Recomendamos que você discuta a necessidade de modificar a PROM existente e forneça recomendações específicas para melhorias. Por exemplo, se os resultados da validade do conteúdo indicarem que há itens faltando, recomendamos que você indique qual conteúdo está faltando na PROM; ou se itens específicos não foram compreensíveis, podem ser fornecidas sugestões para a reformulação dos itens. Seu estudo sobre a validade estrutural pode sugerir outra estrutura na PROM além da sugerida anteriormente, e você pode propor outro algoritmo de pontuação para a PROM. Cada melhoria em uma PROM leva a uma nova versão, que exige nova avaliação. Se for necessário propor muitos ajustes na PROM que está sendo estudada, recomendamos que você proponha enfaticamente a descontinuação do uso dessa versão da PROM (veja também o item DC2).

Elementos essenciais

• Discuta quais modificações são necessárias na PROM existente.

Exemplo 1

“end-users should note that the removal of items pertaining to pain, headaches, lifting, reading and sleep should not be taken to suggest that these domains are unimportant. However, for either conceptual or statistical reasons, they either do not fit with the construct of neck-related function, do not function well in their current form, or are statistically redundant and therefore do not add enough additional information to warrant retention. Each of these constructs have been deemed more or less important to the neck pain experience by previous authorship groups.” [76]

Tradução: “usuários finais devem observar que a remoção de itens relativos à dor, dores de cabeça, levantamento de peso, leitura e sono não deve ser interpretada como uma sugestão de que esses domínios não são importantes. No entanto, por razões conceituais ou estatísticas, eles não se encaixam no construto da função relacionada à cervical, não funcionam bem em sua forma atual ou são estatisticamente redundantes e, portanto, não acrescentam informações adicionais suficientes para justificar a retenção. Cada um desses construtos foi considerado mais ou menos importante para a experiência da dor no pescoço por grupos de autores anteriores.”

Exemplo 2

"The results of our study, and of previous studies indicate that this item bank in its present form, has limitations, and it is questionable whether it can be used without restrictions in short forms and in computerized adaptive testing (CAT) applications. (...) The results of our study, together with the results of other studies reporting on psychometric properties could be used to improve the item bank, for example, by removing items with poor scalability. We recommend more validation studies of translated versions in other languages and in populations with other types of pain, especially to study dimensionality and monotonicity." [77]

Tradução: "Os resultados de nosso estudo e de estudos anteriores indicam que esse banco de itens, em sua forma atual, tem limitações, e é questionável se ele pode ser usado sem restrições em formulários curtos e em aplicações de testes adaptativos computadorizados (TAC). (...) Os resultados de nosso estudo, juntamente com os resultados de outros estudos que relatam propriedades psicométricas, poderiam ser usados para melhorar o banco de itens, por exemplo, removendo itens com baixa escalabilidade. Recomendamos mais estudos de validação de versões traduzidas em outros idiomas e em populações com outros tipos de dor, especialmente para estudar a dimensionalidade e a monotonicidade."

DC6 - Pesquisas futuras

Descreva novas perguntas ou hipóteses de pesquisa geradas a partir desses resultados e forneça/descreva a pesquisa necessária para responder a essas perguntas.

Explicação: Se uma PROM parecer promissora para uso (ou seja, ainda não há evidências de propriedades de medida insuficientes e não há sugestões para melhorar a PROM em sua forma atual), as recomendações para pesquisas futuras permitem que seus leitores reconheçam e abordem as deficiências ou a falta de conhecimento da qualidade da PROM que está sendo estudada. Ao destacar as lacunas no conhecimento ou as áreas em que são necessárias mais investigações, a comunidade de pesquisa é informada para aumentar as evidências sobre a qualidade da PROM. Recomendamos que você indique quais novas perguntas de pesquisa ou hipóteses geradas a partir dessas descobertas surgiram ou ainda não foram abordadas, seguidas de sugestões para pesquisas futuras sobre a(s) propriedade(s) de medida específica(s) da PROM. Por exemplo, inclua estudos longitudinais para avaliar a responsividade da PROM. Isso é particularmente importante para medir as mudanças no estado de saúde ao longo do tempo. As recomendações para pesquisas futuras também podem sugerir a exploração do uso da PROM em subpopulações ou contextos culturais específicos. Por exemplo, em vários ambientes, como atenção primária, clínicas especializadas ou ambientes de pesquisa. Em resumo, as sugestões para pesquisas futuras fornecem um roteiro para a criação de evidências sobre a qualidade da PROM.

Elementos essenciais

- Quais novas questões surgiram.
- Sugestões para pesquisas futuras sobre propriedades de medida específicas da PROM ou em vários ambientes.

Exemplo 1

"The evaluation of responsiveness, the ability of an instrument to detect changes over time in the construct to be measured, was beyond our scope. Therefore, we suggest that future studies assess Female Genital Self-image Scale (FGSIS) responsiveness in the Brazilian population." [42]

Tradução: "A avaliação da responsividade, a capacidade de um instrumento de detectar mudanças ao longo do tempo no construto a ser medido, estava além de nosso escopo. Portanto, sugerimos que estudos futuros avaliem a responsividade do *Female Genital Self-image Scale* (FGSIS) na população brasileira."

Exemplo 2

"The results of our study, and of previous studies indicate that this item bank in its present form, has limitations, and it is questionable whether it can be used without restrictions in short forms and in computer adaptive testing (CAT) applications. Further item response theory (IRT) analyses are recommended in a Dutch general population and on a combined set of clinical and general population data to determine the optimal IRT item parameters for use of CAT in The Netherlands and Flanders (Dutch or Flemish speaking part of Belgium). The results of our study, together with the results of other studies reporting on psychometric properties could be used to improve the item bank, for example, by removing items with poor scalability. We recommend more validation studies of translated versions in other languages and in populations with other types of pain, especially to study dimensionality and monotonicity." [77]

Tradução: "Os resultados de nosso estudo e de estudos anteriores indicam que esse banco de itens, em sua forma atual, tem limitações e é questionável se ele pode ser usado sem restrições em formulários curtos e em aplicações de Testes Adaptativos Computadorizados (TAC). Recomenda-se a realização de outras análises da teoria da resposta ao item (TRI) em uma população geral holandesa e em um conjunto combinado de dados clínicos e da população geral para determinar os parâmetros ideais de itens da TRI para uso do TAC na Holanda e em Flandres (parte da Bélgica que fala holandês ou flamengo). Os resultados do nosso estudo, juntamente com os resultados de outros estudos que relatam propriedades psicométricas, podem ser usados para melhorar o banco de itens, por exemplo, removendo itens com baixa escalabilidade. Recomendamos mais estudos de validação de versões traduzidas em outros idiomas e em populações com outros tipos de dor, especialmente para estudar a dimensionalidade e a monotonicidade."

DC7 - Conclusões

Forneça as conclusões gerais sobre o uso da PROM.

Explicação: Fornecer as conclusões gerais do seu estudo oferece uma oportunidade para os leitores interpretarem os resultados no contexto dos objetivos do estudo, das hipóteses e da literatura existente. Recomendamos que você forneça suas conclusões gerais sobre se as propriedades de medida estudadas foram consideradas suficientes ou insuficientes, e conclua se a PROM pode ser usada com segurança. Conclusões claras podem melhorar a padronização da seleção e do uso da PROM. No corpo da seção Discussão, recomendamos que você explique sua conclusão (veja o item DC2) e que repita sua conclusão sobre a aplicabilidade da PROM em estudo bem no final do artigo.

Elementos essenciais

- Conclusões gerais sobre as propriedades de medida estudadas da PROM.
- Conclusão geral sobre o uso da PROM.

Exemplo 1

"The Dutch-Flemish PROMIS Pain Behavior item bank showed that all items fitted the item response theory (IRT) model, and our results supported cross-cultural validity. However, the assumptions of unidimensionality and monotonicity were not met. Bifactor analysis indicated a low risk of biased scores when assuming unidimensionality, although the fit of the bifactor model was still suboptimal. Our study showed limitations of the Dutch-Flemish PROMIS Pain Behavior item bank when used in a primary care population with musculoskeletal complaints." [77]

Tradução: "O banco de itens do PROMIS *Pain Behavior* holandês-flamengo mostrou que todos os itens se encaixavam no modelo da teoria da resposta ao item (TRI), e nossos resultados apoiaram a validade transcultural. No entanto, as suposições de unidimensionalidade e monotonicidade não foram atendidas. A análise de bifatores indicou um baixo risco de pontuações tendenciosas quando se pressupõe a unidimensionalidade, embora o ajuste do modelo de bifatores ainda estivesse abaixo do ideal. Nosso estudo mostrou limitações do banco de itens do PROMIS *Pain Behavior* holandês-flamengo quando usado em uma população de cuidados primários com queixas musculoesqueléticas."

Exemplo 2

"Comparisons of these results with the validation study of the English version are therefore limited to the examination of the convergent validity, which showed similarly high correlations with comparative measurements such as the short form of the Stroke Impact Scale 2.0 (SF-SIS) 3.0, the EQ-5D-5L and the National Institutes of Health Stroke Scale (NIHSS). The analysis of structural validity did not confirm the suggested unidimensionality of the scale and found evidence for an underlying two-factor solution with a physical and cognitive domain. Further research is needed to improve and clarify the underlying factorial structure of the SF-SIS. The discriminative validity had also not been investigated so far, but showed sufficient ability to distinguish between mildly and severely affected stroke survivors. Adequate test-retest reliability was found in stable subjects after one week." [78]

Tradução: "As comparações desses resultados com o estudo de validação da versão em inglês são, portanto, limitadas ao exame da validade convergente, que mostrou correlações igualmente altas com medidas comparativas, como a forma curta da *Stroke Impact Scale* 2.0 (SF-SIS) 3.0, a EQ-5D-5L e a *National Institutes of Health Stroke Scale* (NIHSS). A análise da validade estrutural não confirmou a unidimensionalidade sugerida da escala e encontrou evidências de uma solução subjacente de dois fatores com um domínio físico e cognitivo. São necessárias mais pesquisas para melhorar e esclarecer a estrutura fatorial subjacente da SF-SIS. A validade discriminativa também não havia sido investigada até o momento, mas mostrou capacidade suficiente para distinguir entre sobreviventes de acidente vascular cerebral com comprometimento leve e grave. Foi encontrada uma confiabilidade adequada de teste-reteste em indivíduos estáveis após uma semana."

Seção do relato: Outras informações

O1 - Conflitos de interesse

Declare qualquer conflito de interesse que você possa ter em relação à PROM. Isso pode incluir qualquer envolvimento no desenvolvimento da PROM ou qualquer financiamento ou lucro comercial.

Explicação: Um conflito de interesse em um estudo refere-se a uma situação em que os interesses profissionais ou pessoais dos indivíduos envolvidos na pesquisa podem comprometer a integridade, a objetividade ou a imparcialidade do estudo. Os conflitos de interesse podem surgir de várias formas e envolver relacionamentos financeiros, pessoais ou profissionais que possam influenciar o projeto, a condução, a análise ou o relatório dos resultados da pesquisa. Recomendamos que você revele quaisquer conflitos de interesse relevantes para a PROM que está sendo estudada. Por exemplo, se você recebeu financiamento para realizar o estudo, recomendamos que descreva a parte financiadora; se você for o desenvolvedor da PROM, recomendamos que declare isso; se você obtiver lucro de outras partes quando elas usarem a PROM que você desenvolveu, isso deve ser declarado. Muitas revistas têm diretrizes e políticas para gerenciar e atenuar conflitos de interesse.

Elementos essenciais

• Conflitos de interesse relacionados à PROM.

Exemplo 1

"Stein Arne Rimehaug serves as a national contact person for PROMIS in Norway, Aaron J Kaat and Stein Arne Rimehaug are both members of the 2020 and 2021 Scientific Advisory Committee for the PROMIS Health Organization International. J E Nordvik, M Klokkerud and H S Robinson have no conflicts of interest to disclose." [79]

Tradução: "Stein Arne Rimehaug atua como pessoa de contato nacional do PROMIS na Noruega, Aaron J Kaat e Stein Arne Rimehaug são membros do Comitê Consultivo Científico de 2020 e 2021 da PROMIS *Health Organization International*. J E Nordvik, M Klokkerud e H S Robinson não têm conflitos de interesse a divulgar."

Exemplo 2

"The authors declare that they have no competing interest." [30]

Tradução: "Os autores declaram que não têm nenhum interesse concorrente."

Exemplo 3

"(…) is one of the developers of the Arm Function in Multiple Sclerosis Questionnaire (AMSQ)." [80]

Tradução: "(...) é um dos desenvolvedores do *Arm Function in Multiple Sclerosis Questionnaire* (AMSQ)."

Recomendações específicas de relatos para estudos para cada propriedade de medida

Validade de conteúdo

A validade de conteúdo é o grau em que o conteúdo de um instrumento é um reflexo adequado do construto a ser medido [1]. Três aspectos da validade de conteúdo são distinguidos: (1) relevância (todos os itens em uma PROM devem ser relevantes para o construto de interesse, para a população-alvo e para o contexto de uso), (2) abrangência (nenhum aspecto-chave do construto deve estar faltando) e (3) compreensão (os itens devem ser entendidos pelos pacientes como pretendido). Todos esses aspectos podem ser avaliados no mesmo estudo usando os mesmos métodos.

Validade de conteúdo: Métodos

CV1 - Relevância

Especifique se, e como, os pacientes e/ou profissionais foram questionados se as instruções, cada um dos itens, as opções de resposta e o período de recordação eram relevantes para o(s) construto(s), a população e o contexto de uso.

Explicação: Para garantir que a PROM capture adequadamente o construto pretendido para a população-alvo e o contexto de uso, recomendamos especificar se e como os pacientes e/ou profissionais (por exemplo, clínicos, pesquisadores) foram questionados se as instruções, cada um dos itens, as opções de resposta e o período de recordação eram relevantes para o construto, a população do estudo e o contexto de uso. Cada um desses aspectos deve ser avaliado separadamente. O período de recordação pode ser incluído nas instruções ou nos itens e, portanto, pode ser avaliado simultaneamente. Os pacientes e/ou profissionais avaliam a relevância da PROM com base em sua experiência ou conhecimento do construto. A relevância da PROM pode ser avaliada por meio de um estudo qualitativo e/ou quantitativo (levantamento/*survey*). Estudos qualitativos que envolvam comunicação direta com pacientes e/ou profissionais, como grupos focais ou entrevistas individuais, são recomendados para captar adequadamente suas perspectivas sobre questões de importância relativas ao foco da PROM [81]. Quando forem realizados grupos focais ou entrevistas individuais, recomendamos que você informe se utilizou uma abordagem estruturada, semiestruturada ou não estruturada

e descreva o tópico ou o guia de entrevista utilizado (o tópico completo ou o guia de entrevista geralmente é publicado como um documento suplementar). O objetivo desse guia é estabelecer procedimentos, manter a consistência na coleta de dados, evitar influência indevida do moderador/entrevistador e manter a conversa no tópico. Também é recomendável informar se foram feitas perguntas abertas para permitir o relato espontâneo e garantir uma coleta de dados imparcial. Se você utilizou entrevistas ou grupos focais, recomendamos que informe se as gravou com equipamento de áudio ou vídeo para capturar totalmente o contexto e o conteúdo de cada sessão e se todas as entrevistas ou grupos focais foram transcritos literalmente. Recomendamos também que informe se foram envolvidos moderadores/ entrevistadores qualificados para entrevistar os pacientes. Para relatar a análise de dados em um estudo qualitativo, informe o método escolhido (por exemplo, análise de conteúdo, análise dedutiva, análise de estrutura, teoria fundamentada) juntamente com o tipo de codificação usada (por exemplo, aberta, axial, seletiva) e se pelo menos duas pessoas codificaram independentemente parte dos dados. Se duas pessoas codificaram os dados, recomendamos que você informe a porcentagem de códigos que foram codificados repetidamente. Recomendamos também que você informe se, e como, os dados qualitativos foram coletados até que nenhum novo conhecimento relevante fosse obtido de novos pacientes. Em estudos quantitativos (*survey*), os participantes são solicitados a classificar a relevância de cada aspecto da PROM (por exemplo, as instruções, cada um dos itens, as opções de resposta e o período de recordação) para o construto que está sendo medido, a população do estudo e o contexto de uso. Os participantes podem ser solicitados a fornecer classificações em uma escala Likert e/ou perguntas abertas para obter feedback qualitativo sobre a relevância dos aspectos da PROM.

Elementos essenciais

• Se os pacientes e/ou profissionais foram questionados se as instruções, cada um dos itens, as opções de resposta e o período de recordação eram relevantes para o construto, a população e o contexto de uso.

• Método usado (qualitativo e/ou quantitativo).

• Em um estudo qualitativo, que tipo de método (grupos focais ou entrevistas individuais) e abordagem (estruturada, semiestruturada ou não estruturada) foram usados.

• Em um estudo qualitativo, descrição de um tópico ou guia de entrevista; se foram usadas perguntas abertas; se foram usados moderadores/entrevistadores qualificados; descrição do método de análise de dados (por exemplo, análise de conteúdo, análise dedutiva, análise de estrutura, teoria fundamentada); tipo de codificação usada (por exemplo, codificação aberta, codificação axial, codificação seletiva); e evidências de que nenhum novo conhecimento relevante foi obtido de novos pacientes.

Exemplo 1

"(...) face-to-face cognitive interviews were held during December 2019 and January 2020. Interviews were held at the De Hoogstraat Rehabilitation center or in the respondents' homes, as they wished. (...) The preliminary 25-item version of the self-regulation assessment (SeRA) was used in the first series of cognitive interviews. Respondents were asked to think aloud and explain all of their thoughts when answering the items. Probing was used when respondents did not spontaneously express their thoughts on the items. Reflective questions on the SeRA in general were asked directly after the cognitive interview, e.g., whether the items fully reflect the concept, what their thoughts were concerning the items in general, reflection on the instructions and the response options, and if they missed items. Interviews were held in the Dutch language and were audiotaped. Interviews lasted between 40 and 90 min. Prior to the interviews, a few test and feedback interviews were conducted to ensure qualification of the interviewer. All eight interviews were held by the same interviewer (KH), and during the first three interviews, her supervisor (TM) was the second interviewer.
All audio recordings were transcribed verbatim and anonymized. Data analyses were performed by using MaxQDA software (Verbi Gmbh MaxQDA 2018.2). Thematic analysis and open coding were used for content analysis. Cognitive interviews were initially analyzed by the interviewer, and next by the first author of this manuscript. The coding system of the analyses was based on three categories in which 10 criteria for good content validity are divided: (a) comprehensibility, (b) comprehensiveness, and (c) relevance [ref]. Comprehensibility covers four criteria: instructions understood as intended, items and response options understood as intended, items worded appropriately, and match between questions and response options. Comprehensiveness refers to the criterion whether no key concepts are missing. Finally, relevance contains five criteria: all items relevant for the construct, for the target population, and for the context of use; are response options appropriate; and if the recall period is appropriate [ref]. (...) All authors involved in the content analyses were, or were supervised by, experienced qualitative researchers and were from different backgrounds such as psychology, health sciences, and rehabilitation medicine. (...) To ensure the data credibility, each transcript was independently analyzed by at least two researchers." [82]

Tradução: "(...) entrevistas cognitivas presenciais foram realizadas em dezembro de 2019 e janeiro de 2020. As entrevistas foram realizadas no Centro de Reabilitação De Hoogstraat ou nas casas dos entrevistados, conforme a preferência deles. (...) A versão preliminar de 25 itens do *self-regulation assessment* (SeRA) foi usada na primeira série de entrevistas cognitivas. Os entrevistados foram solicitados a pensar em voz alta e explicar todos os seus pensamentos ao responder aos itens. As perguntas de aprofundamento foram usadas quando os entrevistados não expressaram espontaneamente seus pensamentos sobre os itens. Perguntas reflexivas sobre o SeRA em geral foram feitas diretamente após a entrevista cognitiva, por exemplo, se os itens refletiam totalmente o conceito, o que eles pensavam a respeito dos itens em geral, a reflexão sobre as instruções e as opções de resposta e se eles haviam perdido itens. As entrevistas foram realizadas no idioma holandês e gravadas em áudio. As entrevistas duraram entre 40 e 90 minutos. Antes das entrevistas, foram realizadas algumas entrevistas de teste e feedback para garantir a qualificação do entrevistador. Todas as oito entrevistas foram realizadas pelo mesmo entrevistador (KH) e, durante as três primeiras entrevistas, seu supervisor (TM) foi o segundo entrevistador.
Todas as gravações de áudio foram transcritas na íntegra e tornadas anônimas. As análises de dados foram realizadas com o software MaxQDA (Verbi Gmbh MaxQDA 2018.2). A análise temática e a codificação aberta foram usadas para a análise de conteúdo. As entrevistas cognitivas foram inicialmente analisadas pelo entrevistador e, em seguida, pelo primeiro autor deste artigo. O sistema de codificação das análises foi baseado em três categorias nas quais são divididos 10 critérios para uma boa validade de conteúdo: (a) compreensão, (b) abrangência e (c) relevância [ref]. A compreensão abrange quatro critérios: instruções entendidas como pretendidas, itens e opções de resposta entendidos como pretendidos, itens redigidos adequadamente e correspondência entre perguntas e opções de resposta. A abrangência refere-se ao critério de não haver conceitos-chave faltando. Finalmente, a relevância contém cinco critérios: todos os itens são relevantes para o construto, para a população-alvo e para o contexto de uso; as opções de resposta são apropriadas; e se o período de recordação é apropriado [ref]. (...) Todos os autores envolvidos nas análises de conteúdo eram, ou foram supervisionados por pesquisadores com experiência em estudos qualitativos e tinham diferentes formações, como psicologia, ciências da saúde e medicina de reabilitação. (...) Para garantir a credibilidade dos dados, cada transcrição foi analisada de forma independente por pelo menos dois pesquisadores."

CV2 - Abrangência

Especifique se, e como, os pacientes e/ou profissionais foram questionados se todos os conceitos-chave estão incluídos na PROM.

Explicação: Para determinar se a PROM capta adequadamente a amplitude e a profundidade do construto-alvo, recomendamos que você especifique se, e como, os pacientes e/ou profissionais foram questionados se todos os conceitos-chave estão incluídos na PROM. A abrangência deve ser avaliada para todas as subescalas. A abrangência da PROM pode ser avaliada por meio de um estudo qualitativo e/ou quantitativo (levantamento/*survey*). Todos os métodos recomendados para avaliar a abrangência são semelhantes àqueles para avaliar a relevância e estão descritos anteriormente (veja o item CV1).

Elementos essenciais

• Se, e como, foi perguntado aos pacientes e/ou profissionais se todos os conceitos-chave estão incluídos na PROM.
• Método utilizado (qualitativo e/ou quantitativo).
• Em um estudo qualitativo, que tipo de método (por exemplo, grupos focais, entrevistas individuais, método Delphi) e abordagem (estruturada, semiestruturada ou não estruturada) foram usados.
• Em um estudo qualitativo, descrição de um tópico ou guia de entrevista; se foram feitas perguntas abertas; se foram usados moderadores/entrevistadores qualificados; o método de análise de dados (por exemplo, análise de conteúdo, análise dedutiva, análise de estrutura, teoria fundamentada); tipo de codificação usada (por exemplo, codificação aberta, codificação axial, codificação seletiva); evidências de que nenhum novo conhecimento relevante foi obtido de novos pacientes.

Exemplo 1

"The study objective was to assess the content validity of the Cough and Sputum Assessment Questionnaire (CASA-Q) cough domains and the Shortness-of-Breath Questionnaire (UCSD-SOBQ) for use in patients with Idiopathic Pulmonary Fibrosis (IPF). (…) The cough domains of the CASA-Q include 11-items (...). The 9 questions of the CASA-Q sputum domains were not included for this assessment (...). The study consisted of one-on-one interviews. Participants completed the CASA-Q cough domains and the UCSD-SOBQ, followed by debriefing questions using a semi-structured interview guide. The interview guide contained questions about the participant's understanding of the instructions for each instrument, the recall period, the intended meaning and

relevance of the items and response options, and general questions about the overall instrument and missing concepts." [83]

Tradução: "O objetivo do estudo foi avaliar a validade do conteúdo dos domínios de tosse do *Cough and Sputum Assessment Questionnaire* (CASA-Q) e do *Shortness-of-Breath Questionnaire* (UCSD-SOBQ) para uso em pacientes com Fibrose Pulmonar Idiopática (FPI). (...) Os domínios de tosse do CASA-Q incluem 11 itens (...). As 9 perguntas dos domínios de escarro do CASA-Q não foram incluídas nessa avaliação (...). O estudo consistiu em entrevistas individuais. Os participantes preencheram os domínios de tosse do CASA-Q e o UCSD-SOBQ, seguidos de perguntas de esclarecimento usando um guia de entrevista semiestruturado. O guia de entrevista continha perguntas sobre a compreensão do participante das instruções de cada instrumento, o período de recordação, o significado pretendido e a relevância dos itens e das opções de resposta, além de perguntas gerais sobre o instrumento geral e os conceitos ausentes."

Exemplo 2

"Cognitive debriefing (~40 min) involved the participants completing each Vitiligo Patient Priority Outcome (ViPPO) measure using a 'think aloud' technique in which they spoke their thoughts aloud while reading the questions and selecting an answer. Participants were asked direct and specific questions to establish the content validity of the ViPPO measures (Table 1)."

Table 1. Example cognitive debriefing questions

Concept	Example interview questions
Understanding of item wording	What does the question mean to you?<br>How would you say this in your own words?
Interpretation of the measurement concept	What does [self-conscious] mean to you?
Relevance of the measurement concept	Have you ever experienced this?<br>Can you give me an example?
Item redundancy	Do you think feeling [embarrassed] and [self-conscious] are the same or different?
Relationship between items	Is it important to ask about feeling [sad] and feeling [depressed] separately? Or could be questions be combined?
Comprehensiveness	Was there anything missing from the questionnaire?
Suitability of the different response scales/ options	What are your thoughts on the response scale?<br>Which scale do you prefer?
Relevance of the recall period to the measurement concepts	How easy or difficult was it to answer thinking about the past 7 days?<br>Would a different timeframe be easier to think about?

[84]

Tradução: "O interrogatório (~40 min) envolveu os participantes que completaram cada medida *Vitiligo Patient Priority Outcome* (ViPPO) usando uma técnica de 'pensar em voz alta' na qual eles falaram seus pensamentos em voz alta enquanto liam as perguntas e selecionavam uma resposta. Foram feitas perguntas diretas e específicas aos participantes para estabelecer a validade do conteúdo das medidas da ViPPO (Tabela 1)."

Tabela 1. Exemplo de perguntas de interrogatório

Conceito	Exemplos de perguntas de entrevista
Compreensão da redação do item	O que a pergunta significa para você?<br>Como você diria isso com suas próprias palavras?
Interpretação do conceito de medição	O que [autoconsciente] significa para você?
Relevância do conceito de medição	Você já teve essa experiência?<br>Pode me dar um exemplo?
Redundância de itens	Você acha que sentir-se [constrangido] e [autoconsciente] são a mesma coisa ou são diferentes?
Relação entre os itens	É importante perguntar sobre sentir-se [triste] e sentir-se [deprimido] separadamente? Ou essas perguntas poderiam ser combinadas?
Abrangência	Faltou alguma coisa no questionário?
Adequação das diferentes escalas/ opções de resposta	O que você acha da escala de resposta?<br>Qual escala você prefere?
Relevância do período de recordação para os conceitos de medição	Quão fácil ou difícil foi responder pensando nos últimos 7 dias?<br>Seria mais fácil pensar em um período de tempo diferente?

CV3 - Compreensão

Especifique se, e como, os pacientes e/ou profissionais avaliaram a compreensão das instruções, dos itens, das opções de resposta e do período de recordação da PROM.

Explicação: Se as instruções, os itens, as opções de resposta e/ou o período de recordação da PROM não forem claros, poderão ser coletadas informações incorretas ou os pacientes poderão ficar frustrados por não entenderem como preencher a PROM. A avaliação da compreensão de uma PROM garante que a linguagem, o texto e o *layout* da PROM sejam claros e acessíveis aos respondentes pretendidos. Recomendamos que você especifique se, e como, a compreensão das instruções, dos itens, das opções de resposta e do período de recordação da PROM foi avaliada pelos pacientes e/ou profissionais. Para a avaliação da compreensão, você pode usar métodos amplamente reconhecidos (por exemplo, método pensar em voz alta (do inglês, *Think aloud*), entrevista-teste em três etapas, interrogatório (do inglês, *debriefing*), sondagem ou outras formas de entrevista cognitiva). A avaliação da compreensão pode fazer parte de um processo de desenvolvimento de uma PROM (também chamado de fase de teste-piloto da PROM), como um estudo de validade de conteúdo ou como parte de um processo de tradução. Em essência, os métodos para testar a compreensão são semelhantes nessas diferentes fases. Recomendamos que informe a fase em que a compreensão está sendo testada (fase de desenvolvimento, fase de validade do conteúdo ou fase de tradução). Recomendamos que você descreva detalhes sobre o uso de um tópico ou guia de entrevista, gravação e transcrição, moderadores ou entrevistadores, métodos para analisar os dados e o tipo de código usado (incluindo as pessoas envolvidas), semelhante ao descrito para avaliar a relevância (veja o item CV1).

Elementos essenciais

• Se, e como, os pacientes e/ou profissionais foram questionados sobre a compreensão das instruções, cada um dos itens, as opções de resposta e o período de recordação para o construto, a população e o contexto de uso; fase em que a compreensão está sendo testada (ou seja, fase de desenvolvimento, fase de validade do conteúdo ou fase de tradução).
• Método usado (qualitativo e/ou quantitativo).
• Em um estudo qualitativo, que tipo de método e abordagem (estruturado, semiestruturado ou não estruturado) foram usados.
• Em um estudo qualitativo, descrição de um tópico ou guia de entrevista; se foram feitas perguntas abertas; se foram usados moderadores/entrevistadores qualificados; o método de análise de dados (por exemplo, análise de conteúdo, análise dedutiva, análise de estrutura,

teoria fundamentada); tipo de codificação usado (por exemplo, codificação aberta, codificação axial, codificação seletiva); evidências de que nenhum novo conhecimento relevante foi obtido de novos pacientes.

Exemplo 1

"Interviews took place between November 2020 and March 2021, during the COVID-19 pandemic, and thus were conducted remotely. Interviews were conducted by one of six trained qualitative interviewers (...) All interviews were audio-recorded and transcribed verbatim. Transcripts were analysed using ATLAS.ti v7.5. Each transcript was coded by one analyst, with codes and quotations reviewed by the project lead at regular intervals. The project team revisited the verbatim quotes when summarizing the findings. (…) As recommended in the Best Practice Guidelines for Establishing Content Validity of PRO [patient-reported outcome] Instruments [ref] and FDA PFDD Draft Guidance 2 [ref], interview transcripts were grouped into four equal sets in chronological order, and the elicited concepts were compared between sets. (...) Data obtained via cognitive debriefing were subject to framework coding [ref], whereby a predefined code list was applied to identify the relevance and appropriateness of item wording, response options and the recall period." [84]

Tradução: "As entrevistas ocorreram entre novembro de 2020 e março de 2021, durante a pandemia da COVID-19 e, portanto, foram realizadas remotamente. As entrevistas foram conduzidas por um dos seis entrevistadores qualitativos treinados (...) Todas as entrevistas foram gravadas em áudio e transcritas literalmente. As transcrições foram analisadas usando o ATLAS.ti v7.5. Cada transcrição foi codificada por um analista, com códigos e citações revisados pelo líder do projeto em intervalos regulares. A equipe do projeto revisou as citações literais ao resumir os resultados. (...) Conforme recomendado nas *Best Practice Guidelines for Establishing Content Validity of PRO [patient-reported outcome] Instruments* [ref] e no FDA PFDD *Draft Guidance* 2 [ref], as transcrições das entrevistas foram agrupadas em quatro conjuntos iguais em ordem cronológica, e os conceitos obtidos foram comparados entre os conjuntos. (...) Os dados obtidos por meio do interrogatório foram submetidos à codificação de estrutura [ref], por meio da qual uma lista de códigos predefinida foi aplicada para identificar a relevância e a adequação do texto do item, as opções de resposta e o período de recordação."

Validade de conteúdo: Resultados

CV4 - Relevância

Especifique se as instruções, todos os itens, as opções de resposta e o período de recordação foram considerados relevantes pelos pacientes e/ou profissionais, para o construto, para a população e para o contexto de uso.

Explicação: Recomendamos que você especifique se as instruções, os itens, as opções de resposta e o período de recordação foram considerados relevantes para o construto, a população e o contexto de uso pelos pacientes e profissionais. Se os pacientes e/ou profissionais identificaram questões importantes (por exemplo, alguns itens não são relevantes para a população ou o período de recordação não é relevante para o construto), é possível informar o número (porcentagem) de itens que foram considerados relevantes (e não relevantes) e indicar quais itens (ou aspectos da PROM) foram considerados não relevantes. Se alguns itens forem relevantes apenas para um pequeno número de pacientes, mas forem necessários para captar toda a gama de experiências dos pacientes, recomendamos que você apresente uma justificativa para a inclusão deles. Se a sua conclusão for que a validade do conteúdo é insuficiente em termos de relevância e que a PROM precisa ser aprimorada, recomendamos que você dê sugestões de aprimoramento com base nos seus resultados (veja o item DC5).

Elementos essenciais

• Se os pacientes e/ou profissionais consideraram as instruções, todos os itens, as opções de resposta e o período de recordação relevantes para o construto, a população e o contexto de uso de interesse.

• Número e/ou porcentagem de pacientes e/ou profissionais que não consideraram as instruções, os itens, as opções de resposta e o período de recordação relevantes.

Exemplo geral

"A relevância das instruções, dos itens, das opções de resposta e do período de recordação do [nome da PROM] foi avaliada por x profissionais e y pacientes. Todos os profissionais consideraram as instruções, os itens, as opções de resposta e o período de recordação do [nome da PROM] relevantes para avaliar [construto] em [população e contexto de uso de interesse]. A maioria dos pacientes (n = z, z%) considerou o item A irrelevante para a avaliação de [construto] em [população e contexto de uso de

interesse]. As instruções, os outros itens, as opções de resposta e o período de recordação foram considerados relevantes para todos os pacientes."

Exemplo 1

"Eight respondents were included. Respondents underscored the relevance of the items for a rehabilitation population. They particularly emphasized the items on gaining insights and understanding, and items on determination of own boundaries or possibilities. The respondents marked them as very relevant for medical rehabilitation treatment, and also for the construct of self-regulation. Comments from two respondents were: (...). Respondents also expressed that all items were relevant. An example quote is: (...). Also, items were marked as relevant for the rehabilitation population. Two respondents commented as follows: (...). Four items were marked as overlapping with other items, because they were similar to other items, or due to their wording. Concerning the response options, a few remarks were made that these were not appropriate for each item. Two respondents indicated that answering the questions is not a matter of indicating agree or not agree and missed the option to clarify their answers. Finally, respondents seemed not to have any difficulties regarding the recall period. None of the respondents had problems to remember anything needed to answer the items." [82]

Tradução: "Oito entrevistados foram incluídos. Os entrevistados ressaltaram a relevância dos itens para uma população em reabilitação. Eles enfatizaram especialmente os itens sobre a obtenção de percepções e compreensão, e os itens sobre a determinação dos próprios limites ou possibilidades. Os respondentes os consideraram muito relevantes para o tratamento de reabilitação médica e também para o construto de autorregulação. Os comentários de dois entrevistados foram os seguintes (...). Os entrevistados também expressaram que todos os itens eram relevantes. Um exemplo de citação é: (...). Além disso, os itens foram marcados como relevantes para a população de reabilitação. Dois entrevistados comentaram o seguinte: (...). Quatro itens foram marcados como sobrepostos a outros itens, porque eram semelhantes a outros itens ou devido à sua redação. Com relação às opções de resposta, foram feitos alguns comentários de que elas não eram apropriadas para cada item. Dois respondentes indicaram que responder às perguntas não é uma questão de concordar ou não concordar e perderam a opção de esclarecer suas respostas. Por fim, os respondentes parecem não ter tido nenhuma dificuldade com relação ao período de recordação. Nenhum dos entrevistados teve problemas para se lembrar de qualquer coisa necessária para responder aos itens."

CV5 - Abrangência

Especifique se os pacientes e/ou profissionais consideraram que todos os conceitos-chave foram incluídos na PROM.

Explicação: Recomendamos que você especifique se os pacientes e/ou profissionais consideraram que todos os conceitos-chave foram incluídos na PROM. Você pode relatar os resultados da abrangência em termos de quais aspectos estavam faltando e quantos pacientes e/ou profissionais consideraram que o aspecto estava faltando. A porcentagem de pacientes/profissionais que consideraram a PROM abrangente também pode ser indicada. Os pacientes podem ter relatado a falta de conteúdo que não está relacionada ao construto pretendido ou ao contexto de interesse da PROM. Você poderia informar esse conteúdo e explicar por que ele não está incluído na PROM. Dessa forma, você reconhece a contribuição dos pacientes, mas explica que ela está além do escopo da PROM que está sendo estudado.

Elementos essenciais

• Se os pacientes e/ou profissionais consideraram que todos os conceitos-chave foram incluídos na PROM.

• Especifique quais conceitos foram considerados ausentes.

Exemplo 1

"[Participants in total: Providers (n = 10) and Family members (n = 9)]. Families suggested adding questions about medication choices (n = 1), feeling comfortable with the provider (n = 1), the ability to stop or refuse treatment (n = 1), addressing crises in a timely fashion (n = 3), role of family involvement (n = 5), and locating services (n = 1). Providers suggested adding questions about accessible information (n = 2), overall service effectiveness (n = 1), evaluating previous experiences (not current experiences, n = 1), the role of family involvement (n = 3), insurance (n = 1), provider preferences (n = 2), and other types of therapeutic approaches (e.g., outdoor therapy, n = 1)." [85]

Tradução: "[Participantes no total: Provedores (n = 10) e familiares (n = 9)]. As famílias sugeriram acrescentar perguntas sobre opções de medicação (n = 1), sentir-se à vontade com o provedor (n = 1), capacidade de interromper ou recusar o tratamento (n = 1), abordagem de crises em tempo hábil (n = 3), papel do envolvimento da família (n = 5) e localização de serviços (n = 1). Os provedores sugeriram acrescentar perguntas sobre informações acessíveis (n = 2), eficácia geral do serviço (n = 1), avaliação de experiências anteriores (não de experiências atuais, n = 1), papel do envolvimento da família (n = 3), seguro (n = 1), preferências do provedor (n = 2) e outros tipos de abordagens terapêuticas (por exemplo, terapia ao ar livre, n = 1)."

Exemplo 2

"The Vitiligo Patient Priority Outcome (ViPPO) items were well understood by all participants, including adolescents (Supplementary Material 4). The instructions were largely understood (n = 55/60, 92%). Five participants initially considered their total body when completing the ViPPO facial vitiligo only (ViPPO-F) but understood the intention when the interviewer repeated the instruction.

No participants experienced difficulty understanding either response scale:

"I think it's good. I think it gives you plenty of choices, um, to, like, measure exactly where you feel about it." (Female, 51 years old; 'frequency' responses)

"I think they were a good choice of answers to pick out of." (Female, 13 years old; 'severity' responses)

When asked which response scale they preferred (Fig. 2), over half (n = 33/58, 57%) preferred 'frequency'; 18 (n = 18/58, 31%) preferred 'severity'; six (n = 6/58, 10%) had no preference; while an additional participant (n = 1/58, 2%) felt that their preference changed for each domain.

Fifty-three participants (n = 53/59, 90%) discussed the recall period. Of these, 24 (n = 24/53, 45%) understood and used the recall period correctly throughout:

"I'm thinking in the last seven days […] I kept saying that was the last seven days because some of my answers would be different if you would have asked me in the past, you know, six months." (Female, 37 years old)

Thirty-seven participants (n = 37/59, 63%) were asked whether it would be easy or difficult to recall the past 7 days, regardless of whether they had used the recall period correctly. Most (n = 29/37, 78%) felt that it would be easy. Eight (n = 8/37, 22%) felt that it would be difficult, half of whom (n = 4/8, 50%) suggested that the activities on the ViPPO-F are unlikely to have happened over a 7-day period, particularly the concepts included in the social functioning domain." [84]

Tradução: "Os itens do *Vitiligo Patient Priority Outcome* (ViPPO) foram bem compreendidos por todos os participantes, inclusive pelos adolescentes (Material Suplementar 4). As instruções foram amplamente compreendidas (n = 55/60, 92%). Cinco participantes inicialmente consideraram o corpo inteiro ao preencher o ViPPO apenas para vitiligo facial (ViPPO-F), mas entenderam a intenção quando o entrevistador repetiu a instrução.
Nenhum participante teve dificuldade para entender qualquer uma das escalas de resposta:
"Acho que é bom. Acho que ela lhe dá muitas opções para medir exatamente como você se sente em relação a isso." (Mulher, 51 anos; respostas de 'frequência')
"Acho que foi uma boa escolha de respostas para escolher." (Mulher, 13 anos; respostas de 'gravidade')
Quando perguntados sobre qual escala de resposta eles preferiam (Fig. 2), mais da metade (n = 33/58, 57%) preferiu "frequência"; 18 (n = 18/58, 31%) preferiram "gravidade"; seis (n = 6/58, 10%) não tinham preferência; enquanto um participante adicional (n = 1/58, 2%) sentiu que sua preferência mudava para cada domínio.
Cinquenta e três participantes (n = 53/59, 90%) discutiram o período de recordação. Desses, 24 (n = 24/53, 45%) entenderam e usaram o período de recordação corretamente durante todo o tempo:
"Estou pensando nos últimos sete dias [...] continuei dizendo que eram os últimos sete dias porque algumas das minhas respostas seriam diferentes se você tivesse me perguntado nos últimos, sabe, seis meses." (Mulher, 37 anos)
Trinta e sete participantes (n = 37/59, 63%) foram questionados se seria fácil ou difícil recordar os últimos sete dias, independentemente de terem usado o período de recordação corretamente. A maioria (n = 29/37, 78%) achou que seria fácil. Oito (n = 8/37, 22%) acharam que seria difícil, sendo que metade deles (n = 4/8, 50%) sugeriu que é improvável que as atividades do ViPPO-F tenham ocorrido em um período de 7 dias, especialmente os conceitos incluídos no domínio de funcionamento social."

CV6 - Compreensão

Especifique se os pacientes entenderam as instruções, os itens, as opções de resposta e o período de recordação da PROM conforme pretendido e/ou se os profissionais consideraram que as instruções, os itens, as opções de resposta e o período de recordação estavam adequadamente redigidos.

Explicação: Recomendamos que você especifique se os pacientes entenderam as instruções, os itens, as opções de resposta e o período de recordação da PROM conforme pretendido pelos desenvolvedores e/ou se os profissionais consideraram as instruções, os itens, as opções de resposta e o período de recordação adequadamente redigidos. Os resultados específicos podem incluir informações sobre a clareza da redação, quaisquer ambiguidades em potencial e a facilidade geral de compreensão do conteúdo da PROM. Se os pacientes e/ou profissionais identificarem questões importantes (por exemplo, alguns itens têm linguagem altamente técnica para a população), recomendamos que você informe o número (porcentagem) de itens que foram compreensíveis (e não compreensíveis) e indique quais itens (ou aspectos da PROM) foram considerados não compreensíveis. Se a sua conclusão for que a validade do conteúdo é insuficiente em termos de compreensão (veja o item DC1) e que a PROM precisa ser aprimorada, você pode dar sugestões de aprimoramento com base nos seus resultados (veja o item DC5).

Elementos essenciais

- Se os pacientes entenderam as instruções, os itens, as opções de resposta e o período de recordação da PROM.
- Se os profissionais consideraram as instruções, os itens, as opções de resposta e o período de recordação da PROM adequadamente redigidos.

Exemplo 1

"The intended meaning of the items was clearly understood by most of the participants (89-100%) (Figure 1). Two participants (11%) reported difficulty in understanding Item 10. One participant indicated that they could not differentiate between "falling asleep" and "falling back to sleep." The other participant indicated that "waking up," "preventing you from falling asleep," and "preventing you back to sleep" were the same concept. For Item 1 and 2, one participant was either not asked or did not reply to the question during the interview, resulting in missing data. (…) The Cough and Sputum Assessment Questionnaire (CASA-Q) instructions were well understood by all participants. The recall period was (...) [understood] by 89% (n=16) of the study participants. A majority of the participants (n=13 of 17, 76%) demonstrated understanding [by debriefing interviews] of the response options. The

remaining 24% (n=4) [participants] demonstrated difficulty in distinguishing specific response options, such as, distinguishing “rarely” from “sometimes,” distinguishing “quite a bit” from “a lot,” distinguishing “somewhat” from “a little,” and “often” from “always.”” [83]

Tradução: “O significado pretendido dos itens foi claramente compreendido pela maioria dos participantes (89-100%) (Figura 1). Dois participantes (11%) relataram dificuldade em entender o Item 10. Um participante indicou que não conseguia diferenciar entre “cair no sono” e “voltar a dormir”. O outro participante indicou que “acordar”, “impedir que você adormeça” e “impedir que você volte a dormir” eram o mesmo conceito. Para os Itens 1 e 2, um participante não foi perguntado ou não respondeu à pergunta durante a entrevista, o que resultou em dados faltantes. (...) As instruções do Co*ugh and Sputum Assessment Questionnaire* (CASA-Q) foram bem compreendidas por todos os participantes. O período de recordação foi (...) [compreendido] por 89% (n=16) dos participantes do estudo. A maioria dos participantes (n=13 de 17, 76%) demonstrou compreensão [nas entrevistas de interrogatório] das opções de resposta. Os 24% (n=4) [participantes] restantes demonstraram dificuldade em distinguir opções de resposta específicas, como distinguir “raramente” de “às vezes”, distinguir “bastante” de “muito”, distinguir “um tanto” de “um pouco” e “frequentemente” de “sempre””.

Validade estrutural

A validade estrutural refere-se ao grau em que as pontuações de uma PROM são um reflexo adequado da dimensão do construto a ser medido [1]. Existem dois tipos de modelos de medição: modelos reflexivos e formativos. Em um modelo reflexivo, os itens refletem um construto latente subjacente e espera-se que sejam altamente correlacionados e intercambiáveis. Em um modelo formativo, os itens juntos formam o construto e não precisam ser correlacionados [86, 87]. A análise fatorial e a análise da Teoria de Resposta ao Item (TRI) podem ser usadas para avaliar a validade estrutural das PROMs com base em um modelo reflexivo. O modelo fatorial (por exemplo, um modelo unidimensional) representa a estrutura pretendida da PROM e informa sobre seu algoritmo de pontuação (por exemplo, cálculo de uma pontuação de soma ou pontuação *Theta*). Os padrões para avaliar a validade estrutural dos modelos formativos ainda não fazem parte das diretrizes COSMIN.

Validade estrutural: Métodos

SV1 - Justificativa da abordagem

Forneça uma justificativa para a abordagem utilizada (por exemplo, análise fatorial, Teoria de Resposta ao Item (TRI)/Análise de Rasch).

Explicação: Para garantir que sua abordagem seja consistente com os objetivos do estudo, recomendamos que você informe por que escolheu a abordagem usada para avaliar a validade estrutural (por exemplo, análise fatorial exploratória ou confirmatória, Teoria de Resposta ao Item (TRI)/análise de Rasch). Essas abordagens exigem modelos de medição reflexivos. Portanto, recomendamos que você declare e explique que a PROM que está sendo estudada é, de fato, baseada em um modelo reflexivo. A análise fatorial exploratória (AFE) é indicada quando não existem hipóteses claras sobre as dimensões subjacentes. A análise fatorial confirmatória (AFC) testa se os dados se ajustam a uma estrutura fatorial predeterminada com base na teoria ou em análises anteriores. As hipóteses a priori sobre a estrutura devem ser relatadas e testadas [88]. Enquanto a análise fatorial permite que os pesquisadores explorem ou confirmem a estrutura fatorial subjacente de um instrumento, a análise TRI/Rasch pode ser usada para avaliar a relação entre as respostas do paciente e a característica latente.

Elementos essenciais

• Justificativa para a abordagem utilizada.

Exemplo 1

"For CFA, three models were compared: (...). These models were based on the results of EFA and compared with results on the internal structure of Pelvic Floor Distress Inventory (PFDI-20) in the literature, assuming that the three subscales of PFDI-20 form 3 dimensions (pelvic organ prolapse, anorectal and urinary symptoms) and the five factors found by Ma et al. [ref]." [30]

Tradução: "Para a AFC, três modelos foram comparados: (...). Esses modelos foram baseados nos resultados da AFE e comparados com os resultados sobre a estrutura interna do *Pelvic Floor Distress Inventory* (PFDI-20) na literatura, assumindo que as três subescalas do PFDI-20 formam 3 dimensões (prolapso de órgãos pélvicos, sintomas anorretais e urinários) e os cinco fatores encontrados por Ma et al. [ref]."

Exemplo 2

"Since the Norwegian Fatigue Characteristics and Interference Measure (FCIM) is a new instrument and includes items with different response categories, we applied a Partial Credit Model (PCM) which make no assumptions about the equidistance between thresholds across items [ref]. Then we assessed rating scale functioning according to Linacre's guidelines to determine whether the scale was suitable for Rasch analysis [ref] (Table1). The primary focus in the Rasch analysis was to address two main aims: item reduction and evaluation of FCIM's structural validity and internal consistency." [89]

Tradução: "Como a *Norwegian Fatigue Characteristics and Interference Measure* (FCIM) é um novo instrumento e inclui itens com diferentes categorias de resposta, aplicamos um Modelo de Crédito Parcial (MCP) que não faz suposições sobre a equidistância entre os limiares dos itens [ref]. Em seguida, avaliamos o funcionamento da escala de classificação de acordo com as diretrizes de Linacre para determinar se a escala era adequada para a análise de Rasch [ref] (Tabela 1). O foco principal da análise de Rasch era atender a dois objetivos principais: redução de itens e avaliação da validade estrutural e da consistência interna da FCIM."

SV2 - Análises estatísticas

Análises fatoriais exploratórias (AFE) ou Confirmatórias (AFC)

Descreva o modelo testado (por exemplo, número de fatores, quais itens estão incluídos em cada fator), o método de estimativa, o tipo de matriz de correlação e os métodos e critérios para um bom ajuste do modelo.

TRI/Análise de Rasch

Descreva o tipo de modelo TRI/Rasch, o método de estimativa, os métodos para verificar as suposições (por exemplo, dimensionalidade, independência local, monotonicidade), os métodos e critérios para bons parâmetros de itens e ajuste do modelo. Indique o software e a versão utilizada.

Outras abordagens

Forneça detalhes dos métodos, caso tenham sido usadas outras abordagens.

Explicação: A análise fatorial e a TRI/análise de Rasch são importantes para testar a dimensionalidade da PROM e para garantir que os itens sejam explicados de forma coerente por suas respectivas dimensões (reflexivas). Se a AFE ou a AFC foram usadas, você deve fornecer o método de estimação (por exemplo, máxima verossimilhança, mínimos quadrados ponderados etc.), o tipo de matriz de correlação (por exemplo, correlações policóricas para pontuações de itens ordinais), o(s) modelo(s) testado(s) (ou seja para a AFE, quantos fatores podem ser extraídos; para a AFC, um diagrama de caminho explicando quais itens são um reflexo de qual fator), métodos e critérios para um bom ajuste do modelo (por exemplo, lideranças de fatores; gráfico dos autovalores/*scree plot* e autovalores acima de 1 para a AFE; ou estatísticas de bom ajuste para a AFC (por exemplo, CFI, TLI ou medida comparável, RMSEA ou SRMR)). Se a TRI/análise de Rasch foi usada, recomendamos que você forneça o tipo de modelo da TRI usado (por exemplo, modelo de 1 ou 2 parâmetros (por exemplo, modelo de crédito parcial ou modelo de resposta gradual)), o método de estimação (por exemplo, máxima verossimilhança, mínimos quadrados ponderados/*Marginal Maximum Likelihood* (MML) *estimation* com base no algoritmo de *Expectation Maximization* (EM) ou a estimação totalmente bayesiana por meio do uso de técnicas de simulação de *Markov Chain Monte Carlo* (MCMC)), métodos para testar suposições (por exemplo, dimensionalidade, independência local, monotonicidade) e métodos e critérios usados para ajuste do modelo e retenção ou exclusão de itens.

Elementos essenciais

Para AFE/AFC:

• Método de estimação.

• Tipo de matriz de correlação.

• Modelo testado (por exemplo, número de fatores, quais itens estão incluídos em cada fator).

• Métodos e critérios para um bom ajuste do modelo.

Para TRI/análise de Rasch:

• Tipo de TRI/análise de Rasch (por exemplo, modelo de 1 ou 2 parâmetros (por exemplo, modelo de crédito parcial ou modelo de resposta gradual)).

• Método de estimação.

• Métodos para testar as suposições (por exemplo, dimensionalidade, independência local, monotonicidade).

• Métodos e critérios usados para ajuste do modelo e retenção ou exclusão de itens.

Exemplo 1

"(…) it was necessary to assess the structural validity of this WHODAS 2.0 version in a sample of women with dysmenorrhea by exploratory factor analysis and confirmatory factor analysis. To assess the factorability of the data, Kaiser-Meyer-Olkin (KMO) test and the Bartlett sphericity test were used. KMO ≥ 0.70 and p ≤ 0.05 in the Bartlett sphericity test indicated criterion to perform exploratory factor analysis. We performed exploratory factor analysis on the total sample, and used Minimum rank and Parallel Analysis to retain the number of factors with quartimax rotation. If necessary, items with factor loading < 0.40 were excluded [ref]. For the confirmatory factor analysis, the sample was divided equally and randomly in SPSS 22 [and tested in all models]. Thus, we used the maximum likelihood robust [method of estimation], Root Mean Square Error of Approximation (RMSEA), Standardized Root Mean Squared Residual (SRMR), Comparative Fit Index (CFI) and Tucker-Lewis Index (TLI). The model was considered adequate when RMSEA and SRMR < 0.08, CFI and TLI > 0.90 [ref]. Items with higher modification indices (MI) had error covariances." [18]

Tradução: "(…) foi necessário avaliar a validade estrutural dessa versão do WHODAS 2.0 em uma amostra de mulheres com dismenorreia por meio de análise fatorial exploratória e análise fatorial confirmatória. Para avaliar a fatorabilidade dos dados, foram usados o teste *Kaiser-Meyer-Olkin* (KMO) e o teste de esfericidade de Bartlett.

KMO ≥ 0,70 e p ≤ 0,05 no teste de esfericidade de Bartlett indicaram o critério para realizar a análise fatorial exploratória. Realizamos a análise fatorial exploratória na amostra total e usamos a classificação mínima e a análise paralela para reter o número de fatores com rotação quartimax. Se necessário, os itens com carga fatorial < 0,40 foram excluídos [ref]. Para a análise fatorial confirmatória, a amostra foi dividida de forma igual e aleatória no SPSS 22 [e testada em todos os modelos]. Assim, usamos o método robusto de máxima verossimilhança [método de estimação], *Root Mean Square Error of Approximation* (RMSEA), *Standardized Root Mean Squared Residual* (SRMR), *Comparative Fit Index* (CFI) e *Tucker-Lewis Index* (TLI). O modelo foi considerado adequado quando RMSEA e SRMR < 0,08, CFI e TLI > 0,90 [ref]. Os itens com índices de modificação (IM) mais altos tinham covariâncias de erro."

Exemplo 2

"We assessed unidimensionality using a scree plot, Kaiser criterion analysis (with a threshold of 1.0 eigenvalues) and a confirmatory factor analysis (CFA) with polychoric correlation and a diagonally weighted least squares estimator in the lavaan package (version 0.6-11). (…) We used the following fit statistics and thresholds to indicate good model fit: root mean squared error of approximation < 0.06, standardized root means square residual ≤ 0.08, comparative fit index ≥ 0.95, and Tucker-Lewis index ≥ 0.95. (…) We took Loevinger's Hi values > 0.3 to indicate monotonicity. (…) We tested for this [local independence] using Yen's Q3 residual correlation statistic, with a threshold of > 0.20 indicating undesirable local dependence between items. (…) Using the mirt package (version 1.36.1), we fitted graded response models (GRMs) to the complete preoperative item response sets in each dataset and used these to calculate IRT scores (specifically, expected a posteriori scores computed with a standard normal prior)." [90]

Tradução: "Avaliamos a unidimensionalidade usando um gráfico de dispersão, análise de critério de Kaiser (com um limite de 1,0 autovalor) e uma análise fatorial confirmatória (AFC) com correlação policórica e um estimador de mínimos quadrados ponderado diagonalmente no pacote lavaan (versão 0.6-11). (...) Usamos as seguintes estatísticas e limites de ajuste para indicar um bom ajuste do modelo: raiz do erro quadrático médio de aproximação < 0,06, raiz padronizada do quadrado médio residual ≤ 0,08, índice de ajuste comparativo ≥ 0,95 e índice de Tucker-Lewis ≥ 0,95. (...) Consideramos os valores Hi de Loevinger > 0,3 para indicar monotonicidade. (...) Testamos essa [independência local] usando a estatística de correlação residual Q3 de Yen, com um limite de > 0,20 indicando dependência local indesejável entre os itens. (...) Usando o pacote mirt (versão 1.36.1), ajustamos modelos de resposta gradual

(MRG) aos conjuntos completos de respostas de itens pré-operatórios em cada conjunto de dados e os usamos para calcular as pontuações da TRI (especificamente, pontuações a posteriori esperadas calculadas com uma prévia normal padrão)."

Validade estrutural: Resultados

SV3 - Análises estatísticas

AFE ou AFC

Para AFE: forneça todas as cargas fatoriais, autovalores e % de variância explicada do modelo que reflete a estrutura original da PROM e o modelo de melhor ajuste, se aplicável.
Para AFC: forneça todas as cargas fatoriais e os resultados dos índices de ajuste do modelo que refletem a estrutura original da PROM e o modelo de melhor ajuste, se aplicável.

TRI/Análise de Rasch

Forneça os resultados do ajuste do item/modelo, todos os parâmetros do item e figuras, se apropriado (por exemplo, curvas características do item, mapeamento pessoa-item, funções de informação do item e/ou do teste).

Outras abordagens

Forneça os resultados relevantes de outras abordagens utilizadas.

Explicação: Fornecer os resultados da análise fatorial ou da TRI/análise de Rasch é essencial para que os leitores do seu estudo interpretem a dimensionalidade da PROM e compreendam o desempenho do item. Recomendamos fornecer os resultados para a estrutura mais comumente usada da PROM (por exemplo, se uma pontuação total for sempre usada, recomendamos fornecer os resultados para um modelo unidimensional) e, se os resultados mostrarem que esse modelo não se sustenta em seus dados, forneça os resultados para outros modelos testados que se sustentam. Se você realizou a análise fatorial, recomendamos que forneça as cargas fatoriais do(s) modelo(s) testado(s), cujos resultados são usados para determinar o ajuste do modelo: para a AFE, recomendamos que você forneça os autovalores, um gráfico dos autovalores/*scree plot* e a porcentagem da variância explicada; para a AFC, recomendamos que você forneça os resultados das estatísticas de ajuste que você escolher usar (por exemplo, CFI, TLI, RMSEA etc.). Além disso, podem ser fornecidos os índices de modificação e as correlações residuais. Se você realizar a TRI, recomendamos que forneça informações sobre as suposições de unidimensionalidade e independência local, sobre o ajuste do item/modelo e todos os parâmetros do item. Além disso, recomenda-se que sejam fornecidas as curvas características do item, o mapeamento pessoa-item e as funções de informação do teste. Todas essas informações podem ser apresentadas em tabelas ou figuras no próprio manuscrito ou em um apêndice.

Elementos essenciais

Para AFE/AFC:

- Cargas de fator do modelo que reflete a estrutura original da PROM.
- Modelo de melhor ajuste (se aplicável).
- Autovalores e/ou % de variância explicada.
- Índices de ajuste do modelo.

Para TRI/análise de Rasch:

- Ajuste do item/modelo.
- Parâmetros do item.
- Pressupostos de unidimensionalidade e independência local.
- Figuras (se apropriado) (por exemplo, curvas características do item, mapeamento pessoa-item, funções de informação do teste).

Exemplo 1

"In the total sample, Bartlett's sphericity test [χ2(df) = 807.58(11), p < 0.0001] and KMO (0.913) were adequate. A single factor was suggested with an explained variance of 51% for WHODAS 2.0.

Table 2 presents the factor loadings (> 0.50) and fit indexes of the WHODAS 2.0 for the split sample. The confirmatory factor analysis showed the one-factor structure with the following fit indexes for model 1 [χ2(df) = 388.576(54), p < 0.001; CFI = 0.462; TLI = 0.342; RMSEA = 0.093 (90%CI 0.089-0.102); SRMR = 0.066]. To improve model fit, error covariances were freed items 8 and 9 (MI = 206.677), 10 and 11 (MI = 124.583), 1 and 7 (MI = 52.526), 1 and 2 (MI = 49.172), 2 and 11 (MI = 48.225), 5 and 6 (MI = 28.442), and 1 and 11 (MI = 22.067). This resulted in an adequately-fitting model 2 [χ2(df) = 93.118(46), p < 0.001; CFI = 0.924; TLI = 0.900; RMSEA = 0.038 (90%CI 0.027-0.049); SRMR = 0.029]. The path diagram of the model 2 for WHODAS 2.0 is shown in Fig. 1. Although the structure of model 2 contains correlated errors, it should be accepted as a better structure compared to model 1 due to better indexes."

Table 2. Factor loadings and fit indexes of WHODAS 2.0.

Items	Factor loadings Split sample (n=717)	
	Model 1	Model 2
1. Stand up	0.655	0.637
2. Do housework	0.698	0.705
3. Learn a new task	0.695	0.699
4. Participate in community activities	0.704	0.714
5. Be emotionally affected	0.675	0.668
6. Focus for 10 minutes	0.637	0.630
7. Walking for long distances	0.649	0.635
8. Take a shower	0.547	0.505
9. Dress up	0.617	0.586
10. Dealing with strangers	0.602	0.590
11. Keep a friendship	0.592	0.609
12. Work	0.652	0.657
χ^2(df)	388.576 (54)	93.118 (46)
CFI	0.462	0.924
TLI	0.342	0.900
RMSEA (90% CI)	0.093 (0.084 - 0.102)	0.038 (0.027 - 0.049)
SRMR	0.066	0.029

WHODAS 2.0: WHO Disability Assessment Schedule 2.0. CFI: Comparative Fit Index. df: degrees of freedom. RMSEA: Root Mean Square Error of Approximation. SRMR: Standardized Root Mean Squared Residual. TLI: Tucker-Lewis Index.

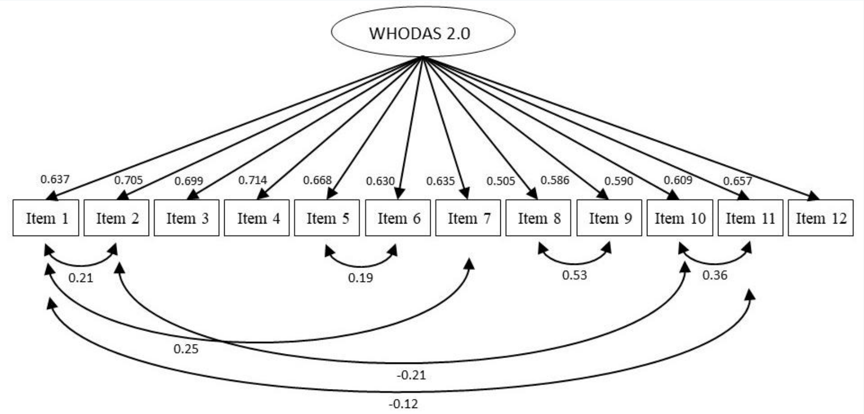

Figure 1. Path diagram of the WHODAS 2.0 for dysmenorrhea.

Table 3. Multigroup Confirmatory Factor Analysis among geographic regions of Brazil for the WHODAS 2.0.

Measurement invariance	χ^2(df)	CFI	RMSEA	$\Delta\chi^2$(df)	ΔCFI	ΔRMSEA
**Geographic regions of Brazil (Southeast vs. Northeast vs. South vs. North vs. Midwest)**						
Configural invariance	1337.078(270)	0.843	0.119	-	-	-
Metric invariance	1417.469(314)	0.838	0.113	80.391(44)	0.005	0.006
Scalar invariance	1507.029(358)	0.831	0.108	89.56(44)	0.007	0.005

WHODAS 2.0: WHO Disability Assessment Schedule 2.0. CFI: Comparative Fit Index. ΔCFI: Difference in Comparative Fit Index. RMSEA: Root Mean Square Error of Approximation. ΔRMSEA: Difference in Root Mean Square Error of Approximation. df: Degrees of freedom.

[18]

Tradução: "Na amostra total, o teste de esfericidade de Bartlett [χ2(gl) = 807,58(11), p < 0,0001] e o KMO (0,913) foram adequados. Um único fator foi sugerido com uma variância explicada de 51% para o WHODAS 2.0.

A Tabela 2 apresenta as cargas fatoriais (> 0,50) e os índices de ajuste do WHODAS 2.0 para a amostra dividida. A análise fatorial confirmatória mostrou a estrutura de um fator com os seguintes índices de ajuste para o modelo 1 [χ2(gl) = 388,576(54), p < 0,001; CFI = 0,462; TLI = 0,342; RMSEA = 0,093 (90%CI 0,089-0,102); SRMR = 0,066]. Para melhorar o ajuste do modelo, as covariâncias de erro foram liberadas nos itens 8 e 9 (MI = 206,677), 10 e 11 (MI = 124,583), 1 e 7 (MI = 52,526), 1 e 2 (MI = 49,172), 2 e 11 (MI = 48,225), 5 e 6 (MI = 28,442) e 1 e 11 (MI = 22,067). Isso resultou em um modelo 2 com ajuste adequado [χ2(gl) = 93,118(46), p < 0,001; CFI = 0,924; TLI = 0,900; RMSEA = 0,038 (90%CI 0,027-0,049); SRMR = 0,029]. O *path diagram* do modelo 2 do WHODAS 2.0 é mostrado na Fig. 1. Embora a estrutura do modelo 2 contenha erros correlacionados, ela deve ser aceita como uma estrutura melhor em comparação com o modelo 1 devido aos melhores índices."

Tabela 2. Cargas fatoriais e índices de ajuste do WHODAS 2.0.

Itens	Cargas de fator Amostra dividida (n=717)	
	Modelo 1	Modelo 2
1. Levantar	0,655	0,637
2. Fazer o trabalho doméstico	0,698	0,705
3. Aprender uma nova tarefa	0,695	0,699
4. Participar de atividades comunitárias	0,704	0,714
5. Ser afetado emocionalmente	0,675	0,668
6. Concentrar-se por 10 minutos	0,637	0,630
7. Caminhar por longas distâncias	0,649	0,635
8. Tomar banho	0,547	0,505
9. Vestir-se	0,617	0,586
10. Lidar com estranhos	0,602	0,590
11. Manter uma amizade	0,592	0,609
12. Trabalhar	0,652	0,657
χ^2(gl)<br>CFI<br>TLI<br>RMSEA (IC 90%)<br>SRMR	388,576 (54)<br>0,462<br>0,342<br>0,093 (0,084 - 0,102)<br>0,066	93,118 (46)<br>0,924<br>0,900<br>0,038 (0,027 - 0,049)<br>0,029

WHODAS 2.0: WHO *Disability Assessment Schedule* 2.0. CFI: *Comparative Fit Index*. gl: graus de liberdade. RMSEA: *Root Mean Square Error of Approximation*. SRMR: *Standardized Root Mean Squared Residual*. TLI: *Tucker-Lewis Index*.

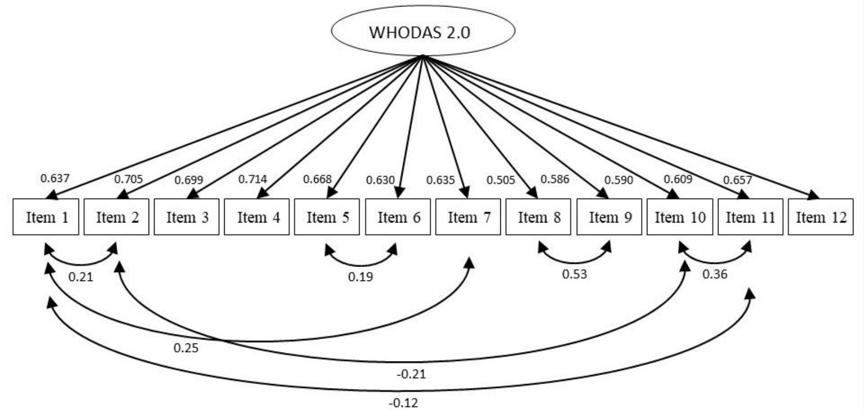

Figura 1. *Path diagram* do WHODAS 2.0 para dismenorreia.

Tabela 3. Análise fatorial confirmatória multigrupo entre regiões geográficas do Brasil para o WHODAS 2.0.

Invariância da medida	χ^2(gl)	CFI	RMSEA	$\Delta\chi^2$(gl)	ΔCFI	ΔRMSEA
**Regiões geográficas do Brasil (Sudeste vs. Nordeste vs. Sul vs. Norte vs. Centro-Oeste)**						
Invariância configural	1337,078(270)	0,843	0,119	-	-	-
Invariância métrica	1417,469(314)	0,838	0,113	80,391(44)	0,005	0,006
Invariância escalar	1507,029(358)	0,831	0,108	89,56(44)	0,007	0,005

WHODAS 2.0: WHO *Disability Assessment Schedule* 2.0. CFI: *Comparative Fit Index*. ΔCFI: *Difference in Comparative Fit Index*. RMSEA: *Root Mean Square Error of Approximation*. ΔRMSEA: *Difference in Root Mean Square Error of Approximation*. gl: graus de liberdade.

Exemplo 2

"Unidimensionality within the scales was concluded from the three performed single CFAs. (...) The basic IRT assumptions were evaluated and showed monotonicity (H for pain interference items ranged 0.59 to 0.70 [total scale H = 0.68], fatigue items ranged 0.53-0.69 [total scale H = 0.63] and physical activity items ranged 0.48-0.72 [total scale H = 0.65]), and local independence was found among the items. (...) The item parameter estimates and the χ2 mean square item fit statistics are shown in Table 2. In this table the items are sorted in order of decreasing discrimination (a), so the generally best indicators of pain interference, fatigue, and physical activity are near the top of the tables. The best and the worst discriminating items are shown in category characteristic curves, see Fig. 2. (…) For the pain interference items, five of the items exhibited significant lack of fit as indicated by the SS χ2 item fit ($p < 0.001$, χ2 ranged from 503.88 to 754.07, df = 391) (Table 2), after Benjamini-Hochberg correction for multiplicity. For the fatigue items, three of the items showed significant lack of fit ($p < 0.05$, χ2 ranged from 887.04 to 1232.74, df = 636), and for physical activity items, three items showed significant lack of fit ($p < 0.05$, χ2 ranged from 856.52 to 1007.04, df = 662).

The Test Information Function (TIF), Item Information Curves (IIC), and Standard Errors (SE), were satisfactory (see Fig. 3). SE for pain interference items ranged from 0.07 to 0.62 (M = 0.35, SD = 0.68), SE for fatigue items ranged from 0.11 to 0.49 (M = 0.19, SD = 0.70), and SE for physical activity items ranged from 0.16 to 0.52 (M = 0.22, SD = 0.70).”

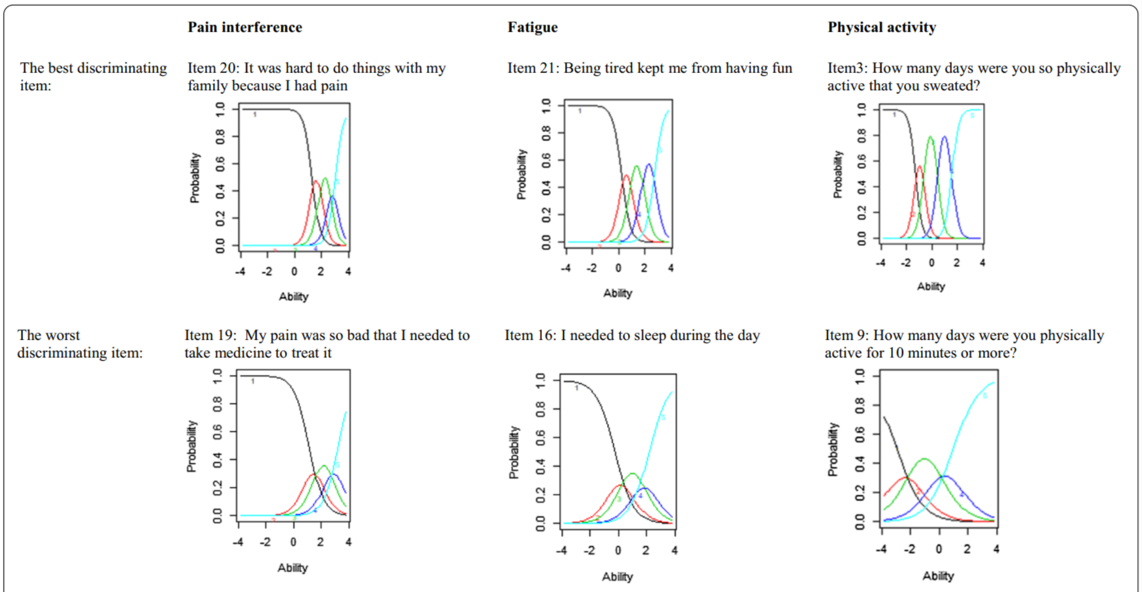

Fig. 2 The best and worst discriminating items in the Swedish translated item

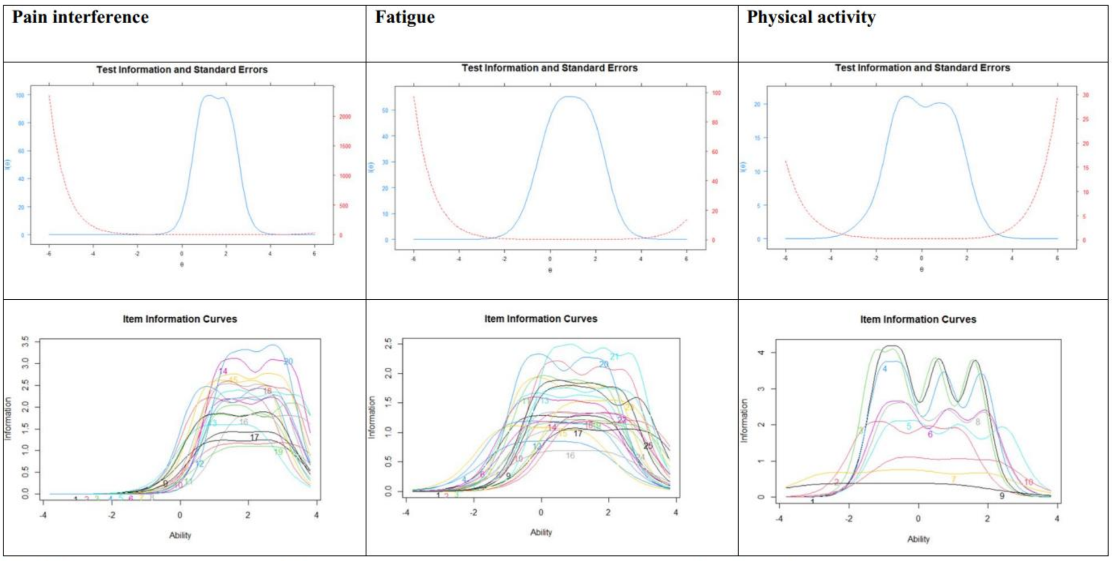

Fig. 3 Test information function, standard error, item information curves of the Swedish translated PROMIS item banks

[91]

Tradução: "(...) As premissas básicas da TRI foram avaliadas e mostraram monotonicidade (H para os itens de interferência da dor variou de 0,59 a 0,70 [escala total H = 0,68], os itens de fadiga variaram de 0,53 a 0,69 [escala total H = 0,63] e os itens de atividade física variaram de 0,48 a 0,72 [escala total H = 0,65]), e foi encontrada independência local entre os itens. (...) As estimativas dos parâmetros do item e as estatísticas de ajuste do item do χ2 quadrado médio são mostradas na Tabela 2. Nessa tabela, os itens estão classificados em ordem decrescente de discriminação (a), de modo que os melhores indicadores gerais de interferência da dor, fadiga e atividade física estão próximos ao topo da tabela. Os itens com melhor e pior discriminação são mostrados nas curvas características da categoria, veja a Fig. 2. (...) Para os itens de interferência da dor, cinco dos itens exibiram falta significativa de ajuste, conforme indicado pelo ajuste do item SS χ2 ($p < 0,001$, χ2 variou de 503,88 a 754,07, gl = 391) (Tabela 2), após a correção de Benjamini-Hochberg para multiplicidade. Para os itens de fadiga, três dos itens mostraram falta de ajuste significativo ($p < 0,05$, χ2 variou de 887,04 a 1232,74, gl = 636), e para os itens de atividade física, três itens mostraram falta de ajuste significativo ($p < 0,05$, χ2 variou de 856,52 a 1007,04, gl = 662).
A Função de Informação do Teste (FIT), as Curvas de Informação do Item (CII) e os Erros Padrão (EP) foram satisfatórios (veja a Fig. 3). O EP para os itens de interferência da dor variou de 0,07 a 0,62 (M = 0,35, DP = 0,68), o SE para os itens de fadiga variou de 0,11 a 0,49 (M = 0,19, DP = 0,70) e o SE para os itens de atividade física variou de 0,16 a 0,52 (M = 0,22, DP = 0,70)."

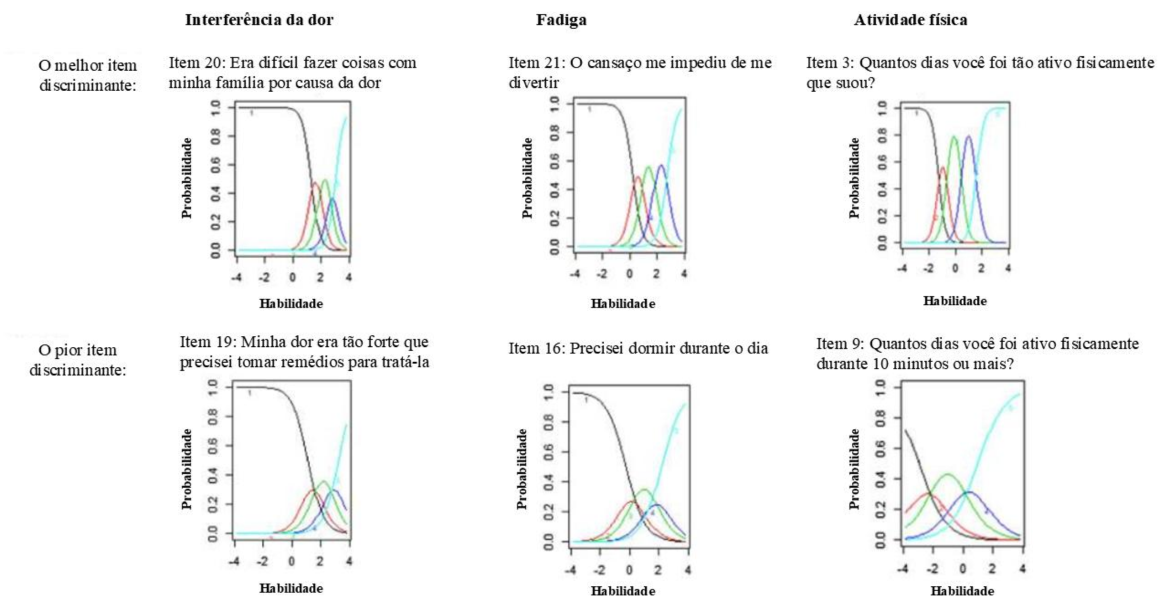

Fig. 2 Os melhores e os piores itens discriminantes no item sueco traduzido

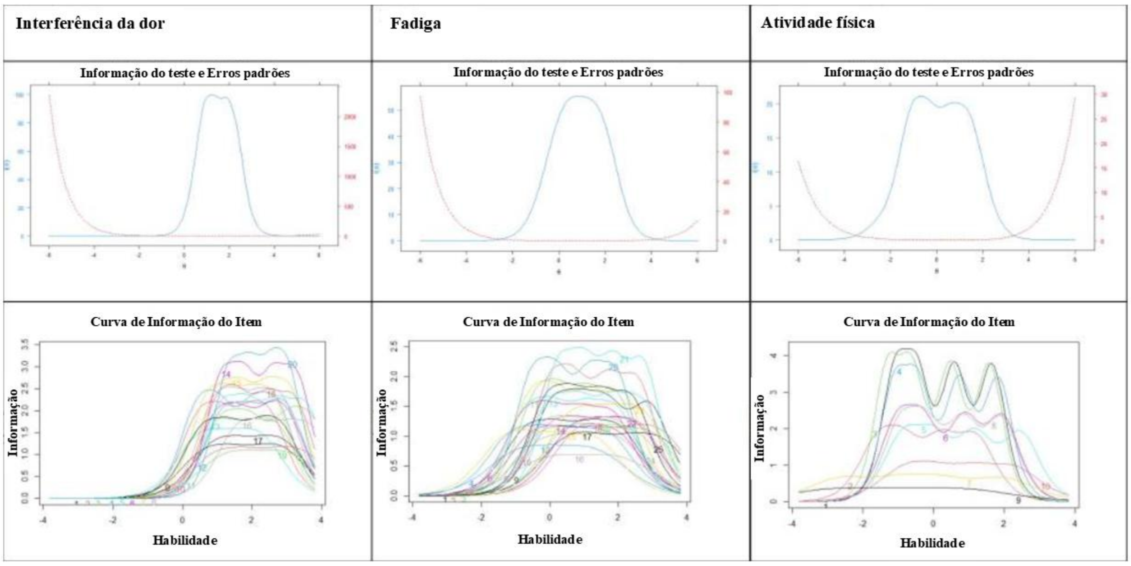

Fig. 3 Função de informação do teste, erro padrão e curvas de informação dos itens das versões traduzidas para o sueco dos bancos de itens PROMIS

Consistência interna

A consistência interna se refere ao grau de inter-relação entre os itens e faz parte do domínio confiabilidade [1]. Um estudo sobre confiabilidade informa sobre a influência de fontes de variação na pontuação observada [92]. Em um estudo de consistência interna, os itens são considerados medições repetidas dos mesmos construtos subjacentes, e itens diferentes são variados entre as medições repetidas. O objetivo é avaliar se há "medições repetidas" suficientes (ou seja, itens suficientes) em uma (sub)escala. Para uma interpretação apropriada do parâmetro de consistência interna, os itens juntos devem formar uma escala unidimensional (e, portanto, com base em um modelo reflexivo), pois são considerados medições repetidas do mesmo construto (consulte a validade estrutural para saber como avaliar a unidimensionalidade).

Consistência interna: Métodos

IC1 - Análises estatísticas

Forneça evidências da unidimensionalidade da PROM (subescalas) e forneça evidências da ausência de dependência local do item. Descreva os métodos estatísticos usados para calcular a consistência interna.

Explicação: Para poder interpretar adequadamente a estatística de consistência interna (por exemplo, Alfa de Cronbach), a escala precisa ser unidimensional. Portanto, recomendamos que você verifique a suposição de unidimensionalidade, por exemplo, mostrando os resultados de uma análise fatorial ou citações de estudos em que isso é demonstrado. Isso pode vir de um estudo sobre validade estrutural (no caso de PROMs multidimensionais) ou, alternativamente, a unidimensionalidade por subescala pode ser testada. Se for usada uma pontuação total para um PROM multidimensional, deve ser fornecida evidência de que essa pontuação total também se encaixa em um fator (por exemplo, com base em um modelo de fator único, o fator geral em um modelo bifatorial ou o fator de primeira ordem em um modelo de fator hierárquico). A consistência interna é calculada posteriormente com o Alfa de Cronbach, estatísticas Ômega-total ou um erro padrão (EP) de *Theta* (em um estudo de TRI/de Rasch). Recomendamos que você informe qual estatística foi usada.

Elementos essenciais

- Evidência da unidimensionalidade da PROM (subescalas).
- Estatística utilizada.

Exemplo 1

"Cronbach's alpha was calculated separately for each construct [unidimensional scale] of the Bilateral Vestibulopathy Questionnaire (BVQ) to measure the internal consistency of each item in its respective construct. A low Cronbach's alpha suggested a low or absent correlation between each item in the same construct, whereas a very high Cronbach's alpha indicated that the items measured identical concepts. Therefore, Cronbach's alpha was considered good when it ranged from 0.70 to 0.95 [ref]." [93]

Tradução: "O alfa de Cronbach foi calculado separadamente para cada construto (escala unidimensional) do Questionário de Vestibulopatia Bilateral (BVQ) a fim de medir a consistência interna de cada item em seu respectivo construto. Um alfa de Cronbach baixo sugeriu uma correlação baixa ou ausente entre os itens do mesmo construto, enquanto um alfa de Cronbach muito alto indicou que os itens medeiam conceitos idênticos. Portanto, o alfa de Cronbach foi considerado bom quando variava de 0,70 a 0,95 [ref]."

Consistência interna: Resultados

IC2 - Análises estatísticas

Forneça os resultados de consistência interna para cada escala unidimensional ou subescala separadamente.

Explicação: Fornecer os resultados da consistência interna ajuda a estabelecer a confiabilidade do instrumento. Você deve fornecer os resultados de cada escala unidimensional ou subescala separadamente. Se a consistência interna foi calculada para várias (sub)escalas, você pode apresentar os resultados em uma tabela.

Elementos essenciais

• Resultados para cada escala ou subescala unidimensionais separadamente.

Exemplo 1

"(...) exploratory factor analyses (EFA) (...) suggested the one-dimensionality of the Pelvic Floor Distress Inventory (PFDI-20) (…) The Cronbach's alpha value of the one-dimensional model was considered adequate (α = 0.93)." [30]

Tradução: "(...) a análise fatorial exploratória (AFE) (...) sugeriu a unidimensionalidade do *Pelvic Floor Distress Inventory* (PFDI-20) (...) O valor alfa de Cronbach do modelo unidimensional foi considerado adequado (α = 0,93)."

Validade transcultural/Invariância da medida

A validade transcultural refere-se ao grau em que o desempenho dos itens de uma PROM traduzida ou adaptada culturalmente é um reflexo adequado do desempenho dos itens da versão original da PROM. Ao usar as PROMs, presumimos que os pacientes de diferentes grupos (por exemplo, diferentes países ou idiomas) com a mesma pontuação verdadeira no construto ou traço latente (o mesmo nível de, por exemplo, dor ou depressão), têm a mesma probabilidade de escolher uma determinada opção de resposta em um item da PROM. Se esse não for o caso, o item não tem invariância da medida ou ocorre funcionamento diferencial do item/*Differential Item Functioning* (DIF) nesse item. As violações da invariância da medida ou a ocorrência de DIF podem impedir a interpretação significativa dos dados de medição. Por exemplo, um item não foi bem traduzido e tem outro significado no novo idioma; ou, devido a diferenças culturais, um item pode ter outro significado. Por exemplo, o item se você consegue se levantar do vaso sanitário pode ter outro significado para as pessoas na Holanda do que para as pessoas no Canadá, porque, em geral, os vasos sanitários no Canadá são mais baixos e é mais difícil se levantar de um vaso sanitário mais baixo.

O teste de invariância da medida geralmente envolve uma série de análises estatísticas que comparam a estrutura do modelo de medida entre grupos, usando a Análise Fatorial Confirmatória Multigrupo (AFCMG) ou testes DIF. Cada abordagem tem vantagens exclusivas: a AFCMG avalia se a estrutura fatorial (invariância configural) e as cargas fatoriais (invariância métrica) são equivalentes entre os grupos; o DIF pode ser avaliado por uma série de análises de regressão e permite uma análise detalhada em nível de item. Primeiro, testa se a resposta a um item (desfecho) depende do uso da pontuação *Theta* baseada na TRI (em que *Theta* representa o traço latente que está sendo medido) ou da soma das pontuações (que se baseia na pontuação total ou na soma das respostas a itens individuais em uma escala) (modelo 1); em segundo lugar, testa se uma variável do grupo também determina a resposta ao item (resultado) (modelo 2); por último, testa se há mesmo uma interação entre a pontuação *Theta* ou a pontuação da soma e a variável do grupo (modelo 3). Ao comparar os modelos 1 e 2, você testa o DIF uniforme; ao comparar os modelos 2 e 3, você testa o DIF não uniforme. Para executar qualquer uma dessas análises, você precisa ter dados da PROM de dois ou mais (sub)grupos diferentes (por exemplo, usando duas versões de idiomas diferentes). Os subgrupos também podem ser baseados em qualquer outra variável, como idade, gênero e características da doença. Os princípios para testar a invariância da medida ou DIF são os mesmos, independentemente de qual variável de subgrupo é usada na análise.

Validade transcultural/Invariância da medida: Métodos

CCV1 - Variável de grupo

Descreva a variável que difere entre os subgrupos que estão sendo comparados.

Explicação: Para avaliar a validade transcultural, você compara dados de dois (ou mais) subgrupos que têm características demográficas e de doença semelhantes, exceto pelo fato de que eles diferem em uma característica específica (ou seja, a variável do grupo). Aqui, você descreve a(s) variável(is) para a(s) qual(is) testará a invariância da medida ou DIF (por exemplo, idioma, país, sexo, idade etc.).

Elementos essenciais

• Descreva a variável que é usada para criar subgrupos nos quais a invariância da medida está sendo testada.

Exemplo 1

"For measurement invariance, we conducted multigroup CFA (MGCFA) between groups (on-menses and off-menses women) and sociodemographic region of Brazil (Southeast, North East, South, North and Midwest) in total sample (…)." [31]

Tradução: "Para verificar a invariância da medida, realizamos uma AFC multigrupo (AFCMG) entre os grupos (mulheres menstruadas e não menstruadas) e a região sociodemográfica do Brasil (Sudeste, Nordeste, Sul, Norte e Centro-Oeste) na amostra total (...)."

CCV2 - Justificativa da abordagem
Forneça uma justificativa para a abordagem usada (por exemplo, análise fatorial confirmatória multigrupo ou análise de regressão logística do funcionamento diferencial do item/*Differential Item Functioning* (DIF) usando *Theta* ou pontuações de soma, ou outra).

Explicação: Várias abordagens, como AFCMG e DIF com base em análises de regressão logística, podem ser usadas para avaliar a validade transcultural/invariância da medida de uma PROM. Recomendamos que você forneça uma justificativa para a abordagem usada para avaliar essa propriedade de medida. Ao fornecer uma justificativa para a abordagem usada para analisar os dados quanto à validade transcultural/invariância da medida, você permite que os leitores do seu artigo entendam as escolhas metodológicas feitas para garantir a aplicabilidade da PROM em diferentes grupos culturais.

Elementos essenciais
• Justificativa da abordagem usada.

Exemplo 1

"The influence of gender and age on the factor structures within the models was examined by multiple-group confirmatory factor analysis (CFA) invariance evaluation [ref] to determine whether the factor structure remained consistent across different groups. This analysis enabled the assessment of whether the relationships between the observed indicators and the latent factors are comparable and invariant across various groups." [94]

Tradução: "A influência do gênero e da idade nas estruturas fatoriais dentro dos modelos foi examinada pela avaliação de invariância [ref] da análise fatorial confirmatória (AFC) de vários grupos para determinar se a estrutura fatorial permaneceu consistente em diferentes grupos. Essa análise permitiu avaliar se as relações entre os indicadores observados e os fatores latentes são comparáveis e invariantes em vários grupos."

Exemplo 2

"We used an iterative hybrid approach of ordinal logistic regression (OLR) and item-response theory (IRT) as implemented in the lordif R-package [ref]. This procedure was used to maintain high comparability with other studies that investigated differential item functioning (DIF) in PROMIS® Pain interference (PROMIS PI) items." [95]

Tradução: “Usamos uma abordagem híbrida iterativa de regressão logística ordinal (RLO) e teoria de resposta ao item (TRI), conforme implementado no pacote R *lordif* [ref]. Esse procedimento foi usado para manter a alta comparabilidade com outros estudos que investigaram o *Differential Item Functioning* (DIF) nos itens do PROMIS® *Pain interference* (PROMIS PI).”

CCV3 - Análises estatísticas

Análise fatorial confirmatória multigrupo (AFCMG)

Forneça o modelo testado, o método de estimativa, o tipo de matriz de correlação e os métodos e critérios para alteração no ajuste do modelo.

Análises do funcionamento diferencial do item/*Differential Item Functioning* (DIF)

Descreva a abordagem estatística utilizada para calcular a pontuação do paciente (por exemplo, pontuação *Theta* baseada em TRI/Rasch ou pontuação de soma (não) ponderada), uma descrição dos modelos de regressão testados e os critérios utilizados para sinalizar itens para DIF.

Outras abordagens

Forneça detalhes dos métodos caso outras abordagens tenham sido usadas.

Explicação: Se a AFCMG foi usada para avaliar a validade transcultural\invariância da medida, recomendamos que você forneça o método de estimação (veja o item SV2), o tipo de matriz de correlação usada (veja o item SV2) e informações sobre os modelos testados (em termos de estrutura interna da PROM). A AFCMG compara modelos com níveis crescentes de restrições para determinar se a PROM funciona de forma semelhante entre os grupos. Recomendamos que você descreva o tipo de invariância da medida testada (ou seja, invariância configural, métrica, escalar e residual). Além disso, recomendamos que você forneça informações sobre os métodos (por exemplo, *Root Mean Square Error of Approximation* - RMSEA, *Comparative Fit Index* - CFI, *Tucker-Lewis Index* - TLI, etc.) e os critérios para mudanças relevantes no ajuste do modelo (por exemplo, magnitude da mudança nos índices de adequação). Para as análises de DIF baseadas em regressão, recomendamos que você descreva como a pontuação do paciente (usada como determinante no modelo de regressão) foi calculada (por exemplo, pontuação *Theta* (e o tipo de modelo de TRI/de Rasch usado (veja o item SV2)) ou pontuação de soma (não) ponderada), quais modelos de regressão subsequentes foram testados e comparados (tanto para DIF uniforme quanto não uniforme) e os critérios usados para sinalizar itens para DIF. Se outras abordagens foram usadas, forneça detalhes sobre os métodos utilizados.

Elementos essenciais

Para a AFCMG:

• Modelo testado (por exemplo, número de fatores, quais itens foram incluídos em cada fator).
• Método de estimação.

- Tipo de matriz de correlação.
- Métodos e critérios para mudanças relevantes no ajuste do modelo.

Para DIF:

- Tipo de TRI/análise de Rasch (por exemplo, modelo de crédito parcial, modelo de resposta gradual etc.).
- Descrição do modelo de regressão testado, se aplicável.
- Tipo de DIF.
- Critérios usados para marcar itens para DIF.

Exemplo 1

"The other main objective of this study is the testing of Measurement invariance (MI) across different cultural backgrounds. For MI testing, the two samples were tested in one model using a multigroup confirmatory factor analysis (MGCFA) for the CPC-12R. The procedure of the MGCFA was inspired by Rudnev et al. [ref] as well as by Chen et al. [ref]. The MGCFA was conducted using MLR [Maximum likelihood with robust standard errors] as the estimator. As a prerequisite for the MGCFA on second-order level, the levels of invariance (viz., configural, metric, scalar, and residual invariance) were tested for the first-order factors aforehand. To test the MI, the criteria proposed by Cheung and Rensvold [ref] and those from Chen [ref] were used. Cheung and Rensvold declare that a ΔCFI larger than −0.01 should lead to the null hypothesis of invariance being rejected. (…) In this study, for MI testing with an adequate sample size (total N > 300), unequal sample sizes and mixed lack of invariance, the following cut-offs proposed by Chen [ref] were applied: For testing loading invariance, a change of ≥ −0.01 in the CFI, in addition with a change of ≥ 0.015 in RMSEA, or a change of ≥ 0.03 in SRMR indicates non-invariance. When testing intercept or residual invariance, a change of ≥ −0.01 in CFI, supplemented by a change of ≥ 0.015 in RMSEA, or a change of ≥ 0.01 in SRMR indicates non-invariance." [96]

Tradução: "O outro objetivo principal deste estudo é o teste de invariância da medida (IM) em diferentes contextos culturais. Para o teste de IM, as duas amostras foram testadas em um modelo usando uma análise fatorial confirmatória multigrupo (AFCMG) para o CPC-12R. O procedimento da AFCMG foi inspirado em Rudnev et al. [ref] e também em Chen et al. [ref]. A AFCMG foi conduzida usando o MLR [*Maximum likelihood with robust standard errors*] como estimador. Como pré-requisito para o AFCMG no nível de segunda ordem, os níveis de invariância (ou seja, invariância configural, métrica, escalar e residual) foram testados para os fatores de primeira ordem anteriormente. Para testar a IM, foram usados os critérios propostos por Cheung e

Rensvold [ref] e os de Chen [ref]. Cheung e Rensvold declaram que um ΔCFI maior que -0,01 deve levar à rejeição da hipótese nula de invariância. (...) Neste estudo, para o teste de IM com um tamanho de amostra adequado (N total > 300), tamanhos de amostra desiguais e falta de invariância mista, foram aplicados os seguintes pontos de corte propostos por Chen [ref]: Para testar a invariância de carga, uma alteração ≥ -0,01 no CFI, além de uma alteração ≥ 0,015 no RMSEA, ou uma alteração ≥ 0,03 no SRMR indica não invariância. Ao testar a invariância residual ou de interceptação, uma alteração ≥ -0,01 no CFI, complementada por uma alteração ≥ 0,015 no RMSEA, ou uma alteração ≥ 0,01 no SRMR indica não invariância."

Exemplo 2

"DIF for gender, age (median split), language (Swedish translated vs US original pediatric PROMIS item banks of pain and fatigue) [ref], were calculated for each item on each scale using the IRT Likelihood Ratio DIF approach [ref], using LR χ2 item fit statistics, as implemented in the software R package mirt [ref]. The Benjamini-Hochberg procedure [ref] was used to control for multiplicity of comparisons in DIF (see Table 2). McFadden's R^2 was used to evaluate when DIF was detected (> 2%) [ref]. McFadden's R^2 could be interpreted as < 0.035 = negligible DIF, 0.035-0.07 = moderate DIF, and > 0.07 = large DIF [ref]. The level of the effect size was evaluated tabular and graphically using methods outlined by Steinberg and Thissen [ref] for items with significant DIF." [91]

Tradução: "O DIF para gênero, idade (divisão da mediana), idioma (bancos de itens de dor e fadiga do PROMIS pediátrico traduzido para o sueco versus original dos EUA) [ref] foi calculado para cada item em cada escala usando a abordagem DIF de razão de verossimilhança da TRI [ref], usando estatísticas de ajuste de item LR χ2, conforme implementado no pacote de software R mirt [ref]. O procedimento Benjamini-Hochberg [ref] foi usado para controlar a multiplicidade de comparações no DIF (veja a Tabela 2). O R^2 de McFadden foi usado para avaliar quando o DIF foi detectado (> 2%) [ref]. O R^2 de McFadden pode ser interpretado como < 0,035 = DIF insignificante, 0,035-0,07 = DIF moderado e > 0,07 = DIF grande [ref]. O nível do tamanho do efeito foi avaliado em tabelas e gráficos usando os métodos descritos por Steinberg e Thissen [ref] para itens com DIF significativo."

Validade transcultural/Invariância da medida: Resultados

CCV4 - Análises estatísticas

Forneça resultados sobre mudanças ou diferenças no ajuste do modelo entre os modelos testados (por exemplo, AFCMG, TRI/Análise de Rasch, análises de regressão logística de DIF ou outras abordagens).

Explicação: Se a AFCMG foi usada, recomendamos que você informe os índices de ajuste do modelo (por exemplo, CFI, TLI, RMSEA) por modelo (ou seja, modelos configural, métrico, escalar ou residual) e, especificamente, as alterações no ajuste do modelo entre os modelos (por exemplo, ΔCFI, ΔRMSEA). Se forem feitas modificações (por exemplo, covariância entre itens) para obter invariância da medida, recomendamos que você informe os índices de modificação e as justificativas para quaisquer alterações. Se foram realizadas análises de regressão para testar o DIF, recomendamos que você informe as estatísticas de DIF para cada item avaliado (por exemplo, índice de Mantel-Haenszel, coeficientes de regressão logística ou parâmetros de itens em modelos da TRI).

Elementos essenciais

• Resultados para estatísticas calculadas.

Exemplo 1

"The resulting fit indices of the test for configural invariance, metric invariance and scalar invariance are displayed in Table 2. Indices of the second-order MGCFA, that should be emphasized, are the CFI = 0.97; TLI = 0.97; RMSEA = 0.05; 90% CI RMSEA = (0.04, 0.06) and SRMR = 0.05, regarding the metric invariance. Furthermore, the CFI = 0.97; TLI = 0.97; RMSEA = 0.05; 90% CI RMSEA = (0.04, 0.07) and SRMR = 0.06."

Table 2. Confirmatory factor analyses and measurement invariance results

Model	χ^2 (df)	Sig.	CFI	TLI	RMSEA [CI]	SRMR
CFA German Sample	81.69 (50)	0.003	0.97	0.96	0.07 [0.46, 0.83]	0.05
CFA US sample	160.72 (100)	<0.001	0.98	0.97	0.05 [0.03, 0.64]	0.04
Configural invariance	160.72 (100)	<0.001	0.98	0.97	0.05 [0.04, 0.06]	0.04
Metric invariance	184.70 (111)	<0.001	0.97	0.97	0.05 [0.04, 0.07]	0.05
Scalar invariance	199.47 (118)	<0.001	0.97	0.97	0.05 [0.04, 0.07]	0.06

χ^2 refers to the Chi-square difference value with respective degrees of freedom (df). Sig. is used to display the p-value of the Chi-square difference test. The confirmatory fit index is reported as CFI, the Tucker-Lewis index as TLI. RMSEA is the robust Root Mean Square Error of Approximation with respective 90%-confidence intervals [CI]. The SRMR is the Root Mean Square Residual. CFA means confirmatory factor analysis. MGCFA means multigroup confirmatory factor analysis.
[96]

Tradução: "Os índices de ajuste resultantes do teste de invariância configural, invariância métrica e invariância escalar são exibidos na Tabela 2. Os índices da AFCMG de segunda ordem, que devem ser enfatizados, são o CFI = 0,97; TLI = 0,97; RMSEA = 0,05; 90% CI RMSEA = (0,04, 0,06) e SRMR = 0,05, com relação à invariância métrica. Além disso, o CFI = 0,97; TLI = 0,97; RMSEA = 0,05; 90% CI RMSEA = (0,04, 0,07) e SRMR = 0,06."

Tabela 2. Análises fatoriais confirmatórias e resultados de invariância da medida

Modelo	χ^2 (gl)	Sig.	CFI	TLI	RMSEA [IC]	SRMR
Amostra de AFC alemã	81,69 (50)	0,003	0,97	0,96	0,07 [0,46; 0,83]	0,05
Amostra de AFC dos Estados Unidos	160,72 (100)	<0,001	0,98	0,97	0,05 [0,03; 0,64]	0,04
Invariância configural	160,72 (100)	<0,001	0,98	0,97	0,05 [0,04; 0,06]	0,04
Invariância métrica	184,70 (111)	<0,001	0,97	0,97	0,05 [0,04; 0,07]	0,05
Invariância escalar	199,47 (118)	<0,001	0,97	0,97	0,05 [0,04; 0,07]	0,06

χ^2 refere-se ao valor da diferença do qui-quadrado com os respectivos graus de liberdade (gl). Sig. é usado para exibir o valor de p do teste de diferença de qui-quadrado. O índice de ajuste confirmatório é relatado como CFI e o índice de Tucker-Lewis como TLI. RMSEA é a raiz robusta do erro quadrático médio de aproximação com os respectivos intervalos de confiança de 90% [IC]. O SRMR é a raiz quadrada média residual. AFC significa análise fatorial confirmatória. AFCMG significa análise fatorial confirmatória multigrupo.

Exemplo 2

"Table 2 shows the results of the DIF analyses. Two items in the anxiety item bank, 'It scared me when I felt nervous' (EDANX03) and 'I felt worried' (EDANX30), showed uniform and non-uniform DIF, respectively. The item 'I felt worried' is present in the PROMIS anxiety 7a short form. The items are present in respectively 1 and 3% of the CAT-based assessments. In the depression item bank, two items showed uniform DIF: 'I felt worthless' (EDDEP04) and 'I felt unhappy' (EDDEP36). (...) For the item 'It scared me when I felt nervous', the threshold parameters for the Dutch population were mostly slightly lower than the thresholds for the US population, indicating that the Dutch population endorses higher response categories at the same level of anxiety. (...) Fig 1 illustrates the impact of DIF on respondents total scores. The plots on the left show the impact of DIF when all items are considered, whereas the plots on the right show the impact of DIF when only DIF items are considered. The plots show that DIF had a minimal impact on the total score when all items are administered in each item bank."

Table 2. McFadden's pseudo R^2 and IRT parameters for items displaying DIF

Item bank	Item with DIF	DIF type	McFadden's pseudo R^2	Slope; and threshold parameters	Included in CAT[d]
Anxiety	EDANX03: It scared me when I felt nervous	Uniform	$R^2_{12} = 0.021$<br>$R^2_{23} = 0.011$	**NL:** 2.62; 0.15, 0.97, 2.02<br>US: 3.74; 0.59, 1.18, 1.95	1%
	EDANX30: I felt worried[a]	Non-uniform	$R^2_{12} = 0.010$<br>$R^2_{23} = 0.033$	NL: 2.16; -1.12, -0.10, 1.29, 2.64<br>US: 3.14; -0.57, 0.24, 1.22, 2.12	3%
Depression	EDDEP04: I felt worthless[b]	Uniform	$R^2_{12} = 0.024$<br>$R^2_{23} = 0.013$	**NL:** 2.93; -0.17, 0.58, 1.56, 2.61<br>US: 4.37; 0.29, 0.88, 1.61, 2.36	8%
	EDDEP36: I felt unhappy[c]	Uniform	$R^2_{12} = 0.037$<br>$R^2_{23} = 0.001$	NL: 4.21; -0.14, 0.61, 1.33, 2.20<br>**US:** 3.44; -0.64, 0.23, 1.20, 2.17	100%

The bold population had lower thresholds compared to the other population, indicating that this population endorses higher item response categories at the same level of the domain (anxiety, depression)
[a] present in the anxiety 7a short form
[b] present in the depression 4a, 6a, 8a and 8b short form
[c] present in the depression 6a, 8a and 8b short form
[d] Based on 4047 CAT-based assessments for anxiety and 4293 CAT-based assessments for depression

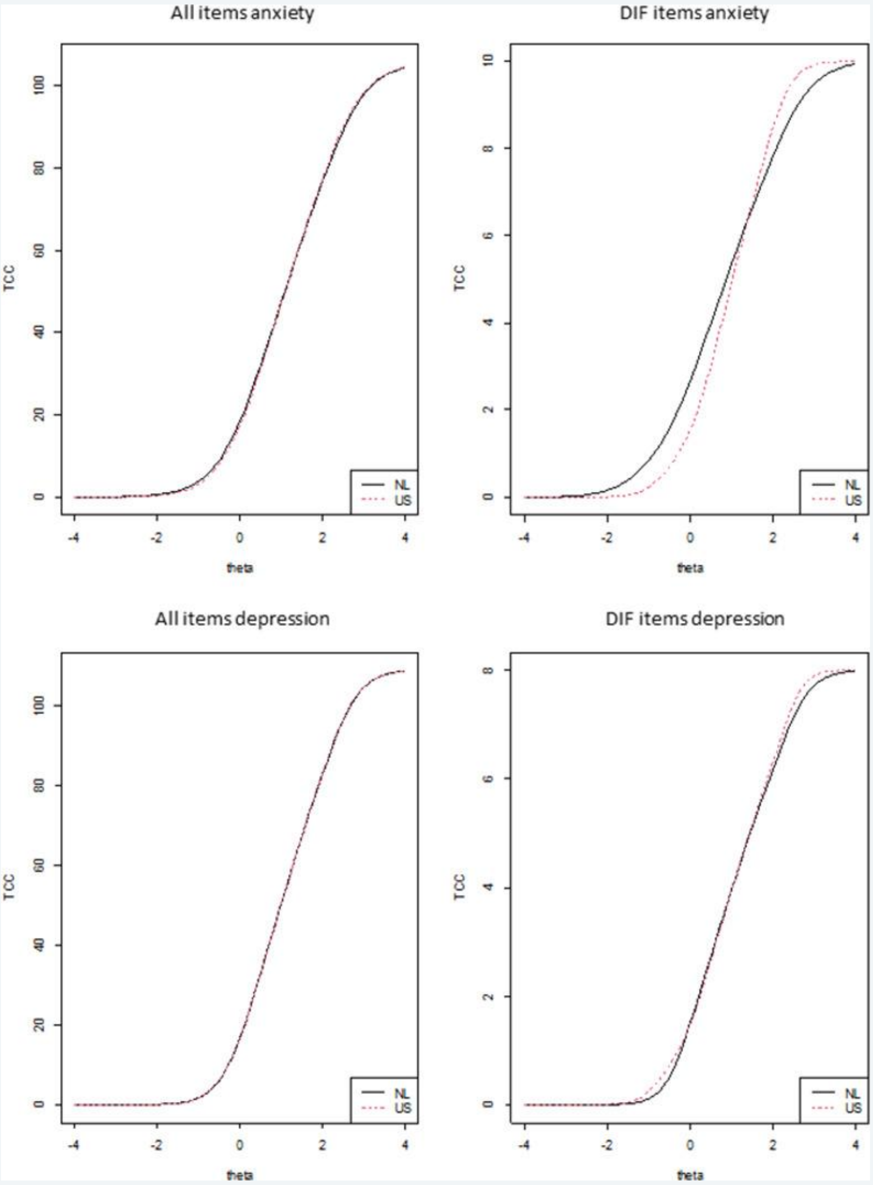

Figure 1. Total impact of DIF on the test characteristic curve (TCC) for anxiety and depression. [97]

Tradução: "A Tabela 2 mostra os resultados das análises de DIF. Dois itens do banco de itens de ansiedade, "Fiquei assustado quando me senti nervoso" (EDANX03) e "Senti-me preocupado" (EDANX30), apresentaram DIF uniforme e não uniforme, respectivamente. O item "Eu me senti preocupado" está presente no formulário curto do PROMIS anxiety 7a. Os itens estão presentes em 1 e 3% das avaliações baseadas no CAT, respectivamente. No banco de itens de depressão, dois itens apresentaram DIF uniforme: "Eu me senti inútil" (EDDEP04) e "Eu me senti infeliz" (EDDEP36). (...) Para o item 'Fiquei assustado quando me senti nervoso', os parâmetros de limiar para a população holandesa foram, em sua maioria, ligeiramente inferiores aos limiares para a população dos EUA, indicando que a população holandesa endossa categorias de resposta mais altas no mesmo nível de ansiedade. (...) A Figura 1 ilustra o impacto do DIF nas pontuações totais dos entrevistados. Os gráficos à esquerda mostram o impacto do DIF quando todos os itens são considerados, enquanto os gráficos à direita mostram o impacto do DIF quando apenas os itens DIF são considerados. Os gráficos mostram que o DIF teve um impacto mínimo na pontuação total quando todos os itens foram administrados em cada banco de itens."

Tabela 2. Pseudo R^2 de McFadden e parâmetros da TRI para itens que apresentam DIF

Banco de itens	Item com DIF	Tipo de DIF	Pseudo R^2 de McFadden	Inclinação; e parâmetros de limite	Incluído no CAT[d]
Ansiedade	EDANX03: Isso me assustava quando eu ficava nervoso	Uniforme	R^2_{12} = 0,021<br>R^2_{23} = 0,011	**NL:** 2,62; 0,15, 0,97, 2,02<br>US: 3,74; 0,59, 1,18, 1,95	1%
	EDANX30: Fiquei preocupado[a]	Não-uniform	R^2_{12} = 0,010<br>R^2_{23} = 0,033	NL: 2,16; -1,12, -0,10, 1,29, 2,64<br>US: 3,14; -0,57, 0,24, 1,22, 2,12	3%
Depressão	EDDEP04: Eu me senti inútil[b]	Uniforme	R^2_{12} = 0,024<br>R^2_{23} = 0,013	**NL:** 2,93; -0,17, 0,58, 1,56, 2,61<br>US: 4,37; 0,29, 0,88, 1,61, 2,36	8%
	EDDEP36: Eu me senti infeliz[c]	Uniforme	R^2_{12} = 0,037<br>R^2_{23} = 0,001	NL: 4,21; -0,14, 0,61, 1,33, 2,20<br>**US:** 3,44; -0,64, 0,23, 1,20, 2,17	100%

A população em negrito apresentou limiares mais baixos em comparação com a outra população, indicando que essa população endossa categorias de resposta de itens mais altas no mesmo nível do domínio (ansiedade, depressão)
[a] presente no formulário curto de ansiedade 7a
[b] presente no formulário abreviado de depressão 4a, 6a, 8a e 8b
[c] presente no formulário curto de depressão 6a, 8a e 8b
[d] Com base em 4047 avaliações baseadas em CAT para ansiedade e 4293 avaliações baseadas em CAT para depressão

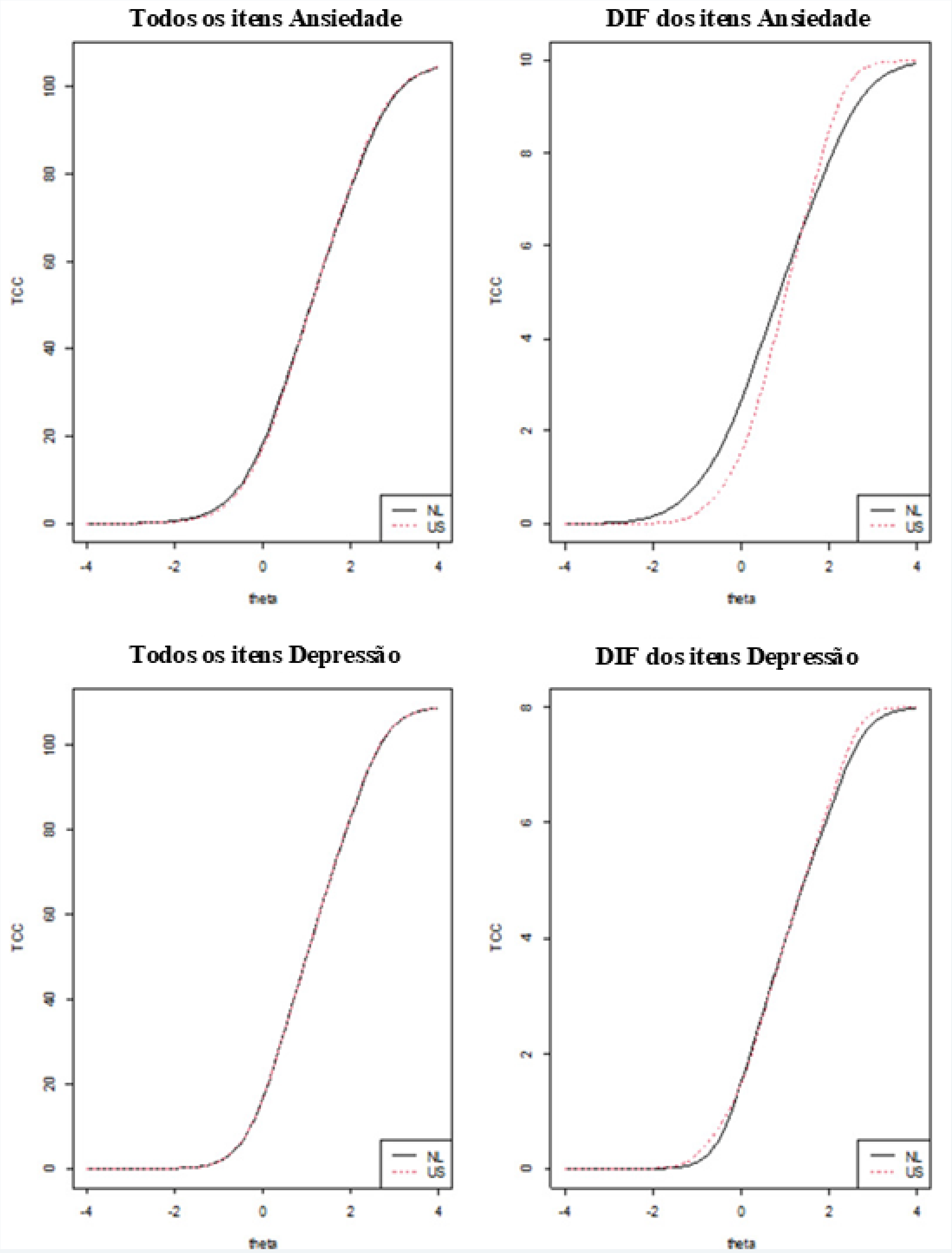

Figura 1. Impacto total do DIF na curva característica do teste (CCT) para ansiedade e depressão.

Confiabilidade

A confiabilidade refere-se à proporção da variação total nas medições que se deve às diferenças “verdadeiras” entre os pacientes. “Verdadeira” é a pontuação média que seria obtida se a escala fosse administrada um número infinito de vezes à mesma pessoa [92]. A confiabilidade também pode ser explicada como a capacidade de uma PROM de distinguir entre pacientes [92]. Dentro de um grupo homogêneo, é difícil distinguir entre os pacientes [98]. Uma suposição importante feita em um estudo de confiabilidade (e em um estudo sobre erro de medida) é que os pacientes são estáveis no construto a ser medido entre as medições repetidas.

Confiabilidade: Métodos

R1 - Estabilidade dos pacientes

Forneça argumentos para assumir a estabilidade dos pacientes no construto de interesse entre as medições repetidas.

Explicação: A confiabilidade é avaliada por medições repetidas em pacientes estáveis. O que são “pacientes estáveis” depende do construto a ser medido e da população-alvo. Você deve fornecer argumentos para presumir a estabilidade dos pacientes no construto de interesse entre as medições repetidas para garantir que a confiabilidade das medições repetidas não seja comprometida por mudanças no construto a ser medido. Os dados também podem ser coletados sobre intervenção(ões) relacionada(s) ao construto que está sendo medido no período intermediário (por exemplo, cirurgia, reabilitação, tratamento psicológico etc.). Se uma intervenção for realizada no período intermediário, pode-se presumir que (muitos dos) pacientes mudaram no construto a ser medido. Por exemplo, se você estivesse medindo o impacto da dor lombar na qualidade de vida, seus pacientes não se submeteriam à reabilitação para aliviar a dor; ou se você fosse medir a autoestima em relação ao corpo, as terapias psicológicas poderiam influenciar a estabilidade de seus pacientes.

Elementos essenciais

• Argumentos para presumir a estabilidade dos pacientes no construto de interesse entre as medições repetidas.

Exemplo 1

“Patients were excluded from the retest when they experienced a change in disease activity from active to quiescent disease or vice versa according to the Harvey-Bradshaw Index/Simple Clinical Colitis Activity Index (HBI/SCCAI).” [99]

Tradução: "Os pacientes foram excluídos do reteste quando apresentaram uma mudança na atividade da doença de ativa para quiescente ou vice-versa, de acordo com o *Harvey-Bradshaw Index/Simple Clinical Colitis Activity Index* (HBI/SCCAI)."

Exemplo 2

"The patients completed the questionnaire at two time points. (…) A long interval between test administrations increases the risk of change in patients' health status in a test-retest study, whereas a short interval increases the risk of recall bias [ref]. (…) Patients' mental health was assumed to be stable during the time period from test 1 to test 2, since the health status of epilepsy patients is not likely to change over a period of 2 weeks." [100]

Tradução: "Os pacientes preencheram o questionário em dois momentos. (...) Um longo intervalo entre as administrações de teste aumenta o risco de mudança no estado de saúde dos pacientes em um estudo de teste-reteste, enquanto um intervalo curto aumenta o risco de viés de recordação [ref]. (...) A saúde mental dos pacientes foi considerada estável durante o período de tempo entre o teste 1 e o teste 2, já que o estado de saúde dos pacientes com epilepsia provavelmente não mudará em um período de duas semanas."

Exemplo 3

"For the retest evaluation, a link was returned with the Female Genital Self-image Scale (FGSIS) and the following question: "Did you undergo treatment (surgery/physical therapy/medication on genitals) between the first and second assessments of this research?". The answer options were "yes" or "no." Only women who answered "no" to this question were included in the test-retest reliability." [42]

Tradução: "Para a avaliação do reteste, foi devolvido um link com a *Female Genital Self-image Scale* (FGSIS) e a seguinte pergunta: "Você se submeteu a tratamento (cirurgia/fisioterapia/medicação nos órgãos genitais) entre a primeira e a segunda avaliação desta pesquisa?". As opções de resposta eram "sim" ou "não". Somente as mulheres que responderam "não" a essa pergunta foram incluídas na confiabilidade teste-reteste."

R2 - Análises estatísticas

Forneça o coeficiente de correlação intraclasse/*Intraclass Correlation Coefficient* (ICC) específico ou fórmula kappa usada e justifique a escolha da fórmula usada.

Explicação: A estatística de confiabilidade preferida depende da unidade de medida da PROM. Para pontuações contínuas, é preferível um coeficiente de correlação intraclasse/*Intraclass Correlation Coefficient* (ICC) para avaliar a confiabilidade. Para pontuações ordinais, é preferível um kappa ponderado, para o qual o esquema de ponderação específico deve ser descrito [101]. Para uma pontuação dicotômica/nominal, é preferível um kappa não-ponderado. Para o ICC, o modelo específico deve ser descrito, como um modelo de efeitos aleatórios bidirecionais para concordância ($ICC_{concordância}$ ou $ICC_{(2.1)}$) [102], que leva em conta todos os erros de medida, inclusive a influência da fonte de variação de interesse (por exemplo, uma mudança sistemática entre os pontos de tempo em um delineamento de teste-reteste) [92]. Outro modelo poderia ser um modelo de efeitos mistos de duas vias para consistência ($ICC_{consistência}$ ou $ICC_{(3.1)}$) que não considera uma mudança sistemática entre as medições repetidas (por exemplo, a diferença média entre entrevistadores em um delineamento de confiabilidade entre entrevistadores). Se esse modelo for usado, você deve justificar por que não está interessado na diferença sistemática (ou mudança) da fonte de variação que variou entre as medições repetidas. Um terceiro modelo que poderia ser usado é um modelo de efeitos aleatórios unidirecionais ($ICC_{(1.1)}$) que leva em conta todos os erros [92], mas não investiga a influência específica de outra fonte de variação (por exemplo, ocasiões ou entrevistadores). Modelos mais complexos são descritos em outro lugar [92]. O coeficiente de correlação de Pearson ou Spearman sem evidências da ocorrência de mudanças sistemáticas ao longo do tempo pode superestimar a confiabilidade e não é recomendado porque a confiabilidade pode ser superestimada quando uma mudança sistemática não foi levada em conta.

Elementos essenciais

- Descrição do modelo ou esquema de ponderação para o ICC ou kappa específico usado.
- Justificativa para a escolha da fórmula utilizada.

Exemplo 1

"To investigate test-retest reliability of the Arm Function in Multiple Sclerosis Questionnaire (AMSQ), we calculated one-way ICC for the whole sample due to an incomplete design [ref] (research question 1). (...) In addition, we investigated whether there was a systematic difference between two measurements due to differences in mode of administration (research question 2), and whether there was a systematic difference between two measurements due to different observers (research question 3). These two questions were investigated by calculating ICC two-way ANOVA random effect models for agreement for patients who completed baseline and retest questionnaires in different ways of administration (i.e., paper at baseline and online at follow-up), and for patients who were interviewed. [ref]" [103]

Tradução: "Para investigar a confiabilidade teste-reteste do *Arm Function in Multiple Sclerosis Questionnaire* (AMSQ), calculamos o ICC unidirecional para toda a amostra devido a um projeto incompleto [ref] (pergunta de pesquisa 1). (...) Além disso, investigamos se havia uma diferença sistemática entre duas medições devido a diferenças no modo de administração (questão de pesquisa 2) e se havia uma diferença sistemática entre duas medições devido a diferentes observadores (questão de pesquisa 3). Essas duas perguntas foram investigadas por meio do cálculo de modelos de efeito aleatório de ANOVA de duas vias do ICC para concordância de pacientes que preencheram questionários de linha de base e de reteste em diferentes modos de administração (ou seja, papel na linha de base e on-line no acompanhamento) e para pacientes que foram entrevistados. [ref]"

Exemplo 2

"Test-retest reliability of single ordinal items was assessed using weighted kappa statistic with squared weights and corresponding 95% CI estimated using the bootstrap method (1000 replications)." [100]

Tradução: "A confiabilidade teste-reteste de itens ordinais únicos foi avaliada usando a estatística kappa ponderada com pesos ao quadrado e o IC95% correspondente estimado usando o método *bootstrap* (1.000 replicações)."

R3 - Fontes de variação

Especifique qual fonte de variação foi propositalmente variada entre as medições repetidas.

Explicação: Cada medição deve ser realizada sob as mesmas condições de medição, exceto pela condição (também chamada de fonte de variação) de interesse que foi intencionalmente variada entre as medições repetidas. As fontes de variação podem ser ocasiões, momentos do dia, diferentes entrevistadores, diferentes contextos ou ambientes, diferentes modos de administração, diferentes softwares etc. Você deve especificar qual fonte de variação é de seu interesse principal, a qual você variou propositadamente entre as medições repetidas em seu delineamento. Por exemplo, se a ocasião foi variada, você poderia indicar isso como "delineamento teste-reteste". Isso permite que os leitores do seu artigo entendam o planejamento usado no seu estudo.

Elementos essenciais

• Qual fonte de variação foi propositalmente variada.

Exemplo geral de delineamento teste-reteste

"Avaliamos a confiabilidade teste-reteste da PROM com pacientes que completaram a PROM na linha de base e 7 dias depois."

Exemplo geral de estudo de confiabilidade entre entrevistadores

"Dois entrevistadores diferentes administraram a PROM aos mesmos pacientes para avaliar a confiabilidade entre avaliadores."

Exemplo geral para diferentes ambientes

"Usamos um projeto de confiabilidade com medições repetidas em que os pacientes preencheram a PROM uma vez em casa e outra no hospital."

Exemplo 1

"Test-retest reliability and measurement error were assessed for women who did not modify complaints of dysmenorrhea by the Global Rating of Change Scale between 7 to 10 days." [104]

Tradução: "A confiabilidade teste-reteste e o erro de medida foram avaliados em mulheres que não modificaram as queixas de dismenorreia pela *Global Rating of Change Scale* entre 7 e 10 dias."

R4 - Intervalo de tempo

Forneça argumentos para a adequação do intervalo de tempo.

Explicação: O intervalo de tempo entre as medições repetidas deve ser longo o suficiente para evitar o viés de recordação e curto o suficiente para garantir que os pacientes não tenham sofrido alterações no construto a ser medido. Você deve apresentar argumentos para a adequação do intervalo de tempo para garantir que a confiabilidade das medições repetidas não seja comprometida pelo intervalo de tempo. O intervalo de tempo adequado depende do construto a ser medido e da população-alvo. Um intervalo de tempo de cerca de 2 semanas é geralmente considerado apropriado para a avaliação de PROMs [5]. Em geral, os argumentos para um intervalo de tempo adequado podem se basear em estudos anteriores ou na experiência de especialistas com o construto que está sendo medido. O intervalo de tempo em si foi descrito no item GM6.

Elementos essenciais

• O intervalo de tempo entre as medições repetidas.
• Argumentos para a adequação do intervalo de tempo.

Exemplo 1

"We set two weeks as the interval between the assessments based on previous research [ref], which also mentioned the clinical status is unlikely to change appreciably in the absence of intervention during this time." [105]

Tradução: "Definimos duas semanas como o intervalo entre as avaliações com base em pesquisas anteriores [ref], que também mencionaram que é improvável que o estado clínico mude consideravelmente na ausência de intervenção durante esse período."

Confiabilidade: Resultados

R5 - Estabilidade dos pacientes

Descreva se os pacientes permaneceram estáveis no construto de interesse entre as medições repetidas.

Explicação: As descrições da estabilidade do paciente são essenciais para garantir a credibilidade dos resultados em um estudo sobre a confiabilidade das medições repetidas. Na seção de resultados, você deve fornecer evidências de que seus pacientes realmente estavam estáveis no construto de interesse entre as medições repetidas. Além disso, você deve fornecer o número de pacientes estáveis e os motivos pelos quais os pacientes foram considerados instáveis. O número de pacientes estáveis é necessário para verificar se ele é consistente com o tamanho da amostra necessário para garantir o poder estatístico do seu estudo. Os motivos de instabilidade podem incluir qualquer intervenção relacionada ao construto que está sendo medido no período intermediário (por exemplo, cirurgia, reabilitação, tratamento psicológico etc.).

Elementos essenciais

- Evidencie se os pacientes estavam estáveis no construto de interesse entre as medições repetidas.
- Número de pacientes estáveis.
- Motivo da não estabilidade dos pacientes.

Exemplo 1

"In the total sample, 355 (57.82%) women returned the questionnaires between 14 and 20 days for retest, and 22 (6.20%) women were excluded for having undergone treatment on the genitals. Thus, test-retest reliability analysis was performed with 333 (93.80%) women." [42]

Tradução: "Na amostra total, 355 (57,82%) mulheres devolveram os questionários entre 14 e 20 dias para reteste, e 22 (6,20%) mulheres foram excluídas por terem sido submetidas a tratamento nos órgãos genitais. Assim, a análise de confiabilidade teste-reteste foi realizada com 333 (93,80%) mulheres."

R6 - Análises estatísticas

Forneça resultados para as estatísticas calculadas (ICC ou kappa) e medidas de precisão (erros padrão ou intervalos de confiança). Forneça resultados de todos os componentes de variância ou tabelas de contingência.

Explicação: Além de fornecer os parâmetros de confiabilidade da PROM em estudo, todos os componentes de variação devem ser relatados para os valores de ICC, bem como seu intervalo de confiança de 95%. Isso facilita a compreensão do impacto de diferentes fontes de variação na pontuação observada. Também facilita a comparação e o agrupamento futuro de resultados em revisões sistemáticas e permite que os leitores do seu artigo compreendam a precisão das estimativas de ICC [92]. Para os valores de kappa, recomendamos informar tabelas de contingência, além do próprio valor de kappa.

Elementos essenciais

- Resultados das estatísticas calculadas (ICC ou kappa).
- Resultados de todos os componentes de variância ou tabelas de contingência.
- Medidas de precisão: intervalos de confiança de 95%.

Exemplo 1

"The $ICC_{agreement}$ was 0.79 (95% CI 0.70-0.84) indicating sufficient test-retest reliability [for the Patient-Reported Outcomes Measurement Information System (PROMIS) short form designed to measure physical function in geriatric rehabilitation patients (PROMIS-PF-GR)]." [106]

Tradução: "O $ICC_{concordância}$ foi de 0,79 (IC95% 0,70-0,84), indicando confiabilidade teste-reteste suficiente [para o formulário curto do *Patient-Reported Outcomes Measurement Information System* (PROMIS) projetado para medir a função física em pacientes de reabilitação geriátrica (PROMIS-PF-GR)]."

Erro de medida

O erro de medida refere-se ao erro sistemático e aleatório da pontuação de um paciente individual que não é atribuído a mudanças verdadeiras no construto a ser medido. Ele se refere à precisão da pontuação [92]. O erro de medida (às vezes também chamado de concordância) é avaliado por medições repetidas em pacientes estáveis. Os mesmos dados coletados para avaliar a confiabilidade podem ser usados.

Erro de medida: Método

ME1 - Estabilidade dos pacientes

Forneça argumentos para assumir a estabilidade dos pacientes no construto de interesse entre as medições repetidas.

Explicação: Semelhante à confiabilidade, o erro de medida é avaliado por medições repetidas em pacientes estáveis (veja o item R1). Você deve fornecer argumentos para presumir a estabilidade dos pacientes no construto de interesse entre as medições repetidas.

Elementos essenciais

- Argumentos para presumir a estabilidade dos pacientes no construto de interesse entre as medições repetidas.

Exemplo 1

"Patients were excluded from the retest when they experienced a change in disease activity from active to quiescent disease or vice versa according to the Harvey-Bradshaw Index/Simple Clinical Colitis Activity Index (HBI/SCCAI)." [99]

Tradução: "Os pacientes foram excluídos do reteste quando apresentaram uma mudança na atividade da doença de ativa para quiescente ou vice-versa, de acordo com o *Harvey-Bradshaw Index/Simple Clinical Colitis Activity Index* (HBI/SCCAI)."

Exemplo 2

"The patients completed the questionnaire at two time points. (…) A long interval between test administrations increases the risk of change in patients' health status in a test-retest study, whereas a short interval increases the risk of recall bias [ref]. (…) Patients' mental health was assumed to be stable during the time period from test 1 to test 2, since the health status of epilepsy patients is not likely to change over a period of 2 weeks." [100]

Tradução: "Os pacientes preencheram o questionário em dois momentos. (...) Um longo intervalo entre as administrações de teste aumenta o risco de mudança no estado de saúde dos pacientes em um estudo de teste-reteste, enquanto um intervalo curto aumenta o risco de viés de recordação [ref]. (...) A saúde mental dos pacientes foi considerada estável durante o período de tempo entre o teste 1 e o teste 2, já que o estado de saúde dos pacientes com epilepsia provavelmente não mudará em um período de duas semanas."

Exemplo 3

"For the retest evaluation, a link was returned with the Female Genital Self-image Scale (FGSIS) and the following question: "Did you undergo treatment (surgery/physical therapy/medication on genitals) between the first and second assessments of this research?". The answer options were "yes" or "no." Only women who answered "no" to this question were included in the test-retest reliability." [42]

Tradução: "Para a avaliação do reteste, foi devolvido um link com a *Female Genital Self-image Scale* (FGSIS) e a seguinte pergunta: "Você se submeteu a tratamento (cirurgia/fisioterapia/medicação nos órgãos genitais) entre a primeira e a segunda avaliação desta pesquisa?". As opções de resposta eram "sim" ou "não". Somente as mulheres que responderam "não" a essa pergunta foram incluídas na confiabilidade teste-reteste."

ME2 - Análises estatísticas

Forneça e justifique a fórmula estatística específica usada para o erro padrão de medida/*Standard Error of Measurement* (SEM), menor mudança detectável/*Smallest Detectable Change* (SDC), limites de concordância/*Limits of Agreement* (LoA) ou porcentagem de concordância (específica).

Explicação: A estatística de erro de medida preferida depende da unidade de medição da PROM. Para pontuações contínuas, são preferíveis o erro padrão de medida/*Standard Error of Measurement* (SEM) para uma única pontuação e a menor mudança detectável/*Smallest Detectable Change* (SDC) - também chamada de mínima mudança detectável/*Minimal Detectable Change* (MDC) - para uma pontuação de alteração. Como alternativa, os limites de concordância/*Limits of Agreement* (LoA) podem ser calculados. Para pontuações dicotômicas, nominais ou ordinais, recomendamos o uso da concordância percentual (específica) para calcular o erro de medida da PROM. Você também deve especificar a fórmula de erro de medida usada para a estatística usada. Da mesma forma que os modelos ICC (veja o item R2), o modelo de SEM específico deve ser descrito (por exemplo, $SEM_{concordância}$) e sua fórmula [92]. Observe que o SDC derivado dos LoA é igual ao $SDC_{consistência}$. Além disso, se as medidas de precisão (por exemplo, intervalos de confiança de 95%) foram calculadas, recomendamos que você descreva quais métodos ou técnicas foram usados para calcular essas medidas.

Elementos essenciais

- O SEM, a SDC, os LoA ou a fórmula de concordância percentual (específica) usada.
- Justificativa para a escolha das fórmulas usadas.

Exemplo 1

"For measurement error, standard error of the measurement ($SEM_{agreement}$), smallest detectable change (SDC) at the individual level, and Bland and Altman graph were used. $SEM_{agreement}$ was estimated by the formula [$_{difference}SD/\sqrt{2}$], in which $_{difference}SD$ was the standard deviation (SD) of the difference between the test and retest score of the Female Genital Self-image Scale (FGSIS). SDC was estimated by [SEM*1.96*√2]. Bland and Altman graph was estimated by limits of agreement (LoA) using the formula [d-±(1.96*$_{difference}SD$)], in which d- is the mean of the differences between the test and retest of the FGSIS." [42]

Tradução: "Para o erro de medida, foram usados o erro padrão da medida/*Standard Error of the Measurement* ($SEM_{concordância}$), a menor mudança detectável/*Smallest Detectable Change* (SDC) em nível individual e o gráfico de Bland e Altman. O

$SEM_{concordância}$ foi estimado pela fórmula [$DP_{diferença}$/√2], em que $DP_{diferença}$ era o desvio padrão (DP) da diferença entre a pontuação do teste e do reteste da *Female Genital Self-image Scale* (FGSIS). O SDC foi estimado por [SEM*1,96*√2]. O gráfico de Bland e Altman foi estimado pelos limites de concordância/*Limits of Agreement* (LoA) usando a fórmula [d-±(1,96*$DP_{diferença}$)], em que d- é a média das diferenças entre o teste e o reteste da FGSIS."

ME3 - Fontes de variação

Especifique qual fonte de variação foi propositalmente variada entre as medições repetidas.

Explicação: Semelhante à confiabilidade, cada medição deve ser realizada sob as mesmas condições de medição, exceto pela condição (também chamada de fonte de variação) de interesse que foi intencionalmente variada entre as medições repetidas (veja o item R3). Recomendamos que você especifique em qual fonte de variação está mais interessado e qual variou propositadamente entre as medições repetidas em seu delineamento.

Elementos essenciais

• Qual fonte de variação foi propositalmente variada.

Exemplo geral de delineamento teste-reteste

"Avaliamos o erro de medida da PROM com pacientes que preencheram a PROM na linha de base e 7 dias depois."

Exemplo geral de estudo de erro de medida entre entrevistadores

"Dois entrevistadores diferentes administraram a PROM aos mesmos pacientes para avaliar a confiabilidade entre avaliadores."

Exemplo geral para diferentes ambientes

"Usamos um projeto de medidas repetidas em que os pacientes preencheram a PROM uma vez em casa e uma vez no hospital."

Exemplo 1

"Test-retest reliability and measurement error were assessed for women who did not modify complaints of dysmenorrhea by the Global Rating of Change Scale between 7 to 10 days." [104]

Tradução: "A confiabilidade teste-reteste e o erro de medida foram avaliados em mulheres que não modificaram as queixas de dismenorreia pela *Global Rating of Change Scale* entre 7 e 10 dias."

ME4 - Intervalo de tempo

Forneça argumentos sobre a adequação do intervalo de tempo.

Explicação: Da mesma forma que a confiabilidade, o intervalo de tempo deve ser longo o suficiente para evitar o viés de recordação e curto o suficiente para garantir que os pacientes não tenham sofrido alterações no construto a ser medido (veja o item R4). Recomendamos que você apresente argumentos para a adequação do intervalo de tempo.

Elementos essenciais

- O intervalo de tempo entre as medições repetidas.
- Argumentos para a adequação do intervalo de tempo.

Exemplo 1

"We set two weeks as the interval between the assessments based on previous research [ref], which also mentioned the clinical status is unlikely to change appreciably in the absence of intervention during this time." [105]

Tradução: "Definimos duas semanas como o intervalo entre as avaliações com base em pesquisas anteriores [ref], que também mencionaram que é improvável que o status clínico mude consideravelmente na ausência de intervenção durante esse período."

Erro de medida: Resultados

ME5 - Estabilidade dos pacientes

Descreva se os pacientes estavam estáveis no construto de interesse entre as medições repetidas.

Explicação: Da mesma forma que a confiabilidade, as descrições da estabilidade do paciente são essenciais para garantir a credibilidade dos resultados em um estudo sobre o erro de medida das medições repetidas (veja o item R5). Na seção de resultados, recomendamos que você descreva se os seus pacientes realmente estavam estáveis no construto de interesse entre as medições repetidas. Além disso, recomendamos que você forneça o número de pacientes estáveis e os motivos dos pacientes instáveis.

Elementos essenciais

• Descrição do fato de os pacientes terem se mantido estáveis no construto de interesse entre as medições repetidas.
• Número de pacientes estáveis.
• Motivo da não instabilidade dos pacientes

Exemplo 1

"In the total sample, 355 (57.82%) women returned the questionnaires between 14 and 20 days for retest, and 22 (6.20%) women were excluded for having undergone treatment on the genitals. Thus, test-retest reliability analysis was performed with 333 (93.80%) women." [42]

Tradução: "Na amostra total, 355 (57,82%) mulheres devolveram os questionários entre 14 e 20 dias para reteste, e 22 (6,20%) mulheres foram excluídas por terem sido submetidas a tratamento nos órgãos genitais. Assim, a análise de confiabilidade teste-reteste foi realizada com 333 (93,80%) mulheres."

ME6 - Análises estatísticas

Forneça resultados para estatísticas calculadas e medidas de precisão (se aplicável). Forneça resultados de todos os componentes de variância incluídos no SEM, forneça a mudança sistemática ou diferença entre as medições repetidas (quando LoA foi aplicado) ou tabelas de contingência.

Explicação: Além de informar os parâmetros de erro de medida da PROM (por exemplo, SEM, SDC, LoA ou porcentagem (específica) de concordância), recomendamos informar todos os componentes de variação nos valores de SEM e SDC, bem como o intervalo de confiança de 95% do SEM. Isso facilita a compreensão do impacto das diferentes fontes de variação sobre o erro de medida. Também facilita a comparação e o agrupamento de seus resultados em revisões sistemáticas e permite que os leitores do seu artigo entendam a precisão das estimativas de SEM [92]. Para pontuações de PROM dicotômicas/nominais/ordinais, recomendamos que você informe a concordância (específica) percentual expressa separadamente para cada categoria da pontuação (por exemplo, concordância positiva e negativa para uma pontuação dicotômica), bem como tabelas de contingência.

Elementos essenciais

- Resultados das estatísticas calculadas (SEM, SDC e/ou LoA, ou porcentagem de concordância).
- Resultados de todos os componentes de variância ou tabelas de contingência.
- Medidas de precisão: intervalos de confiança de 95%.

Exemplo 1

“The mean difference (d-) between the test and retest results was −0.285. $SEM_{agreement}$ and SDC at the individual level were 1.469 and 4.071, respectively. Figure 1 shows the Bland and Altman plot with the lower (−4.359) and upper (3.788) limits of agreement (LoA).” [42]

Tradução: “A diferença média (d-) entre os resultados do teste e do reteste foi de -0,285. O $SEM_{concordância}$ e o SDC em nível individual foram 1,469 e 4,071, respectivamente. A Figura 1 mostra o gráfico de Bland e Altman com os limites de concordância (LoA) inferior (-4,359) e superior (3,788).”

Exemplo 2

"For the measurement error, the percentage of agreement between test and retest was (...) (71.8%), and of disagreement was 28.2%." [45]

Tradução: "Para o erro de medida, a porcentagem de concordância entre o teste e o reteste foi (...) (71,8%), e a de discordância foi de 28,2%."

Validade de critério

A validade de critério refere-se ao grau em que as pontuações de uma PROM são um reflexo adequado de um 'padrão-ouro'. Um 'padrão-ouro' é uma referência ou critério amplamente aceito como o melhor método ou padrão disponível em relação ao qual outros métodos podem ser comparados ou avaliados [107]. Um 'padrão-ouro' de diagnóstico geralmente não é um 'padrão-ouro' apropriado para avaliar a validade de critério de uma PROM, pois a PROM em estudo geralmente mede outro construto que não o 'padrão-ouro' de diagnóstico. No primeiro estudo COSMIN, chegamos ao consenso de que não há 'padrão-ouro' para uma PROM (já que o próprio paciente é o 'padrão-ouro' para um desfecho relatado pelo paciente). Somente a versão longa de uma PROM abreviada pode ser considerada um 'padrão-ouro' [108]. Muitas vezes, o instrumento de comparação é considerado erroneamente como 'padrão-ouro' (por exemplo, quando as pontuações de um novo instrumento são comparadas a um instrumento amplamente utilizado, como o SF-36). Nesse caso, consideramos isso como um teste de hipóteses para a validade de construto, e as hipóteses esperadas sobre a magnitude e a direção da correlação entre (subescalas de) os instrumentos devem ser formuladas e testadas (por exemplo, ≥0,70).

CriV1 - Critério

Justifique a suposição de que o instrumento de comparação é um padrão-ouro aceitável.

Explicação: As PROMs não têm 'padrões-ouro', pois o próprio paciente é o 'padrão-ouro'. A única exceção é que a versão longa de uma PROM curta pode ser considerada o 'padrão-ouro' [1]. Nesse caso, há uma hipótese implícita de que se deve esperar uma correlação muito alta (≥0,90). Se o seu estudo for considerado uma avaliação da validade de critério, recomendamos que você justifique por que um instrumento é considerado o 'padrão-ouro' para o construto de interesse [109].

Elementos essenciais

• Justificativa para presumir que o comparador é um padrão-ouro apropriado.

Exemplo 1

"Our hypothesis was that there would be a more adequate Roland-Morris Disability Questionnaire (RMDQ) short version, and that this short version would be positively correlated with the original RMDQ. We assessed criterion validity and considered the 24-item long version of the RMDQ as the gold standard." [15]

Tradução: "Nossa hipótese era que haveria uma versão curta mais adequada do *Roland-Morris Disability Questionnaire* (RMDQ) e que essa versão curta estaria positivamente correlacionada com o RMDQ original. Avaliamos a validade de critério e consideramos a versão longa de 24 itens do RMDQ como o padrão-ouro."

Exemplo 2

"Criterion validity estimates how well one measure is correlated with a "gold standard". The long-version scores of the questionnaires [Pain Catastrophizing Scale and Tampa Scale for Kinesiophobia] were considered the "gold standard" and correlations with the short-version scores were calculated. Pearson correlation coefficients > 0.7 were expected." [110]

Tradução: "A validade de critério estima o grau de correlação de uma medida com um "padrão-ouro". As pontuações de versão longa dos questionários [*Pain Catastrophizing Scale e Tampa Scale for Kinesiophobia*] foram considerados o "padrão-ouro" e as correlações com as pontuações de versão curta foram calculadas. Esperavam-se coeficientes de correlação de Pearson > 0,7."

Validade de critério: Métodos

CriV2 - Análises estatísticas

Forneça e justifique as estatísticas usadas: correlações quando o critério tem pontuações contínuas ou área sob a curva *Receiver Operating Characteristic* (ROC)/*area under the ROC curve*, e sensibilidade e especificidade quando o critério é dicotômico.

Explicação: Recomendamos que você forneça e justifique as estatísticas usadas para avaliar a validade de critério. Quando a PROM em estudo tiver uma pontuação contínua, você poderá usar um coeficiente de correlação de Pearson ou Spearman quando o 'padrão-ouro' também for contínuo. Quando o 'padrão-ouro' for dicotômico, é possível calcular a área sob a curva *Receiver Operating Characteristic* (ROC)/*area under the* ROC *curve* [111].

Elementos essenciais

- As estatísticas usadas.
- Justifique as estatísticas usadas.

Exemplo 1

"(...) we used Spearman's correlation coefficient (rho) to correlate the long and short versions, given that the data did not present a normal distribution when analyzed using the Kolmogorov-Smirnov test. Correlation magnitude > 0.70 was considered the appropriate cut-off point for criterion validity [ref]." [15]

Tradução: "(...) usamos o coeficiente de correlação de Spearman (rho) para correlacionar as versões longa e curta, uma vez que os dados não apresentaram uma distribuição normal quando analisados por meio do teste de Kolmogorov-Smirnov. A magnitude da correlação > 0,70 foi considerada o ponto de corte apropriado para a validade do critério [ref]."

Exemplo 2

"For the PROMIS Anxiety Computer Adaptive Test (PROMIS-A-CAT) and PROMIS Anxiety Short Form (PROMIS-A-SF), we calculated sensitivity, specificity, negative predictive value (NPV) and positive predictive value (PPV) at the T score cut-points for mild (55) and moderate (65) anxiety proposed by (...). Too few people were classified with severe anxiety to warrant analysis. To examine cut-points we used Youden's Index, a commonly used summary measure of sensitivity and specificity [ref] ranging from zero to

one, where one represents perfect performance. Youden's index assumes equal importance of sensitivity and specificity and therefore may not be optimal for anxiety screening programs [ref], which might give higher weight to sensitivity, so that cases are not missed. Nevertheless, we used it to allow comparison with previous studies [ref]." [112]

Tradução: "Para o PROMIS *Anxiety Computer Adaptive Test* (PROMIS-A-CAT) e o PROMIS *Anxiety Short Form* (PROMIS-A-SF), calculamos a sensibilidade, a especificidade, o valor preditivo negativo (VPN) e o valor preditivo positivo (VPP) nos pontos de corte do *T-score* para ansiedade leve (55) e moderada (65) propostos por (...). Poucas pessoas foram classificadas com ansiedade grave para justificar a análise. Para examinar os pontos de corte, usamos o Índice de Youden, uma medida resumida de sensibilidade e especificidade [ref] comumente usada, que varia de zero a um, no qual um representa um desempenho perfeito. O índice de Youden pressupõe a mesma importância da sensibilidade e da especificidade e, portanto, pode não ser ideal para programas de triagem de ansiedade [ref], que podem valorizar mais a sensibilidade, para que os casos não sejam perdidos. No entanto, nós o usamos para permitir a comparação com estudos anteriores [ref]."

Validade de critério: Resultados

CriV3 - Análises estatísticas

Forneça resultados para as estatísticas calculadas.

Explicação: No estudo da validade de critério, recomendamos que você forneça os resultados das estatísticas calculadas (veja o item CriV2). Quando a análise de correlação for realizada entre a pontuação da PROM e o 'padrão-ouro', a direção (positiva ou negativa) e a magnitude do coeficiente de correlação de Pearson ou Spearman devem ser fornecidas. Ao realizar uma análise da área sob a curva ROC entre a pontuação da PROM e o padrão-ouro, é essencial informar o valor da área sob a curva ROC/*area under the* ROC *curve* e seu intervalo de confiança (IC), bem como a sensibilidade (taxa positiva verdadeira) e a especificidade (taxa negativa verdadeira) em diferentes pontos de corte. Você também pode incluir o gráfico da curva ROC em seu artigo para ilustrar a troca entre sensibilidade e especificidade em diferentes pontos de corte. Esses resultados melhoram a compreensão dos usuários finais do seu artigo sobre o desempenho do instrumento em comparação com o 'padrão-ouro' estabelecido.

Elementos essenciais

• Resultados para estatísticas calculadas.

Exemplo 1

"Area under the curve (AUC) were in the 'good' range for the PROMIS Anxiety Computer Adaptive Test (0.82, 95%CI = 0.73-0.90) and PROMIS Anxiety Short Form (0.80, 95%CI = 0.71-0.89) (Table2). The AUC for the Generalised Anxiety Disorder-7 (0.84) was also in the good range while the AUC for all other legacy measures fell into the 'useful' range (0.72 to 0.79)."

Table 2. Area under the curve (AUC) for each measure versus diagnosis of any anxiety disorder using the structured clinical interview for DMS disorders (SCID) [DSM-IV-TR Axis I Disorders]

Measure	AUC (95% CI)
PROMIS-A-CAT T score	.82 (.73 - .90)
PROMIS-A-SF T score	.80 (.71 - .89)
GAD-7	.84 (.77 - .91)
HADS-A	.79 (.70 - .89)
PSYCH-6	.79 (.70 - .89)
DASS-S	.77 (.68 - .86)
DASS-A	.72 (.62 - .82)
DT	.72 (.62 - .82)

n = 122 with all measures

[112]

Tradução: “A área sob a curva (AUC) estava na faixa 'boa' para o PROMIS Anxiety Computer Adaptive Test (0,82, IC95% = 0,73-0,90) e o PROMIS Anxiety Short Form (0,80, IC95% = 0,71-0,89) (Tabela 2). A AUC para o Transtorno de Ansiedade Generalizada-7 (0,84) também ficou na faixa boa, enquanto a AUC para todas as outras medidas herdadas ficou na faixa 'útil' (0,72 a 0,79).”

Tabela 2. Área sob a curva (AUC) para cada medida versus diagnóstico de qualquer transtorno de ansiedade usando a entrevista clínica estruturada para transtornos de DMS (SCID) [DSM-IV-TR Axis I *Disorders*]

Medidas	AUC (IC95%)
T-score do PROMIS-A-CAT	,82 (,73 - ,90)
T-score do PROMIS-A-SF	,80 (,71 - ,89)
GAD-7	,84 (,77 - ,91)
HADS-A	,79 (,70 - ,89)
PSYCH-6	,79 (,70 - ,89)
DASS-S	,77 (,68 - ,86)
DASS-A	,72 (,62 - ,82)
DT	,72 (,62 - ,82)

n = 122 com todas as medidas

Exemplo 2

“Regarding criterion validity, the correlation between the Roland-Morris Disability Questionnaire (RMDQ) with 24 items and 15 items is adequate (rho = 0.954, p < 0.001).” [15]

Tradução: “Com relação à validade de critério, a correlação entre o *Roland-Morris Disability Questionnaire* (RMDQ) com 24 itens e 15 itens é adequada (rho = 0,954, p < 0,001).”

Teste de hipóteses para validade de construto

O teste de hipóteses para validade de construto refere-se ao grau em que as pontuações de uma PROM são consistentes com as hipóteses baseadas na suposição de que a PROM mede validamente o(s) construto(s) a ser(em) medido(s). Muitos tipos de hipóteses podem ser testados para avaliar a validade de construto de uma PROM. Em geral, essas hipóteses se referem a uma relação esperada entre a pontuação transversal da PROM em estudo e a pontuação transversal de outro(s) instrumento(s) de comparação bem definido(s) e de alta qualidade, ou a diferenças esperadas nas pontuações entre subgrupos (também chamadas de validade de grupos "conhecidos").

Teste de hipóteses para validade de construto: Métodos

ConV1 - Hipóteses

Formule hipóteses e forneça justificativas para cada hipótese.

Explicação: Muitos tipos de hipóteses podem ser testados para avaliar a validade de construto de uma PROM. As hipóteses devem incluir tanto a direção (positiva ou negativa) quanto a magnitude (absoluta ou relativa) das correlações esperadas ou as diferenças nas pontuações [109]. As hipóteses podem ser formuladas em torno de correlações esperadas entre a PROM em estudo e outro instrumento de desfecho, ou diferenças nas pontuações da PROM entre grupos. Recomendamos que você forneça todas as hipóteses, incluindo a direção e a magnitude esperadas das correlações entre as pontuações da PROM e as pontuações dos instrumentos de comparação; ou a direção e a magnitude esperadas das diferenças nas pontuações da PROM entre os grupos. Por exemplo, se uma PROM tem como objetivo medir a participação social, pode-se esperar uma correlação moderada (mas não alta) com uma PROM que tenha como objetivo medir as limitações de atividade, pois esse é um construto diferente; ou pode-se esperar que os pacientes crônicos obtenham uma média de 10 pontos a mais na PROM em estudo para a intensidade da dor no joelho do que os pacientes agudos. Sem essa especificação, é difícil decidir posteriormente se os resultados estão de acordo com a hipótese [109]. Também recomendamos que você explique por que escolheu suas hipóteses, que podem ser baseadas na literatura (teoria e evidência empírica) e/ou em sua experiência (clínica/pesquisa) [109]. Isso permite que os leitores de seu artigo interpretem seus resultados. Quanto mais específicas forem as hipóteses e quanto mais hipóteses estiverem sendo testadas, mais evidências serão reunidas para a validade de construto.

Elementos essenciais

• Hipóteses, incluindo a direção e a magnitude esperadas das correlações entre as pontuações da PROM e as pontuações dos instrumentos de comparação (se aplicável).
• Hipóteses, incluindo a direção e a magnitude esperadas das diferenças nas pontuações da PROM entre os grupos (se aplicável).
• Justificativas para as hipóteses.

Exemplo 1

"Because Celiac Disease Quality of Life Survey (CD-QOL) items assess social limitations, emotional concerns, and cognitive concerns rather than physical aspects, we hypothesized moderate (r = 0.40–0.69) negative correlations between CD-QOL total and SF-36 social functioning, emotional well-being, and general health subscale scores, and small (r = 0.00–0.39) negative correlations between CD-QOL total and SF-36 physical functioning, role limitations due to physical and emotional problems, energy/fatigue, and bodily pain, such that worse Celiac disease-specific quality of life would be related to worse generic quality of life." [113]

Tradução: "Como os itens do *Celiac Disease Quality of Life Survey* (CD-QOL) avaliam limitações sociais, preocupações emocionais e preocupações cognitivas em vez de aspectos físicos, nossa hipótese era de correlações negativas e moderadas (r = 0,40-0,69) entre o CD-QOL total e as pontuações das subescalas de funcionalidade social, bem-estar emocional e saúde geral do SF-36, e correlações negativas e pequenas (r = 0. 00-0,39) entre o CD-QOL total e o SF-36, funcionalidade física, limitações de função devido a problemas físicos e emocionais, energia/fadiga e dor corporal, de modo que uma pior qualidade de vida específica da doença celíaca estaria relacionada a uma pior qualidade de vida genérica."

Exemplo 2

"With respect to construct validity, known-group validity was assessed for groups that were expected to differ in score: groups differing in age (three age groups were compared), gender, and chronic diseases (yes/no) were evaluated. The expected direction and magnitude of the differences were based on previous research on other Dutch adults on the same domains [ref]. Furthermore, Pearson correlations between each of the domains and the pain intensity item were calculated. The magnitude and direction of the expected correlation was based on previous knowledge on and

experience with the measured constructs. In total, 88 a priori hypotheses were formulated (see Table 6).”

Table 6. Confirmation of priori hypothesis regarding the expected difference between groups and the correlation domains of the PROMIS-89

Hypotheses	Number confirmed
Older participants score better than younger participants on the domains fatigue, sleep disturbance, anxiety and depression; a difference of at least 1 point is expected between each adjacent age group (18–39, 40-64, ≥ 65)[a]	2/8
Younger participants score better than older participants on the domains physical function and pain interference and on pain intensity; a difference of at least 1 point is expected between each adjacent age group (18–39, 40-64, ≥ 65) for physical function and pain interference, and a difference of at least 0.5 point for pain intensity[a]	5/6
The youngest (18–39) and oldest (≥ 65) age group score at least 1 point better than the middle age group (40-64) on the domains ability to participate in social roles and activities[a]	0/2
Males score at least 1 point better than females on all domains, and 0.5 point better on pain intensity[a]	7/8
Participants without chronic diseases score at least 2 points better than people with chronic diseases on all domains, and 1 point better on pain intensity[a]	5/8
The following domains have a correlation between 0.6 and 0.7: physical function and pain interference (negative), physical function and pain intensity (negative), anxiety and depression, sleep disturbance and fatigue	4/8
The domains ability to participate in social roles and activities and physical function have a correlation between 0.4 and 0.6	2/2
The domain pain interference has a correlation of at least 0.6 with pain intensity	2/2
The remaining domains have a correlation of less than 0.5 (depending on the direction, this might be negative)	42/44
Total	69/88 (78%)
Physical function	10/11 (91%)
Ability to participate in social roles and activities	8/11 (73%)
Anxiety	8/11 (73%)
Depression	8/11 (73%)
Fatigue	9/11 (82%)
Sleep disturbance	7/11 (64%)
Pain interference	11/11 (100%)
Pain intensity	8/11 (73%)

[a]Better means higher T-scores for the domains physical function and ability to participate in social roles and activities, and lower T-scores for the domains anxiety, depression, fatigue, sleep disturbance, pain interference; for pain intensity, better means a lower score on the 0-10 numeric scale
[23]

Tradução: “Com relação à validade de construto, a validade de grupo conhecido foi avaliada para grupos que se esperava apresentarem diferenças na pontuação: foram avaliados grupos que diferiam em idade (três grupos etários foram comparados), gênero e doenças crônicas (sim/não). A direção e a magnitude esperadas das diferenças foram baseadas em pesquisas anteriores com outros adultos holandeses nos mesmos domínios [ref]. Além disso, foram calculadas as correlações de Pearson entre cada um dos domínios e o item de intensidade da dor. A magnitude e a direção da correlação

esperada foram baseadas no conhecimento prévio sobre e experiência com os construtos medidos. No total, foram formuladas 88 hipóteses a priori (veja a Tabela 6).”

Tabela 6. Confirmação da hipótese a priori com relação à diferença esperada entre os grupos e os domínios de correlação do PROMIS-89

Hipóteses	Número confirmado
Os participantes mais velhos pontuam melhor do que os mais jovens nos domínios fadiga, distúrbios do sono, ansiedade e depressão; espera-se uma diferença de pelo menos 1 ponto entre cada faixa etária adjacente (18-39, 40-64, ≥ 65)[a]	2/8
Os participantes mais jovens pontuam melhor do que os mais velhos nos domínios função física e interferência da dor e na intensidade da dor; espera-se uma diferença de pelo menos 1 ponto entre cada grupo etário adjacente (18-39, 40-64, ≥ 65) para função física e interferência da dor, e uma diferença de pelo menos 0,5 ponto para intensidade da dor[a]	5/6
A faixa etária mais jovem (18-39) e a mais velha (≥ 65) pontuam pelo menos 1 ponto a mais do que a faixa etária intermediária (40-64) nos domínios capacidade de participar de papéis e atividades sociais[a]	0/2
Os homens pontuam pelo menos 1 ponto a mais do que as mulheres em todos os domínios e 0,5 ponto a mais na intensidade da dor[a]	7/8
Os participantes sem doenças crônicas pontuam pelo menos 2 pontos a mais do que as pessoas com doenças crônicas em todos os domínios, e 1 ponto a mais na intensidade da dor[a]	5/8
Os seguintes domínios têm uma correlação entre 0,6 e 0,7: função física e interferência da dor (negativa), função física e intensidade da dor (negativa), ansiedade e depressão, distúrbios do sono e fadiga	4/8
Os domínios capacidade de participar de papéis e atividades sociais e função física têm uma correlação entre 0,4 e 0,6	2/2
O domínio interferência da dor tem uma correlação de pelo menos 0,6 com a intensidade da dor	2/2
Os domínios restantes têm uma correlação de menos de 0,5 (dependendo da direção, isso pode ser negativo)	42/44
Total	69/88 (78%)
Função física	10/11 (91%)
Capacidade de participar de papéis e atividades sociais	8/11 (73%)
Ansiedade	8/11 (73%)
Depressão	8/11 (73%)
Fadiga	9/11 (82%)
Distúrbios do sono	7/11 (64%)
Interferência na dor	11/11 (100%)
Intensidade da dor	8/11 (73%)

[a]Melhor significa *T-scores* mais altas para os domínios função física e capacidade de participar de funções e atividades sociais, e pontuações T mais baixas para os domínios ansiedade, depressão, fadiga, distúrbios do sono, interferência da dor; para intensidade da dor, melhor significa uma pontuação mais baixa na escala numérica de 0 a 10

ConV2 - Análises estatísticas

Especifique todos os métodos estatísticos usados para testar as hipóteses.

Explicação: Para permitir que os leitores do seu artigo entendam claramente seus resultados, recomendamos que você especifique os métodos estatísticos usados para testar suas hipóteses. Se comparar a pontuação da PROM em estudo com outros instrumentos, recomendamos que calcule a correlação entre os dois instrumentos [109]; se comparar subgrupos, forneça informações descritivas dos dois grupos (por exemplo, pontuação média da PROM em ambos os grupos).

Elementos essenciais

• Métodos estatísticos ou descritivos usados para testar as hipóteses.

Exemplo 1

"Convergent validity was assessed by computing Spearman's rho correlation coefficients for Celiac Disease Quality of Life Survey (CD-QOL) total and subscale scores and scores on the SF-36 scales, (…). Coefficients r = 0.00-0.39 were considered small, r = 0.40-0.69 were considered moderate, and r = 0.70-1.00 were considered large. Because CD-QOL items assess social limitations, emotional concerns, and cognitive concerns rather than physical aspects, we hypothesized moderate negative correlations between CD-QOL total and SF-36 social functioning, emotional well-being, and general health subscale scores, and small negative correlations between CD-QOL total and SF-36 physical functioning, role limitations due to physical and emotional problems, energy/fatigue, and bodily pain, such that worse Celiac disease-specific quality of life would be related to worse generic quality of life." [113]

Tradução: "A validade convergente foi avaliada calculando-se os coeficientes de correlação rho de Spearman para as pontuações totais e de subescala do *Celiac Disease Quality of Life Survey* (CD-QOL) e as pontuações das escalas SF-36, (...). Coeficientes r = 0,00-0,39 foram considerados pequenos, r = 0,40-0,69 foram considerados moderados e r = 0,70-1,00 foram considerados grandes. Como os itens do CD-QOL avaliam limitações sociais, preocupações emocionais e preocupações cognitivas em vez de aspectos físicos, levantamos a hipótese de correlações negativas moderadas entre o CD-QOL total e as pontuações das subescalas de funcionamento social, bem-estar emocional e saúde geral do SF-36, e pequenas correlações negativas entre o CD-QOL total e o SF-36 de funcionalidade física, limitações de função devido a problemas físicos e emocionais, energia/fadiga e dor corporal, de modo que uma pior

qualidade de vida específica da doença celíaca estaria relacionada a uma pior qualidade de vida genérica."

Exemplo 2

"To determine construct validity, we assessed the difference in T-scores between groups, testing four hypotheses about the ability of the PROMIS item banks to distinguish between these groups. In line with COSMIN guidelines, construct validity is considered sufficient when ≥75% of the results are in accordance with the hypotheses (...). We expected PROMIS T-scores to be higher (worse) in (a) the sleep-clinic sample compared with the high-school sample, (b) the adolescents with a high risk of sleep problems compared with healthy high school students, and (c) high-school students with health issues compared with healthy high-school students. (...) A mean difference of ≥2 points, with a higher (worse) score in the clinical or health issues sample, was considered clinically relevant (...). The fourth hypothesis was that T-scores would worsen (increase) with more problems (higher response category) on item Sleep20 (I had a sleep problem). This item is included in the original PROMIS Sleep Disturbance item bank, but not in the Sleep Disturbance-23. For the fourth hypothesis we merged the last two response categories ('Quite a bit' and 'Very much') because the separate groups were too small. Differences in T-scores between groups were evaluated using linear regression analysis, with correction for relevant demographic variables based on the results from the analyses of the mean values." [35]

Tradução: "Para determinar a validade de construto, avaliamos a diferença nas *T-scores* entre os grupos, testando quatro hipóteses sobre a capacidade dos bancos de itens do PROMIS de distinguir entre esses grupos. De acordo com as diretrizes do COSMIN, a validade do construto é considerada suficiente quando ≥75% dos resultados estão de acordo com as hipóteses (...). Esperávamos que as *T-scores* do PROMIS fossem mais altas (piores) (a) na amostra da clínica do sono em comparação com a amostra do ensino médio, (b) nos adolescentes com alto risco de problemas de sono em comparação com alunos saudáveis do ensino médio e (c) nos alunos do ensino médio com problemas de saúde em comparação com alunos saudáveis do ensino médio. (...) Uma diferença média de ≥2 pontos, com uma pontuação maior (pior) na amostra de problemas clínicos ou de saúde, foi considerada clinicamente relevante (...). A quarta hipótese era que as *T-scores* piorariam (aumentariam) com mais problemas (categoria de resposta mais alta) no item Sleep20 (Tive um problema de sono). Esse item está incluído no banco de itens original do PROMIS *Sleep Disturbance*, mas não no *Sleep Disturbance*-23. Para a quarta hipótese, mesclamos as duas últimas categorias de resposta ("Um pouco" e "Muito") porque os grupos separados eram muito pequenos. As

diferenças nas *T-scores* entre os grupos foram avaliadas por meio de análise de regressão linear, com correção para variáveis demográficas relevantes com base nos resultados das análises dos valores médios."

Teste de hipóteses para validade de construto: Resultados

ConV3 - Análises estatísticas

Forneça todos os resultados e especifique se cada resultado está de acordo com sua hipótese.

Explicação: Para ajudar os leitores a interpretar os resultados de acordo com suas hipóteses, recomendamos que você forneça todos os resultados e especifique se cada resultado está de acordo com a respectiva hipótese. Recomendamos também que você informe cada resultado na forma de um valor numérico para a direção e a magnitude de uma correlação ou diferença média (por exemplo, r = -0,49, DM = 2,55) ou em uma tabela (por exemplo, destacando os resultados que estão de acordo com as hipóteses em negrito). Recomendamos que você ignore os valores de p das correlações e as pontuações médias de mudança porque não é relevante testar se as correlações ou as diferenças médias são estatisticamente diferentes de zero. Em vez disso, a validade diz respeito a se a direção e a magnitude de uma correlação ou diferença média são semelhantes ao que seria esperado com base no(s) construto(s) que está(ão) sendo medido(s).

Elementos essenciais

- Todos os resultados das análises de validade do construto.
- Indique se cada resultado está de acordo com sua hipótese.

Exemplo 1

"(…) we hypothesized moderate negative correlations between CD-QOL [Celiac Disease Quality of Life Survey] total and SF-36 social functioning, emotional well-being, and general health subscale scores, and small negative correlations between CD-QOL total and SF-36 physical functioning, role limitations due to physical and emotional problems, energy/fatigue, and bodily pain, such that worse CeD-specific quality of life would be related to worse generic quality of life. (…) correlations between CD-QOL total and SF-36 social functioning, emotional well-being, and general health and scores were moderate (r = 0.40-0.69) and correlations between CD-QOL total and SF-36 physical functioning, role limitations, and bodily pain were small (r = 0.00-0.39), as expected (...)" [113]

Tradução: "(...) nossa hipótese era de que haveria correlações negativas moderadas entre o CD-QOL [*Celiac Disease Quality of Life Survey*] total e as pontuações das subescalas de funcionalidade social, bem-estar emocional e saúde geral do SF-36, e pequenas correlações negativas entre o CD-QOL total e o SF-36 de funcionalidade física, limitações de função devido a problemas físicos e emocionais, energia/fadiga e

dor corporal, de modo que uma pior qualidade de vida específica da doença celíaca estaria relacionada a uma pior qualidade de vida genérica. (...) as correlações entre o CD-QOL total e a funcionalidade social, o bem-estar emocional e a saúde geral e as pontuações do SF-36 foram moderadas (r = 0,40-0,69) e as correlações entre o CD-QOL total e a funcionalidade física, as limitações de função e a dor corporal do SF-36 foram pequenas (r = 0,00-0,39), conforme esperado (...)"

Exemplo 2

"For both the Sleep Disturbance-23 and the Sleep-Related Impairment-11 all results were in accordance with the hypotheses (Table 4). The Sleep Disturbance-23 showed differences between the different samples of 2.7 to 10.6 points, and the largest difference was found between the high-school sample and the sleep-clinic sample. Additionally, adolescents who reported having more sleep problems on the single item also had worse T-scores. The Sleep-Related Impairment-11 showed differences between the different healthy and non-healthy samples of 4.0 to 8.6 points, corrected for age and gender. Here also, the largest difference was found between the high-school sample and the sleep-clinic sample. Adolescents who reported having more sleep problems on the single item also had worse T-scores."

Table 4. Hypothesis testing Sleep Disturbance-23 and Sleep-related Impairment-11

	**Mean difference in T-score (95% confidence interval)**[a]	
**We expected that:**	**Sleep Disturbance−23**	**Sleep-related Impairment−11**[b]
1. The sleep-clinic sample had higher scores than the high-school students	10.6 (8.1-13.1)	8.6 (5.2-11.9)
2. The adolescents with sleep problems and/or relevant health issues had higher scores than healthy high-school students	4.8 (3.5-6.2)	5.3 (3.5-7.1)
3. The high-school students with relevant health issues had higher scores than healthy high-school students	2.7 (1.2-4.2)	4.0 (2.0-6.0)
4. Adolescents who answered item Sleep20 'I had a sleep problem' with a higher response category, would have higher scores		
'Not at all' versus 'A little bit'	6.1 (5.1-7.1)	4.5 (3.0-6.0)
'A little bit' versus 'Somewhat'	2.2 (0.9-3.4)	3.9 (1.7-6.1)
'Somewhat' versus 'Quite a bit/very much'	5.2 (4.0-6.5)	3.2 (0.6-5.7)

[a]A mean difference of ≥2 points was considered clinically relevant.

[b]Corrected for age and gender.

[35]

Tradução: "Tanto para o *Sleep Disturbance*-23 quanto para o *Sleep-Related Impairment*-11, todos os resultados estavam de acordo com as hipóteses (Tabela 4). O *Sleep*

Disturbance-23 mostrou diferenças entre as diferentes amostras de 2,7 a 10,6 pontos, e a maior diferença foi encontrada entre a amostra do ensino médio e a amostra da clínica do sono. Além disso, os adolescentes que relataram ter mais problemas de sono no item único também tiveram *T-scores* piores. O *Sleep-Related Impairment*-11 mostrou diferenças entre as diferentes amostras saudáveis e não saudáveis de 4,0 a 8,6 pontos, corrigidas para idade e gênero. Aqui também, a maior diferença foi encontrada entre a amostra da escola secundária e a amostra da clínica do sono. Os adolescentes que relataram ter mais problemas de sono em um único item também tiveram *T-scores* piores."

Tabela 4. Teste de hipóteses *Sleep Disturbance*-23 e *Sleep-related Impairment*-11

	**Diferença média no *T-scores* (intervalo de confiança de 95%)[a]**	
**Nós esperávamos que:**	***Sleep Disturbance*–23**	***Sleep-related Impairment*–11[b]**
1. A amostra da clínica de sono tivesse pontuações mais altas do que os alunos do ensino médio	10,6 (8,1-13,1)	8,6 (5,2-11,9)
2. Os adolescentes com problemas de sono e/ou problemas de saúde relevantes tivessem pontuações mais altas do que os estudantes saudáveis do ensino médio	4,8 (3,5-6,2)	5,3 (3,5-7,1)
3. Os alunos do ensino médio com problemas de saúde relevantes tivessem pontuações mais altas do que os alunos saudáveis do ensino médio	2,7 (1,2-4,2)	4,0 (2,0-6,0)
4. Os adolescentes que respondessem ao item Sleep20 "Eu tive um problema de sono" com uma categoria de resposta mais alta teriam pontuações mais altas		
'Nem um pouco' versus 'Um pouco'	6,1 (5,1-7,1)	4,5 (3,0-6,0)
'Um pouco' versus 'Um pouco'	2,2 (0,9-3,4)	3,9 (1,7-6,1)
'Um pouco' versus 'Um pouco/muito'	5,2 (4,0-6,5)	3,2 (0,6-5,7)

[a]Uma diferença média de ≥2 pontos foi considerada clinicamente relevante.

[b]Corrigido para idade e gênero.

Responsividade

A responsividade refere-se à capacidade de uma PROM de detectar mudanças ao longo do tempo no construto a ser medido. Embora a responsividade seja considerada uma propriedade de medida separada da validade, a única diferença entre a validade (construto e critério) e a responsividade é que a validade se refere à validade de uma única pontuação (transversal) e a responsividade se refere à validade de uma pontuação de mudança (validade longitudinal) [114]. Portanto, os padrões de responsividade são semelhantes aos padrões de validade de construto e critério. A abordagem de critério para responsividade diz respeito a uma comparação entre a pontuação de mudança da PROM e a pontuação de mudança do padrão-ouro. É possível distinguir três tipos de hipóteses para usar a abordagem de construto para avaliar a responsividade de um instrumento: (1) hipóteses sobre a direção e a magnitude esperadas das correlações entre as pontuações de mudança na PROM em estudo e as pontuações de mudança nos instrumentos de comparação; (2) hipóteses sobre as diferenças esperadas nas pontuações de mudança entre diferentes subgrupos; ou (3) hipóteses sobre a magnitude da mudança nas pontuações que podemos esperar após receber um tratamento com eficácia conhecida no construto de interesse [115].

Responsividade: Métodos

Resp1 – Hipóteses

Formule hipóteses e forneça justificativas para cada hipóteses.

Explicação: Da mesma forma que a validade de construto (veja o item ConV1), recomendamos que você forneça todas as hipóteses, incluindo a direção e a magnitude esperadas das correlações entre as pontuações de alteração da PROM e as pontuações de alteração no(s) instrumento(s) de comparação, ou a direção e a magnitude esperadas das diferenças nas pontuações de alteração da PROM entre os grupos. Além disso, recomendamos que você explique os fundamentos para a escolha das hipóteses (veja o item ConV1). Hipóteses sobre tamanhos de efeito/*Effect Size* (ES), média de resposta padronizada/ *Standardized Response Mean* (SRM) ou área sob a curva ROC/*area under the* ROC *curve* também podem ser usadas, mas somente se for fornecida uma hipótese explícita e uma justificativa para a magnitude esperada do ES, SRM ou área sob a curva ROC/*area under the* ROC *curve* após uma intervenção com eficácia conhecida [109]. Sem especificar a direção e a magnitude da pontuação da mudança, é impossível decidir posteriormente se os resultados são consistentes com a hipótese [109].

Elementos essenciais

• Hipóteses, incluindo a direção e a magnitude esperadas das correlações entre as pontuações de mudança da PROM e as pontuações de mudança nos instrumentos de comparação (se aplicável).

• Hipóteses incluindo a direção e a magnitude esperadas das diferenças nas pontuações de alteração da PROM entre os grupos (se aplicável).

• Hipóteses, incluindo ES, SRM ou área sob a curva ROC/*area under the* ROC *curve*.

• Justificativas para as hipóteses.

Exemplo 1

"For both the Pediatric Quality of Life Inventory (PedsQL) and the Children's Dermatology Life Quality Index (CDLQI), we formulated 7 hypotheses (Table 1). These hypotheses were based on previous studies assessing responsiveness and methodology guidelines by COSMIN (COnsensus-based Standards for the Selection of Health Measurement INstruments). [ref]

Table 1. Hypotheses for testing responsiveness of the PedsQL and CDLQI total scores. If ≥75% of these hypotheses were confirmed, it was considered responsive to change

PedsQL<br>1. High positive correlation (>0.5) between PedsQL total change score and the GRC scale<br>2. High positive correlation (>0.5) between PedsQL total change score and CDLQI total change score<br>3. Moderate positive correlation (0.3-0.5) between PedsQL total change score and the CDLQI leisure subscale change score<br>4. Low positive (<0.3) or negative correlation between PedsQL total change score and the CDLQI treatment subscale change score<br>5. Patients indicating improvement on the GRC scale should have a positive mean change score<br>6. Patients indicating worsening on the GRC scale should have a negative mean change score<br>7. The mean change score of patients indicating improvement should be higher than the mean change score of unchanged patients, which in turn should be higher than the mean change score of worsened patients<br>CDLQI<br>1. High positive correlation (>0.5) between CDLQI total change score and the GRC scale<br>2. High positive correlation (>0.5) between CDLQI total change score and PedsQL total change score<br>3. Moderate positive correlation (0.3-0.5) between CDLQI total change score and the PedsQL physical subscale change score<br>4. Low positive (<0.3) or negative correlation between CDLQI total change score and the PedsQL school subscale change score<br>5. Patients indicating improvement on the GRC scale should have a positive mean change score<br>6. Patients indicating worsening on the GRC scale should have a negative mean change score<br>7. The mean change score of patients indicating improvement should be higher than the mean change score of unchanged patients, which in turn should be higher than the mean change score of worsened patients

Correlation strength was interpreted as high (≥0.5), moderate (0.3-0.5), and low (<0.3), based on previous studies and guidelines for assessing responsiveness. [ref]
Hypotheses 5, 6, and 7 concerned the mean change scores of improved, unchanged,

and worsened patients according to the global rating of change (GRC) scale. (...) Patients were categorized as improved or unchanged according to their GRC scale response. With an AUC [*area under the* ROC *curve*] of ≥0.7, an instrument can be considered responsive. [ref]" [116]

Tradução: "Para o *Pediatric Quality of Life Inventory* (PedsQL) e o *Children's Dermatology Life Quality Index* (CDLQI), formulamos sete hipóteses (Tabela 1). Essas hipóteses foram baseadas em estudos anteriores que avaliaram a responsividade e as diretrizes metodológicas do COSMIN (*COnsensus-based Standards for the Selection of Health Measurement INstruments*). [ref]

Tabela 1. Hipóteses para testar a responsividade das pontuações totais do PedsQL e do CDLQI. Se ≥75% dessas hipóteses fossem confirmadas, a escala era considerada responsiva à mudança

PedsQL<br>1. Alta correlação positiva (>0,5) entre a pontuação total de mudança do PedsQL e a escala GRC<br>2. Alta correlação positiva (>0,5) entre a pontuação total de mudança do PedsQL e a pontuação total de mudança do CDLQI<br>3. Correlação positiva moderada (0,3-0,5) entre a pontuação de mudança total do PedsQL e a pontuação de mudança da subescala de lazer do CDLQI<br>4. Correlação positiva baixa (<0,3) ou negativa entre a pontuação de mudança total do PedsQL e a pontuação de mudança da subescala de tratamento do CDLQI<br>5. Os pacientes que indicarem melhora na escala GRC devem ter uma pontuação de mudança média positiva<br>6. Os pacientes que indicarem piora na escala GRC devem ter uma pontuação média de mudança negativa<br>7. A pontuação média de mudança dos pacientes que indicam melhora deve ser maior do que a pontuação média de mudança dos pacientes inalterados, que, por sua vez, deve ser maior do que a pontuação média de mudança dos pacientes com piora<br><br>CDLQI<br>1. Alta correlação positiva (>0,5) entre a pontuação total de mudança do CDLQI e a escala GRC<br>2. Alta correlação positiva (>0,5) entre a pontuação total de mudança do CDLQI e a pontuação total de mudança do PedsQL<br>3. Correlação positiva moderada (0,3-0,5) entre a pontuação total de mudança do CDLQI e a pontuação de mudança da subescala física do PedsQL<br>4. Correlação positiva baixa (<0,3) ou negativa entre a pontuação de mudança total do CDLQI e a pontuação de mudança da subescala escolar do PedsQL<br>5. Os pacientes que indicarem melhora na escala GRC devem ter uma pontuação de mudança média positiva<br>6. Os pacientes que indicarem piora na escala GRC devem ter uma pontuação de alteração média negativa<br>7. A pontuação média de mudança dos pacientes que indicam melhora deve ser maior do que a pontuação média de mudança dos pacientes inalterados, que, por sua vez, deve ser maior do que a pontuação média de mudança dos pacientes com piora

A força da correlação foi interpretada como alta (≥0,5), moderada (0,3-0,5) e baixa (<0,3), com base em estudos e diretrizes anteriores para avaliar a responsividade [ref]. As hipóteses 5, 6 e 7 referiam-se às pontuações médias de mudança de pacientes melhorados, inalterados e piorados de acordo com a escala de *Global Rating of Change* (GRC). (...) Os pacientes foram classificados como melhorados ou inalterados de acordo com a resposta da escala GRC. Com uma AUC [área sob a curva ROC/*area under the* ROC *curve*] de ≥0,7, um instrumento pode ser considerado responsivo. [ref]"

Resp2 - Intervenção/Exposição

Forneça a intervenção dada ou a exposição no período intermediário (ou informe que nenhuma intervenção foi fornecida).

Explicação: Todas as intervenções ou exposições que os pacientes receberam no período intermediário devem ser descritas. Isso permite que os leitores de seu artigo identifiquem se as alterações no construto ocorreram devido a essa intervenção ou exposição específica. Para descrever intervenções (por exemplo, cirurgia, medicamentos, tratamento fisioterapêutico, terapia psicológica etc.) ou exposições (por exemplo, poluição ambiental, eventos traumáticos, curso natural da doença etc.), recomendamos que você inclua todas as informações essenciais em termos de equipamentos, suprimentos, medicamentos ou software; membros da equipe (por exemplo, cirurgião, enfermeiro, fisioterapeuta), incluindo suas funções; abordagem da intervenção, incluindo número de sessões, progressão da intervenção, instruções para o paciente realizar a intervenção, etc. [117, 118]. Se você usou dados secundários de outro estudo, pode fornecer citações do protocolo de intervenção ou do estudo primário para obter mais detalhes sobre a intervenção ou exposição. Se nenhuma intervenção ou exposição tiver sido fornecida, recomendamos que você informe isso para que os leitores possam identificar que ocorreram mudanças no construto apesar de nenhuma intervenção ou exposição.

Elementos essenciais

- Intervenção realizada ou exposição no período intermediário (se houver ou não).
- Descrições da intervenção ou exposição.

Exemplo 1

"Participants completed all study assessments both before heart transplant (i.e., "baseline," including any time after the patient was listed for transplant) and after transplant (i.e., "follow-up," 8-12 weeks after surgery, deemed by cardiologist coinvestigators as the minimum time after transplant at which a clinically significant improvement in functioning is typically observed)." [119]

Tradução: "Os participantes concluíram todas as avaliações do estudo antes do transplante cardíaco (ou seja, "linha de base", incluindo qualquer momento após o paciente ter sido listado para o transplante) e após o transplante (ou seja, "acompanhamento", 8 a 12 semanas após a cirurgia, considerado pelos co-investigadores cardiologistas como o tempo mínimo após o transplante no qual uma melhora clinicamente significativa na funcionalidade é normalmente observada)."

Resp3 - Análises estatísticas

Especifique todos os métodos estatísticos usados para testar as hipóteses.

Explicação: A estatística de responsividade depende da unidade de medida, de acordo com sua hipótese. Para pontuações contínuas, as correlações ou área sob a curva ROC/*area under the* ROC *curve* são preferíveis para avaliar a responsividade. Recomendamos que você informe as correlações esperadas com direção (positiva ou negativa) e magnitude (absoluta ou relativa) [109], ignore os valores de p das correlações, e informe a magnitude esperada da área sob a curva ROC/*area under the* ROC *curve* (veja o item ConV3). Se forem usados tamanhos de efeito ou médias de resposta padronizadas, suas magnitudes esperadas devem ser relatadas. Para pontuações dicotômicas, a sensibilidade e a especificidade devem ser usadas e suas magnitudes esperadas devem ser especificadas (veja o item CriV2). A especificação de todos os métodos estatísticos para testar cada hipótese permite que os leitores interpretem os resultados e reproduzam suas análises.

Elementos essenciais

• Métodos estatísticos usados para testar se os resultados estão de acordo com a hipótese.
• Para pontuações contínuas, correlações com direção e magnitude.
• Para pontuações contínuas, magnitude esperada da área sob a curva ROC/*area under the* ROC *curve*.
• Para pontuações dicotômicas, sensibilidade e especificidade esperadas.

Exemplo 1

"Responsiveness was, according to the COSMIN guidelines [ref], assessed by 2 methods: (1) the criterion approach by assessing the area (AUC) under the Receiver Operating Curve (ROC) and (2) the construct approach by hypotheses testing. To assess the criterion approach, the population was dichotomised into an 'improved' group and an 'unchanged' group (...) on the global rating of change scale (GROC) (...). The AUC was calculated as the ability of the Quick Disabilities of the Arm, Shoulder and Hand questionnaire (QuickDASH) and Patient-Specific Functional Scale (PSFS) to discriminate between patients classified as 'improved' and 'unchanged'. An AUC of at least 0.70 was regarded as acceptable responsiveness [ref]. To assess the construct approach, 9 a-priori hypotheses were formulated and tested for both the QuickDASH and PSFS. These hypotheses were (...) described in Table 1. The data were assumed to be normally distributed if there was no or minimal difference between the mean and median value, confirmed by histograms, Q plot and the Shapiro-Wilk test. Pearson correlation

coefficient was used if the data were normally distributed, otherwise, a Spearman's rank correlation coefficient was used. A correlation of less than 0.30 indicates a weak correlation, at least 0.30 and less than 0.60 indicates moderate correlation, and a correlation at least 0.60 indicates good correlation [ref]. The standardised response mean (SRM) was calculated by dividing the mean change score by the standard deviation (SD) of the change. The effect size (ES) was calculated by dividing the mean change score by the SD of the baseline scores [ref]. An instrument was considered having acceptable responsiveness, based on the construct approach, if meeting at least 75% of the hypotheses according to the COSMIN guidelines [ref]." [120]

Tradução: "A responsividade foi avaliada por dois métodos, de acordo com as diretrizes COSMIN [ref]: (1) a abordagem de critério avaliando a [área sob a curva ROC/*area under the* ROC *curve* (AUC)] e (2) a abordagem de construto por meio de testes de hipóteses. Para avaliar a abordagem de critério, a população foi dicotomizada em um grupo "melhorado" e um grupo "inalterado" (...) na *global rating of change scale* (GROC) (...). A AUC foi calculada como a capacidade do questionário *Quick Disabilities of the Arm, Shoulder and Hand* (QuickDASH) e da *Patient-Specific Functional Scale* (PSFS) de discriminar entre os pacientes classificados como "melhorados" e "inalterados". Uma AUC de pelo menos 0,70 foi considerada como responsividade aceitável [ref]. Para avaliar a abordagem de construto, 9 hipóteses a priori foram formuladas e testadas para o QuickDASH e o PSFS. Essas hipóteses foram (...) descritas na Tabela 1. Os dados foram considerados normalmente distribuídos se não houvesse diferença ou houvesse diferença mínima entre a média e a mediana, confirmada por histogramas, gráfico Q e teste de Shapiro-Wilk. O coeficiente de correlação de Pearson foi utilizado se os dados apresentassem distribuição normal; caso contrário, utilizou-se o coeficiente de correlação por postos de Spearman. Uma correlação inferior a 0,30 indica uma correlação fraca, pelo menos 0,30 e menos de 0,60 indica uma correlação moderada e uma correlação de pelo menos 0,60 indica uma boa correlação [ref]. A média de resposta padronizada/*Standardized Response Mean* (SRM) foi calculada dividindo-se a pontuação média de mudança pelo desvio padrão (DP) da mudança. O tamanho do efeito/*Effect Size* (ES) foi calculado dividindo-se a pontuação média da mudança pelo DP das pontuações da linha de base [ref]. Um instrumento foi considerado com responsividade aceitável, com base na abordagem de construto, se atendesse a pelo menos 75% das hipóteses de acordo com as diretrizes do COSMIN [ref]."

Responsividade: Resultados

Resp4 - Análises estatísticas

Forneça resultados para as estatísticas calculadas e especifique, para a abordagem de construto, se cada resultado está de acordo com sua hipótese.

Explicação: Da mesma forma que no ConV3, recomendamos que você relate seus resultados em relação às hipóteses correspondentes usadas para avaliar a responsividade. Recomendamos que você informe cada resultado na forma de um valor numérico para a direção e a magnitude de uma correlação entre as pontuações de alterações; ou a diferença média das pontuações de alterações entre os grupos (veja o item ConV2); ou o valor da área sob a curva ROC/*area under the* ROC *curve* e seu intervalo de confiança (IC), bem como a sensibilidade (taxa de verdadeiro positivo) e a especificidade (taxa de verdadeiro negativo) em diferentes pontos de corte para a abordagem de validade de critério. Você também pode incluir o gráfico da área sob a curva ROC/*area under the* ROC *curve* para ilustrar a compensação entre sensibilidade e especificidade em diferentes pontos de corte (veja o item CriV3).

Elementos essenciais

- Resultados para estatísticas calculadas sobre análises de responsividade.
- Resultados que estão (ou não) de acordo com sua hipótese (para a abordagem de construto).

Exemplo 1

"Criterion approach of responsiveness. Dichotomisation of the GROC [global rating of change scale] showed that 50 patients (47.6%) improved and 55 patients (52.4%) were stable; 1 patient (0.9%) were excluded in the ROC curves analysis, since he or she had worsened clinical condition. The ROC curves (Fig. 2) were similar for both questionnaires, with an AUC for the Quick Disabilities of the Arm, Shoulder and Hand questionnaire (QuickDASH) of 0.75 (95% CI: 0.66, 0.84) and an AUC for the Patient-Specific Functional Scale (PSFS) of 0.75 (95% CI: 0.65, 0.85). The responsiveness for both questionnaires was therefore considered satisfactory based on the criterion approach.

Construct approach of responsiveness. Responsiveness according to testing 9 a-priori hypotheses (Table 1) were met by both instruments; the QuickDASH met 7 hypotheses (77.8%) and the PSFS met 8 hypotheses (88.9%). The correlations between the QuickDASH/PSFS and comparator instruments are presented in Table 4.

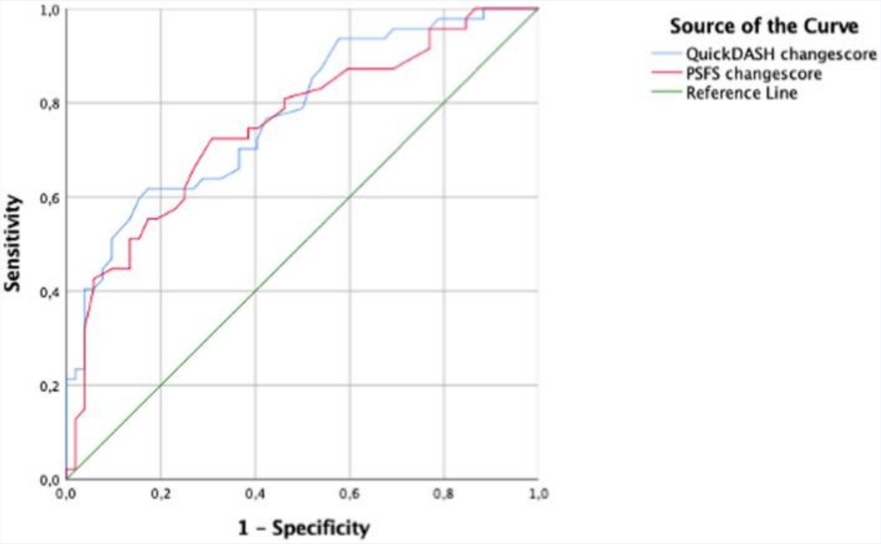

Figure 2. Receiver operating characteristic curves for the QuickDASH and PSFS for 'improved' and 'unchanged' on the GROC. QuickDASH at 3-month follow-up (area under curve = 0.75). PSFS at 3-month follow-up (area under curve = 0.75). Abbreviations: GROC, global rating of change; PSFS, Patient-Specific Functional Scale; QuickDASH, shortened version of the Disabilities of the Arm, Shoulder and Hand questionnaire

Table 4. Correlations among the PROMs' change scores (n = 117)

	QuickDASH[a]	PSFS[a]
QuickDASH (0-100)	-	0.45 (0.28, 0.59)
PSFS (0-10)	0.45 (0.28, 0.59)	-
NRS (0-10)	0.62 (0.49, 0.72)	0.32 (0.14, 0.49)
SQK (0-10)	0.37 (0.20, 0.52)	0.38 (0.20, 0.53)
WA (0-10)	0.44 (0.27, 0.58)	0.46 (0.29, 0.60)
HSCL-25 (0-10)	0.37 (0.20, 0.52)	0.25 (0.06, 0.42)
GROC (1-7)	−0.47 (− 0.61, − 0.31)	−0.50 (− 0.64, − 0.34)

Abbreviations: GROC = global rating of change scale; HSCL-25 = Hopkins Symptom Checklist total score; NRS = numeric pain rating scale; PSFS = Patient-Specific Functional scale; SQK = Single substitute question for kinesiophobia; QuickDASH = Quick Disabilities of the Arm, Hand and Shoulder questionnaire; WA = Workability
n refers to the total sample sizes and may deviate in some of the correlation analysis due to missing data
All correlations were significant at P < 0.01
[a]Values in parentheses are 95% confidence interval
[120]

Tradução: "Abordagem de critério da responsividade. A dicotomização da GROC [*global rating of change scale*] mostrou que 50 pacientes (47,6%) melhoraram e 55 pacientes (52,4%) ficaram estáveis; 1 paciente (0,9%) foi excluído da análise das curvas ROC, pois apresentou piora do quadro clínico. As curvas ROC (Fig. 2) foram semelhantes para ambos os questionários, com uma AUC para o questionário *Quick Disabilities of the Arm, Shoulder and Hand* (QuickDASH) de 0,75 (IC 95%: 0,66, 0,84) e uma AUC para a *Patient-Specific Functional Scale* (PSFS) de 0,75 (IC 95%: 0,65, 0,85). A responsividade

de ambos os questionários foi, portanto, considerada satisfatória com base na abordagem de critério.

Abordagem de construto da responsividade. A responsividade de acordo com o teste de 9 hipóteses a priori (Tabela 1) foi atendida por ambos os instrumentos; o QuickDASH atendeu a 7 hipóteses (77,8%) e o PSFS atendeu a 8 hipóteses (88,9%). As correlações entre o QuickDASH/PSFS e os instrumentos de comparação são apresentadas na Tabela 4.

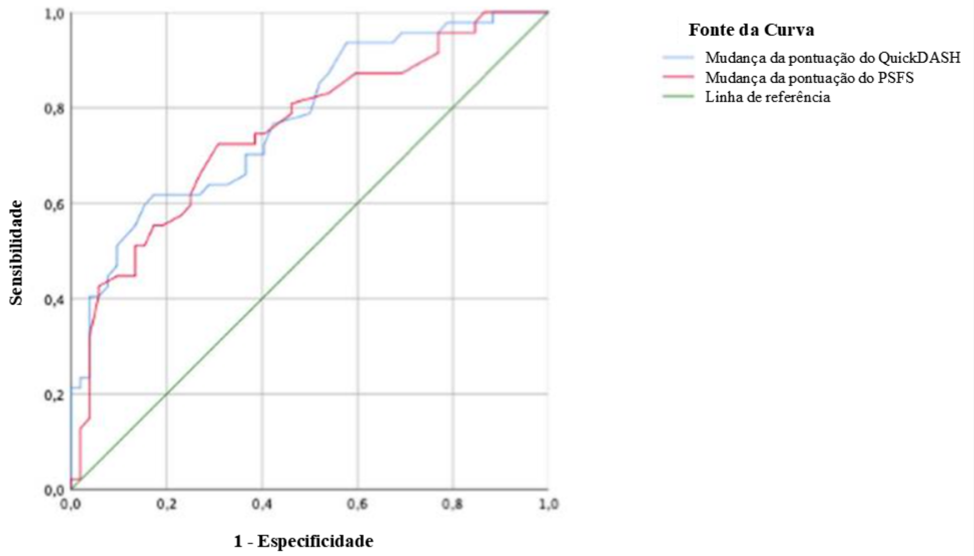

Figura 2. Características da curva ROC para o QuickDASH e PSFS para "melhorado" e "inalterado" no GROC. QuickDASH no acompanhamento de 3 meses (área sob a curva = 0,75). PSFS no acompanhamento de 3 meses (área sob a curva = 0,75). Abreviações: GROC, *global rating of change*; PSFS, *Patient-Specific Functional Scale*; QuickDASH, versão abreviada do questionário *Disabilities of the Arm, Shoulder and Hand*

Tabela 4. Correlações entre as pontuações de alteração dos PROMs (n = 117)

	QuickDASH[a]	PSFS[a]
QuickDASH (0-100)	-	0.45 (0.28, 0.59)
PSFS (0-10)	0,45 (0,28, 0,59)	-
NRS (0-10)	0,62 (0,49, 0,72)	0,32 (0,14, 0,49)
SQK (0-10)	0,37 (0,20, 0,52)	0,38 (0,20, 0,53)
WA (0-10)	0,44 (0,27, 0,58)	0,46 (0,29, 0,60)
HSCL-25 (0-10)	0,37 (0,20, 0,52)	0,25 (0,06, 0,42)
GROC (1-7)	−0,47 (− 0,61, − 0,31)	−0,50 (− 0,64, − 0,34)

Abreviações: GROC = *global rating of change scale*; HSCL-25 = *Hopkins Symptom Checklist total score*; NRS = *numeric pain rating scale*; PSFS = *Patient-Specific Functional scale*; SQK = *Single substitute question for kinesiophobia*; QuickDASH = *Quick Disabilities of the Arm, Hand and Shoulder questionnaire*;
WA = *Workability*
n refere-se ao tamanho total das amostras e pode se desviar em algumas análises de correlação devido à falta de dados
Todas as correlações foram significativas com P < 0,01
[a]Os valores entre parênteses são o intervalo de confiança de 95%

Referências

1. Mokkink LB, Terwee CB, Patrick DL, Alonso J, Stratford PW, Knol DL, et al. The COSMIN study reached international consensus on taxonomy, terminology, and definitions of measurement properties for health-related patient-reported outcomes. J Clin Epidemiol. 2010;63:737–45.

2. Mokkink LB, Prinsen CAC, Bouter LM, Vet HCW de, Terwee CB. The COnsensus-based Standards for the selection of health Measurement INstruments (COSMIN) and how to select an outcome measurement instrument. Brazilian J Phys Ther. 2016;20:105–13.

3. Mokkink LB, Terwee CB, Patrick DL, Alonso J, Stratford PW, Knol DL, et al. The COSMIN checklist for assessing the methodological quality of studies on measurement properties of health status measurement instruments : an international Delphi study. Qual Life Res. 2010;19:539–49.

4. Boers M, Idzerda L, Kirwan JR, Beaton D, Escorpizo R, Boonen A, et al. Toward a Generalized Framework of Core Measurement Areas in Clinical Trials: A Position Paper for OMERACT 11. J Rheumatol. 2014;41:978–85.

5. Streiner DL, Norman GR, Cairney J. Health Measurement Scales. Oxford University Press; 2015.

6. Mokkink LB, Elsman EBM, Terwee CB. COSMIN guideline for systematic reviews of patient-reported outcome measures version 2.0. Qual Life Res. 2024. https://doi.org/10.1007/s11136-024-03761-6.

7. Gagnier JJ, Lai J, Mokkink LB, Terwee CB. COSMIN reporting guideline for studies on measurement properties of patient-reported outcome measures. Qual Life Res. 2021;30:2197–218.

8. Gagnier JJ, de Arruda GT, Terwee CB, Mokkink LB, Elsman EBM, Firth AD, et al. COSMIN reporting guideline for studies on measurement properties of patient-reported outcome measures: version 2.0. Qual Life Res. 2025. https://doi.org/10.1007/s11136-025-03950-x.

9. FDA. Guidance for industry: patient-reported outcome measures: use in medical product development to support labeling claims: draft guidance. Health Qual Life Outcomes. 2006;4:79.

10. Shadzi MR, Rahmanian M, Heydari A, Salehi A. Structural validity of the Pittsburgh Sleep Quality Index among medical students in Iran. Sci Rep. 2024;14:1538.

11. Itakussu EY, Morita AA, Kakitsuka EE, Kuwahara RM, Anami EHT, Pitta F, et al. The Brazilian-Portuguese version of the Upper Extremity Functional Index (UEFI): Translation, cross-cultural adaptation and measurement properties for Brazilian adults after a burn injury. Burns. 2024;50:219–25.

12. De Vet HCW, Terwee CB, Mokkink LB, Knol DL. Measurement in Medicine - A practical guide. 1st edition. New York: Cambridge University Press; 2011.

13. Rathnayake N, Karunadasa R, Abeygunasekara T, De Zoysa W, Palangasinghe D, Lekamwasam S. Katz index of activities of daily living in assessing functional status of older people: Reliability and validity of Sinhala version. Dialogues Heal.

2023;2:100134.

14. Ivarsson M, Danielsson H, Andersson AK, Gothilander J, Granlund M. Structural validity and internal consistency of the Strengths and Stressors in Parenting (<scp>SSF</scp>) Questionnaire in parents of children with developmental disabilities. Scand J Psychol. 2023;64:486–94.

15. Frota NT, Fidelis-de-Paula-Gomes CA, Pontes-Silva A, Pinheiro JS, de Jesus SFC, Apahaza GHS, et al. 15-item Roland-Morris Disability Questionnaire (RMDQ-15): structural and criterion validity on patients with chronic low back pain. BMC Musculoskelet Disord. 2022;23:1–8.

16. Terwee CB, van der Willik EM, van Breda F, van Jaarsveld BC, van de Putte M, Jetten IW, et al. Responsiveness and minimal important change of seven PROMIS computerized adaptive tests (CAT) in patients with advanced chronic kidney disease. J Patient-Reported Outcomes. 2023;7:35.

17. Durgam N, Dashputre AA, Moshkovich O, Rezaie A, Martinez N, Enayati P, et al. Content validation of a daily patient-reported outcome measure for assessing symptoms in patients with Small Intestinal Bacterial Overgrowth. Qual Life Res. 2023;32:2573–85.

18. de Arruda GT, de Melo Mantovan SG, Da Roza T, Silva BI da, Tonon da Luz SC, Avila MA. WHODAS measurement properties for women with dysmenorrhea. Health Qual Life Outcomes. 2023;21:55.

19. Lee MK, Basch E, Mitchell SA, Minasian LM, Langlais BT, Thanarajasingam G, et al. Reliability and validity of PRO-CTCAE® daily reporting with a 24-hour recall period. Qual Life Res. 2023;32:2047–58.

20. Feagan BG, Sandborn WJ, Sands BE, Liu Y, Vetter M, Mathias SD, et al. Qualitative and psychometric evaluation of the PROMIS®-Fatigue SF-7a scale to assess fatigue in patients with moderately to severely active inflammatory bowel disease. J Patient-Reported Outcomes. 2023;7:115.

21. Dubinsky MC, Delbecque L, Hunter T, Harding G, Stassek L, Moses RE, et al. Validation of the bowel urgency numeric rating scale in patients with Crohn’s disease: results from a mixed methods study. Qual Life Res. 2023;32:3403–15.

22. Oladapo A, Ito D, Rodriguez AM, Philpott S, Krupnick R, Allen V, et al. Psychometric evaluation of a patient-reported outcomes instrument for congenital thrombotic thrombocytopenic purpura. J Patient-Reported Outcomes. 2023;7:68.

23. Elsman EBM, Roorda LD, Smidt N, de Vet HCW, Terwee CB. Measurement properties of the Dutch PROMIS-29 v2.1 profile in people with and without chronic conditions. Qual Life Res. 2022;31:3447–58.

24. Pearson NA, Tutton E, Martindale J, Strickland G, Thompson J, Packham JC, et al. Development of the Warwick Axial Spondyloarthritis faTigue and Energy questionnaire (WASTEd)—a new patient-reported outcome measure. Rheumatol Adv Pract. 2022;6.

25. Brod M, Waldman LT, Shu AD, Smith A. Content validation of the SF-36v2® Health Survey Acute for use in hypoparathyroidism. Qual Life Res. 2023;32:1795–806.

26. Pietrabissa G, Castelnuovo G, Semonella M, Mannarini S, Rossi AA. Measuring Motivations to Eat Palatable Foods: Adaptation and Psychometric Properties of the Italian Version of the Palatable Eating Motives Scale (PEMS-IT). Healthcare.

2024;12:574.

27. Wilson IB, Cleary PD. Linking clinical variables with health-related quality of life. A conceptual model of patient outcomes. JAMA. 1995;273:59–65.

28. World Health Organization (WHO). International Classification of functioning, disability and health: ICF. World Health Organization. 2001.

29. Boers M, Beaton DE, Shea BJ, Maxwell LJ, Bartlett SJ, Bingham CO, et al. OMERACT Filter 2.1: Elaboration of the Conceptual Framework for Outcome Measurement in Health Intervention Studies. J Rheumatol. 2019;46:1021–7.

30. de Arruda GT, de Andrade DF, Virtuoso JF. Internal structure and classification of pelvic floor dysfunction distress by PFDI-20 total score. J Patient-Reported Outcomes. 2022;6:51.

31. Mantovan SG de M, de Arruda GT, Da Roza T, da Silva BI, Avila MA, da Luz SCT. Translation, cross-cultural adaptation, and measurement properties of the dysmenorrhea symptom interference (DSI) scale–Brazilian version. Brazilian J Phys Ther. 2024;28:101065.

32. Harrison C, Apon I, Ardouin K, Sidey-Gibbons C, Klassen A, Cano S, et al. The Development, Deployment, and Evaluation of the CLEFT-Q Computerized Adaptive Test: A Multimethods Approach Contributing to Personalized, Person-Centered Health Assessments in Plastic Surgery. J Med Internet Res. 2023;25:e41870.

33. Sivertsen HE, Helvik A-S, Gjøra L, Haugan G. Psychometric validation of the Hospital Anxiety and Depression Scale (HADS) in community-dwelling older adults. BMC Psychiatry. 2023;23:903.

34. Galenkamp H, Stronks K, Mokkink LB, Derks EM. Measurement invariance of the SF-12 among different demographic groups: The HELIUS study. PLoS One. 2018;13:e0203483.

35. van Kooten JAMC, Terwee CB, Luijten MAJ, Steur LMH, Pillen S, Wolters NGJ, et al. Psychometric properties of the Patient-Reported Outcomes Measurement Information System (PROMIS) Sleep Disturbance and Sleep-Related Impairment item banks in adolescents. J Sleep Res. 2021;30.

36. Schougaard LMV, Laurberg T, Lomborg K, Hansen TK, Hjollund NH, Jensen AL. Test–retest reliability and measurement error of the WHO-5 Well-being Index and the Problem Areas in Diabetes questionnaire (PAID) used in telehealth among patients with type 1 diabetes. J Patient-Reported Outcomes. 2022;6:99.

37. Karpecki PM, Findley A, Sloesen BJ, Hodson N, Bentley S, Arbuckle R, et al. Qualitative Research to Understand the Patient Experience and Evaluate Content Validity of the Chronic Ocular Pain Questionnaire (COP-Q). Ophthalmol Ther. 2024;13:615–33.

38. Quintana DT, Casanova MP, Cady AC, Baker RT. Assessing the Structural Validity of the Knee Injury and Osteoarthritis Outcome Score Scale. Healthcare. 2024;12:414.

39. Stathis P, Papadopoulos G. Evaluation and validation of a patient-reported quality-of-life questionnaire for Parkinson’s disease. J Patient-Reported Outcomes. 2022;6:17.

40. Fayers PM, Hand DJ. Factor analysis, causal indicators and quality of life. Qual Life Res. 1997;6:139–50.

41. Jarvis CB, MacKenzie SB, Podsakoff PM. A Critical Review of Construct Indicators and Measurement Model Misspecification in Marketing and Consumer Research. J Consum Res. 2003;30:199–218.

42. Arruda GT de, Silva EV da, Somavilla P, Oliveira MCR de, Braz MM. Female Genital Self-image Scale (FGSIS): cut-off point, reliability, and validation of measurement properties in Brazilian women. Fisioter e Pesqui. 2023;30.

43. Becker B, Bracher M, Chauhan D, Rendas-Baum R, Lin X, Raymond K, et al. Development, psychometric evaluation and cognitive debriefing of the rheumatoid arthritis symptom and impact questionnaire (RASIQ). J Patient-Reported Outcomes. 2021;5:129.

44. Schnettler B, Miranda-Zapata E, Lobos G, Lapo M, Grunert KG, Adasme-Berríos C, et al. Cross-cultural measurement invariance in the satisfaction with food-related life scale in older adults from two developing countries. Health Qual Life Outcomes. 2017;15:113.

45. de Sousa AP, de Arruda GT, Pontes-Silva A, de Souza MC, Driusso P, Avila MA. Measurement properties of the Brazilian online version of the Fibromyalgia Rapid Screening Tool (FiRST). Adv Rheumatol. 2022;62:39.

46. Reeve BB, Edwards LJ, Jaeger BC, Hinds PS, Dampier C, Gipson DS, et al. Assessing responsiveness over time of the PROMIS® pediatric symptom and function measures in cancer, nephrotic syndrome, and sickle cell disease. Qual Life Res. 2018;27:249–57.

47. Guest G, Bunce A, Johnson L. How Many Interviews Are Enough? Field methods. 2006;18:59–82.

48. Francis JJ, Johnston M, Robertson C, Glidewell L, Entwistle V, Eccles MP, et al. What is an adequate sample size? Operationalising data saturation for theory-based interview studies. Psychol Health. 2010;25:1229–45.

49. Malterud K, Siersma VD, Guassora AD. Sample Size in Qualitative Interview Studies. Qual Health Res. 2016;26:1753–60.

50. Browne RH. On the use of a pilot sample for sample size determination. Stat Med. 1995;14:1933–40.

51. Johanson GA, Brooks GP. Initial Scale Development: Sample Size for Pilot Studies. Educ Psychol Meas. 2010;70:394–400.

52. Comrey AL, Lee HB. A First Course in Factor Analysis. Psychology Press; 2013.

53. Edelen MO, Reeve BB. Applying item response theory (IRT) modeling to questionnaire development, evaluation, and refinement. Qual Life Res. 2007;16:5–18.

54. Brown T. Confirmatory Factor Analysis for Applied Research. 2nd editio. The Guilford Press; 2015.

55. Mokkink LB, de Vet H, Diemeer S, Eekhout I. Sample size recommendations for studies on reliability and measurement error: an online application based on simulation studies. Heal Serv Outcomes Res Methodol. 2023;23:241–65.

56. Bujang MA, Baharum N. Guidelines of the minimum sample size requirements for Kappa agreement test. Epidemiol Biostat Public Heal. 2022;14.

57. Fleiss JL, Levin B, Paik MC. Statistical Methods for Rates and Proportions. Wiley;

2003.

58. Landis JR, Koch GG. The measurement of observer agreement for categorical data. Biometrics. 1977;33:159–74.

59. Ikeuchi K, Nishida S, Karikawa M, Sakamoto C, Mori F, Tanaka M. Development of an Assessment Tool to Measure the Quality of Life Goal Setting for Cancer Survivors: A Content Validity Study. Cureus. 2024. https://doi.org/10.7759/cureus.71272.

60. Clément C, Lvovschi V-E, Verot E, du Sartz de Vigneulles B, Darlington-Bernard A, Bourgeois D, et al. Supporting health education policies: translation, cross-cultural adaptation and validation of a health literacy instrument, in French. Front Public Heal. 2023;11.

61. Li Y, Lyu L, Fan X, Xu L, Li Y, Song R. Reliability, validity and minimal detectable change of the Chinese Version of the Assessment of Physical Activity in Frail Older People (APAFOP-C). BMC Geriatr. 2024;24:582.

62. Bazarganipour F, Taghavi SA, Montazeri A, Ahmadi F, Derakhshideh Z, Asadikalameh Z. Psychometric properties of the Iranian version of the polycystic ovary syndrome quality of life questionnaire for married and unmarried women with PCOS: PCOSQoL-47 and PCOSQoL-42. BMC Psychol. 2024;12:722.

63. Vandenbroucke JP, von Elm E, Altman DG, Gøtzsche PC, Mulrow CD, Pocock SJ, et al. Strengthening the Reporting of Observational Studies in Epidemiology (STROBE): Explanation and elaboration. Int J Surg. 2014;12:1500–24.

64. van der Baan FH, Koldenhof JJ, de Nijs EJ, Echteld MA, Zweers D, Hesselmann GM, et al. Validation of the Dutch version of the Edmonton Symptom Assessment System. Cancer Med. 2020;9:6111–21.

65. van Kooten JAMC, van Litsenburg RRL, Yoder WR, Kaspers GJL, Terwee CB. Validation of the PROMIS Sleep Disturbance and Sleep-Related Impairment item banks in Dutch adolescents. Qual Life Res. 2018;27:1911–20.

66. Dornonville de la Cour FL, Schow T, Andersen TE, Petersen AH, Zornhagen G, Visser-Keizer AC, et al. Measurement Properties of the Dutch Multifactor Fatigue Scale in Early and Late Rehabilitation of Acquired Brain Injury in Denmark. J Clin Med. 2023;12:2587.

67. Zulmiyusrini P, Yamin M, Muhadi M, Kurniawan J, Salim S. The validity and reliability of Indonesian version of atrial fibrillation effect on quality of life (AFEQT) questionnaire for atrial fibrillation patients. J Patient-Reported Outcomes. 2023;7:133.

68. Martínez-Fernández MV, Sarabia-Cobo CM, Sánchez-Labraca N. Cross-cultural adaptation, reliability, validity and responsiveness of the Michigan Hand Outcomes Questionnaire (MHQ-Sp) in Spain. J Orthop Surg Res. 2024;19:256.

69. Mashayekh-Amiri S, Asghari Jafarabadi M, Rashidi F, Mirghafourvand M. Translation and measurement properties of the pelvic floor distress inventory-short form (PFDI-20) in Iranian reproductive age women. BMC Womens Health. 2023;23:333.

70. Stephan A, Stadelmann VA, Preiss S, Impellizzeri FM. Measurement properties of PROMIS short forms for pain and function in patients receiving knee arthroplasty. J Patient-Reported Outcomes. 2023;7:18.

71. Daescu A-MC, Dehelean L, Navolan D-B, Pop GN, Stoian DL. Psychometric

properties of the Romanian version of the female sexual function index (FSFI-RO). BMC Womens Health. 2023;23:528.

72. McHorney CA, Bensink ME, Burke LB, Belozeroff V, Gwaltney C. Development and psychometric validation of the Nausea/Vomiting Symptom Assessment patient-reported outcome (PRO) instrument for adults with secondary hyperparathyroidism. J Patient-Reported Outcomes. 2018;2:6.

73. Gharzai LA, Mierzwa ML, Peipert JD, Kirtane K, Casper K, Yadav P, et al. Monitoring Adverse Effects of Radiation Therapy in Patients With Head and Neck Cancer. JAMA Otolaryngol Neck Surg. 2023. https://doi.org/10.1001/jamaoto.2023.2177.

74. da Silva APAF, Bassi-Dibai D, Moreira BL, Gava AD, Takahasi HY, Salomão LGPB, et al. Cross-cultural adaptation, reliability and validation of the Gillette Functional Assessment Questionnaire (FAQ) into Brazilian Portuguese in patients with cerebral palsy. BMC Pediatr. 2023;23:165.

75. Oud T, Tuijtelaars J, Schenk J, Nollet F, Brehm M-A. Validity and reliability of the Dutch translation of the OPUS' client satisfaction with device module in chronic users of hand orthoses. Health Qual Life Outcomes. 2023;21:93.

76. Walton DM, MacDermid JC. A brief 5-item version of the Neck Disability Index shows good psychometric properties. Health Qual Life Outcomes. 2013;11:108.

77. Schuller W, Terwee CB, Klausch T, Roorda LD, Rohrich DC, Ostelo RW, et al. Psychometric properties of the Dutch-Flemish Patient-Reported Outcomes Measurement Information System Pain Behavior item bank in patients with musculoskeletal complaints. J Pain. 2019;20:1328–37.

78. Coppers A, Möller JC, Marks D. Psychometric properties of the short form of the Stroke Impact Scale in German-speaking stroke survivors. Health Qual Life Outcomes. 2021;19:190.

79. Rimehaug SA, Kaat AJ, Nordvik JE, Klokkerud M, Robinson HS. Psychometric properties of the PROMIS-57 questionnaire, Norwegian version. Qual Life Res. 2022;31:269–80.

80. Tacchino A, Ponzio M, Pedullà L, Podda J, Bragadin MM, Pedrazzoli E, et al. Italian validation of the Arm Function in Multiple Sclerosis Questionnaire (AMSQ). Neurol Sci. 2020;41:3273–81.

81. Tong A, Sainsbury P, Craig J. Consolidated criteria for reporting qualitative research (COREQ): a 32-item checklist for interviews and focus groups. Int J Qual Heal Care. 2007;19:349–57.

82. Mol T, Scholten E, van Bennekom C, Post M. Development of the self-regulation assessment and content validation using cognitive interviews in a multicultural post-rehabilitation population. Front Rehabil Sci. 2023;4.

83. Gries KS, Esser D, Wiklund I. Content Validity of CASA-Q Cough Domains and UCSD-SOBQ for Use in Patients with Idiopathic Pulmonary Fibrosis. Glob J Health Sci. 2013;5.

84. Kitchen H, Gandhi K, Carmichael C, Wyrwich KW, Lukic T, Al-Zubeidi T, et al. A Qualitative Study to Develop and Evaluate the Content Validity of the Vitiligo Patient Priority Outcome (ViPPO) Measures. Dermatol Ther (Heidelb). 2022;12:1907–24.

85. Kramer JM, Beasley JB, Caoili A, Kalb L, Urquilla MP, Klein AE, et al. Development and content validity of the Person Experiences Interview Survey (PEIS): a measure of the mental health services experiences of people with developmental disabilities. Front Psychiatry. 2023;14.

86. Fayers PM, Hand DJ, Bjordal K, Groenvold M. Causal indicators in quality of life research. Qual Life Res. 1997;6:393–406.

87. Streiner DL. Being Inconsistent About Consistency: When Coefficient Alpha Does and Doesn't Matter. J Pers Assess. 2003;80:217–22.

88. Floyd FJ, Widaman KF. Factor analysis in the development and refinement of clinical assessment instruments. Psychol Assess. 1995;7:286–99.

89. Skogestad IJ, Kottorp A, Larsson P, Moen TM, Gay CL, Borge CR, et al. Development and evaluation of the Norwegian Fatigue Characteristics and Interference Measure (FCIM) for stroke survivors: cognitive interviews and Rasch analysis. Qual Life Res. 2023;32:3389–401.

90. Harrison CJ, Plessen CY, Liegl G, Rodrigues JN, Sabah SA, Beard DJ, et al. Item response theory assumptions were adequately met by the Oxford hip and knee scores. J Clin Epidemiol. 2023;158:166–76.

91. Carlberg Rindestig F, Wiberg M, Chaplin JE, Henje E, Dennhag I. Psychometrics of three Swedish physical pediatric item banks from the Patient-Reported Outcomes Measurement Information System (PROMIS)®: pain interference, fatigue, and physical activity. J Patient-Reported Outcomes. 2021;5:105.

92. Mokkink LB, Eekhout I, Boers M, van der Vleuten CP, de Vet HC. Studies on Reliability and Measurement Error of Measurements in Medicine – From Design to Statistics Explained for Medical Researchers. Patient Relat Outcome Meas. 2023;Volume 14:193–212.

93. van Stiphout L, Rolfes J, Waardenburg S, Kimman M, Guinand N, Pérez Fornos A, et al. Construct validity and reliability of the Bilateral Vestibulopathy Questionnaire (BVQ). Front Neurol. 2023;14.

94. Sandseter EBH, Sando OJ, Kleppe R, Lorås H, Storli L. Assessment of psychometric properties and measurement invariance of the sensation seeking scale for children in a Norwegian sample. Front Psychol. 2024;15.

95. Obbarius A, Klapproth CP, Liegl G, Christmann PM, Schneider U, Fischer F, et al. Measuring PROMIS pain interference in German patients with chronic conditions: calibration, validation, and cross-cultural use of item parameters. Qual Life Res. 2023;32:2839–52.

96. Lorenz T, Hagitte L, Prasath PR. Validation of the revised Compound PsyCap Scale (CPC-12R) and its measurement invariance across the US and Germany. Front Psychol. 2022;13.

97. Elsman EBM, Flens G, de Beurs E, Roorda LD, Terwee CB. Towards standardization of measuring anxiety and depression: Differential item functioning for language and Dutch reference values of PROMIS item banks. PLoS One. 2022;17:e0273287.

98. de Vet HCW, Terwee CB, Mokkink LB, Knol DL. Measurement in Medicine. Cambridge University Press; 2011.

99. Stoker AMH, Gruters A, Loon MCM van der E, Postulart D, Czuber-Dochan W, Gilissen LPL. Translation, validation and psychometric properties of the Dutch version of the Inflammatory Bowel Disease-Fatigue (IBD-F) self-assessment scale. J Patient-Reported Outcomes. 2023;7:108.

100. Schougaard LMV, de Thurah A, Bech P, Hjollund NH, Christiansen DH. Test-retest reliability and measurement error of the Danish WHO-5 Well-being Index in outpatients with epilepsy. Health Qual Life Outcomes. 2018;16:175.

101. Vach W. The dependence of Cohen's kappa on the prevalence does not matter. J Clin Epidemiol. 2005;58:655–61.

102. Shrout PE, Fleiss JL. Intraclass correlations: Uses in assessing rater reliability. Psychol Bull. 1979;86:420–8.

103. van Leeuwen LM, Mokkink LB, Kamm CP, de Groot V, van den Berg P, Ostelo RWJG, et al. Measurement properties of the Arm Function in Multiple Sclerosis Questionnaire (AMSQ): a study based on Classical Test Theory. Disabil Rehabil. 2017;39:2097–104.

104. Avila MA, Arruda GT de, Godoy AG de, Driusso P. Translation, cross-cultural adaptation to Brazilian Portuguese and measurement properties of the WaLIDD score. Rev Bras Ginecol e Obs / RBGO Gynecol Obstet. 2024;46.

105. Liu S, Liang J, Xu N, Mai S, Wang Q, Zeng L, et al. Reliability and validity of simplified Chinese version of the Italian spine youth quality of life questionnaire in adolescents with idiopathic scoliosis. BMC Musculoskelet Disord. 2021;22:568.

106. Smit EB, Bouwstra H, Roorda LD, van der Wouden J (Hans) C, Wattel E (Lizette) M, Hertogh CMPM, et al. A Patient-Reported Outcomes Measurement Information System Short Form for Measuring Physical Function During Geriatric Rehabilitation: Test-Retest Reliability, Construct Validity, Responsiveness, and Interpretability. J Am Med Dir Assoc. 2021;22:1627-1632.e1.

107. Streiner DL, Norman GR. Health Measurement Scales. Oxford University Press; 2008.

108. Versi E. "Gold standard" is an appropriate term. BMJ. 1992;305:187–187.

109. Prinsen CAC, Bouter LBMLM, Patrick JADL. COSMIN guideline for systematic reviews of patient-reported outcome measures. Qual Life Res. 2018;27:1147–57.

110. Le Carré J, Luthi F, Burrus C, Konzelmann M, Vuistiner P, Léger B, et al. Development and Validation of Short Forms of the Pain Catastrophizing Scale (F-PCS-5) and Tampa Scale for Kinesiophobia (F-TSK-6) in Musculoskeletal Chronic Pain Patients. J Pain Res. 2023;Volume 16:153–67.

111. Moore D, McCabe G, Craig B. Introduction to the Practice of Statistics. 2016.

112. Clover K, Lambert SD, Oldmeadow C, Britton B, Mitchell AJ, Carter G, et al. Convergent and criterion validity of PROMIS anxiety measures relative to six legacy measures and a structured diagnostic interview for anxiety in cancer patients. J Patient-Reported Outcomes. 2022;6:80.

113. Dochat C, Afari N, Arigo D. Psychometric validation of the celiac disease-specific quality of life survey (CD-QOL) in adults with celiac disease in the United States. Qual Life Res. 2023;32:2195–208.

114. Mokkink LB, Terwee CB, Knol DL, Stratford PW, Alonso J, Patrick DL, et al. The

COSMIN checklist for evaluating the methodological quality of studies on measurement properties: A clarification of its content. BMC Med Res Methodol. 2010;10:22.

115. Mokkink L, Terwee C, de Vet H. Key concepts in clinical epidemiology: Responsiveness, the longitudinal aspect of validity. J Clin Epidemiol. 2021;140:159–62.

116. Lokhorst MM, Horbach SER, Waner M, O TM, van der Vleuten CJM, Spuls PI, et al. Responsiveness of quality of life measures in children with peripheral vascular malformations: The OVAMA project. JPRAS Open. 2021;27:70–9.

117. Zhang K, Wu J, Su Z, Ma Y, Shi Q, Barchi LC, et al. The SUPER reporting guideline suggested for reporting of surgical technique: explanation and elaboration. Gland Surg. 2023;12:749–66.

118. Mallinson T, Kozlowski AJ, Johnston M V., Weaver J, Terhorst L, Grampurohit N, et al. Rasch Reporting Guideline for Rehabilitation Research (RULER): the RULER Statement. Arch Phys Med Rehabil. 2022;103:1477–86.

119. Flynn KE, Dew MA, Lin L, Fawzy M, Graham FL, Hahn EA, et al. Reliability and construct validity of PROMIS® measures for patients with heart failure who undergo heart transplant. Qual Life Res. 2015;24:2591–9.

120. Rysstad T, Grotle M, Klokk LP, Tveter AT. Responsiveness and minimal important change of the QuickDASH and PSFS when used among patients with shoulder pain. BMC Musculoskelet Disord. 2020;21:328.
