# Supplementary material for: Cosmin reporting guideline for studies on measurement properties of patient-reported outcome measures and its explanation and elaboration document: translation into Brazilian Portuguese
Source: Qual Life Res. 2026 Jun 5;35(7):165. doi: 10.1007/s11136-026-04282-0 (PMC13241396; doi:10.1007/s11136-026-04282-0)
Supplement: Supplementary file 3 — Supplementary Material 3 [file 11136_2026_4282_MOESM3_ESM.docx]

**Step 1**

Forward translation

(ChatGPT + Human translator)

**Step 2**

Synthesis

(Consensus between authors)

**Step 3**

Back-translation

(ChatGPT)

Discrepancy identification (semantic, idiomatic, experiential, conceptual)

Resolution process (Authors consensus + refinement if needed)

**Step 4**

Expert committee review

(n = 5, independent review)

Final version + COSMIN developer approval

**Figure S1.** Flow diagram of the translation process, including forward translation, synthesis, back-translation, discrepancy identification and classification, resolution procedures, expert committee review, and final approval.
