# Supplementary material for: Cosmin reporting guideline for studies on measurement properties of patient-reported outcome measures and its explanation and elaboration document: translation into Brazilian Portuguese
Source: Qual Life Res. 2026 Jun 5;35(7):165. doi: 10.1007/s11136-026-04282-0 (PMC13241396; doi:10.1007/s11136-026-04282-0)
Supplement: Supplementary file 4 — Supplementary Material 4 [file 11136_2026_4282_MOESM4_ESM.docx]

**Table S1.** Discrepancies between the back-translations and the original version of the COSMIN Reporting Guideline 2.0.

| **Item** | **Back-translated by a human** | **Back-translated by AI** | **Original** | **Type of discrepancy** | **Resolution decision** | **Decision made by** |
| --- | --- | --- | --- | --- | --- | --- |
| T1 | To measure | To assess | To measure | Semantic | Maintained as equivalent to original meaning | Authors consensus |
| A4 | Key results | Main results | Main results | Semantic | Standardized to match original wording | Authors consensus |
| I2 | developed | developed | designed for | Conceptual | Revised to preserve original intent (“designed for”) | Authors consensus |
| I3 | Current state of knowledge and justification;  Not known | Knowledge Status & Rationale;  Unknown | State of knowledge & Rationale;  Not known | Semantic | Adjusted to align with original terminology | Authors consensus |
| GM3 | About;  Version of the PROM that is being studied;  Mode of administration | On;  Studied version;  Administration mode | About;  PROM version being studied;  Mode of administration | Semantic | Standardized wording to match original structure | Authors consensus |
| GM4 | Groups being compared | Compared groups | Groups being compared | Semantic | Maintained as equivalent | Authors consensus |
| GM5 | Provide all time points for all measurements | Provide all time points | Provide all time points for all measurements | Semantic | Revised for completeness (“for all measurements”) | Authors consensus |
| GM6 | Rationale for sample size | Sample Size Justification | Justification for sample size | Conceptual | Revised to reflect original concept (“justification”) | Authors consensus |
| GM7 | See boxes specific to measurement properties;  Describe;  Package used and the version | See specific boxes for each;  Cite;  Software and version used | See measurement properties specific boxes;  Name;  Package used and the version | Conceptual | Reconstructed to preserve full original meaning | Authors consensus |
| GM8 | to dealing with | used to handle | for dealing with | Idiomatic | Standardized to original expression (“dealing with”) | Authors consensus |
| GM9 | Specify any analyses that were not planned and their rationale | Specify analyses that were not pre-planned and provide justification | Specify analyses that were unplanned and their rationale | Semantic | Adjusted to match original phrasing | Authors consensus |
| GR4 | See boxes specific to measurement properties | See specific boxes for each measurement property | See measurement properties specific boxes | Semantic | Standardized to original wording | Authors consensus |
| DC1 | Evidence of measurement property;  Whether | Evidence of measurement property;  Whether | Measurement property evidence;  If | Semantic | Adjusted to maintain original structure | Authors consensus |
| DC4 | Generalization | Generalizability | Generalizability | Conceptual | Revised to reflect correct concept (“If”) | Authors consensus |
| DC5 | Changes to the instrument | Instrument Modifications | Instrument changes | Semantic | Standardized wording | Authors consensus |
| CV2 | Scope;  Providers | Comprehensiveness;  Professionals | Comprehensiveness;  Professionals | Conceptual | Revised to match original construct (“comprehensiveness”) | Authors consensus |
| CV3 | Understanding | Comprehensibility | Comprehensibility | Semantic | Standardized to original term (“comprehensibility”) | Authors consensus |
| SV2 | If other approaches were used, please provide details of the methods. | Provide details of the methods if other approaches were used. | Provide details of the methods if other approaches were used. | Semantic | Adjusted to match original phrasing | Authors consensus |
| ICI1 | Absence | Absence | Lack | Conceptual | Revised to reflect original concept (“lack”) | Authors consensus |
| CCV4 | DIF logistic regression analyses | logistic regression DIF analyses | logistic regression DIF analyses | Semantic | Maintained as equivalent | Authors consensus |
| R1 | For assuming | To support | For assuming | Conceptual | Revised to match original intent (“for assuming”) | Authors consensus |
| R3 | Purposefully;  Across | Intentionally;  Between | Purposefully;  Across | Semantic | Standardized to original wording | Authors consensus |
| ConV1 | Formulate | Formulate | State | Conceptual | Revised to reflect original meaning (“state”) | Authors consensus |
| ConV3 | Agrees with | Supports the | In accordance with | Semantic | Standardized to original expression | Authors consensus |

AI: Artificial Intelligence. DIF: Differential Item Functioning. PROM: Patient-Reported Outcome Measure.
